# Benzo[*d*]imidazole–Naphthalen-Arylmethanone Regioisomers as CB_1_ Ligands: Evaluation of Agonism via an Indirect Cytotoxicity-Based Approach

**DOI:** 10.3390/ijms26209986

**Published:** 2025-10-14

**Authors:** Analia Young Hwa Cho, Renato Burgos Ravanal, Valeria Zuñiga Salazar, Marco Mellado, Marcos Lorca, David Pessoa-Mahana, Jaime Mella, Germán Günther Sapunar, Javier Romero-Parra

**Affiliations:** 1 Organic Chemistry and Physical Chemistry Department, Faculty of Chemical and Pharmaceutical Sciences, Universidad de Chile, Olivos 1007, Santiago 7820436, Chile; young.hwa@ciq.uchile.cl (A.Y.H.C.); renatoburgosravanal@gmail.com (R.B.R.); vale-paz45@hotmail.com (V.Z.S.); ggunther@ciq.uchile.cl (G.G.S.); 2 Centro de Investigación en Ingeniería de Materiales, Universidad Central de Chile, Santiago 8330507, Chile; marco.mellado@ucentral.cl; 3 Carrera de Química y Farmacia, Facultad de Ciencias de la Vida, Universidad Viña del Mar, Viña del Mar 2520000, Chile; marcos.lorca@uvm.cl; 4 Pharmacy Department, School of Pharmacy, Faculty of Chemistry and Pharmacy, Pontificia Universidad Católica de Chile, Vicuña Mackenna 4860, Santiago 7820436, Chile; cpessoa@uc.cl; 5 Instituto de Química, Facultad de Ciencias, Universidad de Valparaíso, Av. Gran Bretaña 1111, Valparaíso 2360102, Chile; jaime.mella@uv.cl; 6 Centro de Investigación, Desarrollo e Innovación de Productos Bioactivos (CInBIO), Universidad de Valparaíso, Av. Gran Bretaña 1111, Valparaíso 2360102, Chile

**Keywords:** cannabinoids, benzo[*d*]midazole, binding, cytotoxic studies, indirect agonism, docking

## Abstract

CB_1_ agonist compounds may be potential drug candidates for the treatment of gliomas, as they have been shown to inhibit tumor cell proliferation, induce apoptosis, and reduce angiogenesis in various preclinical models. Their ability to modulate the endocannabinoid system suggests a promising therapeutic approach for targeting glioma growth and progression. Herein, we report the design, synthesis, biological studies, and bioinformatics assays of novel benzo[*d*]imidazole–naphthalen-arylmethanone regioisomers with affinity for the CB_1_ receptor, as well as propose an indirect methodology to evaluate their presumed CB_1_ agonist activity. Compounds that showed a propensity for binding to the CB_1_ receptor were regioisomers **4d**, **5b**, **5e**, **5f**, and **5f′**. Likewise, derivatives that displaced more than 50% of the radioligand [^3^H]CP-55940 at the CB_1_ receptor were subjected to in vitro viability experiments. Compounds **4d**, **5b**, **5e**, and **5f′** showed toxicity against U87MG cells (malignant glioma) in a considerable percentage. Notably, compound **5f′** showed CB_1_ affinity, with a K_i_ of 2.12 µM, and was selectively toxic to U87MG cells, which highly express the CB_1_ receptor, while exhibiting no toxicity toward the healthy HEK293 cell line, which expresses both cannabinoid receptors at negligible levels. Docking studies at the CB_1_ orthosteric site indicate that **5f′** forms π-π interactions, a T-shaped interaction, and hydrogen bonding through the oxygen atom of the furan ring. Biologically, our experimental indirect model-based on a simple viability assay is supported by well-established evidence that activation of CB_1_ and CB_2_ receptors by agonists induces cell death and inhibits tumor cell growth. Structurally, we conclude that the presence of a furan ring at the 2-position of the benzo[*d*]imidazole core is beneficial for the development of new ligands with potential CB_1_ agonist activity.

## 1. Introduction

The endocannabinoid system (ECS) is a ubiquitous network responsible for information and transduction. It involves lipid signaling neurotransmitters such as anandamide (AEA) and 2-arachidonoylglycerol (2-AG), among others [1,2]. Additionally, it encompasses cannabinoid receptor type 1 (CB_1_R) and cannabinoid receptor type 2 (CB_2_R), as well as the ligand-metabolizing enzymes such as diacylglycerol lipase (DAGL), fatty acid amide hydrolase (FAAH), and monoacylglycerol lipase (MAGL) [3,4]. Within the ECS, it has been well established that CB_1_R is widely distributed throughout the central nervous system (CNS), while CB_2_R can be found predominantly in the immune system cells (monocytes, macrophages, B lymphocytes, and T lymphocytes) [5,6]. Both CB_1_ and CB_2_ receptors are mostly coupled to heterotrimeric G_i/o_ protein, and their activation by endogenous or exogenous ligands leads to the inhibition of adenylate cyclase and the following reduction in cyclic AMP (cAMP) accumulation in many tissues and experimental models [3]. Furthermore, extensive research has demonstrated that the activation of CB_1_ and CB_2_ receptors by specific agonists promotes cell death and exerts inhibitory effects on the proliferation of tumor cells [7,8,9,10,11,12,13]. Thus, the activation of this system may provide valuable insights into neoplasm pathologies, given the established relationship between cannabinoid receptor activation, agonist behavior, and the regulation of cell viability.

In recent years, there has been great interest in developing different synthetic cannabinoid ligands that target both cannabinoid receptors, which has spread due to the wide range of pathological conditions where they are involved. As a result, numerous synthetic agents have been developed in an attempt to find new compounds capable of treating different pathologies, including inflammation, pain management, cardiovascular regulation, metabolic disorders, neurodegenerative diseases, and cancer [14,15,16]. Among the ligands developed to modulate the ECS are classical cannabinoids (*CC*s) and non-classical cannabinoids (*NCC*s), such as (-)-*trans*-Δ^9^-tetrahydrocannabinol (Δ^9^-THC) and cannabidiol (CBD) [17,18]. Additionally, heterocyclic agents have been reported, with aminoalkylindoles (AAIs) and diarylpyrazoles being the most established examples [19]. Some heterocyclic cannabinoid ligands are illicitly marketed as ‘Spice’ or ‘K2’ [20,21]. Most contain indole or indazole cores [21], though azaindole, γ-carbolinone, carbazole, and benzimidazole (e.g., FUBIMINA) scaffolds have also been reported [20,22]. Despite their illicit status, many show promising experimental results, encouraging the synthesis of new heterocyclic cannabinoids with pharmacological and therapeutic potential [20].

Our group has been focused on developing small molecules as chemical modulators of the ECS. Specifically, we have focused our efforts on the synthesis and rational design of benzo[*d*]imidazole-based molecules that target the cannabinoid receptors, as well as performed molecular docking studies and QSAR analysis of our reported derivatives [23,24,25,26,27,28]. Since it is widely supported that the benzo[*d*]imidazole core represents a prominent scaffold in drug discovery and has showcased its versatility as a platform for the development of molecules with a wide range of pharmacological properties [29,30,31], herein we report the synthesis, biological evaluation and docking assays of novel 5-chloro(or fluoro)benzo[*d*]imidazole and 6-chloro(or fluoro)benzo[*d*]imidazole regioisomers as cannabinoid ligands substituted at position 2- with different five- or six-membered aromatic heterocycles and bearing a naphthoyl moiety at the nitrogen 1- (-N1) based on the promising outcomes obtained from certain known cannabimimetics, such as the AAIs, which also possess the naphthalene framework in their structures.

The aim of our study was to develop new agents as tools for investigating cannabinoid receptors, thereby contributing to the understanding of the structure-activity relationship between the benzo[*d*]imidazole core and these receptors. This, in turn, would help to identify the structural features essential for designing and synthesizing novel selective cannabinoid ligands. Hence, all synthesized compounds were evaluated for their binding affinity to human recombinant CB_1_ and CB_2_ cannabinoid receptors. Five derivatives exhibited selective binding to the CB_1_ receptor, with inhibition constants (K_i_) in the micromolar range. Furthermore, given that several studies have reported cannabinoid agonists inducing cell death [7,8,9,10,11,12,13], viability assays were performed for the CB_1_/CB_2_ agonist WIN-55212-2, the CB_1_ inverse agonist/antagonist AM251, and the CB_2_ inverse agonist/antagonist AM630 across three different cell lines serving as experimental models. Our aim was to establish a plausible indirect methodology to suggest a correlation between the activity of cannabinoid ligands and the reduction in cell viability, potentially mediated by an agonist-induced, cannabinoid receptor–dependent cell death mechanism. Thus, human embryonic kidney 293 (HEK293), which shows low expression of both cannabinoid receptors [32], neoplastic glioblastoma cells (U87MG), which highly express the CB_1_ receptor [33], and acute promyelocytic leukemia cells (HL-60), which exclusively express CB_2_ receptors [6] were chosen as the experimental models. Results showed that WIN-55212-2 was exclusively cytotoxic toward U87MG and HL-60 cells, whereas AM251 and AM630 were non-toxic in all three selected cell lines mentioned above. Moreover, when WIN-55212-2 was assayed against increasing concentrations of the CB_1_ inverse agonist/antagonist AM251 in U87MG cells, its toxicity decreased, leading to increased cell viability. This suggests that the cytotoxic effect of WIN-55212-2 may be mediated through cannabinoid receptor activation. Finally, using our proposed indirect methodology for evaluating cytotoxic cannabinoid agonists, regioisomers that displaced more than 50% of the radioligand [^3^H]CP-55940 at the CB_1_ receptor in binding experiments were subjected to in vitro viability assays in HEK293, U87MG, and HL-60 cell lines. Among these, the benzo[*d*]imidazole derivative **5f′** stood out.

## 2. Results and Discussion

### 2.1. Subsection Design Criteria to Develop CB Ligands

**Figure 1:**
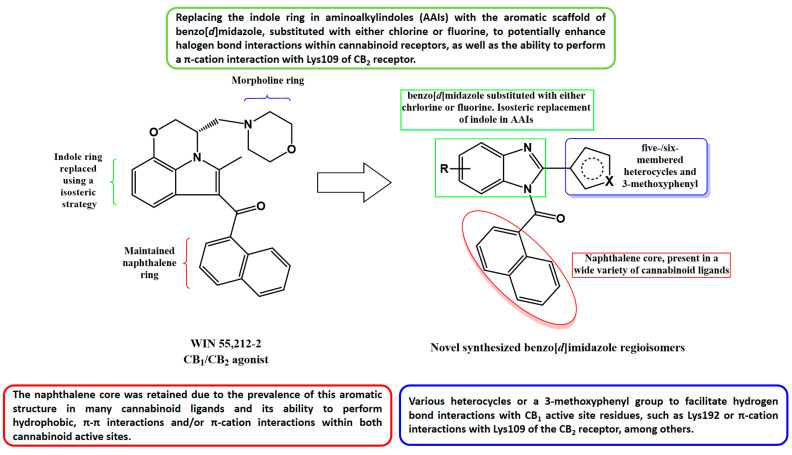
Design strategy for obtaining novel benzo[*d*]imidazoles as cannabinoid ligands from the aminoalkylindole WIN 55212-2.

Extensive research on the role of Lys192 suggests that mutations to this amino acid result in decreased affinities for certain cannabinoid ligands, such as CP-55940, HU-210, and anandamide [34,35,36,37], implying that this residue could play a key role in ligand binding. In this sense, for the CB_1_ receptor, we have set out to corroborate whether Lys192 is important by developing 24 novel benzo[*d*]imidazole compounds with the ability to interact with this residue. Our compounds feature an isosteric replacement of the indole ring in AAIs by benzo[*d*]imidazole substituted with either chlorine or fluorine in order to potentially facilitate hydrogen bond or halogen bond interactions with the amino acids of the active site of the CB_1_ receptor (Figure 1). Additionally, position 2 of the benzo[*d*]imidazole ring of each compound has been functionalized with different heterocycles or with a 3-methoxyphenyl group to enable hydrogen bond interactions with the residues of the CB_1_ active site (including possibly Lys192), as all substituents possess hydrogen atom acceptors. The naphthalene core was maintained since many cannabinoid ligands exhibit this aromatic core.

On the other hand, for the CB_2_ receptor, the structural features of the 24 proposed benzo[*d*]imidazoles could potentially result in favorable affinity outcomes since all derivatives could be considered as AAI analogs and may engage in interactions similar to those observed with WIN-55212-2 at the CB_2_ active site, as reported by Xing et al. in their cryo-EM/WIN-55212-2 complex study [38]. Therefore, the naphthalene core could lead to π-π interactions with Phe91 and Phe94, as well as hydrophobic interactions with Phe87, His95, Pro184, and Phe281. The benzo[*d*]imidazole frameworks could also perform π-π interactions with Phe117 and Trp258, but also hydrophobic interactions with the residues of Ile110, Val113, and Phe183, among others. Moreover, the various heterocycles and the 3-methoxyphenyl group located at position 2- of the halogenated benzo[*d*]imidazoles might interact with hydrophobic residues similarly to the morpholine nucleus observed in WIN-55212-2 [38]. Moreover, we previously reported a potentially pivotal π-cation interaction between Lys109 and different benzo[*d*]imidazole compounds [25]; thereupon, the presented derivatives were designed in order to bear different aromatic rings, with the intention of performing this type of interaction with Lys109 within the active site of the CB_2_ receptor.

Taking into account the aforementioned, Figure 1 summarizes the design strategy applied in the development of the new benzo[*d*]imidazole derivatives.

### 2.2. Chemistry

**Scheme 1:**
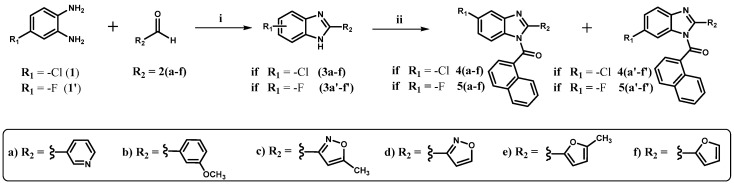
Synthesis of (5-chloro-2-(aryl)-1*H*-benzimidazol-1-yl)(naphtalen-1-yl)methanone, (5-fluoro-2-(aryl)-1*H*-benzimidazol-1-yl)(naphtalen-1-yl)methanone and its regioisomers. Reagents and conditions: (**i**) for aldehydes: DMF, MgCl_2_·6H_2_O (10mol%,) rt, 24 h; for carboxylic acids: polyphosphoric acid (PPA), 180 °C, 3 h. (**ii**) 1-naphthoyl chloride, NaH, N_2_, dry THF, rt, 30 min. The framed structures show R_2_, which represents the substituents at position 2 of the benzo[*d*]imidazole core, derived from the aldehydes or carboxylic acids used, as indicated in the reagents and conditions.

(5-chloro-2-(aryl)-1*H*-benzo[*d*]imidazol-1-yl)(naphtalen-1-yl)methanone (**4a-f**) and its regioisomers (6-chloro-2-(aryl)-1*H*-benzo[*d*]imidazol-1-yl)(naphtalen-1-yl)methanone [39] (**4a′-f′**), as well as the (5-fluoro-2-(aryl)-1*H*-benzo[*d*]imidazol-1-yl)(naphtalen-1-yl)methanone (**5a-f**) and its isomers (6-fluoro-2-(aryl)-1*H*-benzo[*d*]imidazol-1-yl)(naphtalen-1-yl)methanone (**5a′-f′**) were synthesized by first obtaining different chlorinated and fluorinated 2-aryl-1*H*-benzo[*d*]imidazoles **3a-f** and **3a′-f′**, respectively [40,41,42,43]. Therefore, 4-chlorobenzene-1,2-diamine (**1**) and 4-fluorobenzene-1,2-diamine (**1′**) were subjected to condensation reactions with different aromatic heterocyclic aldehydes or aromatic heterocyclic carboxylic acids as required (Scheme 1). Subsequently, the chlorinated and fluorinated 2-aryl-1*H*-benzo[*d*]imidazoles (**3a-f** and **3a′-f′**) were reacted with 1-naphthoyl chloride for 30 min in order to yield the aiming acylated compounds at the nitrogen atom (position -N1) of benzo[*d*]imidazole cores (Scheme 1).

Considering the commercial availability of reagents, 2-aryl-1*H*-benzo[*d*]imidazole intermediates **3c**, **3d**, **3c′**, and **3d′** were synthesized using 5-Methylisoxazole-3-carboxylic acid and isoxazole-3-carboxylic acid instead of their corresponding aldehydes, as was the case for the synthesis of compounds **3a-b**, **3e-f**, **3a′-b′,** and **3e′-f′,** where aldehydes were employed. When carboxylic acids are used to yield benzo[*d*]imidazoles, polyphosphoric acid (PPA) is required at 180 °C for approximately 3 h to obtain the heterocycles. Furthermore, reactions using carboxylic acids to yield 2-aryl-1*H*-benzo[*d*]imidazoles do not need the acids to be anhydrous, making these assays highly advantageous and straightforward procedures [44].

Nucleophilic acyl substitution of the chlorine atom in 1-naphthoyl chloride with **3a-f** and **3a′-f′** was performed using the strong base sodium hydride (NaH). The aforementioned base was used to yield the chlorinated and fluorinated 2-aryl-1*H*-benzimidazole anions by abstracting the hydrogen atom from the nitrogen at position 1- of the benzo[*d*]imidazole core, increasing its nucleophilicity. This approach significantly reduced the time required to react with the carbonyl group of 1-naphthoyl chloride to obtain the desired derivatives in lieu of using weak bases such as triethylamine or pyridine. Nonetheless, starting materials were not completely consumed, and no yield improvements were obtained even when reactions were carried out for more than 30 min.

The acylation reactions (step ii, Scheme 1) gave rise to two regioisomers, given the tautomeric forms that the benzo[*d*]imidazole ring displays. Therefore, chlorine and fluorine atoms can be borne at position 5- or -6 of 2-aryl-1*H*-benzo[*d*]imidazole rings when they are finally acylated. Given the above, the obtained regioisomers were separated owing to the fact that they exhibited different retardation factors (R_f_), allowing for effortless separations. Characterization and identification in order to define the exact structure of each regioisomer were established on the basis of their nuclear magnetic resonance spectral properties (^1^H, ^13^C, DEPT-135, COSY, HSQC, and HMBC). As an example, spectral features of (5-fluoro-2-(5-methylisoxazol-3-yl)-1*H*-benzo[*d*]imidazol-1-yl)(naphthalen-1-yl)methanone (derivative **5c**) and its regioisomer (6-fluoro-2-(5-methylisoxazol-3-yl)-1*H*-benzo[*d*]imidazol-1-yl)(naphthalen-1-yl)methanone (regioisomer **5c′**) are discussed, aiming to display the conducted analysis that led to the identification of each isomer.

**Figure 2:**
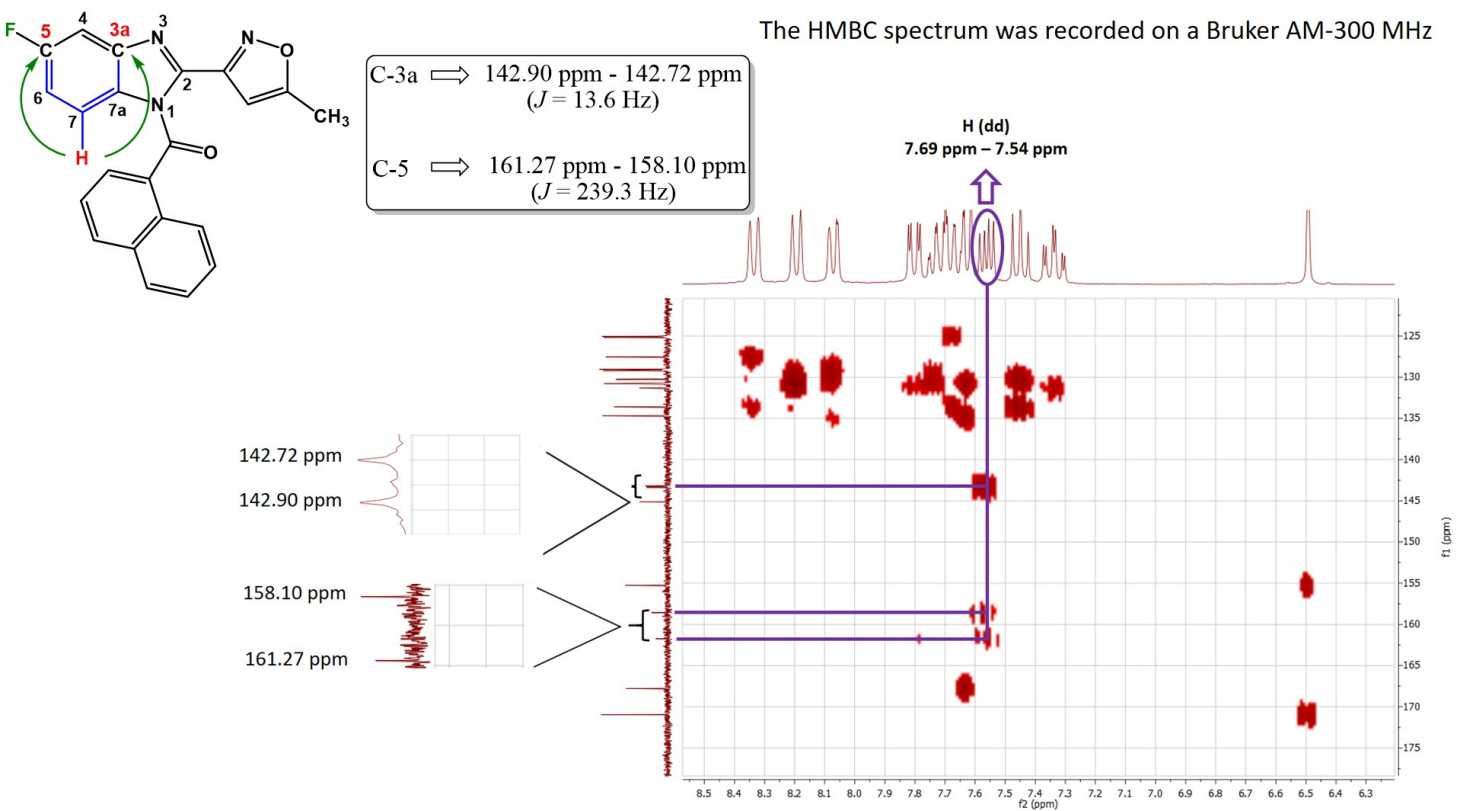
Heteronuclear Multiple Bond Correlation spectrum (HMBC) of regioisomer **5c**. Main interactions are highlighted through an identified hydrogen bond in its structure with the involved carbon atoms, which corroborates the halogen position on the benzo[*d*]imidazole ring. The HMBC spectrum was recorded on a Bruker AM-300 MHz.

Figure 2 shows the HMBC spectrum (Heteronuclear Multiple Bond Correlation) of compound **5c** bearing the fluorine atom at position 5- of the benzo[*d*]imidazole heterocycle. The accurate position of the halogen on the benzo[*d*]imidazole ring was established by identifying the red-highlighted hydrogen atom in Figure 2 due to its multiplicity. This atom corresponds to a doublet of doublets (dd) owing to its ortho coupling with a neighboring hydrogen and with the fluorine active magnetic nucleus. In addition, the observed integral value of 1 further confirms its presence over carbon seven of the heterocyclic aromatic ring, as well as its shifts of 7.54 ppm–7.59 ppm. Subsequently, this red-depicted hydrogen must correlate on the HMBC spectrum with carbon 5 (**C-5**) and carbon 3a (**C-3a**). Both atoms were identified based on their chemical shifts, multiplicities (doublets), and the corresponding coupling constants (*J*). The coupling constant for **C-5**, where the fluorine atom is attached, was determined to be 239.3 Hz. For **C-3a**, located at a three-bond distance relative to the fluorine atom (in the meta position), the coupling constant was found to be 13.6 Hz. The latter confirms the exact position of the halogen and therefore aligns to regioisomer **5c**.

**Figure 3:**
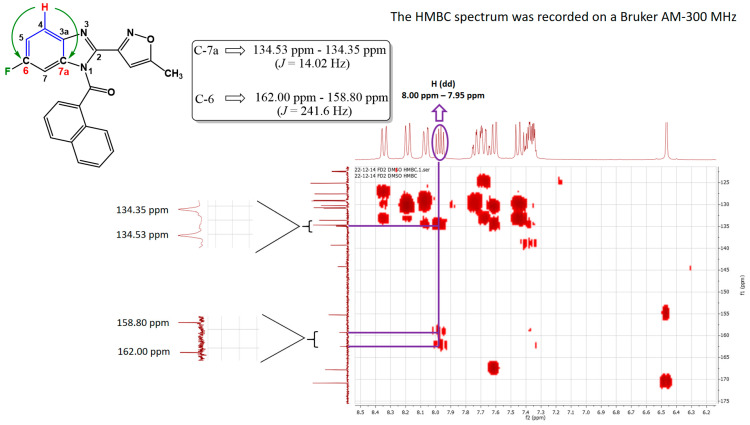
Heteronuclear Multiple Bond Correlation spectrum (HMBC) of regioisomer **5c′**. Main interactions are highlighted through an identified hydrogen bond in its structure with the involved carbon atoms, which corroborates the halogen position on the benzo[*d*]imidazole ring. The HMBC spectrum was recorded on a Bruker AM-300 MHz.

On the other hand, regioisomer **5c′** was also determined employing a strategy based on its two-dimensional spectral features. In this sense, upon analysis of the HMBC spectrum, a doublet of doublets (dd) is observed with an integral value of 1, spanning chemical shifts from 8.00 ppm to 7.95 ppm (red-highlighted hydrogen atom attached to carbon 4, Figure 3). Just like the previous case (isomer **5c**), the dd hydrogen atom should correlate with carbon 6 (**C-6**), where the fluorine atom is attached, with a coupling constant (*J* value) of 241.5 Hz. Additionally, it should correlate with carbon 7a (**C-7a**) with a coupling constant of 14.02 Hz, as it is located at three bonds of distance from the fluorine atom. These correlations are illustrated in Figure 3.

Once 5-chloro(or fluoro)benzo[*d*]imidazole-naphthalen-arylmethanone and 6-chloro(or fluoro)benzo[*d*]imidazole-naphthalen-arylmethanone regioisomers were obtained and characterized, binding assays over CB_1_ and CB_2_ receptors were performed, as well as MTT experiments [3-(4,5-dimethylthiazol-2-yl)-2,5-diphenyltetrazolium bromide] over different lines depending on whether they express cannabinoid receptors (CB_1_ and CB_2_) or not.

### 2.3. Biological Evaluations: Binding Assays and Cell Viability Experiments

All acylated benzo[*d*]imidazole regioisomers were evaluated for their ability to bind to human recombinant CB_1_ and CB_2_ receptors. Table 1 shows the half-maximal inhibitory concentration (IC_50_), the inhibition constant (Kᵢ), and the maximum percentage of displacement at 10 μM, corresponding to the highest percentage of displacement observed at this concentration for the reference radioligand [^3^H]CP-55940.

For binding experiments, the reference radioligand [^3^H]CP-55940 was used at a single concentration of 0.5 nM (Kd for CB_1_ = 0.16 nM and Kd for CB_2_ = 0.18 nM) in the presence of increasing concentrations of the synthesized regioisomers. Nonlinear regression was then applied to determine the 100% (total) and 0% (nonspecific) plateaus. For each regioisomer, the bottom plateau was observed as a constant value corresponding to nonspecific binding, defined as the binding measured in the presence of 10 μM unlabeled WIN-55212-2, a concentration sufficient to displace at least 84% of the specific radioligand binding to cannabinoid receptors.

Based on the displacement of the reference radioligand [^3^H]CP55940, compounds exhibiting more than 50% at 10 μM were considered significant and subjected to further analysis. For these selected molecules, IC_50_ values were experimentally determined, and the corresponding K_i_ values were calculated using the Cheng–Prusoff equation, providing a quantitative measure of binding affinity [45]. This approach ensures that both the observed efficacy (Max % displacement) and the binding potency (IC_50_ and Kᵢ) are considered in identifying optimal compounds. The combined evaluation of these parameters allowed us to prioritize compounds for subsequent cytotoxicity studies.

Compounds were prone to binding to the CB_1_ receptor, highlighting regioisomers **4d**, **5b**, **5e**, **5f,** and **5f′,** which exhibited percentages of displacement over 55% and IC_50_ values of 7.58 μM, 6.23 μM, 7.4 μM, 6.03 μM, and 8.65 μM, respectively (Table 1). Inhibition constants (K_i_) of each compound can also be seen in Table 1. Although some derivatives showed acceptable displacements over the CB_2_ receptor, such as **4a′**, **4e′**, **4f′**, **5b′**, **5d** and **5e′,** only compound **4b′** exhibited the best displacement at 10 μM (58%) over this receptor, showing an IC_50_ = 6.76 μM and a K_i_ = 4.46 μM (Table 1). The above confirms that developed regioisomers behave as better CB_1_ ligands than CB_2_.

The preceding results indicate that binding to the CB_1_ receptor is favored with fluorine substitution on the benzo[*d*]imidazole, as demonstrated by compounds **5b**, **5e**, **5f,** and **5f′**. On the other hand, chlorine substitution tends to abolish binding to the CB_1_ receptor, except for compound **4d**, which still demonstrated CB_1_ binding. Instead, chlorine substitution mostly favors CB_2_ receptor binding, as demonstrated by compounds **4a′**, **4b′**, **4e′,** and **4f′**, although the increase in affinity is modest. It is worth noting that compounds binding to the CB_1_ receptor are mostly regioisomers at the 5- position of the halogen atom on the benzo[*d*]imidazole ring, while compounds showing slight affinity for the CB_2_ receptor are mostly regioisomers at the 6- position of the halogen atom on the benzo[*d*]imidazole core. Regarding the nature of the substituent at position 2- of the benzo[*d*]imidazole framework, both five-membered and six-membered heterocycles were tolerated.

Once the radioligand displacement parameters were determined, and consequently, information on the affinity of our benzo[*d*]imidazole-naphthalen-arylmethanone regioisomers was obtained, we proceeded to carry out cell viability (cytotoxicity) assays. This experimental approach was selected based on several studies indicating that activation of CB_1_ and CB_2_ receptors by agonists induces cell death and inhibits tumor cell growth [7,8,9,10,11,12,13].

**Figure 4:**
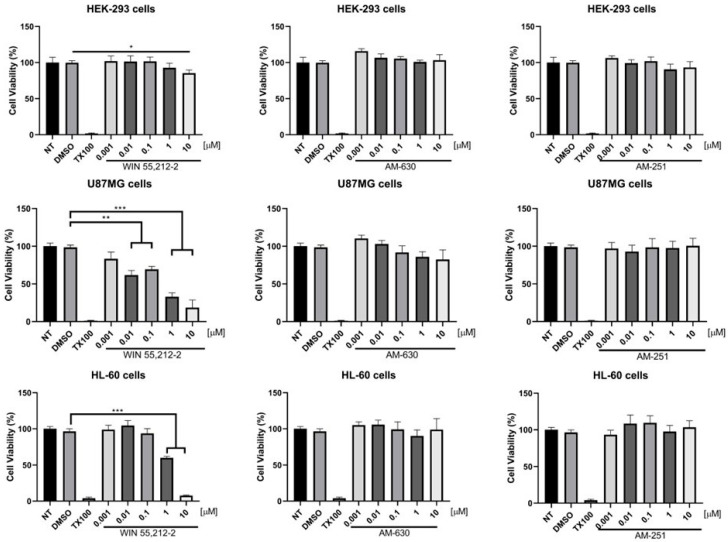
Cell viability [MTT-(3-(4,5-dimethylthiazol-2-yl)-2,5-diphenyltetrazolium bromide)] assays of the known synthetic cannabinoids WIN-55212-2 (CB_1_/CB_2_ agonist), AM251 (CB_1_ inverse agonist/antagonist), and AM630 (CB_2_ inverse agonist/antagonist) against the HEK293 cell line, U87MG cell line and HL-60 cell line. NT = untreated cell control; DMSO = solvent vehicle control; TX100 = known cell death control agent, Triton X-100. Significant differences in comparison with the vehicle (DMSO) are indicated *** *p* ≤ 0.001, ** *p* ≤ 0.01, * *p* ≤ 0.05.

Given the above, the CB_1_/CB_2_ agonist WIN-55212-2, the CB_1_ inverse agonist/antagonist AM251, and the CB_2_ inverse agonist/antagonist AM630 were evaluated for their cytotoxicity in selected in vitro models. These include human embryonic kidney 293 (HEK293) cells, considered a healthy cell line with low expression of both cannabinoid receptors [32]; neoplastic glioblastoma cells (U87MG), which highly express the CB_1_ receptor [33]; and acute promyelocytic leukemia cells (HL-60), which exclusively express CB_2_ receptors [6]. Results from these assays are shown in Figure 4.

Data demonstrate that both inverse agonist/antagonist AM251 and AM630 are non-toxic in the three selected cell lines. However, WIN-55212-2 exhibits cytotoxicity exclusively toward U87MG cells and the HL-60 cell line, which express the CB_1_ receptor and CB_2_ receptor, respectively (Figure 4). It has been reported that WIN-55212-2 shows K_i_ values of 1.89–123 nM for the CB_1_ receptor and 0.280–16.2 nM for the CB_2_ receptor [46,47]. Therefore, the observed cytotoxicity could be attributed to a mechanism involving cannabinoid receptor activation, considering that WIN-55212-2 is a well-established agonist and has been previously associated with the induction of cell death.

**Figure 5:**
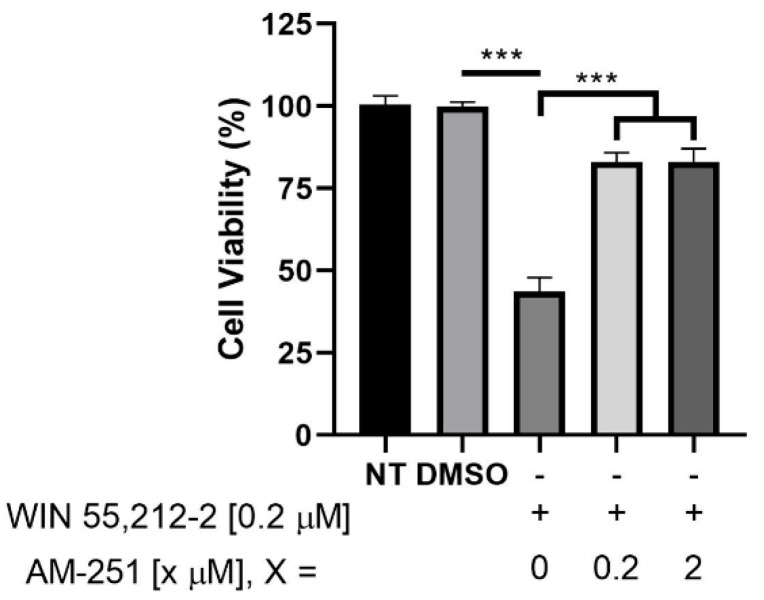
Cell viability experiments over glioblastoma cells (U87MG) between WIN-55212-2, at a predetermined concentration of 0.2 μM, and increasing concentrations of the CB_1_ inverse agonist/antagonist AM251. The toxic effect of WIN-55212-2 could be due to its cannabinoid agonist effect, since the toxicity ceases when the AM251 antagonist is used. NT = untreated cell control; DMSO = solvent vehicle control. Statistically significant differences are indicated *** *p*  <  0.001.

To provide further evidence for the aforementioned idea, WIN-55212-2 was assayed against increasing concentrations of the CB_1_ inverse agonist/antagonist AM251 at a concentration of 0.2 μM, which corresponds to the concentration of the IC_50_ that we obtained for WIN-55212-2 in U87MG cell cultures. U87MG cells, serving as a CB_1_ receptor model [33], were selected for analysis since the synthesized compounds predominantly bind to the CB_1_ receptor. Results showed that, as AM251 concentration increases, U87MG viability also increases, making WIN-55212-2 less toxic to these cells. These observations raise the possibility that the agonist and antagonist could be competing for the same active site and that the cytotoxic effect observed for the aminoalkylindole WIN-55212-2 might be related to its agonistic activity at the CB_1_ receptor. Figure 5 shows the obtained results for the performed assay between WIN-55212-2 and AM251.

**Figure 6:**
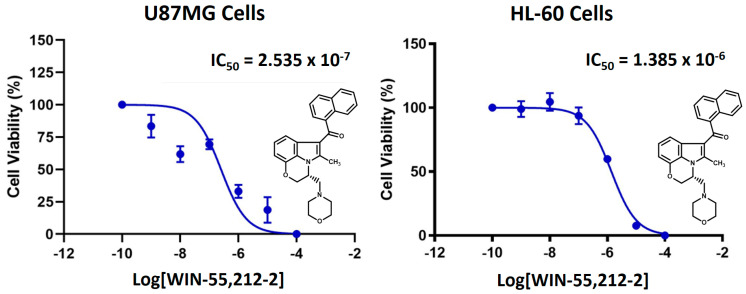
Pharmacological toxicity half maximal inhibitory concentration (IC_50_) for U87MG and HL-60 cell lines. The values correspond to 0.25 μM and 1.39 μM, respectively.

IC_50_ toxicity values of WIN-55212-2 in the U87MG cells and the HL-60 cell line were also determined. This was performed to obtain the specific parameters for our experimental cell line system. IC_50_ toxicity value for U87MG cells was 0.25 μM and for HL-60 was 1.39 μM, respectively (Figure 6).

Considering the results obtained from the assays performed above, which suggest that agonism could exert toxicity through cannabinoid receptor activation, synthesized benzo[*d*]imidazole–naphthalen-arylmethanone regioisomers that displaced more than 50% of the radioligand [^3^H]CP-55940 at the CB_1_ receptor were subjected to in vitro viability experiments at a concentration of 0.1 μM over HEK293 cells, U87MG cells, and HL-60 cells, in order to evaluate their cytotoxic effects according to the possibility of a cannabinoid-mediated agonist-induced cell death mechanism. HEK293 cells were used as a healthy cellular control that either expresses in negligible amounts the type 1 and type 2 cannabinoid receptors [32]. Table 2 summarizes the toxicity percentages of the best derivatives, as well as the IC_50_ values obtained from the experiments for the aminoalkylindole WIN-55212-2 and CB_1_/CB_2_ inverse agonists/antagonists.

Derivatives **4a′**, **4e′,** and **5b′** showed a reasonable radioligand displacement over the CB_2_ receptor; also, the **4f** derivative displaced [^3^H]CP-55940 from the CB_1_ receptor in more than 50%, and compound **5d** was able to displace the radioagent in both cannabinoid receptors (Table 1). Although the previous compounds showed reasonable results in binding assays, none of them exhibited a measurable IC_50_ value. This agrees with toxicity assays in Table 2, where none of these derivatives promoted toxicity in the three selected cell lines. Thus, the aforementioned compounds are unlikely to behave as cannabinoid agonists.

Compound **4f′** showed a displacement percentage of 53.25% for the CB_2_ receptor, in contrast to its regioisomer **4f**, which binds predominantly to the CB_1_ receptor. Derivative **4f′** exhibited 10% toxicity in the HL-60 cell line and 50% toxicity in healthy HEK293 cells, making it a less promising compound. This could be attributed to a potential but uncertain agonist activity in cells expressing the CB_2_ receptor, along with its greater harmful effect on healthy cells.

Regioisomer **4b′,** which has an IC_50_ = 6.76 μM and a K_i_ = 4.46 μM at the CB_2_ receptor, showed a 52% toxicity in HL-60. Nevertheless, this is neither indicative nor suggestive that this derivative is exerting its toxicity through an agonist mechanism on the cannabinoid type 2 receptor. This is because its toxicity is nonspecific, also showing that it is capable of interfering with cell viability in U87MG and HEK293 cell lines. Therefore, **4b′** would exert toxicity through a nonspecific mechanism for all sorts of cells, regardless of their cannabinoid receptor expression pattern. In the same manner, compounds **5b** and **5e** demonstrated IC_50_ values in a micromolar range (μM) and inhibition constants (K_i_’s) according to the Cheng–Prusoff equation of 1.53 μM and 1.82 μM, respectively, over the CB_1_ receptor. However, these synthesized compounds were also non-selective in their toxic activity (see Table 2) and therefore cannot be considered to interfere with cell viability through a presumed CB_1_-mediated agonist-induced mechanism.

On the other hand, compound **4d** showed the ability to bind to the CB_1_ receptor, exhibiting a K_i_ = 1.86 μM. This compound demonstrated selective toxicity toward U87MG cells over HL-60 cells, decreasing cell viability by 50%. This suggests that **4d** could be considered as a CB_1_ receptor agonist. However, it also exhibited slight toxicity toward healthy HEK293 cells, which somewhat limits its utility. Nonetheless, it is worth noting that its toxicity in the HEK293 control cell line, characterized by low expression of both cannabinoid receptors and non-neoplastic nature, is relatively low, with a value of 10% (see Table 2).

Finally, in the case of compound **5f**, the 5-fluoro regioisomer of **5f′**, it possesses an IC_50_ of 6.03 μM and a K_i_ of 1.48 μM over the CB_1_ receptor. This result is remarkable since this compound did not reduce the viability of any of the tested cell lines, and consequently, it is not behaving as an agonist according to our indirect proposed agonist-toxic cannabinoid mechanism. It is possible that **5f**, based on its displacement result shown in Table 1, could be an antagonist of the CB_1_ receptor. However, it would be necessary to perform a competition assay between WIN-55212-2 and **5f** in the U87MG cell culture, like the previous experiment with AM251 shown in Figure 5, in order to clarify this proposal. On the other hand, the 6-fluoro regioisomer **5f′**, with an IC_50_ of 6.03 μM and a K_i_ of 1.48 μM, behaves selectively as an agonist under our indirect determination standards for cannabinoid agonism mediated by toxicity. This is in contrast to its analog **5f**, which, as mentioned earlier, could be a CB_1_ antagonist. Specifically, **5f′** does not show any toxicity in HEK293 or HL-60 cells (see Table 2), but it does reduce viability in the U87MG cell line by 66%. From this, cannabinoid agonism by **5f′** can be inferred and could be considered as a lead compound of the synthesized series.

**Figure 7:**
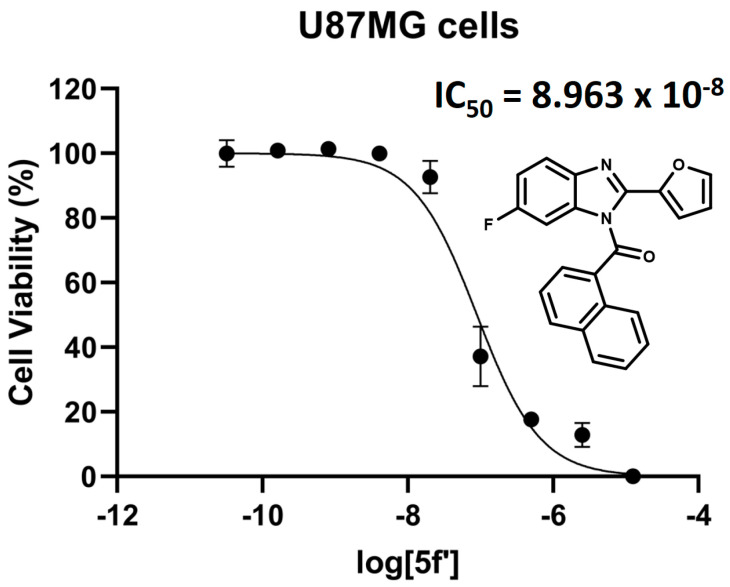
Dose–response curve and pharmacological toxicity half-maximal inhibitory concentration (IC_50_) of regioisomer **5f′** in U87MG cells. The value corresponds to 0.089 μM.

Given the above, the potency of compound **5f′** in U87MG cells was assessed using increasing concentrations of this regioisomer, due to its favorable toxicity profile and potential agonist activity under our indirect methodology. Figure 7 shows the dose–response curve of **5f′**, with a pharmacological toxicity IC_50_ of 0.089 μM. Comparison with WIN-55212-2 (IC_50_ of 0.25 μM) indicates that **5f′** is more potent and, consequently, more toxic to U87MG cells. This result is consistent with the toxicity of **5f′** shown in Table 2 at 0.1 μM, where it reduces U87MG cell viability by 66%. Similarly, the WIN-55212-2 dose–response curve (Figure 5) and bar graph (Figure 4) indicate that at the same concentration, WIN-55212-2 exhibits approximately 30% toxicity.

### 2.4. Docking Simulations

As mentioned in Section 2.1, we proposed a rational design to achieve cannabinoid activity. Specifically, to develop CB_1_ ligands, we employed an isosteric replacement of the indole ring for benzo[*d*]imidazoles containing two different halogen atoms in order to perform different sorts of interactions. Position 2- of the benzo[*d*]imidazoles was functionalized with various heterocycles, facilitating hydrogen bond interactions, π-π interactions, T-shaped bonding, and so on. The naphthalene core was retained owing to its ability to perform π-π interactions and/or π-cation interactions, among others.

For docking assays, compounds **4d**, **4f**, **5b**, **5d**, **5e**, **5f**, and **5f′** were selected due to their favorable cytotoxic data, along with WIN-55212-2 as a control for simulations. The crystal structure of the human CB1 receptor (PDB ID: 7WV9 [48]) was used. Calculations were performed in the orthosteric binding site. The selection of docking poses was guided by the best Glide XP docking scores for each compound, and the docking binding energies expressed in kcal/mol of each selected compound are shown in Table 3.

**Figure 8:**
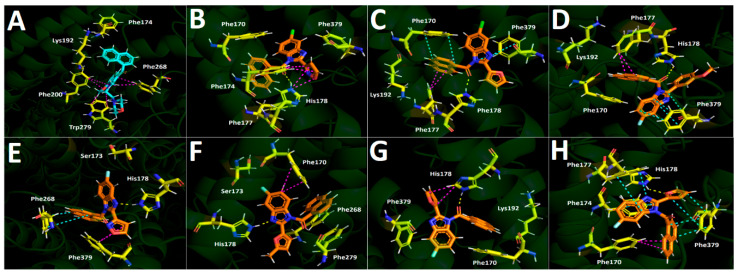
Predicted binding mode and predicted intermolecular interactions among the amino acids of the CB_1_ active site receptor and selected synthesized derivatives. Yellow dotted lines indicate hydrogen bond interactions, cyan dotted lines represent π-π interactions, and magenta dotted lines represent T-shaped interactions. (**A**) Aminoalkylindole WIN-55212-2. (**B**) Compound **4d**. (**C**) Compound **4f**. (**D**) Compound **5b**. (**E**) Compound **5d**. (**F**) Compound **5e**. (**G**) Compound **5f**. (**H**) Compound **5f′**.

WIN-55212-2 exhibited the most favorable scoring at −12.808 kcal/mol. Likewise, the known AAI and the synthesized compounds are arranged within the active site of the CB_1_ receptor in different manners. All benzo[*d*]imidazoles occupy the same region of the orthosteric pocket, at the site where CP-55940 binds in the 7WV9 protein, with most of them overlapping. Benzo[*d*]imidazoles **4d**, **4f, 5b,** and **5f** are arranged in the same manner, forming a group of compounds with a similar pose. Likewise, compounds **5d**, **5e,** and **5f′** form another molecular cluster, with their structures aligning similarly within the CB_1_ receptor’s active site. Figure 8A shows the aminoalkylindole, WIN-55212-2 (in cyan), positioned within the orthosteric pocket of the CB_1_ receptor and its main interactions within the active site.

The chlorinated derivatives **4d** and **4f**, which demonstrated displacement of more than 50% of the radioligand [^3^H]CP-55940, show the following docking descriptors. Derivative **4d** performs two T-shaped interactions through its isoxazole moiety with Phe174 and Phe177, as well as a hydrogen bond interaction with His178 through the oxygen atom of the carbonyl group (Figure 8B). Conversely, **4f** is surrounded by hydrophobic amino acids, engaging in a π-π interaction and a T-shaped interaction between its naphthalene core and the amino acids of Phe170 and Phe177, respectively. Additionally, **4f** performs a π-π interaction through its benzo[*d*]imidazole moiety and Phe379 and, like derivative **4d**, also performs a hydrogen bond interaction between its carbonyl group and His178 (Figure 8C).

On the other hand, fluorinated compounds **5b**, **5d**, **5e**, **5f,** and **5f′** primarily engage π-π, T-shaped, and hydrogen bond interactions. In the case of compound **5b** (Figure 8D), due to its similar alignment with compounds **4d** and **4f,** shows a T-shaped interaction between Phe177 and the naphthalene framework, a π-π interaction with Phe379 through its benzo[*d*]imidazole ring, and hydrogen bonding with His178 through the amide carbonyl group. Compound **5d** performs a π-π interaction with Phe268 and the naphthalene framework, in accordance with the planned design described in Section 2.1. Furthermore, it also forms a T-shaped interaction with Phe379 through its isoxazole nucleus at position 2- of the benzo[*d*]imidazole ring. Additionally, this derivative forms a hydrogen bond with His178, also through its nitrogen atom at position 3- of the benzo[*d*]imidazole core (Figure 8E). In the same manner, compound **5e**, which aligns similarly to derivative **5d** but with a slight shift within the active site, also carries out a hydrogen bond interaction between the nitrogen atom at position 3- of the benzo[*d*]imidazole core and His178. However, this derivative forms an additional T-shaped interaction with Phe170, despite being surrounded by Phe268 and Phe379 (Figure 8F).

As mentioned above, although the 5-fluorinated regioisomer **5f** possesses certain affinity for the CB_1_ receptor, this derivative does not display toxicity over U87MG cells or even the other cell lines. Hence, the hydrogen bond interaction with His178 through the carbonyl group, similar to derivatives **4d**, **4f**, and **5b**, as well as a T-shaped interaction between the furan framework and the same residue of His178 seen in Figure 8G, should only contribute to the CB_1_ receptor affinity, but not to the supposed, probable, and possible agonist activity. Instead, the lead compound **5f′**, which exhibited the best biological profile by being selectively toxic to U87MG cells, shows four π-π interactions. These include two π-π interactions between the Phe379 residue and the frameworks of naphthalene and furan, another π-π interaction between Phe177 and the benzo[*d*]imidazole core, and a final π-π interaction involving Phe174 and the bifused benzo[*d*]imidazole heterocycle. Moreover, as seen in Figure 8H, a T-shaped interaction occurs between Phe170 and the naphthalene framework, along with a hydrogen bond interaction between the oxygen atom of the furan ring and His178. The differences in the binding modes of **5f** and **5f′** may shed light on the differences in toxicity observed between the compounds. Although compound **5f** displayed slightly higher affinity for the CB_1_ receptor than compound **5f′** (Table 1), only **5f′** exhibited cytotoxicity in U87MG cells. As mentioned, this apparent discrepancy can be rationalized by considering their distinct binding interactions with the His178 residue. In compound **5f**, the carbonyl oxygen forms a hydrogen bond with His178; however, this interaction is weakened by the inductive effect of the fluorine atom at the 5- position, which decreases the electron density on the oxygen atom. In contrast, in compound **5f′,** the interaction with His178 occurs through the oxygen atom of the furan ring, which is positioned farther from the fluorine atom. This spatial arrangement reduces the impact of the fluorine substituent, thereby allowing a stronger hydrogen bond with His178. The enhanced stability of this interaction may contribute to the higher cytotoxicity of **5f′** in U87MG cells, despite its slightly lower overall affinity for CB_1_. Therefore, the binding affinity of **5f′** is probably influenced by its favorable interaction profile and the hydrogen bonding, which contributes to a strong affinity for this regioisomer.

On the other hand, the difference in affinity observed when the ring connected at position 2- is an isoxazole rather than a furan can be rationalized in thermodynamic terms. In general, the CB_1_ receptor cavity demands high lipophilicity from its ligands. The isoxazole ring exhibits a greater hydrogen-bond accepting capacity than furan, possesses a higher dipole moment (3.5 D versus 0.7 D, respectively), and is overall a less aromatic and more electrophilic system compared to furan. Taken together, these features render isoxazole a more easily solvated fragment, which entropically disfavors the binding mode of compounds bearing this substituent within the lipophilic cavity of the CB_1_ receptors.

Based on the reviewed docking and experimental results, it is not possible to determine with certainty whether an interaction with the Lys192 residue in the CB_1_ receptor’s active site is indeed important. None of the synthesized derivatives interact with this amino acid, yet they still exhibit affinity and potentially even a cannabinoid agonist effect, as suggested by our proposed experimental methodology. Thus, considering our findings along with previously reported data, Lys192 could really play an important role.

### 2.5. Integrated SAR Analysis

Our benzo[*d*]imidazole–naphthalen-arylmethanone series was conceived to probe CB_1_/CB_2_ recognition with a constant naphthalene amide fragment and systematic variation at two structural axes: (i) the identity and regiochemistry of a halogen on the benzo[*d*]imidazole core (5- vs. 6-position), and (ii) the heteroaryl/aryl substituent at position -2 of the benzo[*d*]imidazole scaffold (hydrogen-bond acceptors capable of engaging the orthosteric pocket). The SAR emerging from radioligand displacement (Table 1), the indirect functional readout via cell viability (Table 2; Figure 4, Figure 5 and Figure 6), and docking into CB_1_ (Figure 8) is coherent and mechanistically interpretable.

#### 2.5.1. Halogen Identity and Position on the Benzo[*d*]imidazole Core

A clear trend is observed across the set: a fluorine atom enhances CB_1_ recognition, while chlorine generally depresses CB_1_ and modestly biases CB_2_. Thus, fluorinated analogs **5b**, **5e**, **5f,** and **5f′** achieve the highest CB_1_ propensities (K_i_ ≈ 1.5–2.1 μM), whereas chlorine often shifts preference toward CB_2_ (e.g., **4b′**, CB_2_ K_i_ ≈ 4.46 μM) with only modest potency. Regiochemistry is equally consequential: a halogen at position 5- favors CB_1_, whereas at position 6- tends to erode CB_1_ and/or bias CB_2_. The matched pairs **4f**/**4f′** and **5a**/**5a′** exemplify this effect: derivative **4f** (5-Cl) displaces CB_1_ > 50%, while its 6-chloro regioisomer **4f′** preferentially displaces CB_2_; similarly, **5a** (5-F) shows appreciable CB_1_ displacement that is largely lost upon migration to 6-F (**5a′**). These data indicate that the electronic and topological consequences of placing a σ-withdrawing halogen at position 5 of the benzo[*d*]imidazole ring optimally align the π-systems for CB_1_ engagement, whereas substitution at position 6- perturbs this alignment and diminishes CB_1_ fitness.

#### 2.5.2. Substitution at Position 2- of Benzo[*d*]imidazole: Furan Is Favored for CB_1_ Engagement

Within the C-2 substituent series, five-membered oxygen heterocycles are privileged. Furan (**5f**/**5f′**) and 5-methylfuran (**5e**) consistently support CB_1_ affinity (K_i_ ~1.48–2.12 μM). Isoxazole is tolerated but less consistently productive; nevertheless, the chloro-isoxazole derivative **4d** stands out as a CB_1_-binding exception (K_i_ ≈ 1.86 μM). The 3-methoxyphenyl fragment (regioisomer **5b**) can deliver CB_1_ affinity (K_i_ ≈ 1.53 μM) but, as discussed below, is less favorable at the level of functional selectivity.

#### 2.5.3. Binding vs. Functional Outcome (Indirect Agonism Readout)

Because CB_1_/CB*2* agonism is known to reduce viability in receptor-expressing tumor lines, as mentioned, we used selective cytotoxicity as a pragmatic, indirect functional indicator (Figure 4, Figure 5 and Figure 6). The control agonist WIN-55212-2 killed U87MG cells and HL-60 cells while sparing the HEK293 cell line, and its toxicity in U87MG cell culture was antagonized by AM251, supporting the interpretation that “agonism → cytotoxicity” underlies our assay design. The following three patterns are proposed:

1. Affinity without CB_1_-mediated functional selectivity.

Compound **5b** (3-methoxyphenyl at position 2-) and **5e** (5-methylfuran at position 2-) possess respectable CB_1_/K_i_ values but are non-selectively cytotoxic (HEK293/U87MG/HL-60). This implies off-target or receptor-independent liabilities at 0.1 μM and argues that binding alone is not predictive of productive CB_1_ agonism in this chemotype.

2. Putative CB_1_ antagonist vs. CB_1_ agonist within a matched pair (regioisomers **5f** vs. **5f′**).

The 5-fluoro-2-furan regioisomer **5f** binds CB_1_ (K_i_ ≈ 1.48 μM) yet is non-toxic across all three cell lines at 0.1 μM, behavior consistent with a neutral/antagonist profile in our indirect readout. In contrast, its 6-fluoro-2-furan regioisomer **5f′** (K_i_ ≈ 2.12 μM) is selectively cytotoxic to U87MG (≈66%) while sparing HEK293 and HL-60 cells, a pattern congruent with CB_1_-mediated agonism in U87MG. Notably, therefore, regiochemistry can switch functional mode (antagonist-like → agonist-like) without a dramatic change in K_i_.

3. Chloro exception with CB_1_ agonist-like behavior (compound **4d**).

Despite the commonly observed effect of chlorine promoting CB_2_ binding tendency, **4d** (5-Cl, isoxazole) binds CB_1_ (K_i_ ≈ 1.86 μM) and shows preferential U87MG toxicity (≈50%) with low HEK293 toxicity (≈10%), indicating that CB_1_ agonism is still attainable in a chloro background when the pose and contact network are favorable.

#### 2.5.4. Binding-Mode Rationale

Docking into the CB_1_ orthosteric site (Figure 8) rationalizes the above. A recurring interaction across binders is hydrogen bonding to His178, delivered either by the amide carbonyl oxygen (e.g., compounds **5f**, **4d**, **5b**) or, in select cases, by a heteroatom in the ring at position 2- of the benzo[*d*]imidazole scaffold. What differentiates functional agonist-like compounds is the breadth and geometry of π-contacts in the proximal aromatic cage (Phe379, Phe177, Phe174, Phe170):

- Regioisomer **5f** (antagonist-like): engages His178 via the carbonyl oxygen and displays a modest T-shaped contact pattern around Phe170/Phe177. This appears sufficient for affinity but insufficient for productive receptor activation within our model.
- Regioisomer **5f′** (agonist-like): shifts the His178 hydrogen bond donor–acceptor pair to the furan oxygen, which is topologically farther from the fluorine than in regioisomer **5f**, mitigating inductive withdrawal from the hydrogen bond acceptor. Concomitantly, **5f′** builds a richer π-stacking network (dual π–π interaction to Phe379, plus π–π interactions to Phe177 and Phe174, and a T-shaped to Phe170). We hypothesize that this expanded aromatic engagement stabilizes an active-compatible pose, aligning with selective U87MG cytotoxicity.
- Compound **4d** (agonist-like despite chlorine): combines isoxazole-mediated edge-to-face contacts (Phe174/Phe177) with a carbonyl–His178 hydrogen bond, providing an alternative route to an activation-competent geometry even in a chloro series.

Importantly, Lys192—historically implicated in CB_1_ ligand recognition—is not a primary participant for this chemotype under our docking conditions, which helps explain why position 2-hydrogen acceptors (C-2 H-bond acceptors) alone are not sufficient to guarantee agonism unless embedded in the correct π-contact topology.

**Figure 9:**
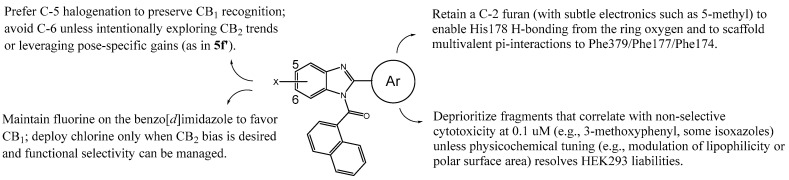
Summary of the proposed SAR from the present study. This information may serve as a guide for the design of new cannabinoid ligands. X = halogen.

Figure 9 presents a summary of the key structure–activity relationships proposed in our work. These insights are useful for guiding the design of future derivatives.

Based on the collective experimental findings reported in this study, we acknowledge that the methodology used to assess CB_1_ agonist activity, relying on cell viability assays, provides an indirect measure of receptor activation. While the observed cytotoxicity is consistent with well-established evidence that CB_1_ and CB_2_ receptor activation by agonists can induce cell death in tumor cells, and considering as well the selective cytotoxic behavior of conventional cannabinoid ligands and our synthesized compounds toward U87MG and HL-60 cells but not toward HEK293 cells, alternative mechanisms independent of CB_1_ activation—such as induction of oxidative stress, direct effects on cell proliferation pathways, or disruption of the cell membrane—cannot be completely ruled out. Therefore, the results should be interpreted as indicative of potential CB_1_ agonist activity rather than definitive proof of receptor-mediated effects. Despite this limitation, the combination of computational predictions and biological outcomes provides valuable guidance for the identification of promising compounds for further investigation.

## 3. Materials and Methods

### 3.1. Binding Assays CB_1_/CB_2_ (Radioligand Displacement)

For CB_1_ and CB_2_ receptor binding assays, the compounds were subjected to a screening using three different concentrations (1 mM, 0.1 mM and 10 μM) of the synthesized compounds; HEK-293 cell membranes overexpressing either the human cannabinoid CB_1_ receptor –hCB1 HEK293-EBNA membranes– (Perkin Elmer Inc., Singapore Pte. Ltd. Product No.: RBHCB1M400UA), or the human recombinant cannabinoid CB_2_ receptor –hCB2 HEK293-EBNA Membranes—(Perkin Elmer Inc., Singapore Pte. Ltd. Product No.: RBXCB2M400UA); and [^3^H]-(-)cis-3-[2-hydroxy-4-(1,1-dimethylheptyl)phenyl]-*trans*-4-(3-hydroxypropyl)cyclohexanol ([^3^H]CP-55940; K_d_ = 500 pM) as the high affinity ligand. Compounds that displaced [^3^H]CP-55940 by more than 50% at 1 mM were further analyzed. Receptors were incubated with compounds at 30 °C for 1.5 h at different increasing concentrations using 0.5 nM of [^3^H]CP-55940. In all cases, K_i_ values were calculated applying the Cheng–Prusoff equation [45]. Displacement curves were generated using GraphPad, GraphPad Software, Inc., La Jolla, CA, USA, version 8.0.

### 3.2. Cell Cultures

Human embryonic kidney 293 (HEK293), neoplastic glioblastoma cells (U87MG), and acute promyelocytic leukemia cells (HL-60) were maintained at 5% CO_2_ and 37 °C in DMEM, EMEM, and RPMI medium, respectively, containing 10% Fetal Bovine Serum and 100 U/mL penicillin and 100 mg/mL streptomycin.

### 3.3. Cell Viability: ([MTT-(3-(4,5-dimethylthiazol-2-yl)-2,5-diphenyltetrazolium bromide)-formazan])

HEK293, U87MG, and HL-60 cells were first quantified to determine the exact number to be used in the MTT cell viability assays, ensuring that absorbance values remained below 1 unit in accordance with Beer–Lambert [49].

HEK293, U87MG, and HL-60 cell lines were seeded in a flat-bottom 96-well plate at a density of 10,000 cells per well. Cells were then incubated with WIN-55212-2, AM251, and AM630 at various concentrations (0.001 μM to 10 μM) in 200 μL of respective culture medium supplemented with 10% fetal bovine serum (FBS), at 37 °C for 72 h. Subsequently, 20 μL of 5 mg/mL MTT solution was added to each well, and cells were incubated at 37 °C for 4 h. The resulting formazan crystals were then solubilized with 10% sodium dodecyl sulfate (SDS) in 0.1 mM HCl, followed by overnight incubation at 37 °C. Untreated cells were used as the control, 0.1% of dimethyl sulfoxide (DMSO) as the vehicle control, and 0.2% of Triton X-100 as a positive control for cell death. IC_50_ toxicity values of WIN-55212-2 in U87MG cells and HL-60 cell line were determined.

Competition assay between WIN-55212-2 and AM251 in U87MG cells was conducted under the same conditions described in the previous paragraph. Therefore, WIN-55212-2 was assayed in the presence of increasing concentrations of the CB_1_ inverse agonist/antagonist AM251 (0 μM to 2.0 μM), while WIN-55212-2 was kept at a fixed concentration of 0.2 μM, based on the previously determined IC_50_. The stimulation with both CB ligands was performed simultaneously.

Cell viability assays of HEK293, U87MG and HL-60 cell lines using compounds that displaced more than 50% of the radioligand [^3^H]CP-55940 at the CB_1_ receptor (regioisomers **4a′**, **4b′**, **4d**, **4e′**, **4f**, **4f′**, **5b**, **5b′**, **5d**, **5e**, **5f** and **5f′**) were performed (Table 2). All compounds were incubated at 0.1 μM in 200 μL of culture medium supplemented with 10% FBS, in a flat-bottom 96-well plate at a density of 10,000 cells per well, at 37 °C for 72 h. Next, 20 μL of a 5 mg/mL MTT solution was added, and the cells were incubated at 37 °C for 4 h. Then, they were solubilized with 10% sodium dodecyl sulfate (SDS) in 0.1 mM HCl and incubated overnight at 37 °C. Formazan absorbance of each well was measured using the EPOCH microplate reader (Biotek, Winooski, VT, USA) at a wavelength of 570 nm.

### 3.4. Molecular Docking Experiments

Docking simulations were performed for regioisomers **4d**, **4f**, **5b**, **5d**, **5e**, **5f**, **5f′**, and WIN-55212-2. Energetic minimization of each molecule were carried out using the LigPrep tool in the program Maestro Schrodinger suite v.11.8 (Schrödinger, LLC, New York, NY, USA) [50]. Cannabinoid receptor type 1 structure obtained from cryo-electron microscopy [CB_1_ receptor PDBID: 7WV9] [48], were obtained from the Protein Data Bank RCSB-PDB [51]. Receptor optimization was performed using the Protein Preparation Wizard available in Maestro software. Water molecules (if applicable) were removed from the protein active site (orthosteric site). Appropriate ionization states for acid and basic amino acid residues, as well as polar hydrogen atoms, were considered at physiological pH = 7.4. The enclosing box was configured as a cube with 26 Å length, and the OPLS3e force field was employed for protein energy minimization. The centroid of the selected residue was determined based on the putative orthosteric active site of the CB_1_ receptor and its known catalytic amino acids, where the orthosteric ligand CP-55940 is positioned. The Glide Induced Fit Docking protocol has been used for the final couplings [52]. Compounds were punctuated by the Glide scoring function in the extra-precision mode (Glide XP; Schrödinger, LLC) [53,54] and were filtered on the basis of the best scores and best RMS values (less than 1 unit as a cutting criterion), in order to obtain the potential intermolecular interactions between compounds and the receptor, as well as the binding mode and docking descriptors.

## 4. Conclusions

We developed a series of benzo[*d*]imidazole-based novel cannabinoid ligands through an isosteric replacement of the indole ring of the aminoalkylindole cannabinoids. Cell viability experiments (toxicity) over three different cell lines with and without expression of cannabinoid receptors were used to determine a likely agonist profile of compounds. Some of the synthesized derivatives likely exhibited the ability to activate the CB_1_ receptor, as suggested by our experimental model, considering that it has been well established that CB_1_ and CB_2_ receptor activation by agonists induces cell death and inhibits tumor cell growth. However, to confirm this definitively, specific assays are required, such as a cAMP control, among others. Nevertheless, our proposal could be innovative and plausible as a suggestion. In this sense, WIN-55212-2 was subjected to viability assays over HEK293, U87MG, and HL-60 cells, showing toxicity on the last cell lines (0.25 μM for U87MG and 1.39 μM for HL-60) and harmlessness for the HEK293 cells. Subsequently, a competition assay was performed between the cannabinoid agonist WIN-55212-2 and the inverse agonist/antagonist AM251, where it was found that increasing the concentration of AM251 reduced the toxicity of WIN-55212-2, thereby supporting the indirect agonism method we have proposed. Thus, compounds that demonstrated affinity for cannabinoid receptors and also were cytotoxic in cell lines expressing the cannabinoid receptor to which the compound is bound could indeed be cannabinoid agonists. Specifically, five compounds (**4d**, **5b**, **5e**, **5f,** and **5f′**) showed affinity for the CB_1_ receptor, allowing the determination of half maximal inhibitory concentrations (IC_50_) and affinity constants (K_i_). It is noteworthy that the K_i_ of the compounds in this study ranges between 1 µM and 2 µM, which is a fold lower than the affinity reported for WIN 55212-2. However, despite the lower affinity, compounds such as **4d** and **5f′** behave as potential agonists in our proposed functional methodology. Indeed, derivative **5f′**, with an IC_50_ of 6.03 μM and a K_i_ of 1.48 μM, behaves selectively as an agonist according to our indirect determination standards for cannabinoid agonism, as indicated by a 66% toxicity at 0.1 μM in the U87MG cell line. However, as was already mentioned, further experiments to confirm the agonistic nature must be conducted to obtain accurate and precise results.

## Figures and Tables

**Table 1:** Radioligand displacement results for benzimidazole 5-chloro(or fluoro)benzo[*d*]imidazole–naphthalen-methanone and 6-chloro(or fluoro)benzo[*d*]imidazole regioisomers-naphthalen-arylmethanone.

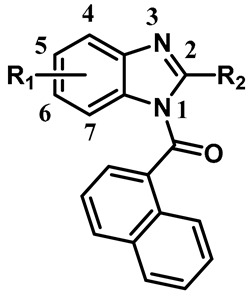								
				**CB_1_**			**CB_2_**	
**Compound**	**R_1_**	**R_2_**	**IC_50_ (µM)**	**K_i_ (µM)**	**Max Tested**	**IC_50_ (µM)**	**K_i_ (µM)**	**Max Tested**
					**µM** **(% Displacement)**			**µM** **(% Displacement)**
**4a**	5-Cl	**pyridin-3-yl**	>10	>10	10 μM (36%)	10	6.6	10 μM (35%)
**4a′**	6-Cl		>10	>10	10 μM (20%)	10	6.6	10 μM (52%)
**4b**	5-Cl	**3-methoxyphenyl**	>10	>10	10 μM (39%)	>10	>10	10 μM (48%)
**4b′**	6-Cl		>10	>10	10 μM (43%)	6.76 ± 3.09	4.46 ± 2.68	10 μM (58%)
**4c**	5-Cl	**5-methylisoxazole**	>10	>10	10 μM (38%)	>10	>10	10 μM (30%)
**4c′**	6-Cl		>10	>10	10 μM (44%)	>10	>10	10 μM (29%)
**4d**	5-Cl	**isoxazole**	7.58 ± 4.21	1.86 ± 2.27	10 μM (55%)	>10	>10	10 μM (15%)
**4d′**	6-Cl		>10	>10	10 μM (47%)	>10	>10	10 μM (42%)
**4e**	5-Cl	**5-methylfuran-2-yl**	>10	>10	10 μM (30%)	>10	>10	10 μM (29%)
**4e′**	6-Cl		>10	>10	10 μM (38%)	>10	>10	10 μM (52.8%)
**4f**	5-Cl	**furan-2-yl**	>10	>10	10 μM (56.5%)	>10	>10	10 μM (46.5%)
**4f′**	6-Cl		>10	>10	10 μM (47.5%)	>10	>10	10 μM (53.25%)
**5a**	5-F	**pyridin-3-yl**	>10	>10	10 μM (41%)	>10	>10	10 μM (43%)
**5a′**	6-F		>10	>10	10 μM (9.5%)	>10	>10	10 μM (16%)
**5b**	5-F	**3-methoxyphenyl**	6.23 ± 3.80	1.53 ± 1.91	10 μM (58.25%)	>10	>10	10 μM (45%)
**5b′**	6-F		>10	>10	10 μM (47%)	>10	>10	10 μM (53.5%)
**5c**	5-F	**5-methylisoxazole**	>10	>10	10 μM (54.5%)	>10	>10	10 μM (24%)
**5c′**	6-F		>10	>10	10 μM (37%)	>10	>10	10 μM (29%)
**5d**	5-F	**isoxazole**	>10	>10	10 μM (54%)	>10	>10	10 μM (51%)
**5d′**	6-F		>10	>10	10 μM (34%)	>10	>10	10 μM (26%)
**5e**	5-F	**5-methylfuran-2-yl**	7.40 ± 3.28	1.82 ± 1.64	10 μM (58.25%)	>10	>10	10 μM (38.5%)
**5e′**	6-F		>10	>10	10 μM (43%)	>10	>10	10 μM (52%)
**5f**	5-F	**furan-2-yl**	6.03 ± 4.09	1.48 ± 2.03	10 μM (65.5)	>10	>10	10 μM (54.5%)
**5f′**	6-F		8.65 ± 3.11	2.12 ± 1.55	10 μM (55%)	>10	>10	10 μM (47%)

Data are expressed as means ± SEM of *n* = 3 independent experiments. Affinity was calculated by using [^3^H]CP-55940 as radioligand on human CB_1_ and CB_2_ cannabinoid receptors. K_i_ and IC_50_ are expressed in μM.

**Table 2:** Percentage of toxicity of benzo[*d*]imidazole–naphthalen-arylmethanone regioisomers that displaced more than 50% the radioligand [^3^H]CP-55940 against U87MG cell line (neoplastic glioblastoma cells) and HL-60 cell line (promyelocytic leukemia cells) at 0.1 μM.

	% Toxicity		
Compound	HEK293T	U87MG	HL-60
**4a′**	NT	NT	NT
**4b′**	50 ± 11	39± 20	52 ± 13
**4d**	10 ± 9	50 ± 14	NT
**4e′**	NT	NT	NT
**4f**	NT	NT	NT
**4f′**	50 ± 23	NT	10 ± 19
**5b**	75 ± 17	25 ± 14	45 ± 17
**5b′**	NT	NT	NT
**5d**	NT	NT	NT
**5e**	55 ± 23	39 ± 18	50 ± 19
**5f**	NT	NT	NT
**5f′**	NT	66 ± 17	NT
	**IC_50_ (μM)**		
**WIN-55212-2**	>10	0.22	1.39
**AM630**	>10	>10	>10
**AM251**	>10	>10	>10

Three independent experiments (*n* = 3) were carried out. NT = Non-toxic at 0.1 μM.

**Table 3:** Binding energies obtained from docking experiments of selected compounds, as well as the known cannabinoid ligand WIN-55212-2, over cannabinoid type 1 receptor (CB_1_ receptor).

Compound	Binding Energy (kcal/mol)
**4d**	−10.227
**4f**	−10.608
**5b**	−10.061
**5d**	−9.767
**5e**	−11.096
**5f**	−10.152
**5f′**	−10.335
**WIN-55212-2**	−12.808

## Data Availability

The original contributions presented in this study are included in the article/Supplementary Materials. Further inquiries can be directed to the corresponding author(s).

## Supplementary Materials

The following supporting information can be downloaded at https://www.mdpi.com/article/10.3390/ijms26209986/s1.
