# Supplementary material for: Benzo[d]imidazole–Naphthalen-Arylmethanone Regioisomers as CB1 Ligands: Evaluation of Agonism via an Indirect Cytotoxicity-Based Approach
Source: Int J Mol Sci. 2025 Oct 14;26(20):9986. doi: 10.3390/ijms26209986 (PMC12564498; doi:10.3390/ijms26209986)
Supplement: Supplementary file 1 [file ijms-26-09986-s001.zip › ijms-3874477-supplementary.pdf]

Supporting Information

Benzo[*d*]imidazole–Naphthalen-Arylmethanone Regioisomers as CB_1 Ligands: Evaluation of Agonism via an Indirect Cytotoxicity-Based Approach

Analia Young Hwa Cho[a], Renato Burgos Ravanal[b], Valeria Zuñiga Salazar[a], Marco Mellado[c], Marcos Lorca[d], David Pessoa-Mahana[b], Jaime Mella-Raipán[e], Germán Günther Sapunar[a] and Javier Romero-Parra [a]*.

[a] *Organic Chemistry and Physical Chemistry Department, Faculty of Chemical and Pharmaceutical Sciences, Universidad de Chile, Olivos 1007, Santiago 7820436, Chile; javier.romero@ciq.uchile.cl*

[b] *School of Pharmacy, Faculty of Chemistry and Pharmacy, Pontificia Universidad Católica de Chile, Vicuña Mackenna 4860, Santiago 7820436, Chile.*

[c] *Centro de Investigación en Ingeniería de Materiales, Universidad Central de Chile, Santiago 8330507, Chile.*

[d] *Facultad de Ciencias de la Vida, Carrera de Química y Farmacia, Universidad Viña del Mar, Viña del Mar 2520000, Chile.*

[e] *Institute of Chemistry and Biochemistry, Faculty of Sciences, Universidad de Valparaíso, Valparaíso 2360102, Chile; Faculty of Pharmacy, Chilean Pharmacopoeia Research Center, Universidad de Valparaíso, Valparaíso 2360102, Chile*

Table of contents

General information…………………………………………………………………………………………..4.

Chemistry……4.

1) Procedure for the synthesis of 6-chloro(or–fluoro)-2-aryl-1H-benzo[d]imidazoles 3a-b/3a'-b' and 3e-f/3e'-f'……………………………………………………………………………………………4.

2) Procedure for the synthesis of 6-chloro(or–fluoro)-2-aryl-1*H*-benzo[*d*]imidazoles 3c-d/3c'-d'……………………………………………………………………………………………4.

- 5(6)-chloro-2-(pyridin-3-yl)-1*H*-benzo[*d*]imidazole (**3a**).
- 5(6)-chloro-2-(3-methoxyphenyl)-1*H*-benzo[*d*]imidazole (**3b**).
- 3-(5(6)-chloro-1*H*-benzo[*d*]imidazol-2-yl)-5-methylisoxazole (**3c**).
- 3-(5(6)-chloro-1*H*-benzo[*d*]imidazol-2-yl)isoxazole (**3d**).
- 5(6)-chloro-2-(5-methylfuran-2-yl)-1*H*-benzo[*d*]imidazole (**3e**).
- 5(6)-chloro-2-(furan-2-yl)-1*H*-benzo[*d*]imidazole (**3f**).
- 5(6)-fluoro-2-(pyridin-3-yl)-1*H*-benzo[*d*]imidazole (**3a'**).
- 5(6)-fluoro-2-(3-methoxyphenyl)-1*H*-benzo[*d*]imidazole (**3b'**).
- 3-(5(6)-fluoro-1*H*-benzo[*d*]imidazol-2-yl)-5-methylisoxazole (**3c'**).
- 3-(5(6)-fluoro-1*H*-benzo[*d*]imidazol-2-yl)isoxazole (**3d'**).
- 5(6)-fluoro-2-(5-methylfuran-2-yl)-1*H*-benzo[*d*]imidazole (**3e'**).
- 5(6)-fluoro-2-(furan-2-yl)-1*H*-benzo[*d*]imidazole (**3f'**).

3) Procedure for the synthesis of (5 or 6)-(chloro or fluoro)-2-(aryl)-1H-benzo[d]imidazol-1-yl)(naphtalen-1-yl)methanone (4a-f)/ (4a'-f') and (5a-f)/(5a'-f')………………………………………10.

- (5-chloro-2-(pyridin-3-yl)-1*H*-benzo[*d*]imidazol-1-yl)(naphthalen-1-yl)methanone (**4a**)
- (6-chloro-2-(pyridin-3-yl)-1H-benzo[d]imidazol-1-yl)(naphthalen-1-yl)methanone (**4a'**).
- (5-chloro-2-(3-methoxyphenyl)-1*H*-benzo[*d*]imidazol-1-yl)(naphthalen-1-yl)methanone (**4b**).
- (6-chloro-2-(3-methoxyphenyl)-1*H*-benzo[*d*]imidazol-1-yl)(naphthalen-1-yl)methanone (**4b'**).
- (5-chloro-2-(5-methylisoxazol-3-yl)-1*H*-benzo[*d*]imidazol-1-yl)(naphthalen-1-yl)methanone (**4c**).
- (6-chloro-2-(5-methylisoxazol-3-yl)-1*H*-benzo[*d*]imidazol-1-yl)(naphthalen-1-yl)methanone (**4c'**).
- (5-chloro-2-(isoxazol-3-yl)-1*H*-benzo[*d*]imidazol-1-yl)(naphthalen-1-yl)methanone (**4d**).
- (6-chloro-2-(isoxazol-3-yl)-1*H*-benzo[*d*]imidazol-1-yl)(naphthalen-1-yl)methanone (**4d'**).
- (5-chloro-2-(5-methylfuran-2-yl)-1*H*-benzo[*d*]imidazol-1-yl)(naphthalen-1-yl)methanone (**4e**).
- (6-chloro-2-(5-methylfuran-2-yl)-1*H*-benzo[*d*]imidazol-1-yl)(naphthalen-1-yl)methanone (**4e'**).
- (5-chloro-2-(furan-2-yl)-1*H*-benzo[*d*]imidazol-1-yl)(naphthalen-1-yl)methanone (**4f**).
- (6-chloro-2-(furan-2-yl)-1*H*-benzo[*d*]imidazol-1-yl)(naphthalen-1-yl)methanone (**4f'**).
- (5-fluoro-2-(pyridin-3-yl)-1*H*-benzo[*d*]imidazol-1-yl)(naphthalen-1-yl)methanone (**5a**).

- (6-fluoro-2-(pyridin-3-yl)-1*H*-benzo[*d*]imidazol-1-yl)(naphthalen-1-yl)methanone (**5a'**).
- (5-fluoro-2-(3-methoxyphenyl)-1*H*-benzo[*d*]imidazol-1-yl)(naphthalen-1-yl)methanone (**5b**).
- (6-fluoro-2-(3-methoxyphenyl)-1*H*-benzo[*d*]imidazol-1-yl)(naphthalen-1-yl)methanone (**5b'**).
- (5-fluoro-2-(5-methylisoxazol-3-yl)-1*H*-benzo[*d*]imidazol-1-yl)(naphthalen-1-yl)methanone (**5c**).
- (6-fluoro-2-(5-methylisoxazol-3-yl)-1*H*-benzo[*d*]imidazol-1-yl)(naphthalen-1-yl)methanone (**5c'**).
- (5-fluoro-2-(isoxazol-3-yl)-1*H*-benzo[*d*]imidazol-1-yl)(naphthalen-1-yl)methanone (**5d**).
- (6-fluoro-2-(isoxazol-3-yl)-1*H*-benzo[*d*]imidazol-1-yl)(naphthalen-1-yl)methanone (**5d'**).
- (5-fluoro-2-(5-methylfuran-2-yl)-1*H*-benzo[*d*]imidazol-1-yl)(naphthalen-1-yl)methanone (**5e**).
- (6-fluoro-2-(5-methylfuran-2-yl)-1*H*-benzo[*d*]imidazol-1-yl)(naphthalen-1-yl)methanone (**5e'**).
- (5-fluoro-2-(furan-2-yl)-1*H*-benzo[*d*]imidazol-1-yl)(naphthalen-1-yl)methanone (**5f**).
- (6-fluoro-2-(furan-2-yl)-1*H*-benzo[*d*]imidazol-1-yl)(naphthalen-1-yl)methanone (**5f'**).

NMR spectra of compounds……………………………………………………………………………………………….27.

FT-IR spectra of compounds……………………………………………………………………………………………….75.

Biological experiments…………………………………………………………………………………………………….87.

- Binding assays CB_1/CB_2 (radioligand displacement).
- Cell cultures.
- Cell viability: ([MTT - (3-(4,5-dimethylthiazol-2-yl)-2,5-diphenyltetrazolium bromide) - formazan].

Molecular docking experiments………………………………………………………………………………………89.

General information

All starting materials and solvents were purchased from commercial suppliers and used without further purification. Solvents were dried by reflux over sodium overnight and freshly distilled before use. Reactions over nitrogen atmosphere were carried out filling the reaction apparatuses through a gas flow of the corresponding, commercially available gas and afterwards closing the filled reaction system with a gas filled balloon. Thin layer chromatography (TLC) was performed on silica gel on aluminum foils (1.5 × 5 cm) pre-coated (0.25 mm) with silica gel (Merck) with fluorescent indicator at 254 nm. For column chromatography silica gel 60 (particle size: 0.063 - 0.200 mm or 0.035 - 0.070 mm) was used. Compounds were visualized by exposure to UV light. Melting points were determined on a Electrothermal 9100 apparatus and are uncorrected. Infrared spectrums were obtained on a FT-IR MIR-NIR Spectrofotometer Perkin-Elmer. Nuclear magnetic resonance spectra were recorded on a Bruker AM-300 MHz instrument using DMSO-d6 or $CDCl_3$ solutions containing tetramethylsilane as internal standard. Chemical shifts are expressed in parts per million (ppm) downfield from TMS, coupling constant values (*J*) are given in Hertz. Multiplicity are abbreviated as, s: singlet; brs: broad singlet d: doublet; t: triplet; q: quartet; p: quintet; m: multiplet; dd: doublet of doublet; and so on. The purity of compounds was determined by TLC and mass spectra (MS) of final compounds were recorded. Samples were analyzed in positive ionization modes in a Quadrupole Time-of-Flight (QTOF), Electrospray Ionization (ESI), with a flow rate of 2 µL/min of 90% acetonitrile. Monoisotopic mass values and their ions in positive mode (M+H) and negative mode (M-H) were obtained using Bruker's 'Isotope Pattern' software.

Chemistry:

1) Procedure for the synthesis of 6-chloro(or–fluoro)-2-aryl-1H-benzo[d]imidazoles 3a-b/3a'-b' and 3e-f/3e'-f'

To a solution of 4-chlorobenzene-1,2-diamine (**1**) (500 mg, 3.5 mmol) or 4-fluorobenzene-1,2-diamine (**1'**) (500 mg, 3.5 mmol) in *N*,*N*-dimethylformamide (DMF) (50 mL) in a reaction flask under aerobic conditions and magnetic stirring, magnesium chloride hexahydrate ($MgCl_2{\cdot}6H_2O$) (71.2mg, 10 mol%) was added. Subsequently, 3.5mmol of the corresponding aldehydes (**2a-b** or **2e-f**) were slowly added dropwise. After addition, the reaction was stirred at room temperature for 24 h after which the DMF was removed in a rotary evaporator in vacuo. The solid residue was purified by column chromatography on silica gel with a mixture of CH_2Cl_2:methanol (99:1) or ethyl acetate as eluents to yield compounds **3a-b** and **3e-f** when 4-chlorobenzene-1,2-diamine (**1**) was used or compounds **3a'-b'** and **3e'-f'** in case of 4-fluorobenzene-1,2-diamine (**1'**) was the starting reagent.

2) Procedure for the synthesis of 6-chloro(or–fluoro)-2-aryl-1*H*-benzo[*d*]imidazoles 3c-d/3c'-d'

To a solution of 4-chlorobenzene-1,2-diamine (**1**) (500 mg, 3.5 mmol) or 4-fluorobenzene-1,2-diamine (**1'**) (500 mg, 3.5 mmol) in 50 mL of polyphosphoric acid (PPA) in a reaction flask under aerobic conditions and magnetic stirring the corresponding carboxylic acids (**2c** and **2d**) (3.5 mmol) were also added. Then, the reaction was stirred at 180°C for 3 h and after this time is over the reaction was poured over a $NaHCO_3$ water solution until pH 7-8 was reached. The mixture was extracted with ethyl acetate (3 x 100 mL) and the organic portions were dried with anhydrous Na_2SO_4 and filtered. Finally, the solvent was removed in rotary evaporator in vacuo, yielding compounds **3c-d** and **3c'-d'** respectively without further purification.

<u>5(6)-chloro-2-(pyridin-3-yl)-1*H*-benzo[*d*]imidazole (3a):</u>

Following the general procedure 1.

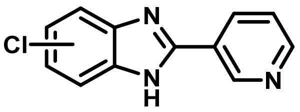

White solid. Yield = 30%. mp: 145 – 147 °C. (*)

^{1}H NMR (300 MHz, DMSO-d^{6}) δ ppm: 13.30(brs, 1H), 9.34 (d, 1H, J_1 =1.6 Hz), 8.69 (dd, 1H, J_1 =4.8 Hz, J_2 =1.6 Hz), 8.48 (dt, 1H, J_1 = 8.0 Hz, J_2 = 1.6 Hz), 7.68 (d, 1H, J_1 = 1.8 Hz), 7.63 (d, 1H, J_1 = 8.6 Hz), 7.60 (dd, 1H, J_1 = 7.8 Hz, J_2 = 4.8 Hz), 7.23 (dd, 1H, J_1 = 8.6 Hz, J_2 = 1.98 Hz).

^{13}C NMR (75.5 MHz, DMSO-d^{6}) δ ppm: 150.83, 150.36, 147.66, 140.61, 137.91, 133.98, 126.83, 125.81, 124.07, 122.78, 116.38, 115.17.

* Jung, M.H., et al., *Synthesis of 2-(1-methyl-1, 2, 5, 6-tetrahydropyridin-3-yl) benzimidazoles.* Journal of heterocyclic chemistry, 2003. **40**(1): p. 37-44.

<u>5(6)-chloro-2-(3-methoxyphenyl)-1*H*-benzo[*d*]imidazole (3b):</u>

Following the general procedure 1.

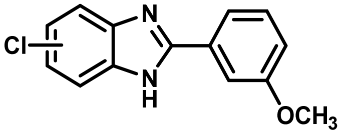

White solid. Yield = 61%. mp: 139 -140 °C. (*)

^{1}H NMR (300 MHz, DMSO-d^{6}) δ ppm: 7.79 (brs, 1H), 7.76 (d, 1H, J_1 = 1.6 Hz), 7.66 (d, 1H, J_1 = 1.6 Hz), 7.60 (d, 1H, J_1 = 8.3 Hz), 7.45 (t, 1H, J_1 = 8.3 Hz), 7.22 (dd, 1H, J_1 = 8.5 Hz, J_2 = 1,6 Hz), 7.06 (dt, 1H, J_1 = 8.5 Hz, J_2 = 1.6 Hz), 3.85 (s, 3H).

^{13}C NMR (75.5 MHz, DMSO-d^{6}) δ ppm: 159.66, 152.64, 140.68, 137.91, 131.11, 130.13, 126.43, 122.35, 118.92, 116.18, 116.05, 114.99, 111.54, 55.29.

(*) Siddiqui, H., et al., *Synthesis and in vitro α-chymotrypsin inhibitory activity of 6 chlorobenzimidazole derivatives.* Bioorganic & Medicinal Chemistry, 2016. **24**(16): p. 3387-3395.

3-(5(6)-chloro-1*H*-benzo[*d*]imidazol-2-yl)-5-methylisoxazole (3c):

Following the general procedure 2.

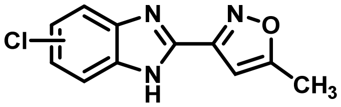

White solid. Yield = 33%. mp = 271.7 – 272-6 °C.

^{1}H NMR (300 MHz, DMSO-d^6) δ ppm: 7.66 (d, 1H, J_1 = 1.8 Hz), 7.62 (d, 1H, J_1 = 8.6 Hz), 7.26 (dd, 1H, J_1 = 8.6 Hz J_2 = 1.8 Hz), 6.82 (d, J_1 = 0.6 Hz 1H), 2,50 (s, 3H).

^{13}C NMR (75.5 MHz, DMSO-d^6) δ ppm: 171.16, 155.46, 143.62, 139.98, 137.46, 127.30, 123.18, 116.94, 115.55, 101.02, 11.85.

3-(5(6)-chloro-1*H*-benzo[*d*]imidazol-2-yl)isoxazole (3d):

Following the general procedure 2.

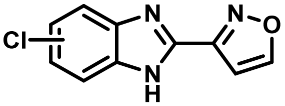

White solid. Yield = 66%. mp: 235.2 – 236.4 °C.

^{1}H NMR (300 MHz, DMSO-d^6) δ ppm: 9.17(d, 1H, J_1 = 1.7 Hz), 7.69 (d, 1H, J_1 = 2.0 Hz), 7.64 (d, 1H, J_1 = 8.6 Hz), 7.27 (dd, 1H, J_1 = 2.0 Hz), 7.22 (d, 1H, J_1 = 1.7 Hz).

^{13}C NMR (75.5 MHz, DMSO-d^6) δ ppm: 161.49, 154.59, 143.36, 140.06, 137.67, 127.38, 123.36, 116.99, 115.51, 104.04.

5(6)-chloro-2-(5-methylfuran-2-yl)-1*H*-benzo[*d*]imidazole (3e):

Following the general procedure 1.

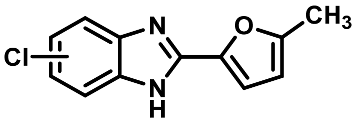

White solid. Yield = 51%. mp: 198 – 199 °C. (*)

^{1}H NMR (300 MHz, DMSO-d^6) δ ppm: 13.0 (brs, 1H), 7.57(s, 1H), 7.52 (d, 1H, J_1 = 8.4 Hz), 7.19 (dd, 1H, J_1 = 8.4 Hz, J_2 = 2.0 Hz), 7.11 (d, 1H, J_1 = 3.3 Hz), 6.33 (dd, 1H, J_1 = 3.3 Hz, J_2 = 1.1 Hz), 2.39 (s, 3H).

^{13}**C NMR** (75.5 MHz, DMSO-d^6) δ ppm: 154.15, 145.12, 143.55, 126.32, 122.23, 117.69 112.28, 108.69, 13.46.

(*) Akande, A.A., et al., *Substituted benzimidazole analogues as potential α-amylase inhibitors and radical scavengers.* ACS omega, 2021. **6**(35): p. 22726-22739.

5(6)-chloro-2-(furan-2-yl)-1*H*-benzo[*d*]imidazole (3f):

Following the general procedure 1.

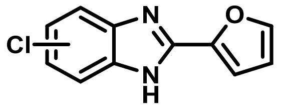

White solid. Yield = 58%.   mp: 200 – 202 °C. (*)

^{1}**H NMR** (300 MHz, DMSO-d^6) δ ppm: 13.12 (brs, 1H), 7.95 (d, 1H, J_1 = 2.2 Hz), 7.68-7.52, (m, 2H), 7.24-7.20 (m, 2H), 6.73 (dd, 1H, J_1 = 3.5 Hz, J_2 = 1.7 Hz).

^{13}**C NMR** (75.5 MHz, DMSO-d^6) δ ppm: 145.12, 144.99, 144.71, 142.50, 135.06, 126.25, 122.67, 119.99, 118.15, 112.43, 111.19.

* Pham, E.C., T.V.T. Le, and T.N. Truong, *Design, synthesis, bio-evaluation, and in silico studies of some N-substituted 6-(chloro/nitro)-1 H-benzimidazole derivatives as antimicrobial and anticancer agents.* RSC advances, 2022. **12**(33): p. 21621-21646.

5(6)-fluoro-2-(pyridin-3-yl)-1*H*-benzo[*d*]imidazole (3a'):

Following the general procedure 1.

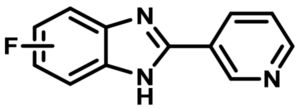

White solid. Yield = 35%.   mp: 241 – 243 °C. (*)

^{1}**H NMR** (300 MHz, DMSO-d^6) δ ppm: 13.06 (brs, 1H), 9.33 (s, 1H), 8,67 (d, 1H, J_1 = 4.8 Hz), 8,47 (d, 1H, J_1 = 8.0 Hz), 7.64-7.54 (m, 2H), 7.43 (d, 1H, J_1 = 8.3 Hz), 7.08 (td, 1H, J_1 = 9.3 Hz, J_2 = 2.5 Hz).

^{13}**C NMR** (75.5 MHz, DMSO-d^6) δ ppm: 158.9 (d, $J_{F,C}$ = 235.7 Hz), 150.69, 150.31, 147.55, 133.84, 125.96, 124.08, 116.13, 110.7 (d, $J_{F,C}$ = 25.7 Hz), 101.08.

(*) Kumar, A., R.A. Maurya, and D. Saxena, *Diversity-oriented synthesis of benzimidazole, benzoxazole, benzothiazole and quinazolin-4 (3H)-one libraries via potassium persulfate–CuSO4-mediated oxidative coupling reactions of aldehydes in aqueous micelles.* Molecular diversity, 2010. **14**(2): p. 331-341.

5(6)-fluoro-2-(3-methoxyphenyl)-1*H*-benzo[*d*]imidazole (3b’):

Following the general procedure 1

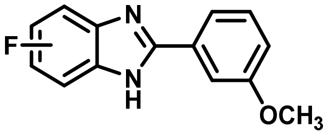

White solid. Yield = 20%. mp = 211.1 – 212.2 °C.

^{1}H NMR (300 MHz, DMSO-d^6) δ ppm: 13.04 (brs, 1H), 7.78-7.75 (m, 2H), 7.60 (brs, 1H), 7.45 (t, 2H, J_1 = 8.2 Hz), 7.10-7.04 (m, 2H), 3.86 (s, 3H).

^{13}C NMR (75.5 MHz, DMSO-d^6) δ ppm: 158.70 (d, $J_{F,C}$ = 233.5 Hz), 159.67, 152.90 – 152.22 (broad band), 144.44 – 143.98 (broad band), 131.23, 130.13, 120.28 – 119.37 (broad band), 118.77, 116.03, 111.43, 110.70 – 109.87 (broad band), 104.86 – 103.59 (broad band), 98.23 – 97.03 (broad band), 55.28.

Note: Carbon signals are observed as clustered peaks within the reported range.

3-(5(6)-fluoro-1*H*-benzo[*d*]imidazol-2-yl)-5-methylisoxazole (3c’):

Following the general procedure 2

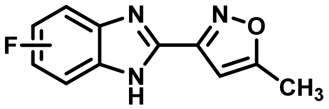

White solid. Yield = 40%. mp = 256.8 – 257.7 °C.

^{1}H NMR (300 MHz, DMSO-d^6) δ ppm: 13.46 (brs, 1H), 7.66, (dd, 1H, J_1 = 8.9 Hz, J_2 = 4.9 Hz), 7.43 (d, 1H, J_1 = 8.9 Hz), 7.15 (td, 1H, J_1 = 9.3 Hz, J_2 = 2.5 Hz), 6.83 (s, 1H), 2.52 (s, 3H).

^{13}C NMR (75.5 MHz, DMSO-d^6) δ ppm: 171.13, 159.05 (d, $J_{F,C}$ = 233.5 Hz), 155.51, 143.54, 118.62 – 115.65 (broad band), 111.34 (d, $J_{F,C}$ = 25.35 Hz), 100.94, 11.85. (a carbon signal was not found).

Note: Carbon signals are observed as clustered peaks within the reported range.

3-(5(6)-fluoro-1*H*-benzo[*d*]imidazol-2-yl)isoxazole (3d'):

Following the general procedure 2

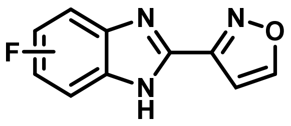

White solid. Yield = 38%. mp = 237.1 – 238.3 °C.

^{1}H NMR (300 MHz, DMSO-d^{6}) δ ppm: ^{1}H RMN (300 MHz, DMSO-d^{6}) δ ppm: 13.49 (brs, 1H), 9.17 (s, 1H), 7.75-7.19 (m, 4H,).

^{13}C NMR (75.5 MHz, DMSO-d^{6}) δ ppm: 161.47, 154.61, 143.89 – 142.86 (broad band), 140.29 – 139.80 (broad band), 134.98 – 134.46 (broad band), 131.57 – 131.12 (broad band), 121.03 – 120.41 (broad band), 113.28 – 110.41 (broad band), 105.27 – 104.40 (broad band), 103.92, 98.69 – 97.84 (broad band).

Note: Carbon signals are observed as clustered peaks within the reported range.

5(6)-fluoro-2-(5-methylfuran-2-yl)-1*H*-benzo[*d*]imidazole (3e'):

Following the general procedure 1

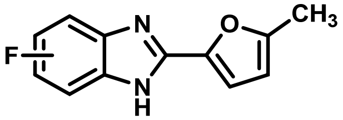

White solid. Yield = 60%. mp: 226 -227 C. (*)

^{1}H NMR (300 MHz, DMSO-d^{6}) δ ppm: 12.94 (brs, 1H), 7.52 (dd, 1H, J_1 = 8.8 Hz, J_2 = 4.9 Hz), 7.32 (d, 1H, J_1 = 8.8 Hz), 7.09 (d, 1H J_1 = 3.4 Hz), 7.02 (ddd, 1H, J_1 = 9.9 Hz, J_2 = 8.8 Hz, J_3 = 2.5 Hz), 6.32 (dd, 1H, J_1 = 3.4 Hz, J_2 = 0.8 Hz), 2.39 (s, 3H).

^{13}C NMR (75.5 MHz, DMSO-d^{6}) δ ppm: 158.67 (d, $J_{F,C}$ = 234.9 Hz), 153.92, 145.19, 143.74, 111.85, 110.02 (d, $J_{F,C}$ = 25.1 Hz), 108.61, 13.44. (a carbon signal was not found)

(*) Akande, A.A., et al., *Substituted benzimidazole analogues as potential α-amylase inhibitors and radical scavengers.* ACS omega, 2021. **6**(35): p. 22726-22739.

5(6)-fluoro-2-(furan-2-yl)-1*H*-benzo[*d*]imidazole (3f'):

Following the general procedure 1

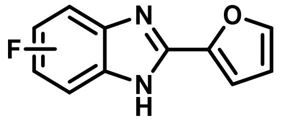

White solid. Yield = 47%. mp: 218 – 220 °C. (*)

^{1}H NMR (300 MHz, DMSO-d^{6}) δ ppm: 13.06 (brs, 1H), 7.94 (d, 1H, J_1 = 1.0 Hz), 7.55 (brs, 1H), 7.36 (brs, 1H), 7.21 (d, 1H, J_1 = 3.4 Hz), 7.05 (td, 1H, J_1 = 9.3 Hz, J_2 = 2.0 Hz), 6.72 (dd, 1H, J_1 = 3.4 Hz, J_2 = 1.7 Hz).

^{13}C NMR (75.5 MHz, DMSO-d^{6}) δ ppm: 158.76 (d, $J_{F,C}$ = 233.91 Hz), 145.29, 144.80, 120.48 – 118.46 (broad band), 112.37, 110.77, 110.58 – 109.89 (broad band), 105.00 – 103.53 (broad band), 98.57 – 97.27 (broad band).

Note: Carbon signals are observed as clustered peaks within the reported range.

(*) Akande, A.A., et al., *Substituted benzimidazole analogues as potential α-amylase inhibitors and radical scavengers.* ACS omega, 2021. **6**(35): p. 22726-22739.

3) Procedure for the synthesis of (5 or 6)-(chloro or fluoro)-2-(aryl)-1H-benzo[d]imidazol-1-yl)(naphtalen-1-yl)methanone (4a-f)/ (4a'-f') and (5a-f)/(5a'-f')

To a solution of the corresponding 6-chloro(or–fluoro)-2-aryl-1*H*-benzo[*d*]imidazole (**3a-f** and **3a'-b'**) (170 mg. 1 eq) and sodium hydride (NaH) (2.8 eq.) in dry THF (50 mL) under anaerobic conditions (N_2) and magnetic stirring was added dropwise 1-naphthoyl chloride (1.3 eq). After addition, reaction was stirred at room temperature for 30 min. Then, the remaining NaH was removed by filtration and the filtrate was poured over 200 mL of water and subsequently extracted with ethyl acetate (3 x 100 mL). The organic portions were dried with anhydrous Na_2SO_4, filtered and concentrated under reduced pressure in a rotary evaporator. The residue was purified by thin layer chromatography with 20% ethyl acetate and 80% hexane as eluent to yield target regioisomers (**4a-f**)/ (**4a'-f'**) and (**5a-f**)/(**5a'-f'**).

(5-chloro-2-(pyridin-3-yl)-1*H*-benzo[*d*]imidazol-1-yl)(naphthalen-1-yl)methanone (4a):

Following the general procedure 3.

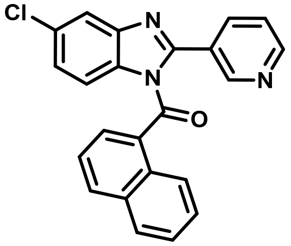

White solid. Yield = 28%. mp: 195.3 – 196.8 °C.

^{1}H NMR (300 MHz, DMSO-d^6) δ ppm: 8.57 (d, 1H, J_1 = 2,0 Hz), 8.29 (dd, 1H, J_1 = 4.9 Hz, J_2 = 1.7 Hz), 8.03 (d, 2H, J_1 = 8.1 Hz), 8.00 (d, 1H, J_1 = 1.7 Hz), 7.95 (dd, 1H, J_1 = 7.6 Hz, J_2 = 2.6 Hz), 7.83-7.78 (m, 2H), 7.68-7.58 (m, 3H), 7.48 (dd, 1H, J_1 = 8.7 Hz, J_2 = 2.1 Hz), 7.38 (dd, 1H, J_1 = 8.2Hz, J_2 = 7.2 Hz), 7.00 (ddd, 1H, J_1 = 8.0 Hz, J_2 = 4.9 Hz, J_3 = 1.0 Hz).

^{13}C NMR (75.5 MHz, DMSO-d^6) δ ppm: 167.54, 152.68, 150.10, 148.81, 143.45, 136.15, 133.59, 133.09, 132.89, 130.38, 130.02, 129.90, 129.18, 128.68, 128.50, 126.99, 126.58, 125.50, 124.69, 124.09, 122.28, 119.73, 115.51.

FT-IR (ATR, cm^1) = 1692.61 (C=O).

HRMS (ESI) *m/z* calcd. For $C_{23}H_{14}ClN_3O$ [M$^+$] 383.08254, [M$^+$+H] 384.089816, found [M$^+$+H] 384.0904.

(6-chloro-2-(pyridin-3-yl)-1H-benzo[d]imidazol-1-yl)(naphthalen-1-yl)methanone (4a'):

Following the general procedure 3.

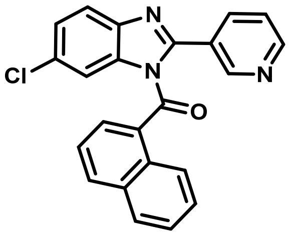

White solid. Yield = 24%. mp: 154.9 – 155.8°C.

^{1}H NMR (300 MHz, $CDCl_3$) δ ppm: 8.50 (d, 1H, J_1 = 2.0 Hz), 8.19 (d, 1H, J_1 = 3.7 Hz), 7.93-7.89 (m, 1H), 7.81-7.70 (m, 4H), 7.56-7.44 (m, 4H), 7.35 (dd, 1H, J_1 = 8.6 Hz, J_2 = 2.0 Hz), 7.23 (m, 1H), 6.76 (dd, 1H, J_1 = 7.7 Hz, J_2 = 4.9 Hz).

^{13}**C NMR** (75.5 MHz, $CDCl_3$) δ ppm: 168.14, 151.99, 150.31, 149.18, 141.54, 135.64, 135.20, 134.28, 133.52, 131,66, 130.79, 130.36, 130.30, 128.98, 128.93, 127.31, 126.77, 126.05, 124.51, 124.15, 122.21, 121.37, 114.69.

FT-IR (ATR, cm^1) = 1697.38 (C=O).

HRMS (ESI) *m/z* calcd. For $C_{23}H_{14}ClN_3O$ [M^+] 383.08254, [M^++H] 384.089816, found [M^++H] 384.0904.

(5-chloro-2-(3-methoxyphenyl)-1*H*-benzo[*d*]imidazol-1-yl)(naphthalen-1-yl)methanone (4b):

Following the general procedure 3.

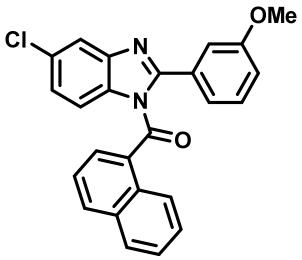

White solid. Yield = 37%. mp: 157.2 – 158.1 °C.

^{1}**H NMR** (300 MHz, DMSO-d^6) δ ppm: 8.17 (d, 1H, J_1 = 8.3 Hz), 8.00 (d, 1H, J_1 = 8.3 Hz), 7.97-7.95 (m, 2H), 7.78 (d, 1H, J_1 = 8.8 Hz), 7.69-7.59 (m, 3H), 7.47 (dd, 1H, J_1 = 8.7 Hz, J_2 = 2.1 Hz), 7.34-7.29 (m, 1H), 7.00-6.89 (m, 3H), 6.67-6.63 (m, 1H), 3.45 (s, 3H).

^{13}**C NMR** (75.5 MHz, DMSO-d^6) δ ppm: 167.94, 158.23, 155.03, 143.37, 133.54, 133.26, 132.90, 131.41, 130.32, 130.13, 130.06, 129.00, 128.84, 128.65, 128.31, 126.81, 125.16, 124.50, 124.34, 121.40, 119.56, 115.99, 115.21, 113.70, 54.89.

FT-IR (ATR, cm^1) = 1693.72 (C=O).

HRMS (ESI) *m/z* calcd. For $C_{23}H_{14}ClN_3O$ [M^+] 412.097855, [M^++H] 413.105132, found [M^++H] 413.1066.

(6-chloro-2-(3-methoxyphenyl)-1*H*-benzo[*d*]imidazol-1-yl)(naphthalen-1-yl)methanone (4b'):

Following the general procedure 3.

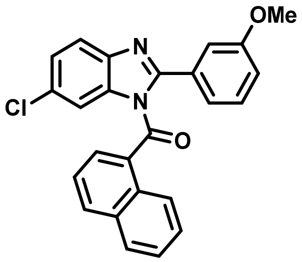

White solid. Yield = 40%. mp: 146.7 – 147.9 °C.

^{1}H NMR (300 MHz, DMSO-d^6) δ ppm: 8.17 (d, 1H, J_1 = 8.4 Hz), 7.98 (d, 1H, J_1 = 8.4 Hz), 7.94 (d, 1H, J_1 = 8.5 Hz), 7.89 (d, 1H, J_1 = 8.6 Hz), 7.83 (d, 1H, J_1 = 2.0 Hz), 7.69-7.58 (m, 3H), 7.52 (dd, 1H, J_1 = 8.6 Hz, J_2 = 2.0 Hz), 7.32-7.27 (m, 1H), 6.96 (d, 1H, J_1 = 7.6 Hz), 6.91-6.85 (m, 2H), 6.64-6.60 (m, 1H), 3.42 (s, 3H).

^{13}C NMR (75.5 MHz, DMSO-d^6) δ ppm: 167.97, 158.18, 154.48, 141.22, 135.04, 133.45, 132.85, 131.44, 130.27, 130.10, 130.04, 129.36, 128.78, 128.62, 128.27, 126.79, 125.15, 124.45, 124.36, 121.37, 121.32, 115.91, 113.85, 113.65, 54.86.

FT-IR (ATR, cm^1) = 1718.06 (C=O).

HRMS (ESI) *m/z* calcd. For $C_{23}H_{14}ClN_3O$ [M$^+$] 412.097855, [M$^+$+H] 413.105132, found [M$^+$+H] 413.1049.

(5-chloro-2-(5-methylisoxazol-3-yl)-1*H*-benzo[*d*]imidazol-1-yl)(naphthalen-1-yl)methanone (4c):

Following the general procedure 3**.**

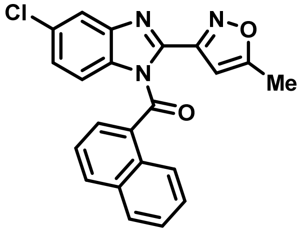

White solid. Yield = 32%. mp: 193.7 – 194.9°C.

^{1}**H NMR** (300 MHz, DMSO-d^6) δ ppm: 8.35 (d, 1H, J_1 = 8.3 Hz), 8.20 (d, 1H, J_1 = 8.2 Hz), 8.07 (d, 1H, J_1 = 7.6 Hz), 8.04 (d, 1H, J_1 = 1.4 Hz), 7.76-7.67 (m, 2H), 7.63 (dd, 1H, J_1 = 7.3 Hz, J_1 = 0.8 Hz), 7.55-7.42 (m, 3H), 6.51 (d, J_1 = 0.7 Hz 1H), 2.22 (s, 3H).

^{13}**C NMR** (75.5 MHz, DMSO-d^6) δ ppm: 170.61, 167.26, 154.73, 144.47, 143.11, 134.42, 133.18, 133.04, 130.51, 130.39, 129.64, 129.20, 128.78, 128.67, 127.14, 126.23, 124.75, 124.60, 120.13, 114.79, 102.26, 11.48.

FT-IR (ATR, cm^1) = 1708.04 (C=O).

HRMS (ESI) *m/z* calcd. For $C_{23}H_{14}ClN_3O$ [M$^+$] 387.077454, [M$^+$+H] 388.084731, found [M$^+$+H] 388.0862.

<u>(6-chloro-2-(5-methylisoxazol-3-yl)-1*H*-benzo[*d*]imidazol-1-yl)(naphthalen-1-yl)methanone (4c')</u>:

Following the general procedure 3.

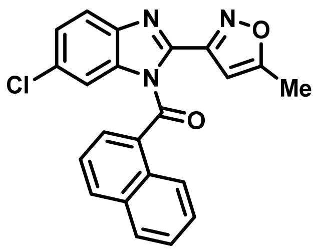

White solid. Yield = 28 %.   mp: 140.3 – 141.1°C.

^{1}**H NMR** (300 MHz, DMSO-d^6) δ ppm: 8.35 (d, 1H, J_1 = 8.3 Hz), 8.19, (d, 1H, J_1 = 8.3 Hz), 8.07 (d, 1H, J_1 = 7.4 Hz), 7.95 (d, 1H, J_1 = 8.6 Hz), 7.75-7.60 (m, 4H), 7.53 (dd, 1H, J_1 = 8.6 Hz, J_2 = 2.0 Hz), 6.48 (d, J_1 = 0.4 Hz, 1H), 2.20 (s, 3H).

^{13}**C NMR** (75.5 MHz, DMSO-d^6) δ ppm: 170.50, 167.24, 154.71, 143.94, 141.01, 134.80, 134.31, 133.12, 130.41, 130.38, 129.65, 128.71, 128.60, 127.09, 125.38, 124.66, 124.64, 121.97, 113.36, 102.21, 11.43. (a carbon signal was not found).

FT-IR (ATR, cm^1) = 1709.68 (C=O).

HRMS (ESI) *m/z* calcd. For $C_{23}H_{14}ClN_3O$ [M$^+$] 387.077454, [M$^+$+H] 388.084731, found 388.0841 [M$^+$+H].

(5-chloro-2-(isoxazol-3-yl)-1*H*-benzo[*d*]imidazol-1-yl)(naphthalen-1-yl)methanone (4d):

Following the general procedure 3**.**

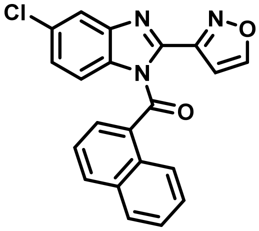

White solid. Yield = 28%. mp: 169.3 – 170.2 °C.

^{1}H NMR (300 MHz, DMSO-d^6) δ ppm: 8.83 (d, 1H, J_1 = 1.7 Hz), 8.35, (d, 1H, J_1 = 8.3 Hz), 8.18 (d, 1H, J_1 = 8.3 Hz), 8.06-8.04 (m, 2H), 7.75-7.63 (m, 3H), 7.57 (d, 1H, J_1 = 8.7 Hz), 7.50 (dd, 1H, J_1 = 8.8 Hz, J_2 = 1.9 Hz), 7.43 (t, 1H, J_1 = 7.7), 6.89 (d, 1H, J_1 = 1.7 Hz).

^{13}C NMR (75.5 MHz, DMSO-d^6) δ ppm: 167.14, 160.92, 153.90, 144.19, 143.11, 134.44, 133.14, 133.06, 130.52, 130.35, 129.60, 129.27, 128.77, 128.65, 127.11, 126.30, 124.66, 124.57, 120.14, 114.88, 104.12.

FT-IR (ATR, cm^1) = 1694.76 (C=O).

HRMS (ESI) *m/z* calcd. For $C_{23}H_{14}ClN_3O$ [M$^+$] 373.061804, [M$^+$+H] 374.069081, found [M$^+$+H] 374.0718.

(6-chloro-2-(isoxazol-3-yl)-1*H*-benzo[*d*]imidazol-1-yl)(naphthalen-1-yl)methanone (4d'):

Following the general procedure 3.

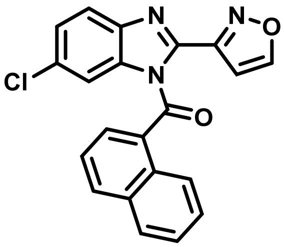

White solid. Yield = 26%. mp: 178.9 – 179.1°C.

^{1}H NMR (300 MHz, DMSO-d^6) δ ppm: 8.80 (d, 1H, J_1 = 1.7 Hz), 8.36 (d, 1H, J_1 = 8.4 Hz), 8.17 (d, 1H, J_1 = 8.3 Hz), 8.05 (d, 1H, J_1 = 7.6 Hz), 7.97 (d, 1H, J_1 = 8.6 Hz), 7.75-7.62 (m, 4H), 7.54 (dd, 1H, J_1 = 8.6 Hz, J_1 = 2.0 Hz), 7.42 (t, 1H, J_1 = 7.7), 6.86 (d, 1H, J_1 = 1.7 Hz).

^{13}C NMR (75.5 MHz, DMSO-d^{6}) δ ppm: 167.16, 160.86, 153.91, 143.69, 141.04, 134.84, 134.38, 133.10, 130.51, 130.47, 130.37, 129.62, 128.75, 128.62, 127.11, 125.48, 124.63, 124.61, 122.01, 113.49, 105.09.

FT-IR (ATR, cm^{1}) = 1690.24 (C=O).

HRMS (ESI) *m/z* calcd. For $C_{23}H_{14}ClN_3O$ [M^{+}] 373.061804, [M^{+}+H] 374.069081, found [M^{+}+H] 374.0714.

<u>(5-chloro-2-(5-methylfuran-2-yl)-1*H*-benzo[*d*]imidazol-1-yl)(naphthalen-1-yl)methanone (4e):</u>

Following the general procedure 3.

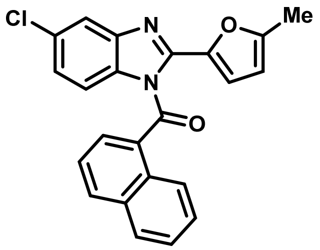

White solid. Yield = 35%. mp: 145.9 – 146.9°C.

^{1}H NMR (300 MHz, DMSO-d^{6}) δ ppm: 8.47 (dd, 1H, J_1 = 8.5 Hz, J_2 = 3.0 Hz), 8.14-8.06 (m, 2H), 7.91-7.90 (m, 1H), 7.77 (dd, 2H, J_1 = 8.5 Hz, J_2 = 3.0 Hz), 7.68 (tt, 1H, J_1 = 7.0 Hz, J_1 = 2.7 Hz), 7.47-7.40 (m, 2H), 7.34 (td, 1H, J_1 = 7.6 Hz, J_1 = 3.4 Hz), 6.89 (t, 1H, J_1 = 3.2 Hz), 5.90-5.88 (m, 1H), 1.31 (d, 3H, J_1 = 2.9 Hz).

^{13}C NMR (75.5 MHz, DMSO-d^{6}) δ ppm: 167.89, 155.00, 145.30, 143.49, 141.57, 133.80, 133.09, 132.83, 130.40, 130.04, 129.23, 129.08, 128.81, 128.56, 126.91, 125.03, 124.61, 124.48, 119.25, 115.18, 114.53, 108.28, 11.83.

FT-IR (ATR, cm^{1}) = 1707.09 (C=O).

HRMS (ESI) *m/z* calcd. For $C_{23}H_{14}ClN_3O$ [M^{+}] 386.082205, [M^{+}+H] 387.089482, found [M^{+}+H] 387.0921.

(6-chloro-2-(5-methylfuran-2-yl)-1*H*-benzo[*d*]imidazol-1-yl)(naphthalen-1-yl)methanone (4e'):

Following the general procedure 3.

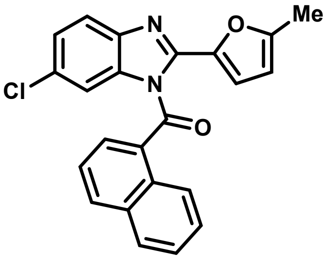

White solid. Yield = 36%. mp: 145.0 – 145.8°C.

^{1}H NMR (300 MHz, $CDCl_3$) δ ppm: 8.58 (d, 1H, J_1 = 8.5 Hz), 7.93-7.87 (m, 2H), 7.80 (d, 1H, J_1 = 1.7 Hz), 7.73-7.54 (m, 3H), 7.30-7.18 (m, 3H), 6.83 (d, 1H, J_1 = 2.1 Hz), 5.72 (d, 1H, J_1 = 2.1 Hz), 1.44 (s, 3H).

^{13}C NMR (75.5 MHz, $CDCl_3$) δ ppm: 168.18, 155.52, 145.95, 143.80, 142.12, 133.77, 133.61, 133.05, 131.21, 130.83, 130.36, 128.94, 128.69, 128.61, 126.99, 125.23, 124.12, 119.78, 115.19, 114.33, 108.10, 12.48. (a carbon signal was not found).

FT-IR (ATR, cm^1) = 1710.42 (C=O).

HRMS (ESI) *m/z* calcd. For $C_{23}H_{14}ClN_3O$ [M^+] 386.082205, [M^++H] 387.089482, found [M^++H] 387.0869.

(5-chloro-2-(furan-2-yl)-1*H*-benzo[*d*]imidazol-1-yl)(naphthalen-1-yl)methanone (4f):

Following the general procedure 3.

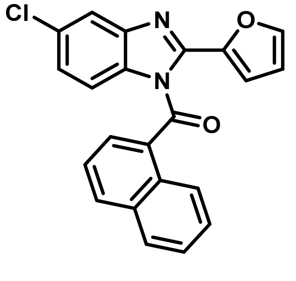

White solid. Yield = 42%. mp: 157.2 – 158.0°C.

^{1}H NMR (300 MHz, $CDCl_3$) δ ppm: 8.49 (d, 1H, J_1 = 8.5 Hz), 7.98 (d, 1H, J_1 = 8.0 Hz), 7.90 (d, 1H, J_1 = 7.9 Hz), 7.82, (d, 1H, J_1 = 1.5 Hz), 7.71-7.56 (m, 2H), 7.44-7.20 (m, 4H), 6.93-6.90 (m, 2H), 6.21 (dd, 1H, J_1 = 3.4 Hz, J_2 = 1.7 Hz).

^{13}C NMR (75.5 MHz, $CDCl_3$) δ ppm:167.99, 145.86, 144.89, 144.02, 143.78, 134.29, 133.82, 132.99, 131.04, 130.43, 129.75, 129.22, 128.76, 127.16, 125.50, 124.95, 124.31, 120.14, 114.24, 113.85, 111.86. (a carbon signal were not found).

FT-IR (ATR, cm^1) = 1694.53 (C=O).

HRMS (ESI) *m/z* calcd. For $C_{23}H_{14}ClN_3O$ [M^+] 372.066555, [M^++H] 373.073832, found [M^++H] 373.0735.

(6-chloro-2-(furan-2-yl)-1*H*-benzo[*d*]imidazol-1-yl)(naphthalen-1-yl)methanone (4f'):

Following the general procedure 3.

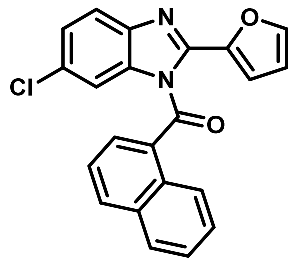

White solid. Yield = 39%. mp: 148.7 – 149.8 °C.

^1H NMR (300 MHz, $CDCl_3$) δ ppm: 8.51 (d, 1H, J_1 = 8.3 Hz), 7.95 (d, 1H, J_1 = 8.1 Hz), 7.88 (d, 1H, J_1 = 7.5 Hz), 7.75 (d, 1H, J_1 = 8.6 Hz), 7.71-7.55 (m, 3H), 7.41-7.23 (m, 3H), 6.86-6.85 (m, 2H), 6.16 (dd, 1H, J_1 = 3.2 Hz, J_1 = 2.0 Hz).

^{13}C NMR (75.5 MHz, $CDCl_3$) δ ppm: 167.95, 145.27, 144.71, 143.98, 141.44, 134.88, 134.26, 133.74, 131.01, 130.93, 130.29, 129.66, 128.70, 127.08, 125.58, 124.94, 124.19, 121.03, 113.66, 113.47, 111.76. (a carbon signal was not found).

FT-IR (ATR, cm^1) = 1709.00 (C=O).

HRMS (ESI) *m/z* calcd. For $C_{23}H_{14}ClN_3O$ [M^+] 372.066555, [M^++H] 373.073832, found [M^++H] 373.0772.

(5-fluoro-2-(pyridin-3-yl)-1*H*-benzo[*d*]imidazol-1-yl)(naphthalen-1-yl)methanone (5a):

Following the general procedure 3.

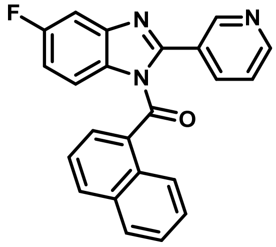

White solid. Yield = 48%.   mp: 158.6 – 159.7°C.

^{1}H NMR (300 MHz, DMSO-d^6) δ ppm: 8.57 (d, 1H, J_1 = 1.6 Hz), 8.28 (dd, 1H, J_1 = 4.9 Hz, J_2 = 1.5 Hz), 8.02 (d, 2H, J_1 = 8.0 Hz), 7.95 (dd, 1H, J_1 = 7.5 Hz, J_2 = 1.4 Hz), 7.81-7.74 (m, 3H), 7.69 (dd, 1H, J_1 = 9.2 Hz, J_2 = 3.7 Hz), 7.61 (qd, 2H, J_1 = 7.5 Hz, J_2 = 6.9 Hz, J_3 = 1.4 Hz), 7.37 (t, 1H, J_1 = 8.0 Hz) 7.31 (td, 1H J_1 = 9.2 Hz, J_2 = 3.7 Hz), 6.99 (dd, 1H, J_1 = 7.9 Hz, J_2 = 4.9 Hz).

^{13}C NMR (75.5 MHz, DMSO-d^6) δ ppm: 167.59, 159.75 (d, $J_{F,C}$ = 238.9 Hz), 152.88, 150.03, 148.78, 143.19 (d, $J_{F,C}$ = 12.7 Hz), 136.09, 133.45, 132.87, 130.90, 130.24, 130.18, 129.90, 128.66, 128.45, 126.96, 126.70, 124.68, 124.08, 122.24, 115.24 (d, $J_{F,C}$ = 9.8 Hz), 113.25 (d, $J_{F,C}$ = 25.4 Hz), 106.21 (d, $J_{F,C}$ = 24.4 Hz).

^{19}F NMR (282 MHz, DMSO-d^6) δ ppm: -117.12 (s, 1F).

FT-IR (ATR, cm^1) = 1690.44 (C=O).

HRMS (ESI) *m/z* calcd. For $C_{23}H_{14}ClN_3O$ [M$^+$] 367.11209, [M$^+$+H] 368.119367, found [M$^+$+H] 368.1205.

(6-fluoro-2-(pyridin-3-yl)-1*H*-benzo[*d*]imidazol-1-yl)(naphthalen-1-yl)methanone (5a'):

Following the general procedure 3.

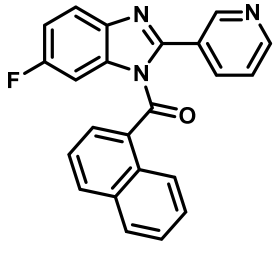

White solid. Yield = 42%.   mp: 135.5 – 136.7°C.

^{1}H NMR (300 MHz, DMSO-d^6) δ ppm: 8.54 (dd, 1H, J_1 = 0.4 Hz), 8.25 (d, 1H, J_1 = 4.4 Hz), 8.01 (d, 2H, J_1 = 8.16 Hz), 7.95-7.90 (m, 2H), 7.76 (d, 2H, J_1 = 7.5 Hz), 7.63 (p, 2H, J_1 = 6.60 Hz), 7.48 (dd, 1H, J_1 = 9.3 Hz, J_2 = 2.3 Hz), 7.37 (t, 2H, J_1 = 7.5 Hz), 6.96 (dd, 1H, J_1 = 7.5 Hz, J_1 = 4.4 Hz).

^{13}C NMR (75.5 MHz, DMSO-d^6) δ ppm: 167.64, 160.04 (d, $J_{F,C}$ = 240.3 Hz), 151.88, 151.84, 149.87, 148.71, 139.08, 136.01, 134.56, 134.37, 133.43, 132.83, 130.20, 129.97 (d, $J_{F,C}$ = 18.3 Hz), 128.65, 128.44, 126.96, 126.73, 124.64, 124.07, 122.17, 121.37 (d, $J_{F,C}$ = 10.2 Hz), 113.03 (d, $J_{F,C}$ = 24.7 Hz), 101.37 (d, $J_{F,C}$ = 29.5 Hz).

^{19}F NMR (282 MHz, DMSO-d^6) δ ppm: -115.32 (s, 1F).

FT-IR (ATR, cm^1) = 1714.26 (C=O).

HRMS (ESI) *m/z* calcd. For $C_{23}H_{14}ClN_3O$ [M$^+$] 367.11209, [M$^+$+H] 368.119367, found [M$^+$+H] 368.1168.

(5-fluoro-2-(3-methoxyphenyl)-1*H*-benzo[*d*]imidazol-1-yl)(naphthalen-1-yl)methanone (5b):

Following the general procedure 3.

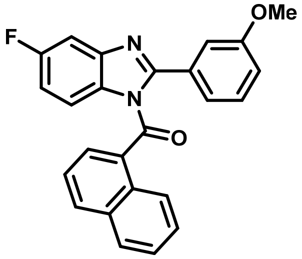

White solid. Yield = 37%. mp: 122.1 – 123.3°C.

^{1}H NMR (300 MHz, DMSO-d^6) δ ppm: 8.16 (d, 1H, J_1 = 7.7 Hz), 7.97 (td, 2H, J_1 = 7.7 Hz, J_2 = 2.4 Hz), 7.79 (dd, 1H, J_1 = 9.2 Hz, J_2 = 5.1 Hz), 7.74-7.70 (m, 1H), 7.67-7.56 (m, 3H), 7.36-7.26 (m, 2H), 7.00-6.85 (m, 3H), 6.64 (dd, 1H, J_1 = 7.9 Hz, J_2 = 1.8 Hz), 3.43 (s, 3H).

^{13}C NMR (75.5 MHz, DMSO-d^6) δ ppm: 168.00, 159.73 (d, $J_{F,C}$ = 233.5 Hz), 158.20, 155.25, 143.09 (d, $J_{F,C}$ = 12.8 Hz), 133.39, 132.89, 131.56, 131.06 (d, $J_{F,C}$ = 1.0 Hz), 130.21, 130.19, 130.13, 128.79, 128.63, 128.25, 126.78, 124.49, 124.34, 121.38, 115.91, 114.91 (d, $J_{F,C}$ = 9.9 Hz), 113.68, 112.87 (d, $J_{F,C}$ = 25.1 Hz), 106.03 (d, $J_{F,C}$ = 24.2 Hz), 54.87.

^{19}F NMR (282 MHz, DMSO-d^6) δ ppm: -117.42 (s, 1F).

FT-IR (ATR, cm^1) = 1694.08 (C=O).

HRMS (ESI) *m/z* calcd. For $C_{23}H_{14}ClN_3O$ [M$^+$] 396.127406, [M$^+$+H] 397.134682, found [M$^+$+H] 397.1380.

(6-fluoro-2-(3-methoxyphenyl)-1*H*-benzo[*d*]imidazol-1-yl)(naphthalen-1-yl)methanone (5b'):

Following the general procedure 3.

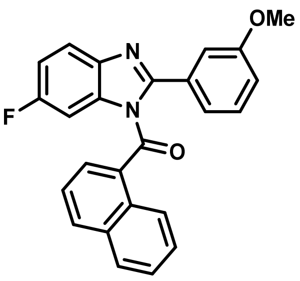

White solid. Yield = 33%.   mp: 148.2 – 149.0°C.

^{1}H NMR (300 MHz, DMSO-d^6) δ ppm: 8.16 (dd, 1H, J_1 = 7.8 Hz, J_2 = 1.8 Hz), 7.96 (t, 2H, J_1 = 6.6Hz), 7.92-7.86 (m, 1H), 7.70-7.56 (m, 4H), 7.39-7.32 (m, 1H), 7.30-7.24 (m, 1H), 6.98-6.83 (m, 3H), 6.64-6.58 (m, 1H), 3.41 (s, 3H).

^{13}C NMR (75.5 MHz, DMSO-d^6) δ ppm: 168.05, 159.93 (d, $J_{F,C}$ = 240.3 Hz), 158.18, 154.28, 154.22 138.96 (d, $J_{F,C}$ = 0.9 Hz), 134.62 (d, $J_{F,C}$ = 13.9 Hz), 133.38, 132.86, 131.62, 130.14, 130.10, 128.75, 128.62, 128.24, 126.77, 124.45, 124.35, 121.31, 121.14 (d, $J_{F,C}$ = 10.2 Hz), 115.77, 113.61, 112.80 (d, $J_{F,C}$ = 24.8 Hz), 101.08 (d, $J_{F,C}$ = 29.9 Hz), 54.87.

^{19}F NMR (282 MHz, DMSO-d^6) δ ppm: -115.87 (s, 1F).

FT-IR (ATR, cm^1) = 1717.75 (C=O).

HRMS (ESI) *m/z* calcd. For $C_{23}H_{14}ClN_3O$ [M$^+$] 396.127406, [M$^+$+H] 397.134682, found [M$^+$+H] 397.1354.

(5-fluoro-2-(5-methylisoxazol-3-yl)-1*H*-benzo[*d*]imidazol-1-yl)(naphthalen-1-yl)methanone (5c):

Following the general procedure 3.

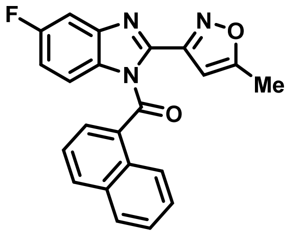

White solid. Yield = 42%.   mp: 147.8 – 148.9°C.

^{1}H NMR (300 MHz, DMSO-d^6) δ ppm: 8.33 (d, 1H, J_1 = 8.3 Hz), 8.19 (d, 1H, J_1 = 8.2 Hz), 7.15 (dd, 1H, J_1 = 7.2 Hz, J_2 = 1.3 Hz), 7.80 (dd, 1H, J_1 = 9.1 Hz, J_2 = 2.4 Hz), 7.75-7.63 (m, 2H), 7.62 (d, 1H, J_1 = 7.2 Hz, J_2 = 1.1 Hz), 7.56 (dd, 1H, J_1 = 9.2 Hz, J_2 = 4.7 Hz), 7.44 (t, 1H, J_1 = 7.7 Hz), 7.33 (td, 1H, J_1 = 9.2 Hz, J_2 = 2.6 Hz), 6.49 (d, 1H, J_1 = 0.8 Hz), 2.21 (s, 3H).

^{13}C NMR (75.5 MHz, DMSO-d^6) δ ppm: 170.48, 167.31, 159.69 (d, $J_{F,C}$ = 239.3 Hz), 154.79, 144.65, 142.81 (d, $J_{F,C}$ = 12.9 Hz), 134.22, 133.14, 130.84 (d, $J_{F,C}$ = 0.8 Hz), 130.36, 130.30, 129.79, 128.72, 128.57, 127.08, 124.70, 124.57, 114.52 (d, $J_{F,C}$ = 10.1 Hz), 114.17 (d, $J_{F,C}$ = 25.7 Hz), 106.47 (d, $J_{F,C}$ = 24.3 Hz), 102.24, 11.44.

^{19}F NMR (282 MHz, DMSO-d^6) δ ppm: -117.12 (s, 1F).

FT-IR (ATR, cm^1) = 1706.49 (C=O).

HRMS (ESI) *m/z* calcd. For $C_{23}H_{14}ClN_3O$ [M$^+$] 371.107005, [M$^+$+H] 372.114281, found [M$^+$+H] 372.1164.

(6-fluoro-2-(5-methylisoxazol-3-yl)-1*H*-benzo[*d*]imidazol-1-yl)(naphthalen-1-yl)methanone (5c'):

Following the general procedure 3.

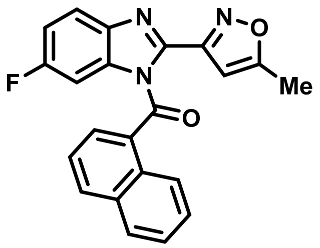

White solid. Yield = 46%.   mp: 142. – 143.2°C.

^{1}H NMR (300 MHz, DMSO-d^6) δ ppm: 8.34 (d, 1H, J_1 = 8.3 Hz), 8.18 (d, 1H, J_1 = 8.2 Hz), 8.06 (dd, 1H, J_1 = 7.2 Hz, J_2 = 1.3 Hz), 7.97 (dd, 1H, J_1 = 9.5 Hz, J_1 = 5.0 Hz), 7.75-7.64 (m, 2H), 7.61 (dd, 1H, J_1 = 7.2 Hz, J_2 = 1.0 Hz), 7.45 (d, 1H, J_1 = 8.0 Hz), 7.41-7.34 (m, 2H), 6.46 (d, 1H, J_1 = 0.8 Hz), 2.19 (s, 3H).

^{13}C NMR (75.5 MHz, DMSO-d^6) δ ppm: 170.40, 167.33, 160.40 (d, $J_{F,C}$ = 241.4 Hz), 154.78, 143.72 138.8 (d, $J_{F,C}$ = 0.8 Hz), 134.44 (d, $J_{F,C}$ = 14.0 Hz), 134.21, 133.12, 130.36, 130.25, 129.75, 128.70, 128.55, 127.07, 124.66, 124.61, 212.94 (d, $J_{F,C}$ = 10.5 Hz), 113.25 (d, $J_{F,C}$ = 25.0 Hz), 102.13, 100.46 (d, $J_{F,C}$ = 29.2 Hz), 11.41.

^{19}F NMR (282 MHz, DMSO-d^6) δ ppm: -114.31 (s, 1F).

FT-IR (ATR, cm^1) = 1711.96 (C=O).

HRMS (ESI) *m/z* calcd. For $C_{23}H_{14}ClN_3O$ [M$^+$] 371.107005, [M$^+$+H] 372.114281, found [M$^+$+H] 372.1154.

(5-fluoro-2-(isoxazol-3-yl)-1*H*-benzo[*d*]imidazol-1-yl)(naphthalen-1-yl)methanone (5d):

Following the general procedure 3.

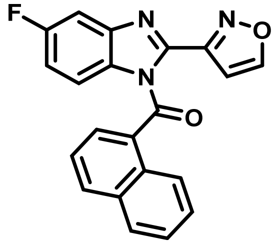

White solid. Yield = 44%. mp: 161.6 – 162.7°C.

^{1}H NMR (300 MHz, DMSO-d^{6}) δ ppm: 8.82 (d, 1H, J_1 = 1.7 Hz), 8.33 (d, 1H, J_1 = 8.3 Hz), 8.17 (d, 1H, J_1 = 8.2 Hz), 8.05 (d, 1H, J_1 = 8.3 Hz), 7.81 (dd, 1H, J_1 = 9.1 Hz, J_2 = 2.5 Hz), 7.72 (m, 1H), 7.64 (m, 2H), 7.59 (t, 1H, J_1 = 4.5 Hz), 7.43 (t, 1H, J_1 = 7.7 Hz), 7.35 (td, 1H, J_1 = 9.3 Hz, J_2 = 2.5 Hz), 6.87 (d, 1H, J_1 = 1.7 Hz).

^{13}C NMR (75.5 MHz, DMSO-d^{6}) δ ppm: 167.22, 159.74 (d, $J_{F,C}$ = 239.3 Hz), 160.85, 153.99, 144.38, 142.83 (d, $J_{F,C}$ = 12.9 Hz), 134.28, 133.12, 130.90, 130.89, 130.35, 129.77, 128.75, 128.59, 127.09, 124.64, 124.57, 114.65 (d, $J_{F,C}$ = 10.7 Hz), 114.29 (d, $J_{F,C}$ = 25.7 Hz), 106.51 (d, $J_{F,C}$ = 24.6 Hz), 105.12.

^{19}F NMR (282 MHz, DMSO-d^{6}) δ ppm: -116.99 (s, 1F).

FT-IR (ATR, cm^{1}) = 1698.67 (C=O).

HRMS (ESI) *m/z* calcd. For $C_{23}H_{14}ClN_3O$ [M^{+}] 357.091355, [M^{+}+H] 358.098631, found [M^{+}+H] 358.0979.

(6-fluoro-2-(isoxazol-3-yl)-1*H*-benzo[*d*]imidazol-1-yl)(naphthalen-1-yl)methanone (5d'):

Following the general procedure 3.

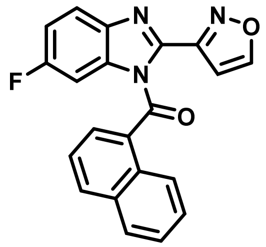

White solid. Yield = 50%. mp: 155.9 – 156.9°C.

^{1}H NMR (300 MHz, DMSO-d^{6}) δ ppm: 8.879 (d, 1H, J_1 = 1.7 Hz), 8.43 (d, 1H, J_1 = 8.2 Hz), 8.17 (d, 1H, J_1 = 8.2 Hz), 8.05 (d, 1H, J_1 = 8.2 Hz), 7.99 (dd, 1H, J_1 = 5.0 Hz, J_2 = 0.8 Hz), 7.70 (m, 2H), 7.62 (dd, 1H, J_1 = 7.3 Hz, J_2 = 0.8 Hz), 7.4 (m, 3H), 6.84 (d, 1H, J_1 = 1.7 Hz).

^{13}C NMR (75.5 MHz, DMSO-d^{6}) δ ppm: 167.25, 160.47 (d, $J_{F,C}$ = 241.6 Hz), 160.78, 153.97, 143.48, 143.43, 138.84 (d, $J_{F,C}$ = 0.9 Hz), 134.49 (d, $J_{F,C}$ = 13.9 Hz), 134.28, 133.09, 130.34, 130.31, 129.71, 128.73, 128.58, 127.08, 124.60, 122.00 (d, $J_{F,C}$ = 10.3 Hz), 113.37 (d, $J_{F,C}$ = 25.08 Hz), 105.00, 100.58 (d, $J_{F,C}$ = 29.2 Hz).

^{19}F NMR (282 MHz, DMSO-d^{6}) δ ppm: -114.16 (s, 1F).

FT-IR (ATR, cm^{1}) = 1712.77 (C=O).

HRMS (ESI) *m/z* calcd. For $C_{23}H_{14}ClN_3O$ [M$^+$] 357.091355, [M$^+$+H] 358.098631, found [M$^+$+H] 358.1001.

(5-fluoro-2-(5-methylfuran-2-yl)-1*H*-benzo[*d*]imidazol-1-yl)(naphthalen-1-yl)methanone (5e):

Following the general procedure 3.

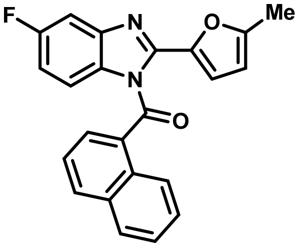

White solid. Yield = 45%. mp: 121.6 – 122.5°C.

^{1}H NMR (300 MHz, DMSO-d^{6}) δ ppm: 8.46 (d, 1H, J_1 = 8.4 Hz), 8.12-8.06 (m, 2H), 7.82-7.76 (m, 2H), 7.70-7.65 (m, 2H), 7.41-7.33 (m, 2H), 7.29 (td, 1H J_1 = 9.0 Hz, J_2 = 2.4 Hz), 6.87 (d, 1H, J_1 = 6.9 Hz), 5.88 (dd, 1H, J_1 = 2.5 Hz, J_2 = 0.8 Hz), 1.30 (s, 3H).

^{13}C NMR (75.5 MHz, DMSO-d^{6}) δ ppm: 167.95, 159.83 (d, $J_{F,C}$ = 238.5 Hz), 154.87, 145.53, 143.21 (d, $J_{F,C}$ = 12.9 Hz), 141.69, 133.64, 133.08, 130.62 (d, $J_{F,C}$ = 0.8 Hz), 130.41, 130.19, 129.07, 128.79, 128.50, 126.88, 124.63, 124.47, 114.98, 114.24 (d, $J_{F,C}$ = 10.1 Hz), 112.79 (d, $J_{F,C}$ = 25.5 Hz), 108.21 105.74 (d, $J_{F,C}$ = 24.6 Hz), 11.80.

^{19}F NMR (282 MHz, DMSO-d^{6}) δ ppm: -117.38 (s, 1F).

FT-IR (ATR, cm^{1}) = 1708.42 (C=O).

HRMS (ESI) *m/z* calcd. For $C_{23}H_{14}ClN_3O$ [M$^+$] 370.111756, [M$^+$+H] 371.119032, found [M$^+$+H] 371.1196.

(6-fluoro-2-(5-methylfuran-2-yl)-1*H*-benzo[*d*]imidazol-1-yl)(naphthalen-1-yl)methanone (5e'):

Following the general procedure 3.

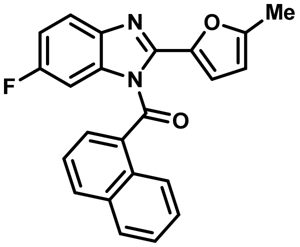

White solid. Yield = 40%. mp: 116.5 – 117.4°C.

^{1}H NMR (300 MHz, DMSO-d^6) δ ppm: 8.46 (d, 1H, J_1 = 8.4 Hz), 8.12-8.06 (m, 2H), 7.85, (dd, 1H, J_1 = 8.8 Hz, J_2 = 4.9 Hz), 7.78 (td, 1H, J_1 = 7.7 Hz, J_2 = 1.2 Hz), 7.67 (td, 1H, J_1 = 7.6 Hz, J_2 = 0.9 Hz), 7.60 (d, 1H, J_1 = 9.1 Hz, J_2 = 2.4 Hz), 7.42 (dd, 1H, J_1 = 7.2 Hz, J_2 = 1,1 Hz), 7.36-7.29 (m, 2H), 6.82 (d, 1H, J_1 = 3.3 Hz), 5.85 (dd, 1H J_1 = 3.3 Hz, J_1 = 0.9 Hz), 1.27 (s, 3H).

^{13}C NMR (75.5 MHz, DMSO-d^6) δ ppm: 167.98, 159.95 (d, $J_{F,C}$ = 240.1 Hz), 154.58, 144.67, 141.69, 139.04 (d, $J_{F,C}$ = 0.9 Hz), 134.18 (d, $J_{F,C}$ = 13.9 Hz), 133.63, 133.05, 130.38, 130.05, 129.07, 128.78, 128.48, 126.86, 124.61, 124.43, 120.92 (d, $J_{F,C}$ = 10.11 Hz), 114.41, 112.91 (d, $J_{F,C}$ = 24.83 Hz), 108.06, 100.49 (d, $J_{F,C}$ = 29.2 Hz), 11.75.

^{19}F NMR (282 MHz, DMSO-d^6) δ ppm: -115.81 (s, 1F).

FT-IR (ATR, cm^1) = 1707.49 (C=O).

HRMS (ESI) *m/z* calcd. For $C_{23}H_{14}ClN_3O$ [M$^+$] 370.111756, [M$^+$+H] 371.119032, found [M$^+$+H] 371.1187.

(5-fluoro-2-(furan-2-yl)-1*H*-benzo[*d*]imidazol-1-yl)(naphthalen-1-yl)methanone (5f):

Following the general procedure 3.

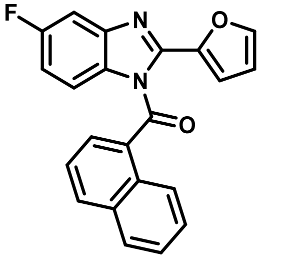

White solid. Yield = 39%. mp: 131.5 - 132.0°C.

^{1}H NMR (300 MHz, DMSO-d^{6}) δ ppm: 8.38, (d, 1H, J_1 = 8.3 Hz), 8.17 (d, 1H, J_1 = 8.2 Hz), 8.06 (d, 1H, J_1 = 7.6 Hz), 7.76-7.67 (m, 3H), 7.24 (d, 1H, J_1 = 7.2 Hz), 7.45 (d, 1H, J_1 = 7.6 Hz), 7.40 (dd, 1H J_1 = 9.2 Hz, J_2 = 4.6 Hz), 7.27 (d, 1H, J_1 = 1.0 Hz), 7.21 (td, 1H, J_1 = 9.4 Hz, J_2 = 2.5 Hz), 6.97 (d, 1H, J_1 = 3.5 Hz), 6.36, (dd, 1H, J_1 = 3.5 Hz, J_2 = 1.7 Hz).

^{13}C NMR (75.5 MHz, DMSO-d^{6}) δ ppm: 167.34, 159.42 (d, $J_{F,C}$ = 238.6 Hz), 145.34, 145.30, 143.10, 142.85 (d, $J_{F,C}$ = 12.9 Hz), 133.94, 132.98, 130.25 (d, $J_{F,C}$ = 0.6 Hz), 129.89, 129.36, 128.53, 128.34, 126.74, 124.41, 124.05, 113.75 (d, $J_{F,C}$ = 10.08 Hz), 113.57, 112.72 (d, $J_{F,C}$ = 25.6 Hz), 111.70, 105.67 (d, $J_{F,C}$ = 24.5 Hz). (a carbon signal was not found).

^{19}F NMR (282 MHz, DMSO-d^{6}) δ ppm: -117.50 (s, 1F).

FT-IR (ATR, cm^{1}) = 1693.10 (C=O).

HRMS (ESI) *m/z* calcd. For $C_{23}H_{14}ClN_3O$ [M^{+}] 356.096106, [M^{+}+H] 357.103382, found [M^{+}+H] 357.1018.

(6-fluoro-2-(furan-2-yl)-1*H*-benzo[*d*]imidazol-1-yl)(naphthalen-1-yl)methanone (5f'):

Following the general procedure 3.

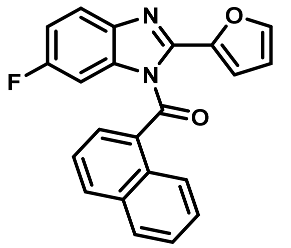

White solid. Yield = 34%. mp: 128. 6 – 129.6°C.

^{1}H NMR (300 MHz, DMSO-d^{6}) δ ppm: 8.37 (d, 1H, J_1 = 8.4 Hz), 8.17 (d, 1H, J_1 = 8.3 Hz), 8.06 (d, 1H, J_1 = 7.4 Hz), 7.87 (dd, 1H, J_1 = 8.9 Hz, J_2 = 4.9 Hz), 7.76-7.64 (m, 2H), 7.60 (dd, 1H, J_1 = 7.2 Hz, J_2 = 1.0 Hz), 7.46-7.41 (m, 1H), 7.31 (td, 1H, J_1 = 9.5 Hz, J_2 = 2.5 Hz), 7.25 (d, 1H, J_1 = 2.4 Hz), 7.22-7.21 (m, 1H), 6.90 (dd, 1H, J_1 = 3.5 Hz, J_2 = 0.5 Hz), 6.33 (dd, 1H J_1 = 3.5 Hz, J_2 = 1.7 Hz).

^{13}C NMR (75.5 MHz, DMSO-d^{6}) δ ppm: 167.64, 159.90 (d, $J_{F,C}$ = 240.3 Hz), 145.34, 144.72, 143.36, 138.99 (d, $J_{F,C}$ = 0.9 Hz), 134.18, 134.00, 133.21, 130.14, 130.08, 129.56, 128.80, 128.61, 127.02, 124.65, 124.34, 121.23 (d, $J_{F,C}$ = 10.2 Hz), 113.36, 112.90 (d, $J_{F,C}$ = 24.8 Hz), 111.87, 100.27 (d, $J_{F,C}$ = 29.2 Hz).

^{19}F NMR (282 MHz, DMSO-d^{6}) δ ppm: -115.50 (s, 1F).

FT-IR (ATR, cm^{1}) = 1707.49 (C=O).

HRMS (ESI) *m/z* calcd. For $C_{23}H_{14}ClN_3O$ [M^{+}] 356.096106, [M^{+}+H] 357.103382, found [M^{+}+H] 357.1059.

NMR spectra of compounds

5(6)-chloro-2-(pyridin-3-yl)-1*H*-benzo[*d*]imidazole (3a)

^{1}H NMR (300 MHz, DMSO-d^{6})

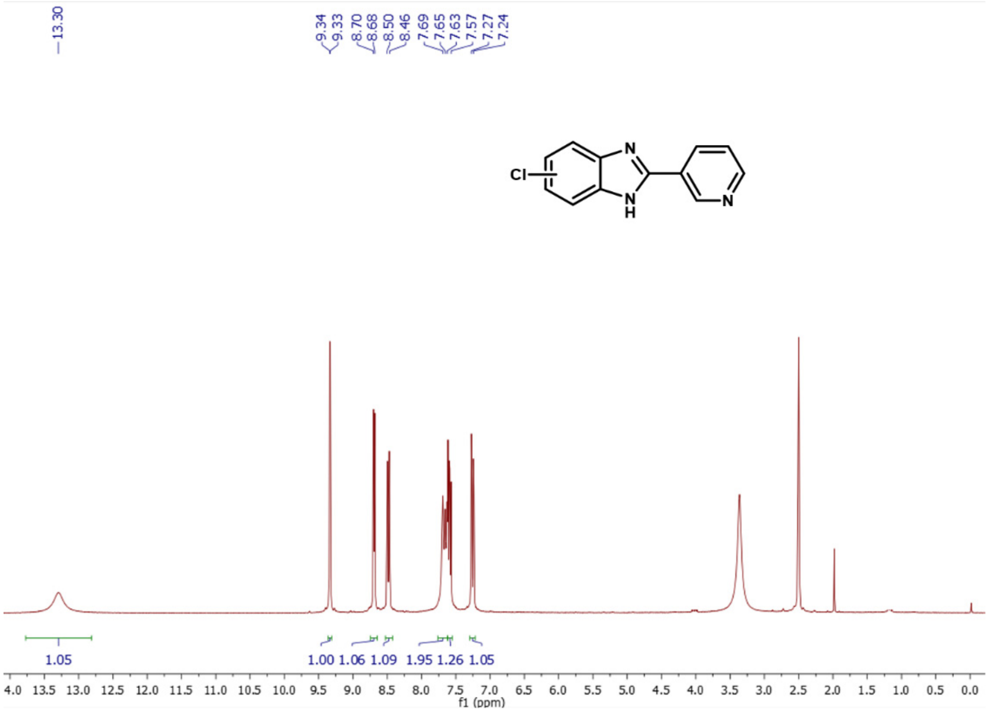

^{13}C NMR (75.5 MHz, DMSO-d^{6})

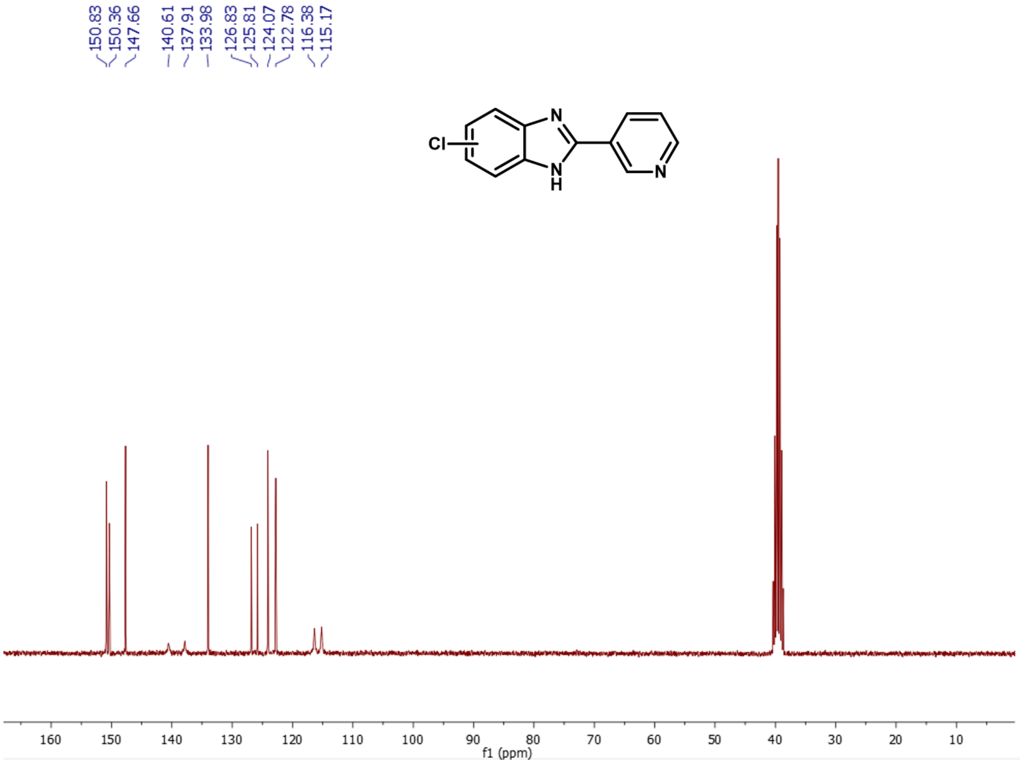

5(6)-chloro-2-(3-methoxyphenyl)-1*H*-benzo[*d*]imidazole (**3b**)

^{1}H NMR (300 MHz, DMSO-d^6)

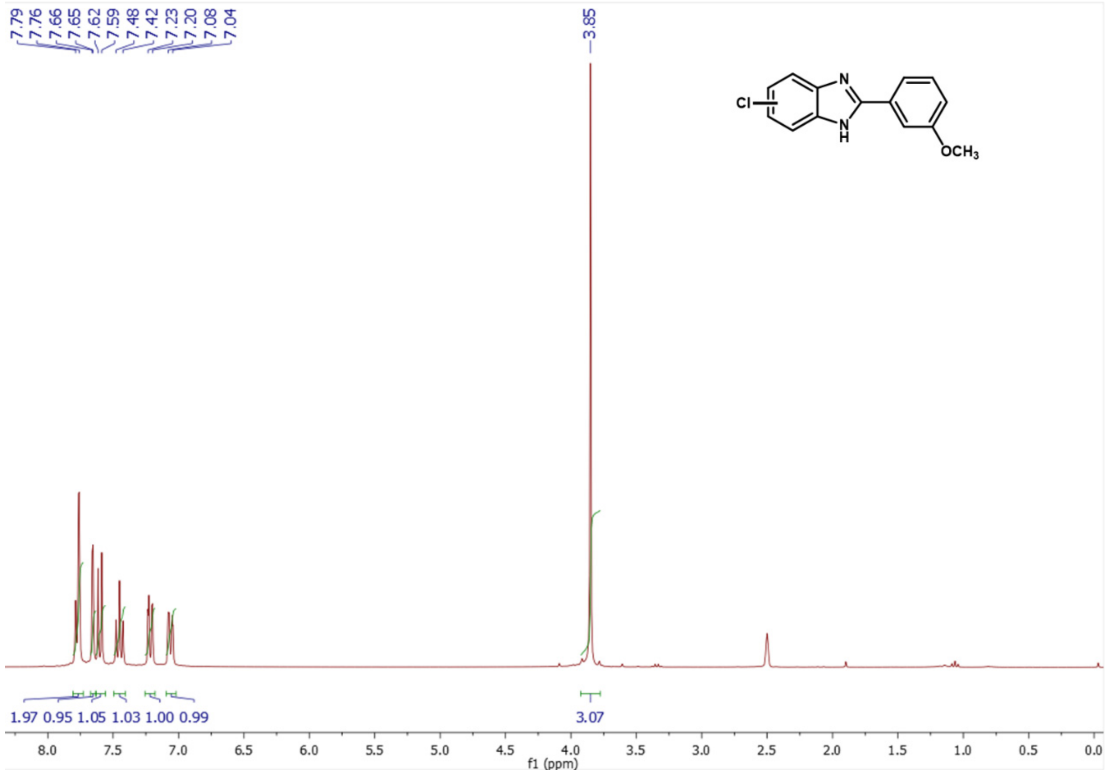

^{13}C NMR (75.5 MHz, DMSO-d^6)

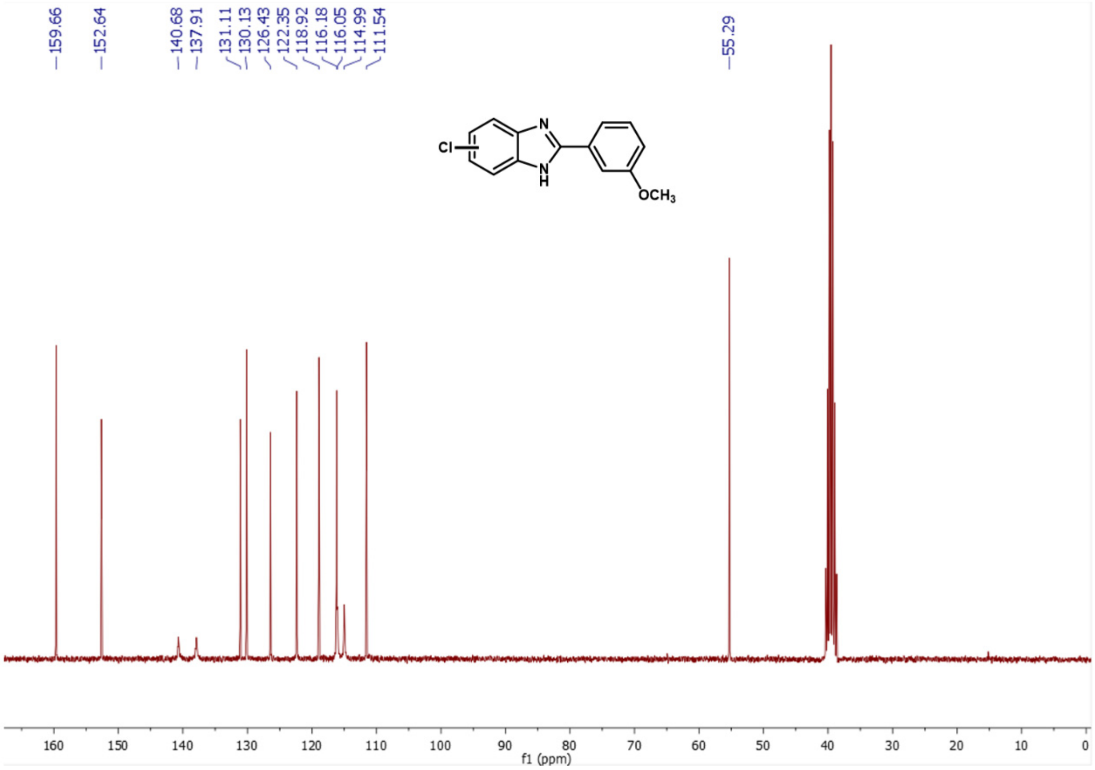

3-(5(6)-chloro-1*H*-benzo[*d*]imidazol-2-yl)-5-methylisoxazole (**3c**)

^{1}H NMR (300 MHz, DMSO-d^{6})

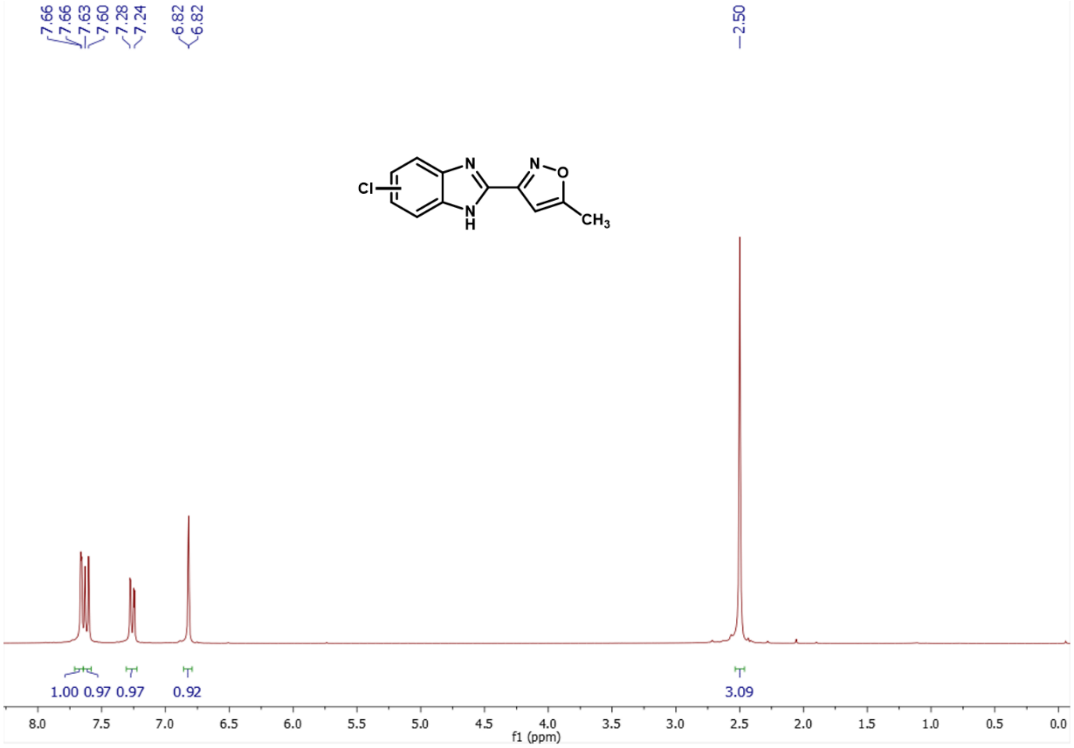

^{13}C NMR (75.5 MHz, DMSO-d^{6})

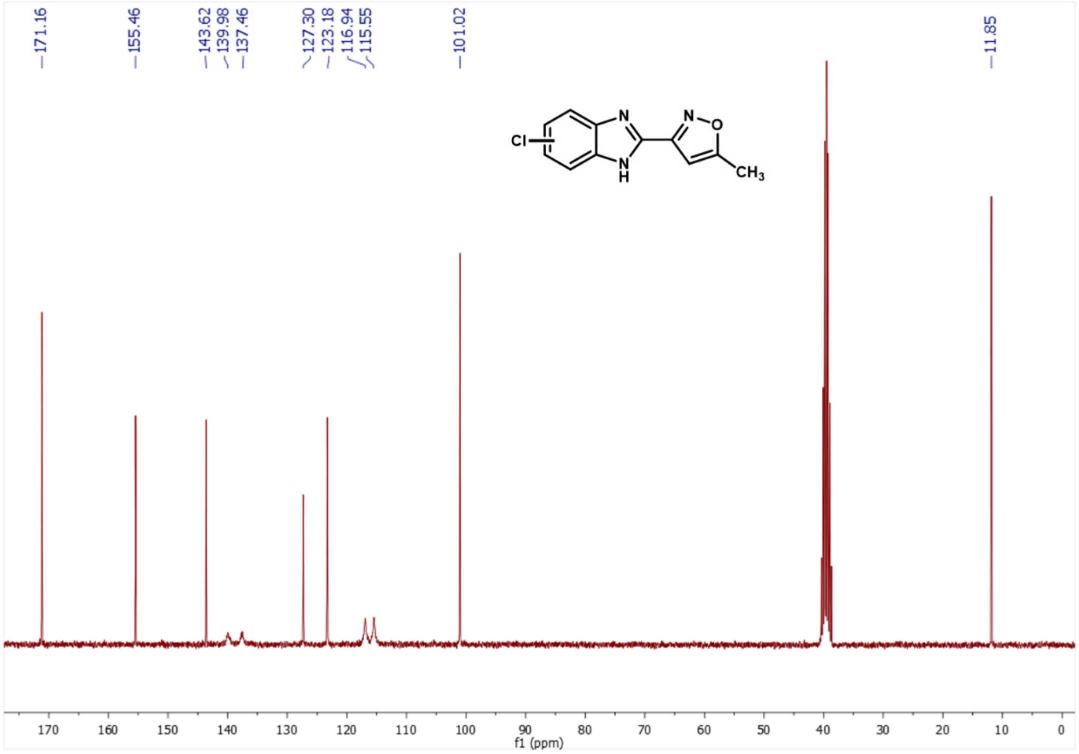

3-(5(6)-chloro-1*H*-benzo[*d*]imidazol-2-yl)isoxazole (**3d**):

^{1}H NMR (300 MHz, DMSO-d^{6})

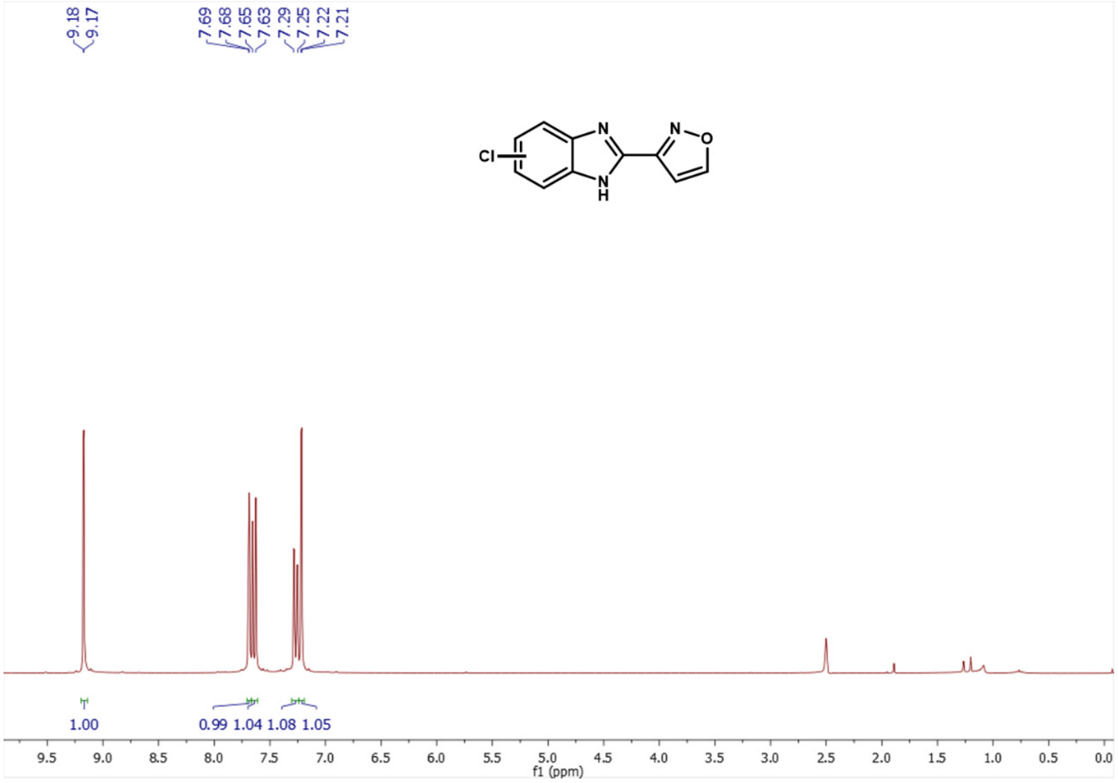

^{13}C NMR (75.5 MHz, DMSO-d^{6})

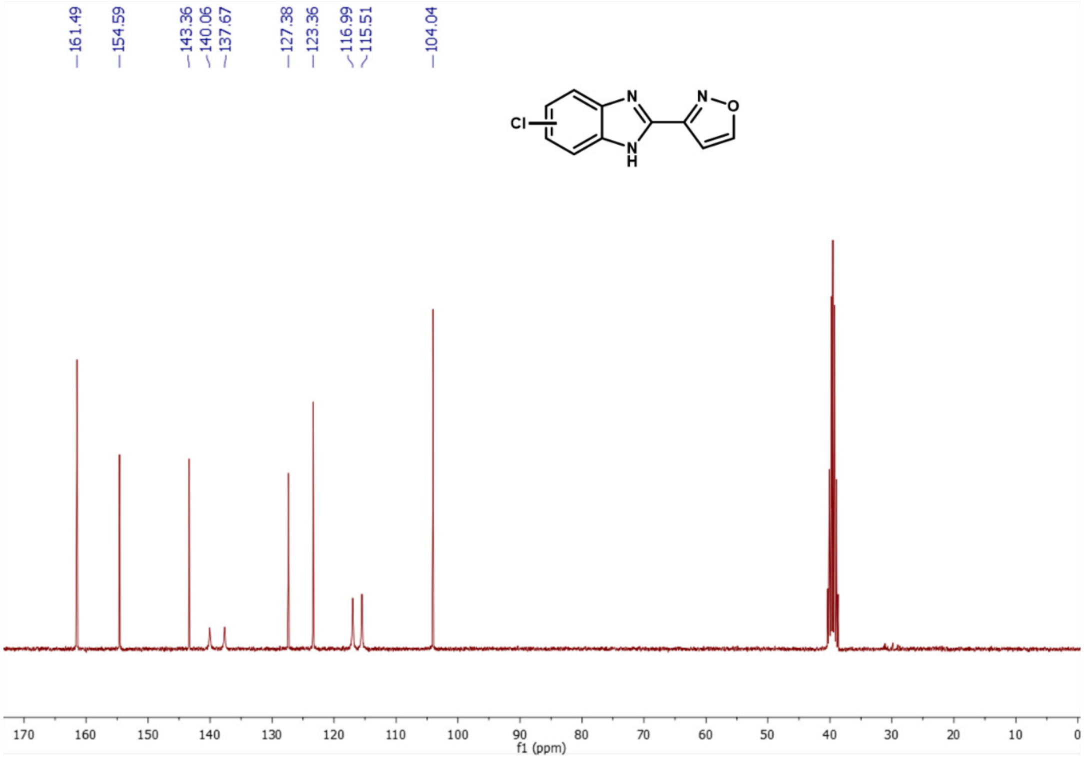

5(6)-chloro-2-(5-methylfuran-2-yl)-1*H*-benzo[*d*]imidazole (**3e**)

^{1}H NMR (300 MHz, DMSO-d^{6})

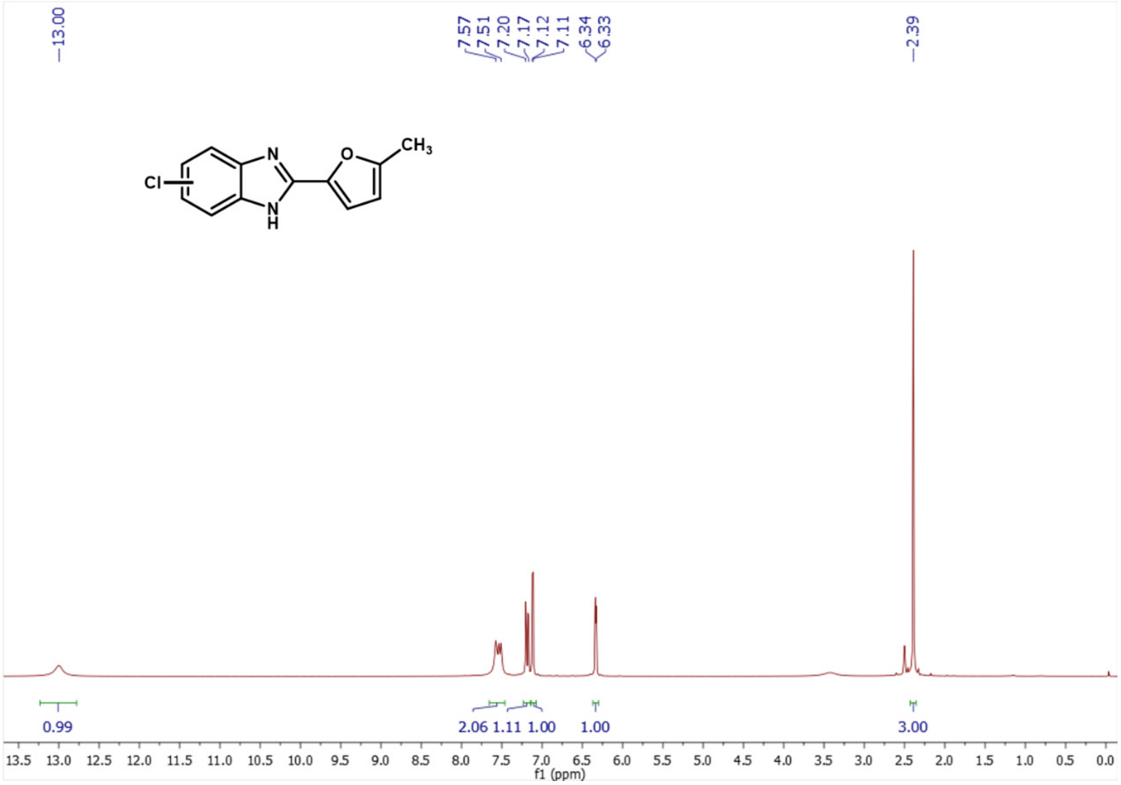

^{13}C NMR (75.5 MHz, DMSO-d^{6})

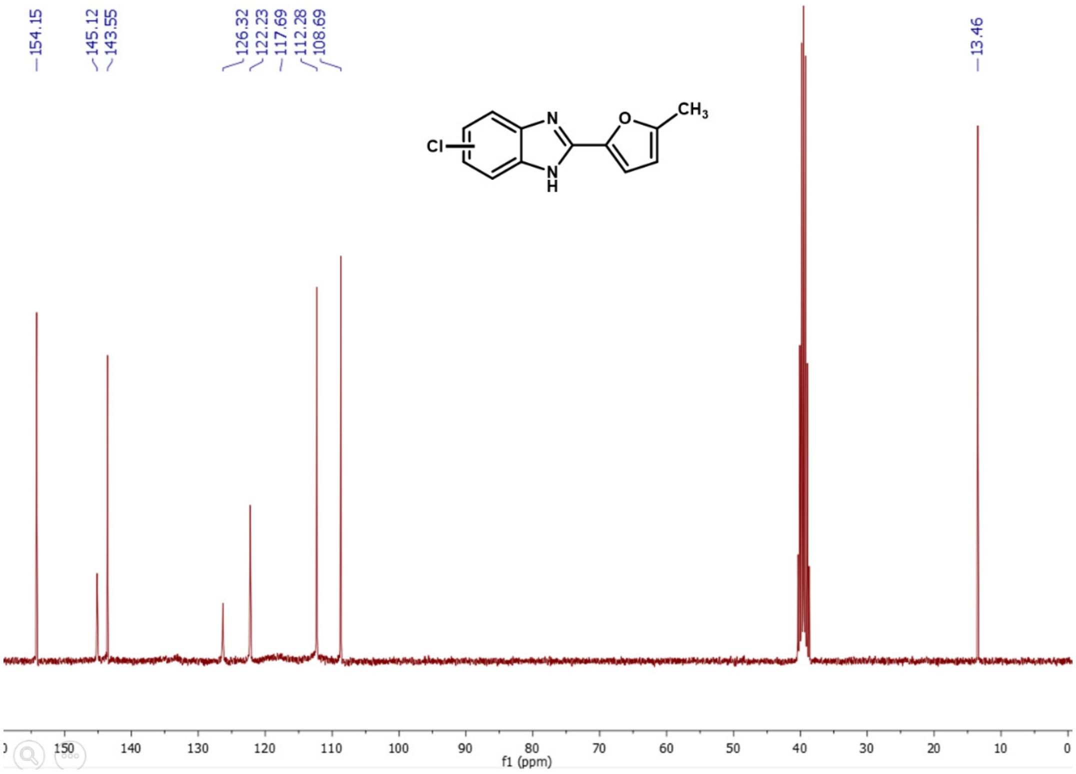

5(6)-chloro-2-(furan-2-yl)-1*H*-benzo[*d*]imidazole (**3f**)

^{1}H NMR (300 MHz, DMSO-d^{6})

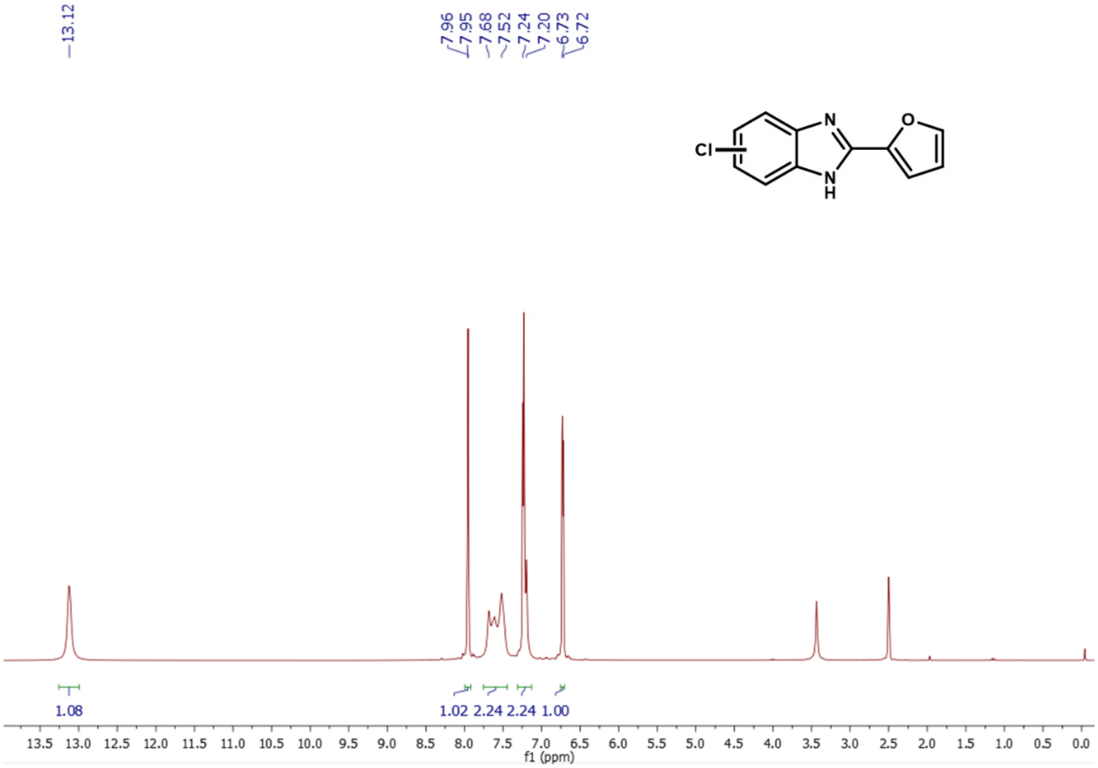

^{13}C NMR (75.5 MHz, DMSO-d^{6})

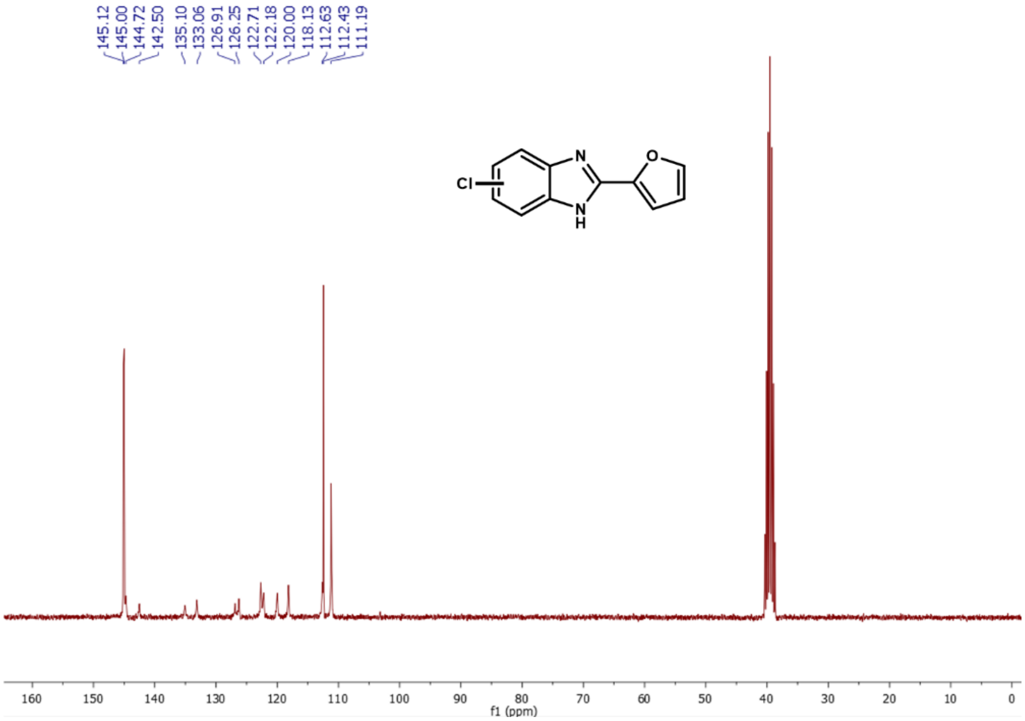

5(6)-fluoro-2-(pyridin-3-yl)-1*H*-benzo[*d*]imidazole (**3a'**):

^{1}H NMR (300 MHz, DMSO-d^{6})

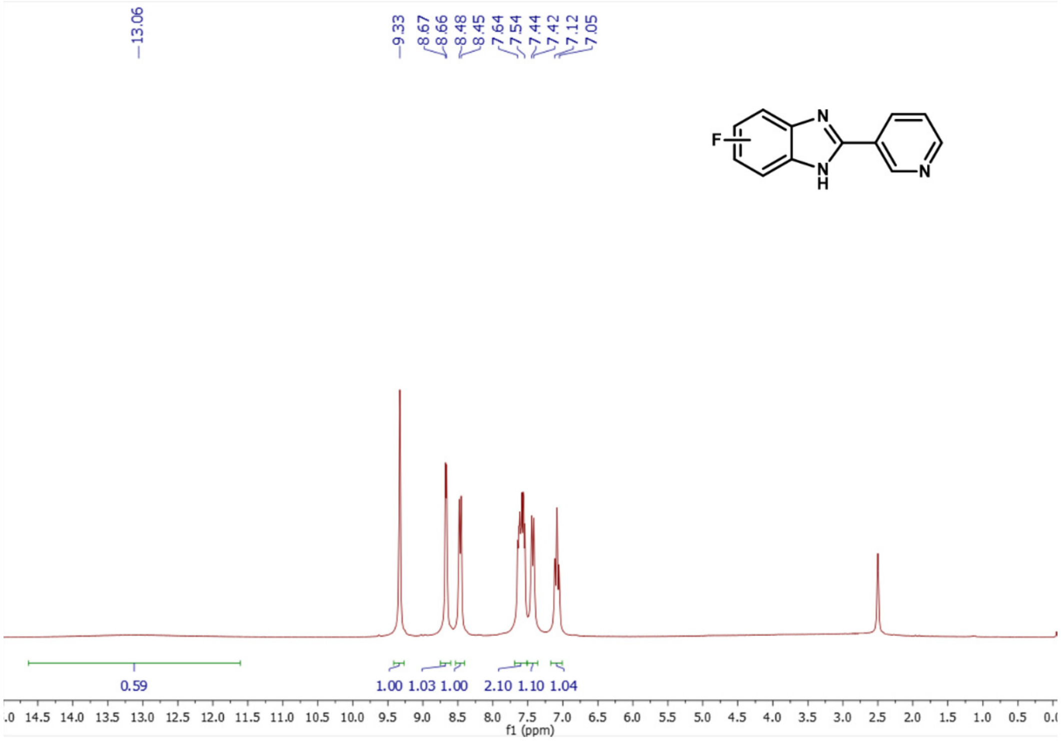

^{13}C NMR (75.5 MHz, DMSO-d^{6})

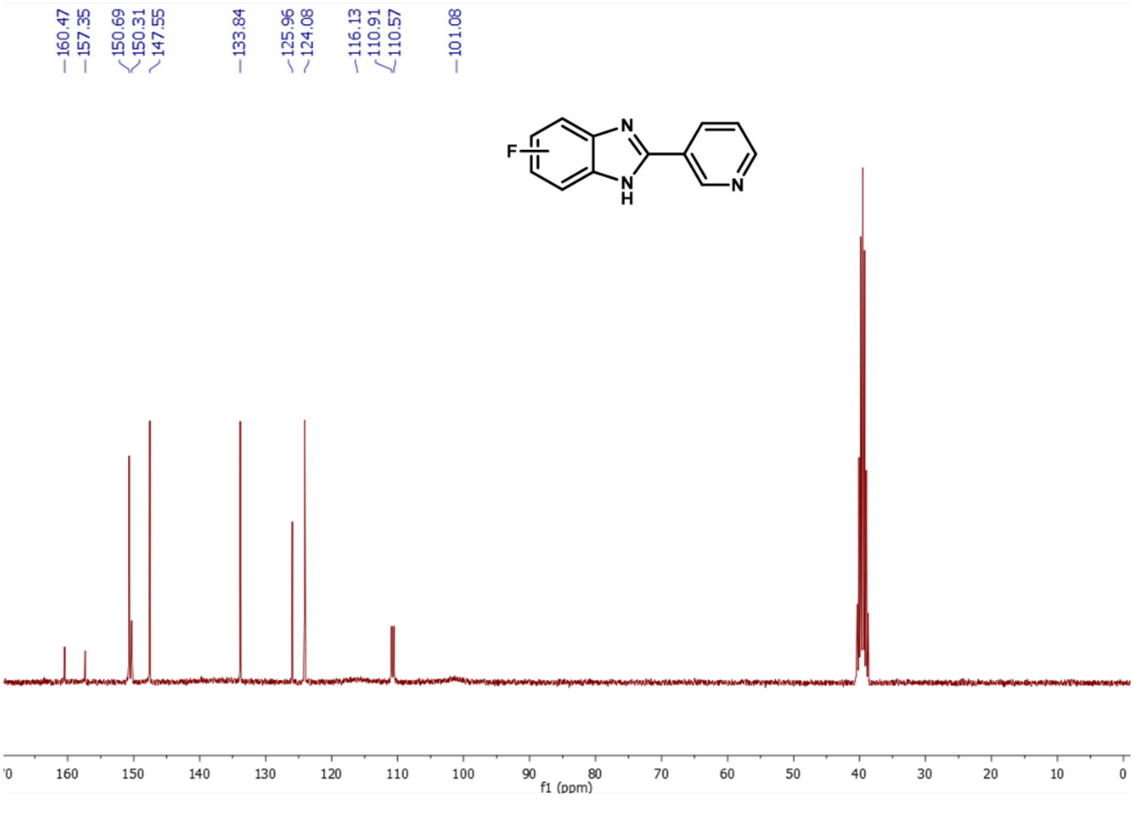

5(6)-fluoro-2-(3-methoxyphenyl)-1*H*-benzo[*d*]imidazole (**3b'**)

^{1}H NMR (300 MHz, DMSO-d^6)

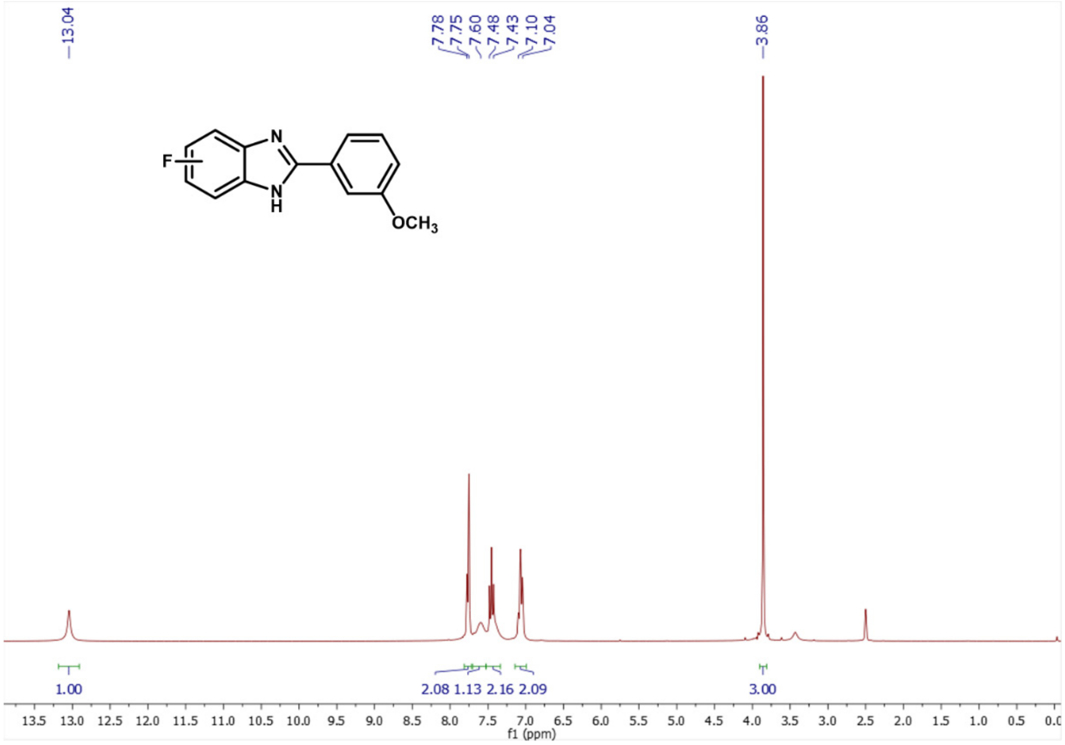

^{13}C NMR (75.5 MHz, DMSO-d^6)

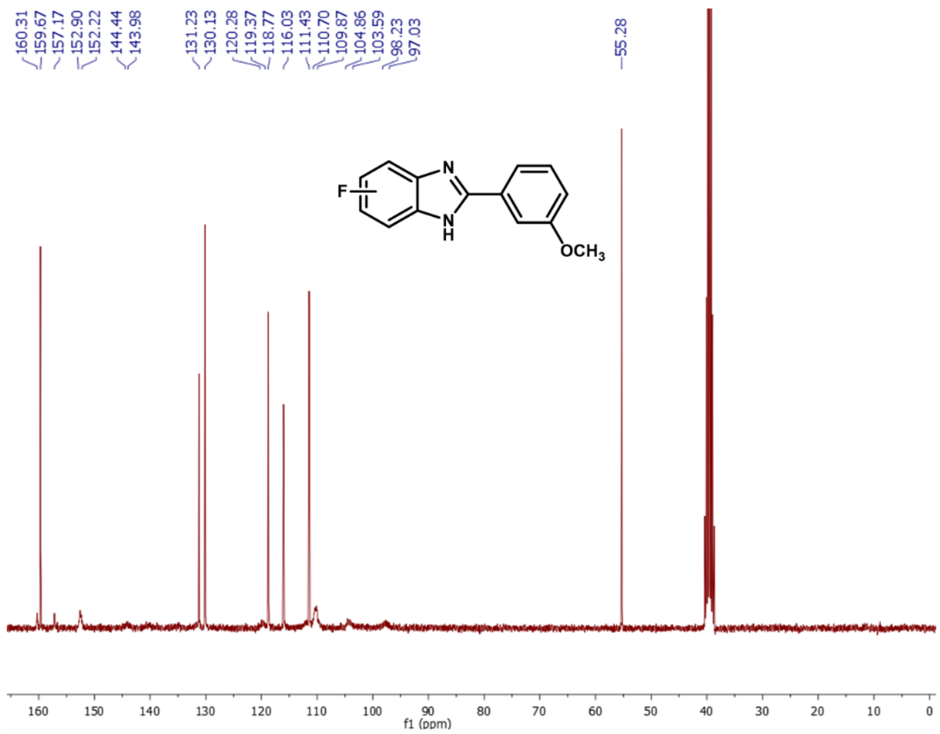

3-(5(6)-fluoro-1*H*-benzo[*d*]imidazol-2-yl)-5-methylisoxazole (**3c'**)

^{1}H NMR (300 MHz, DMSO-d^{6})

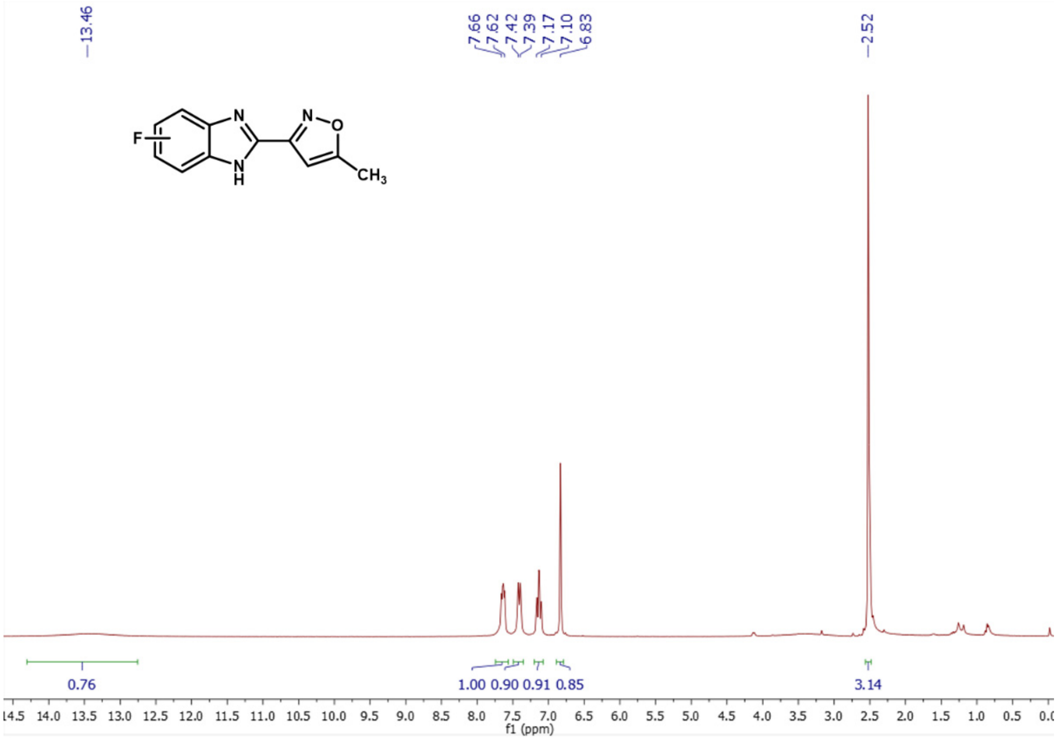

^{13}C NMR (75.5 MHz, DMSO-d^{6})

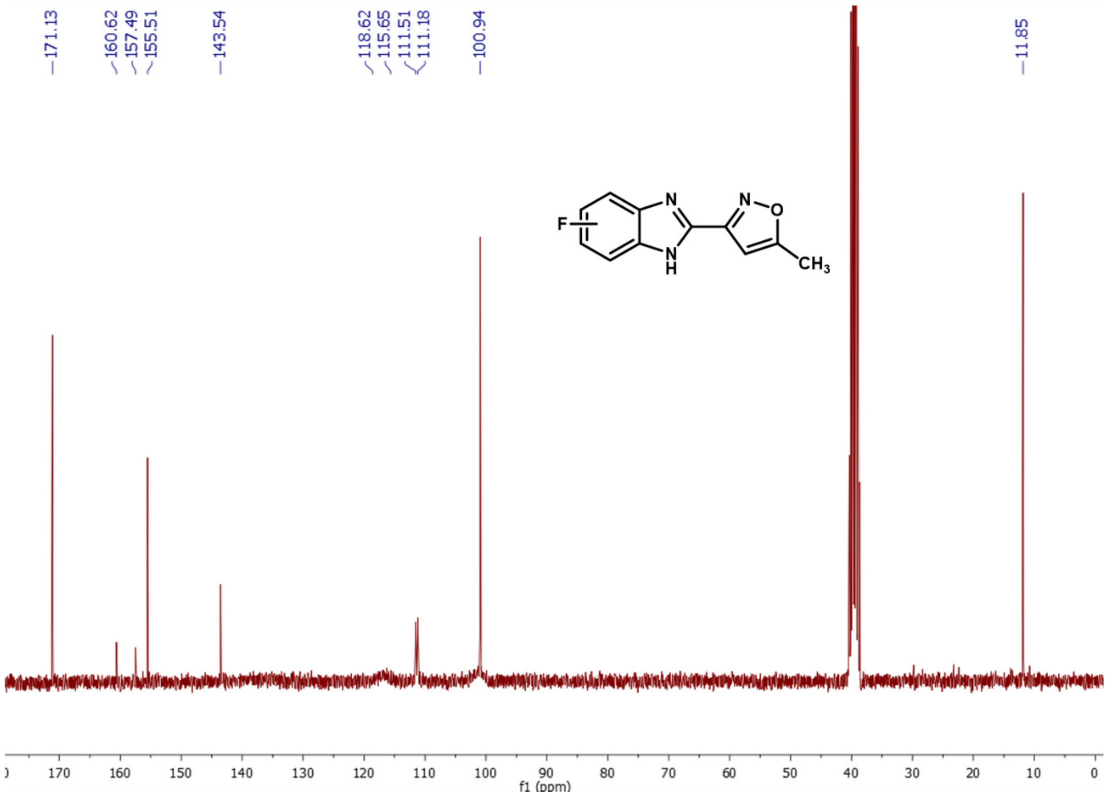

3-(5(6)-fluoro-1*H*-benzo[*d*]imidazol-2-yl)isoxazole (**3d’**):

^{1}H NMR (300 MHz, DMSO-d^{6})

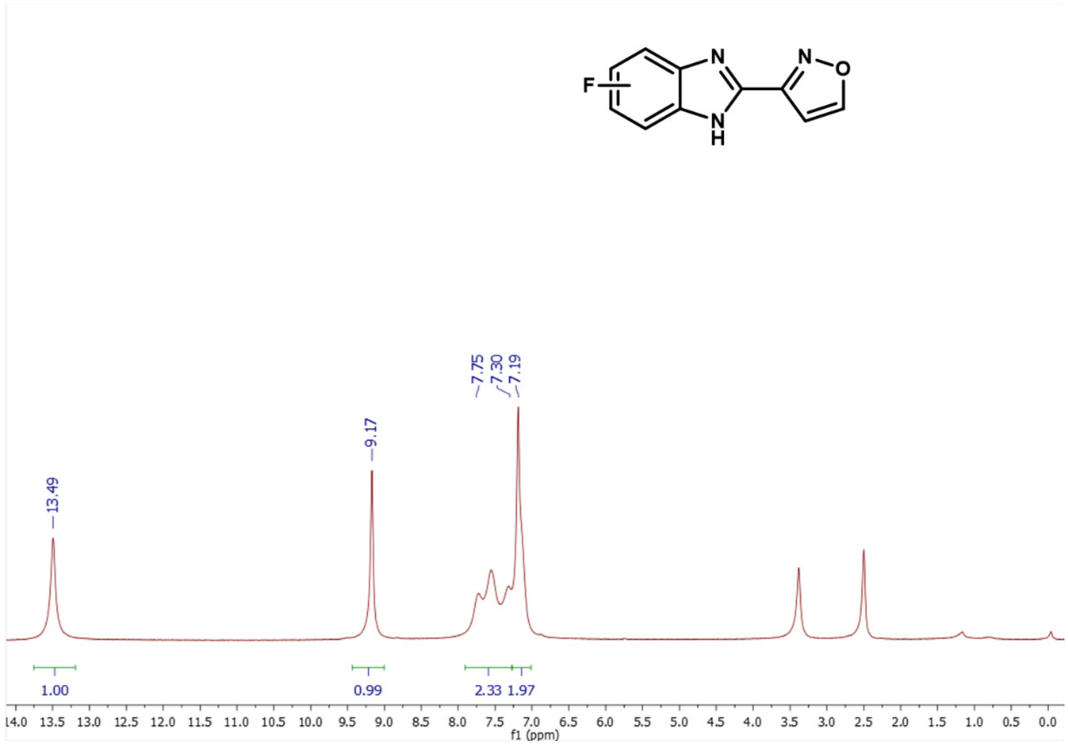

^{13}C NMR (75.5 MHz, DMSO-d^{6})

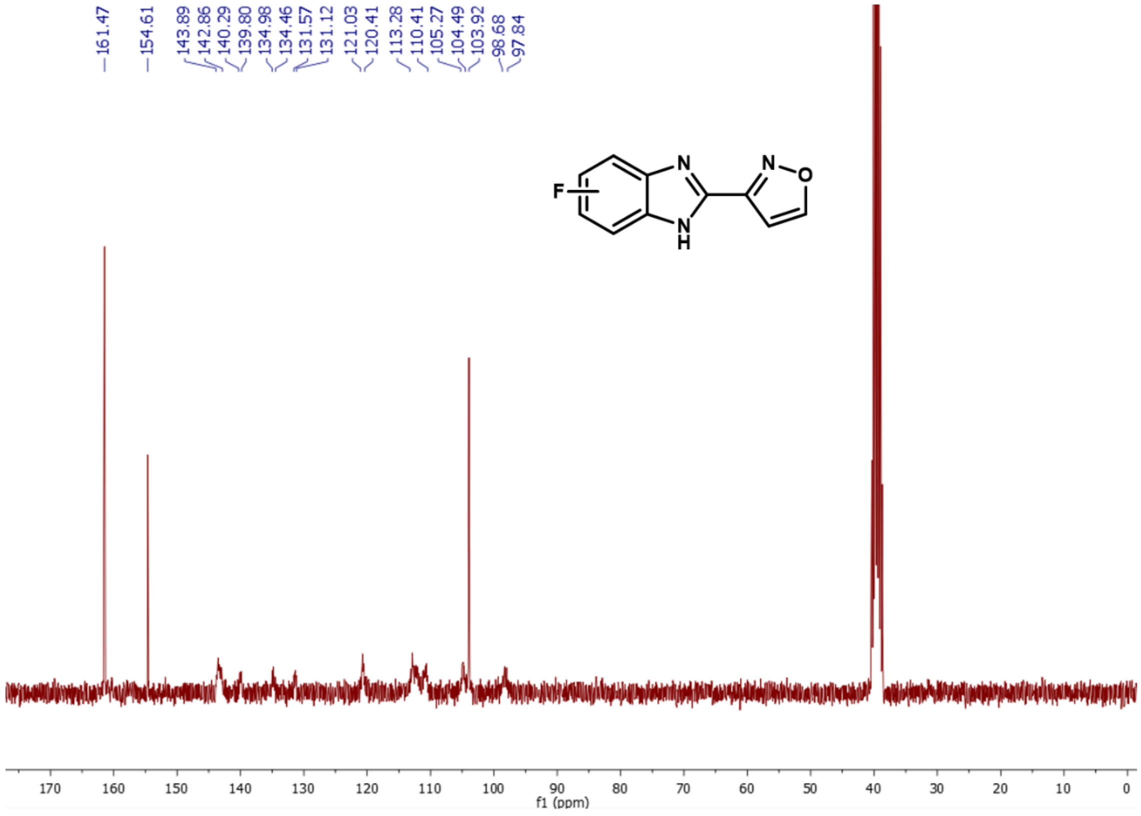

5(6)-fluoro-2-(5-methylfuran-2-yl)-1*H*-benzo[*d*]imidazole (**3e'**)

^{1}**H NMR** (300 MHz, DMSO-d^{6})

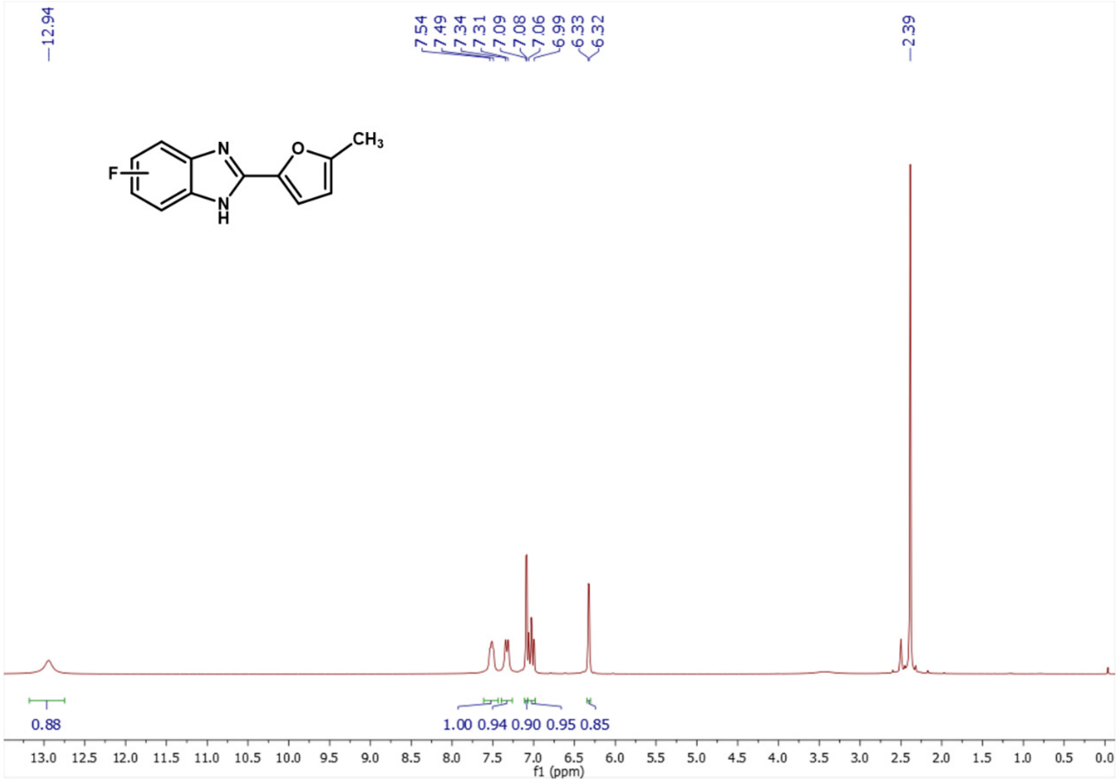

^{13}**C NMR** (75.5 MHz, DMSO-d^{6})

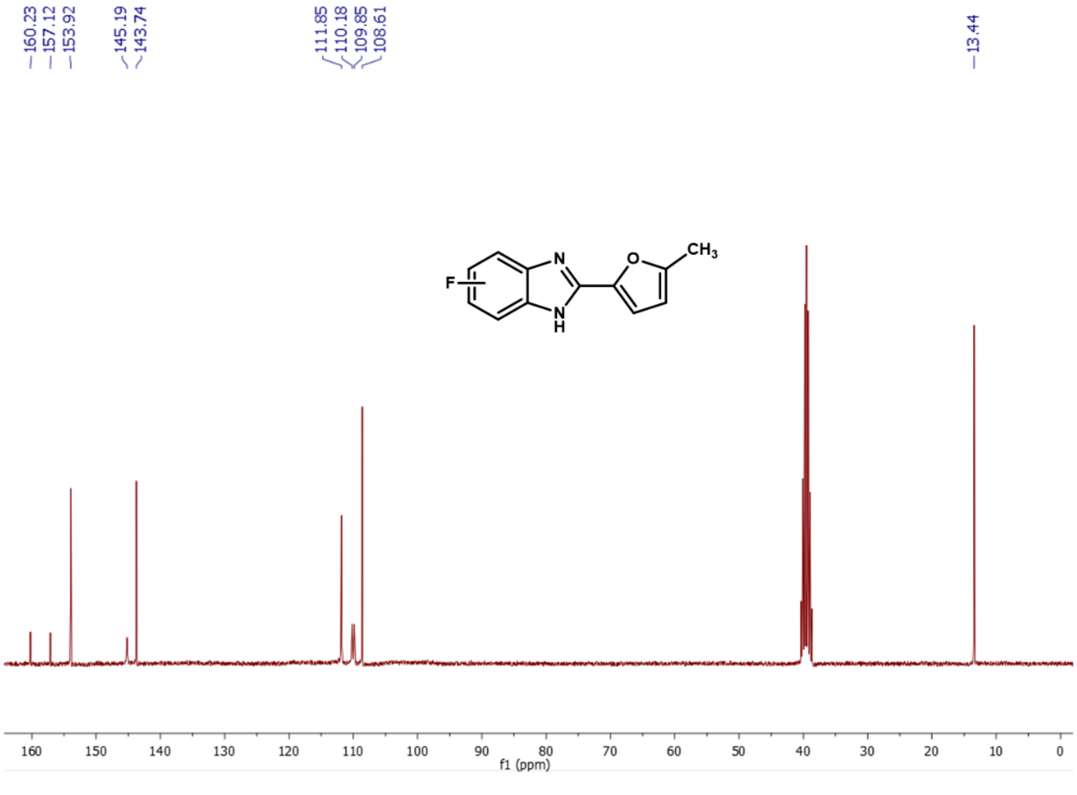

5(6)-fluoro-2-(furan-2-yl)-1*H*-benzo[*d*]imidazole (**3f’**)

^{1}H NMR (300 MHz, DMSO-d^6)

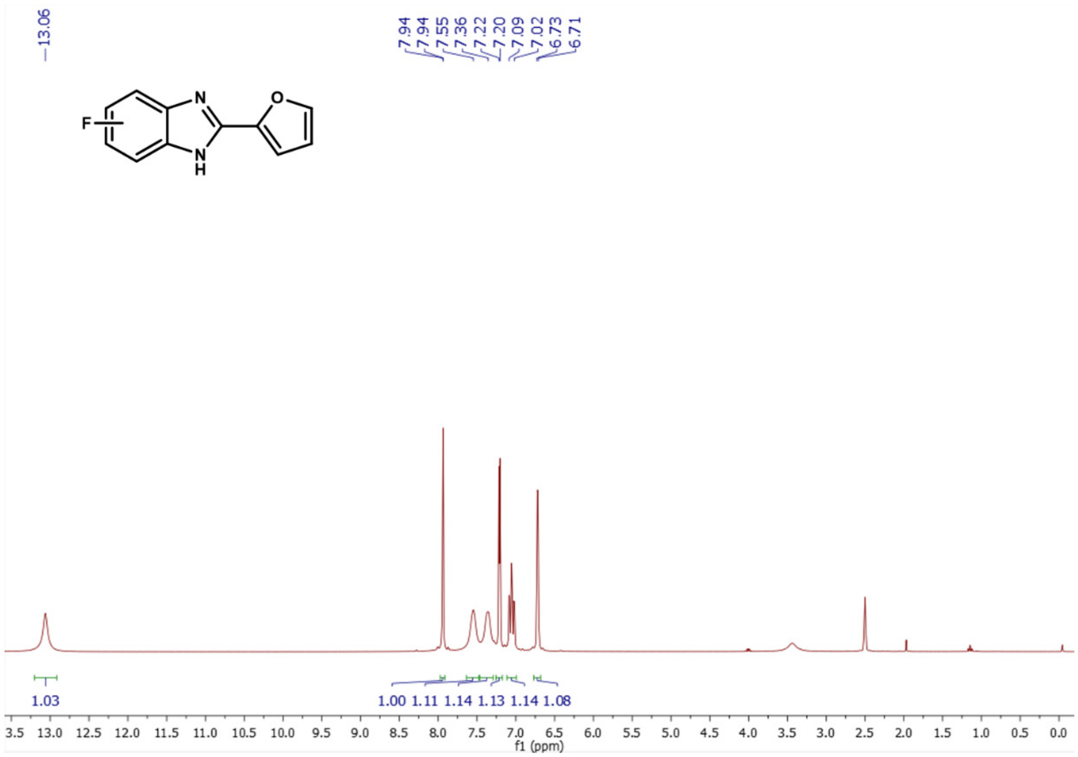

^{13}C NMR (75.5 MHz, DMSO-d^6)

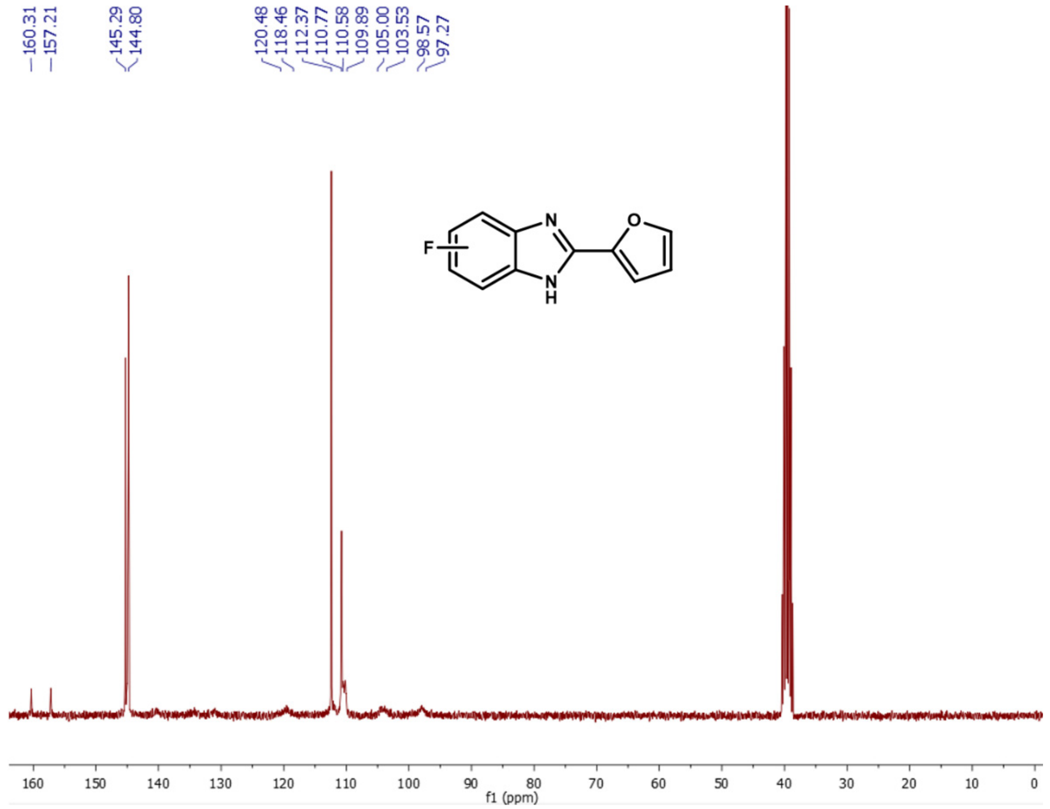

(5-chloro-2-(pyridin-3-yl)-1*H*-benzo[*d*]imidazol-1-yl)(naphthalen-1-yl)methanone (**4a**)

^{1}H NMR (300 MHz, DMSO-d^{6})

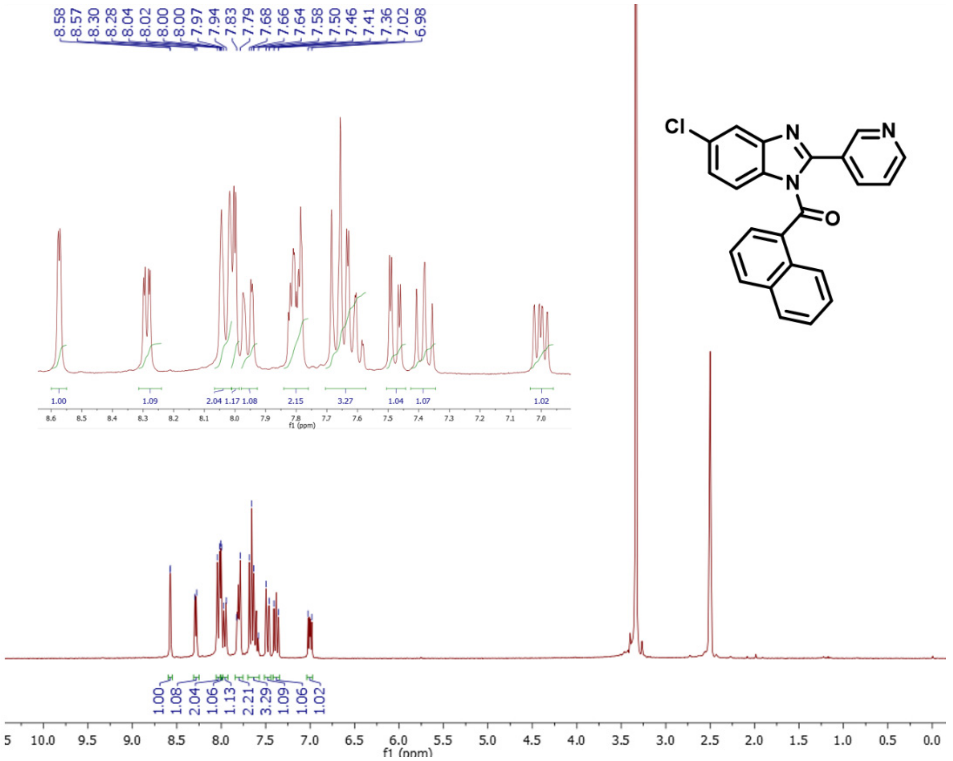

^{13}C NMR (75.5 MHz, DMSO-d^{6})

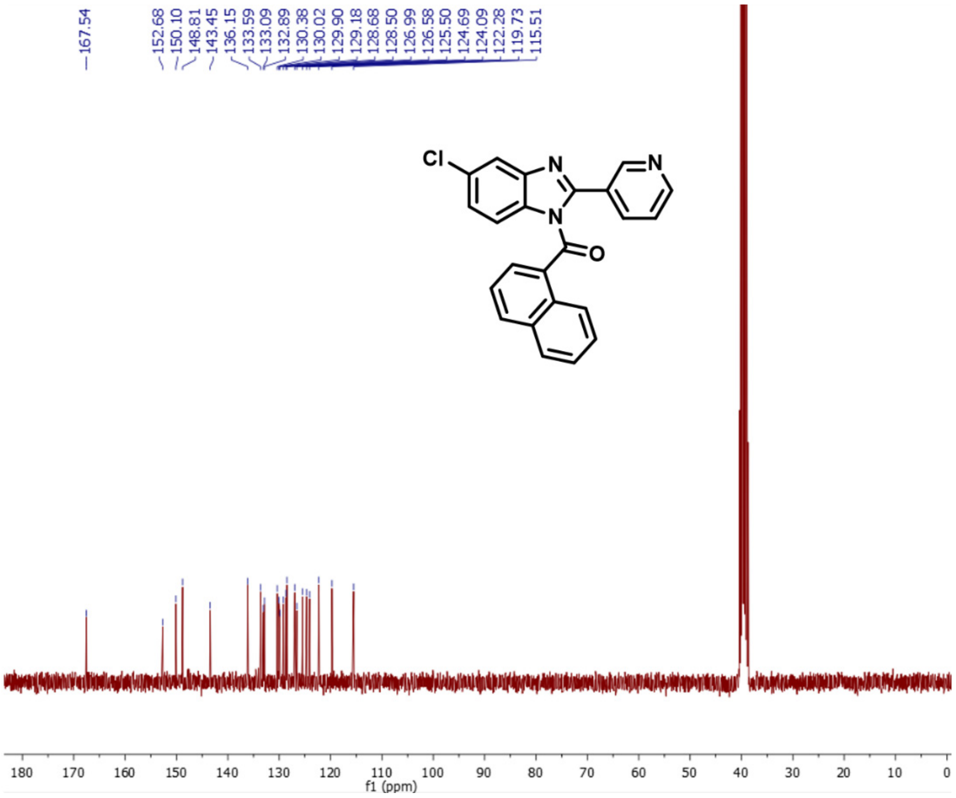

(6-chloro-2-(pyridin-3-yl)-1H-benzo[*d*]imidazol-1-yl)(naphthalen-1-yl)methanone (**4a'**)

^{1}H NMR (300 MHz, DMSO-d^6)

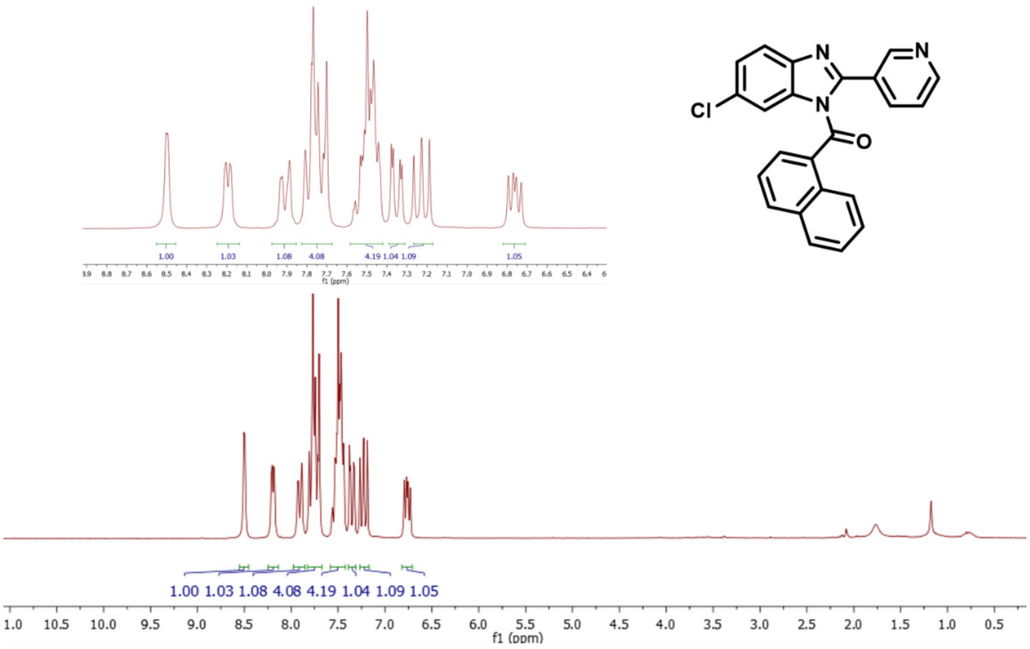

^{13}C NMR (75.5 MHz, DMSO-d^6)

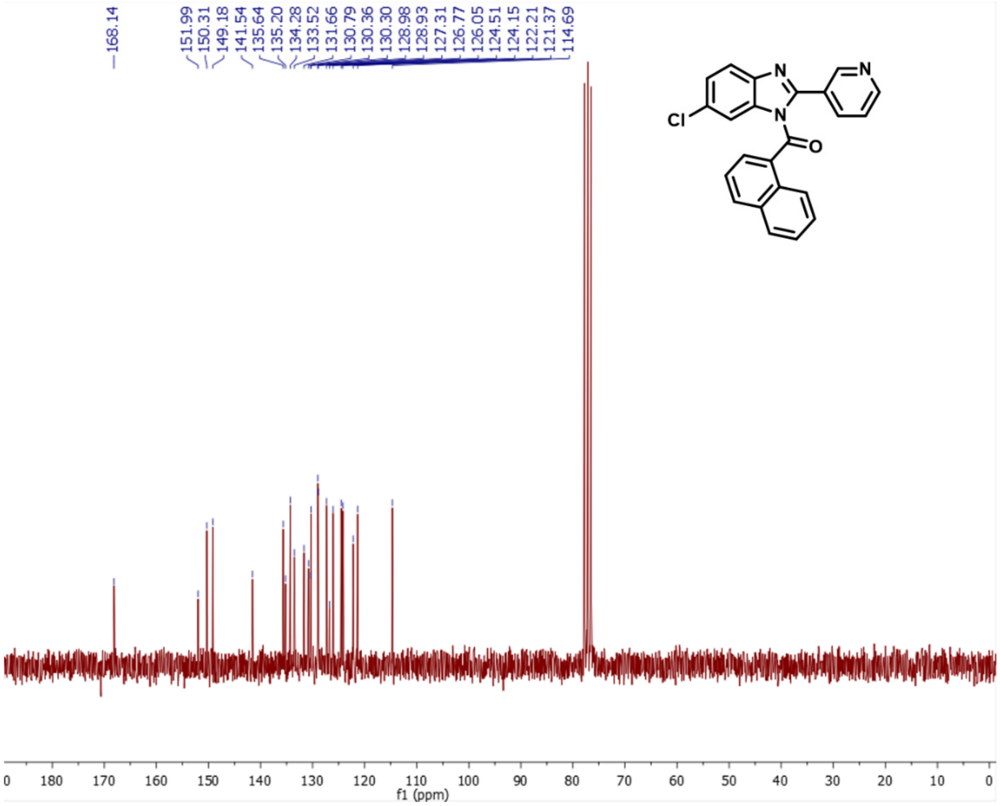

(5-chloro-2-(3-methoxyphenyl)-1*H*-benzo[*d*]imidazol-1-yl)(naphthalen-1-yl)methanone (**4b**)

^{1}H NMR (300 MHz, DMSO-d^{6})

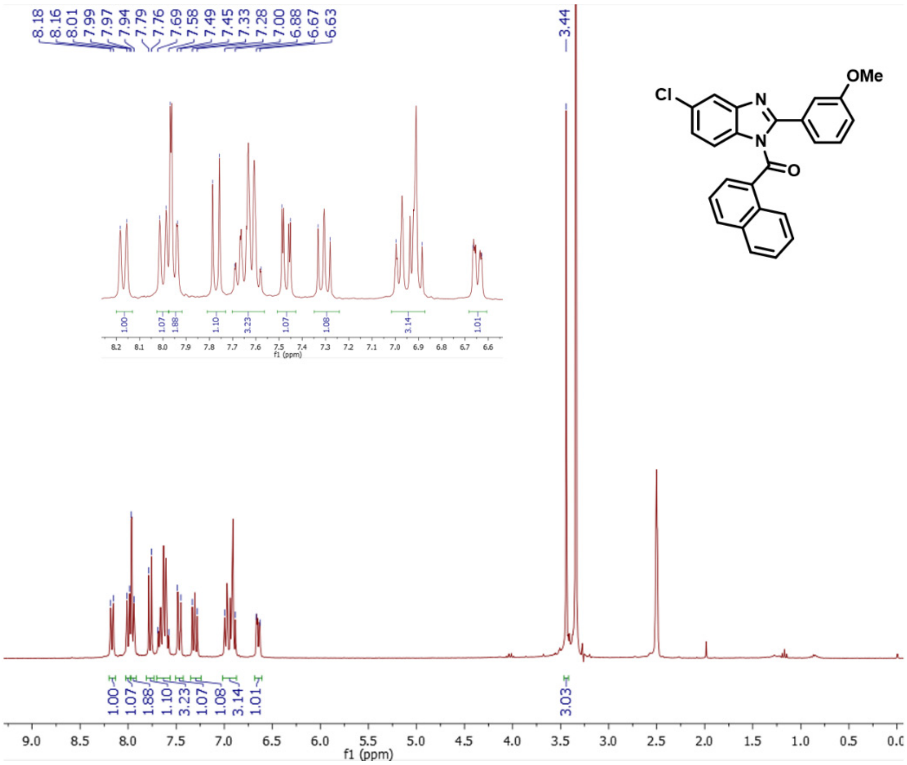

^{13}C NMR (75.5 MHz, DMSO-d^{6})

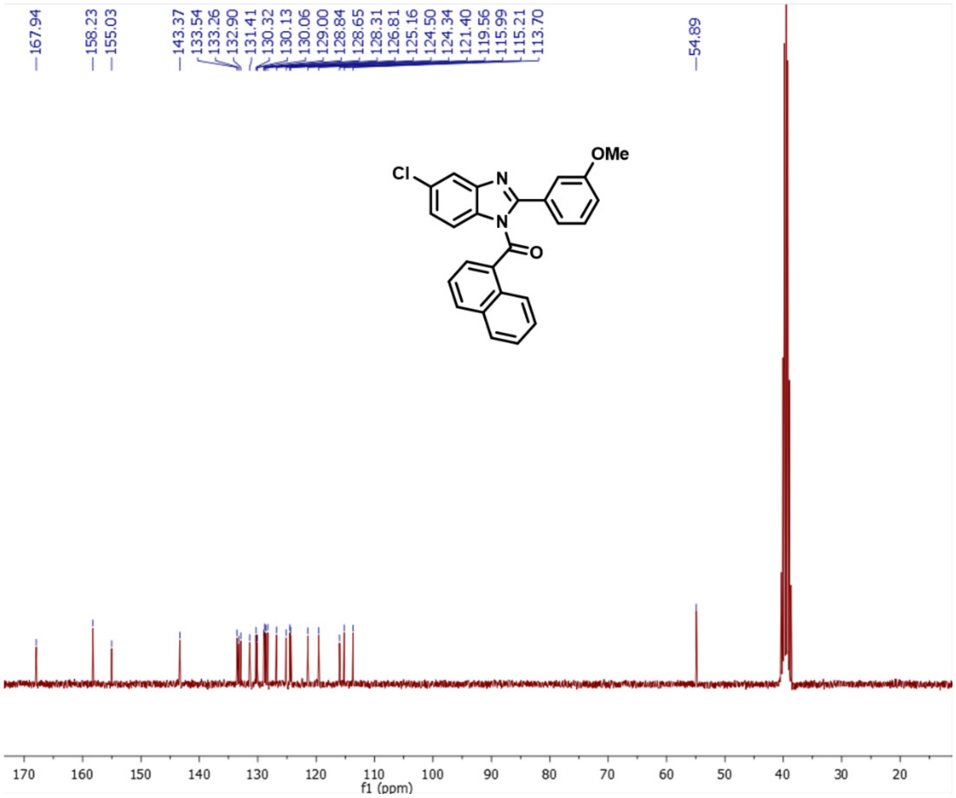

(6-chloro-2-(3-methoxyphenyl)-1*H*-benzo[*d*]imidazol-1-yl)(naphthalen-1-yl)methanone (**4b’**)

^{1}H NMR (300 MHz, DMSO-d^6)

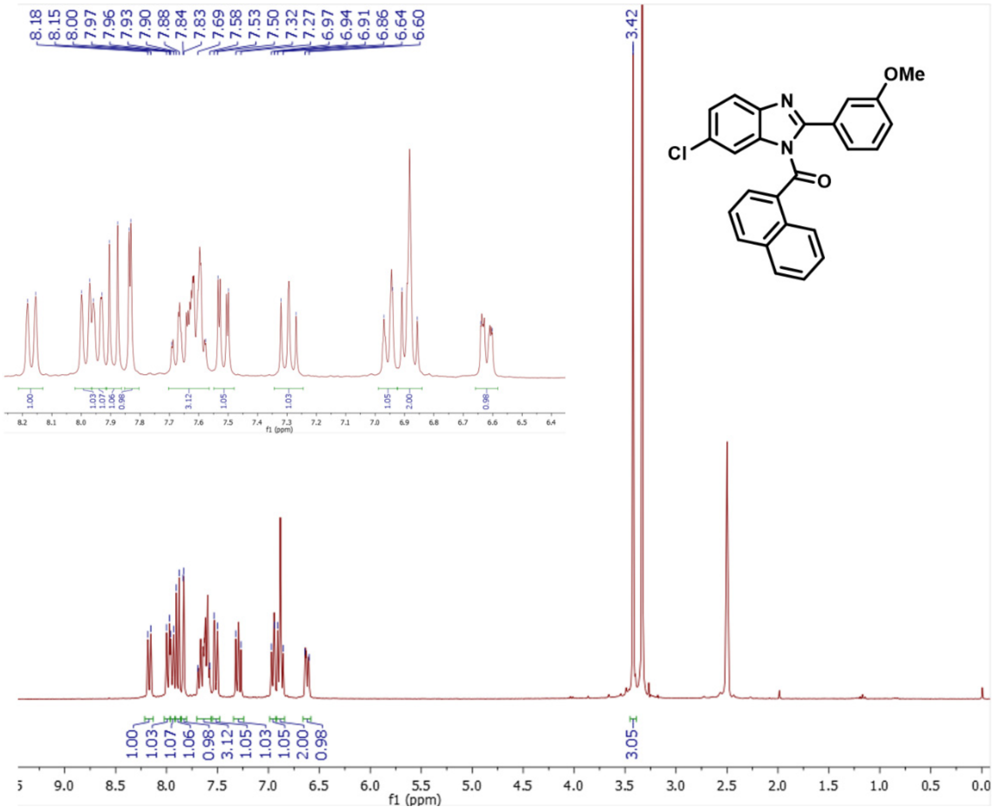

^{13}C NMR (75.5 MHz, DMSO-d^6)

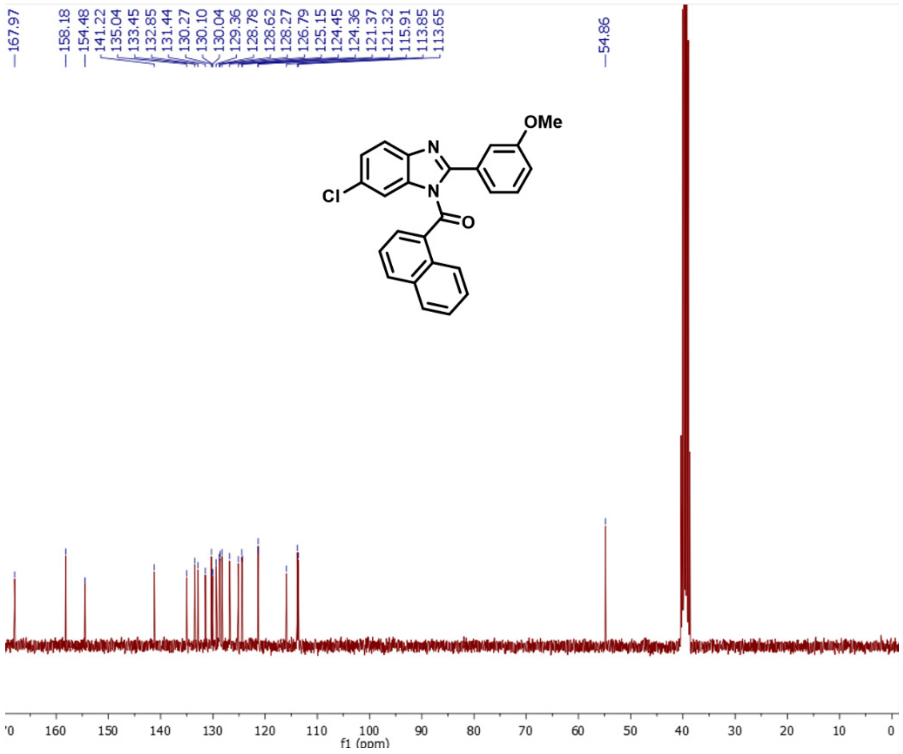

(5-chloro-2-(5-methylisoxazol-3-yl)-1*H*-benzo[*d*]imidazol-1-yl)(naphthalen-1-yl)methanone (**4c**)

^{1}H NMR (300 MHz, DMSO-d[6])

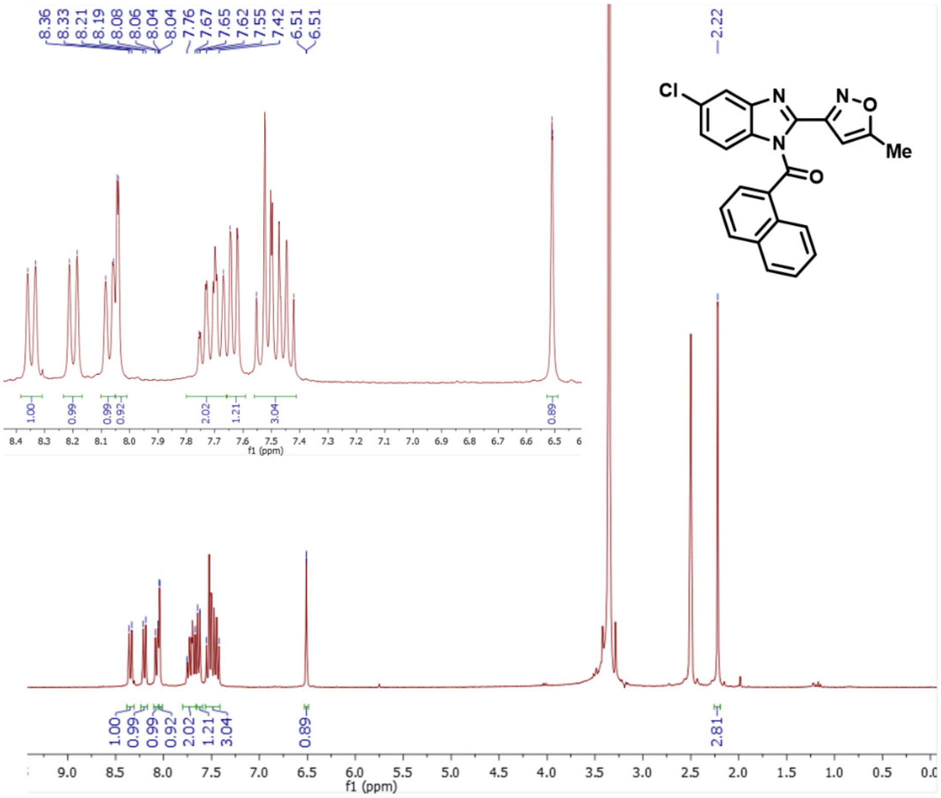

^{13}C NMR (75.5 MHz, DMSO-d[6])

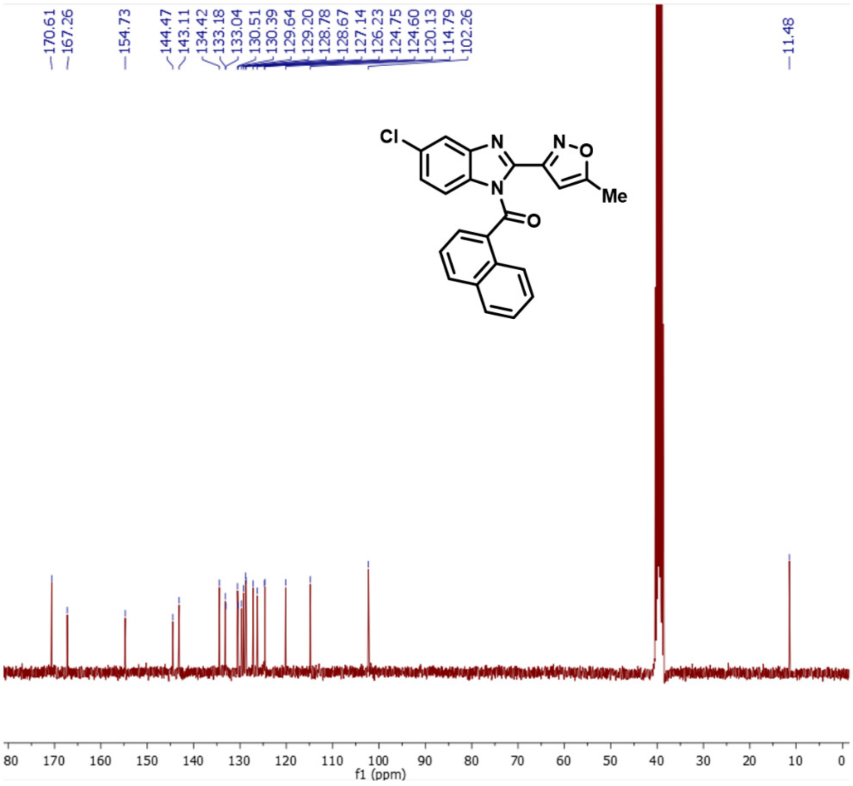

(6-chloro-2-(5-methylisoxazol-3-yl)-1*H*-benzo[*d*]imidazol-1-yl)(naphthalen-1-yl)methanone (**4c'**)

^{1}H NMR (300 MHz, DMSO-d^{6})

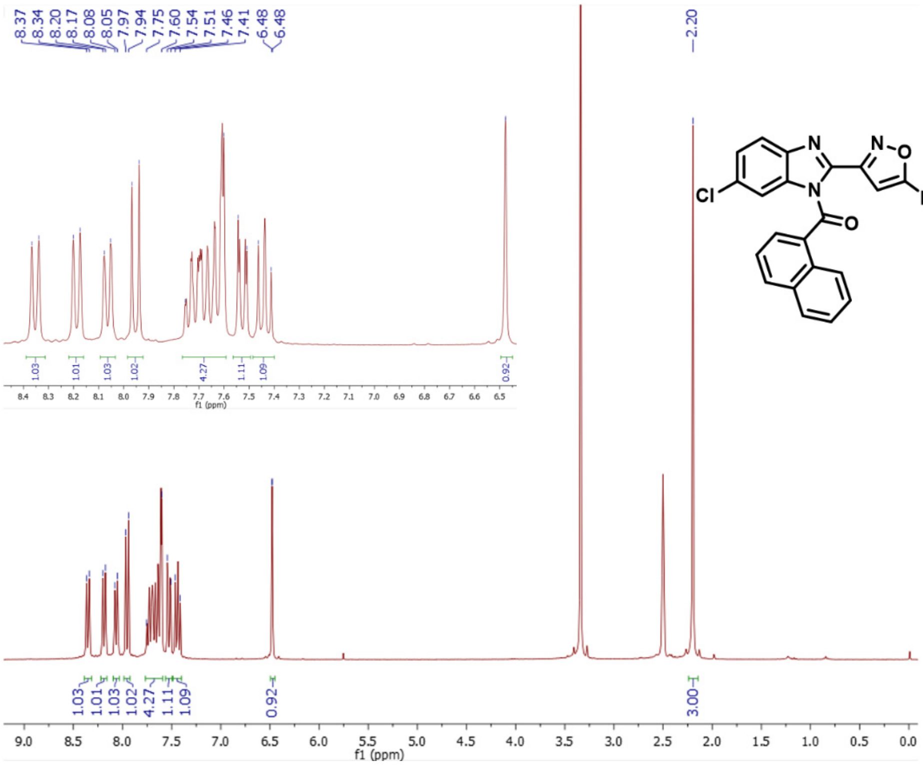

^{13}C NMR (75.5 MHz, DMSO-d^{6})

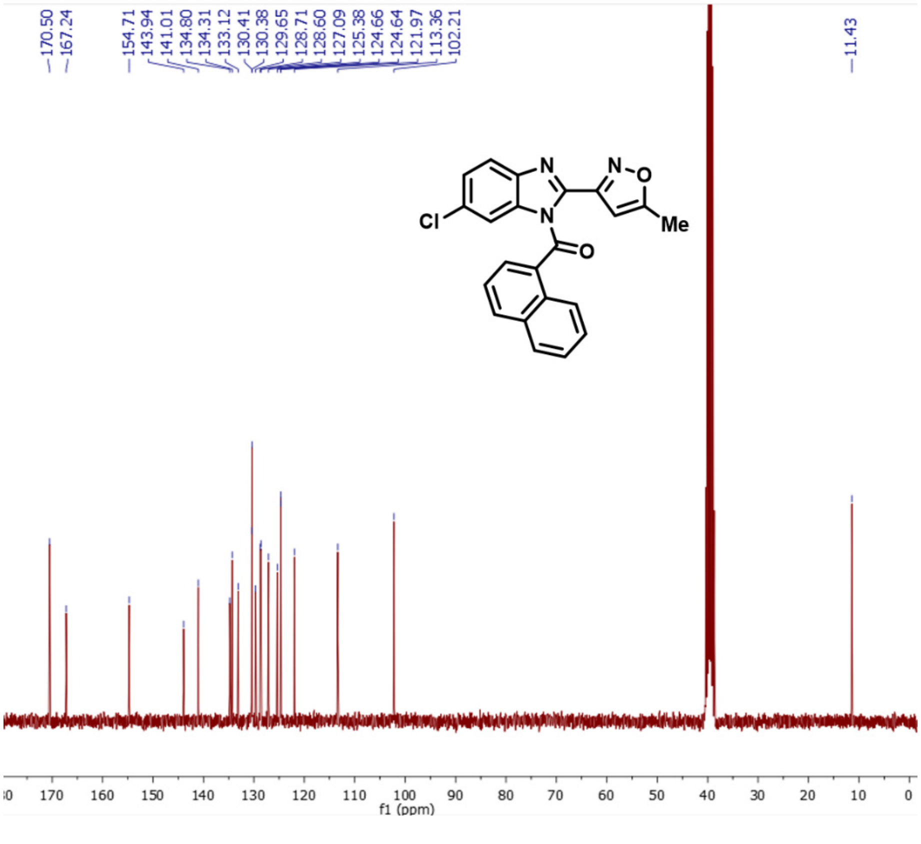

(5-chloro-2-(isoxazol-3-yl)-1*H*-benzo[*d*]imidazol-1-yl)(naphthalen-1-yl)methanone (**4d**)

^{1}H NMR (300 MHz, DMSO-d^{6})

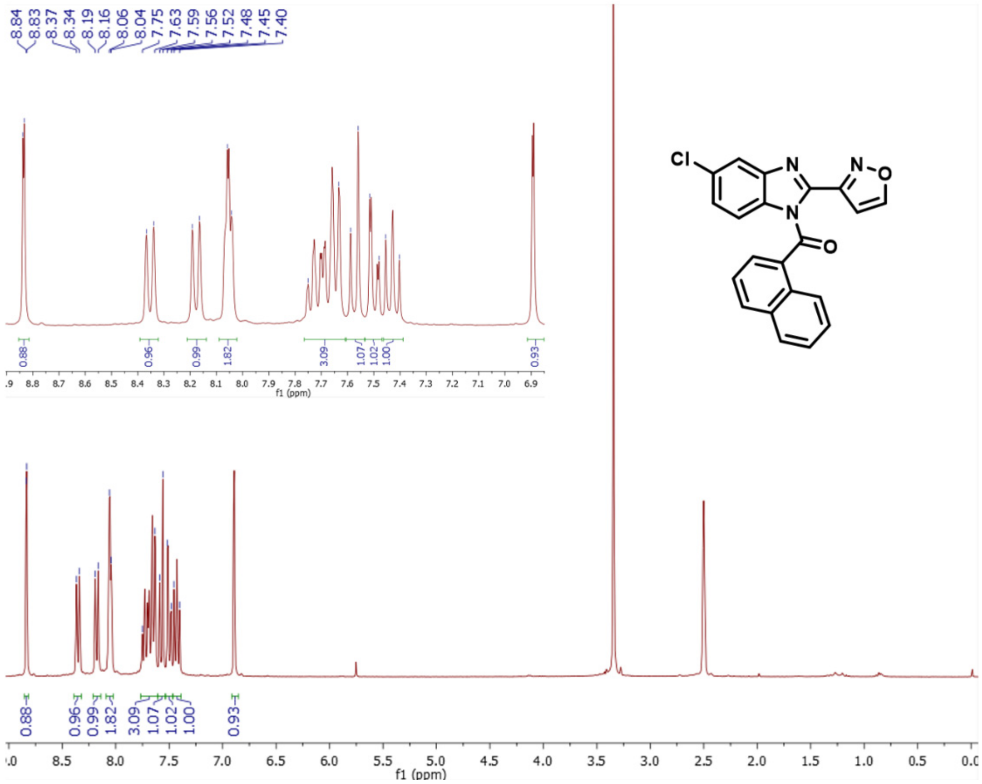

^{13}C NMR (75.5 MHz, DMSO-d^{6})

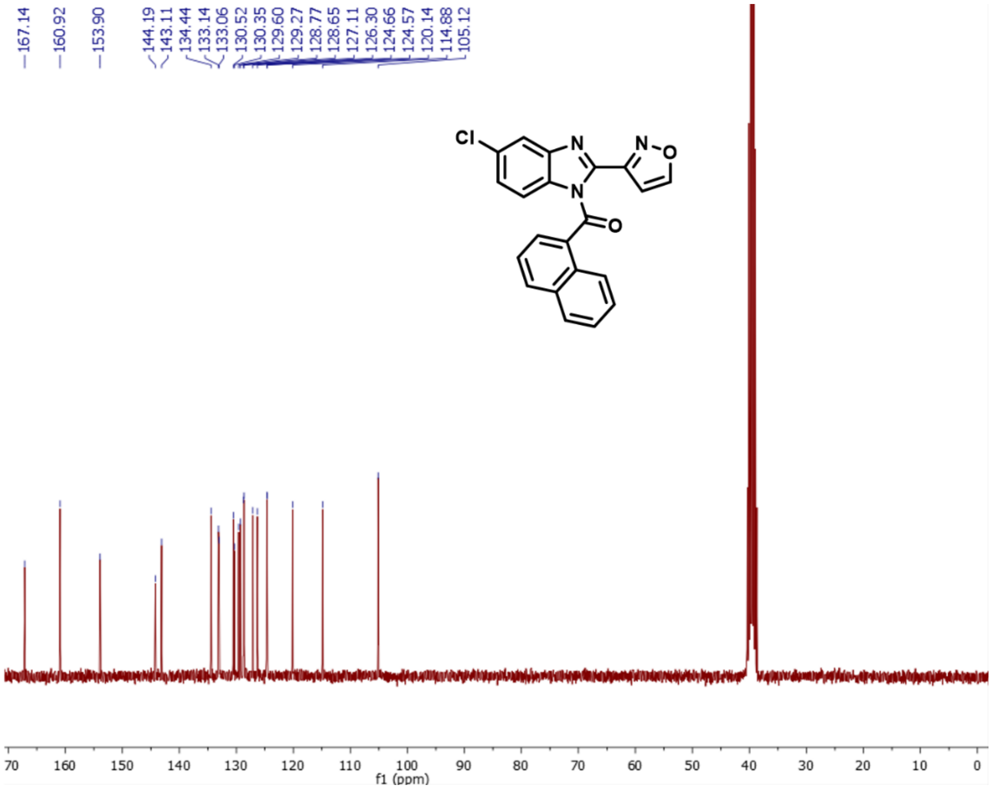

(6-chloro-2-(isoxazol-3-yl)-1*H*-benzo[*d*]imidazol-1-yl)(naphthalen-1-yl)methanone (**4d'**)

^{1}H NMR (300 MHz, DMSO-d^{6})

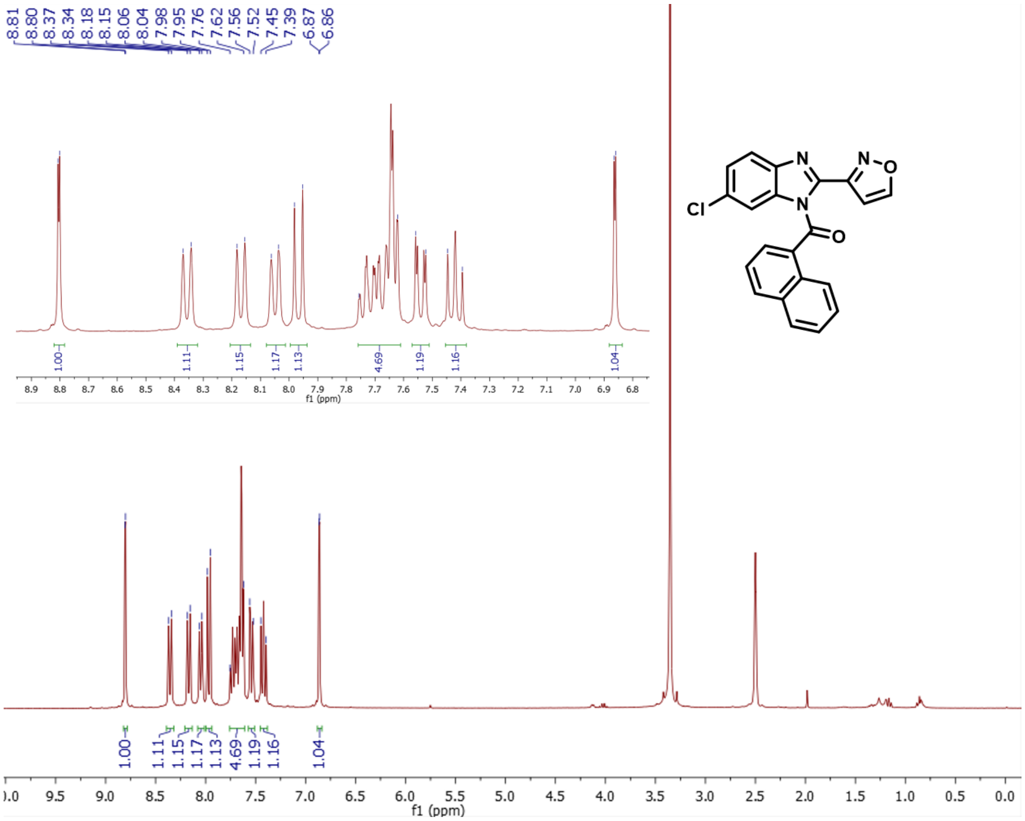

^{13}C NMR (75.5 MHz, DMSO-d^{6})

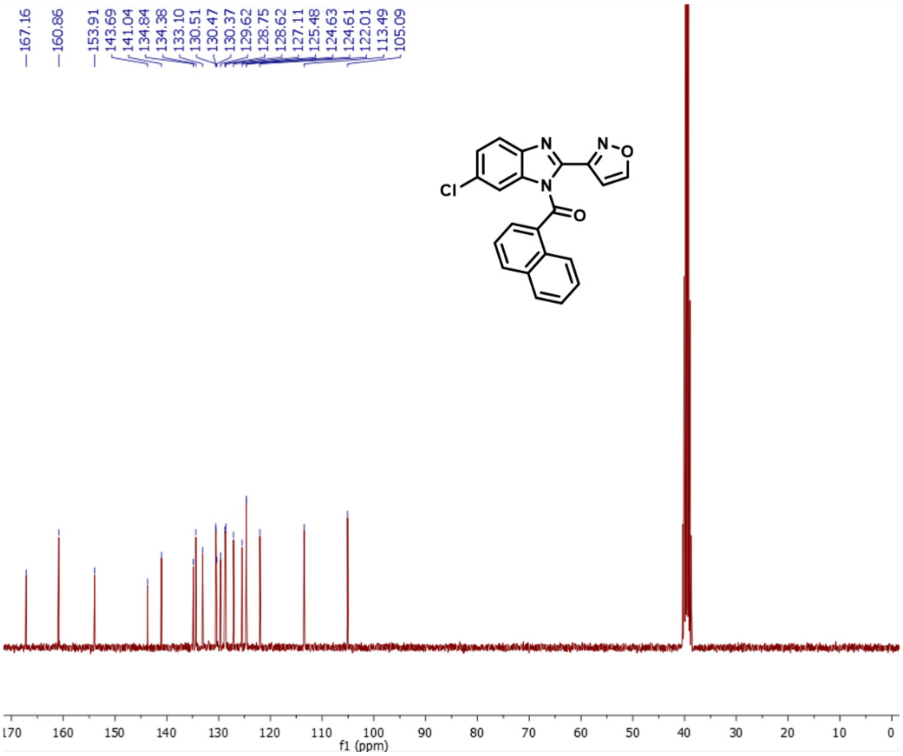

(5-chloro-2-(5-methylfuran-2-yl)-1*H*-benzo[*d*]imidazol-1-yl)(naphthalen-1-yl)methanone (**4e**)

^{1}H NMR (300 MHz, DMSO-d^{6})

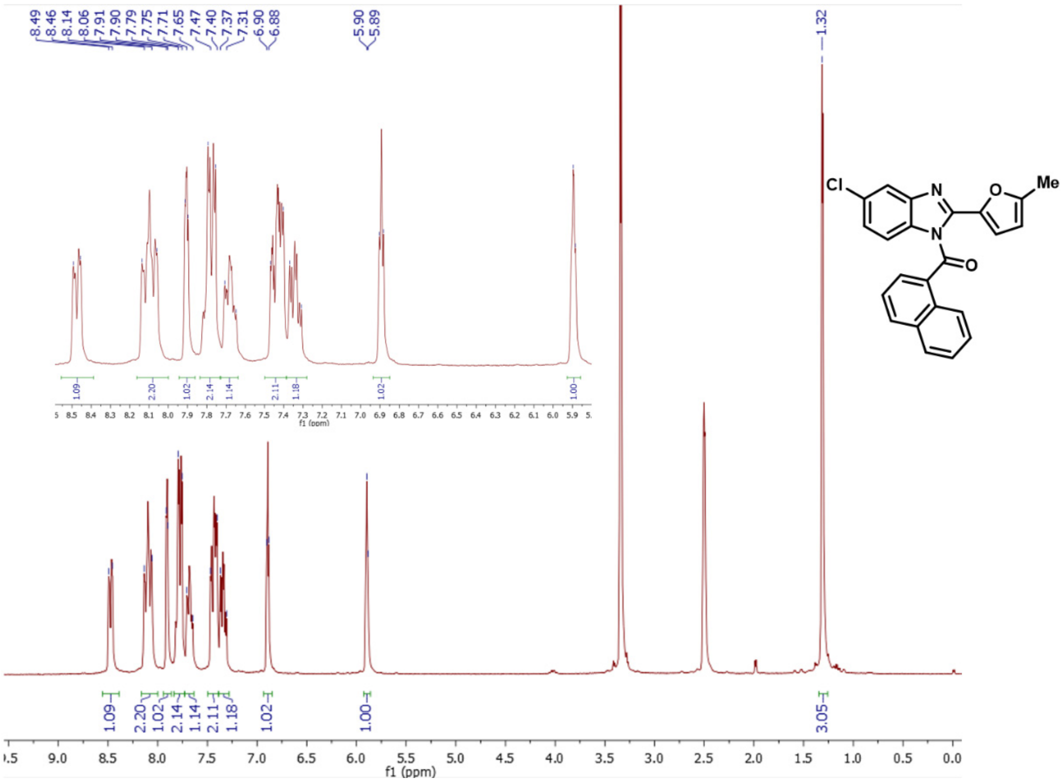

^{13}C NMR (75.5 MHz, DMSO-d^{6})

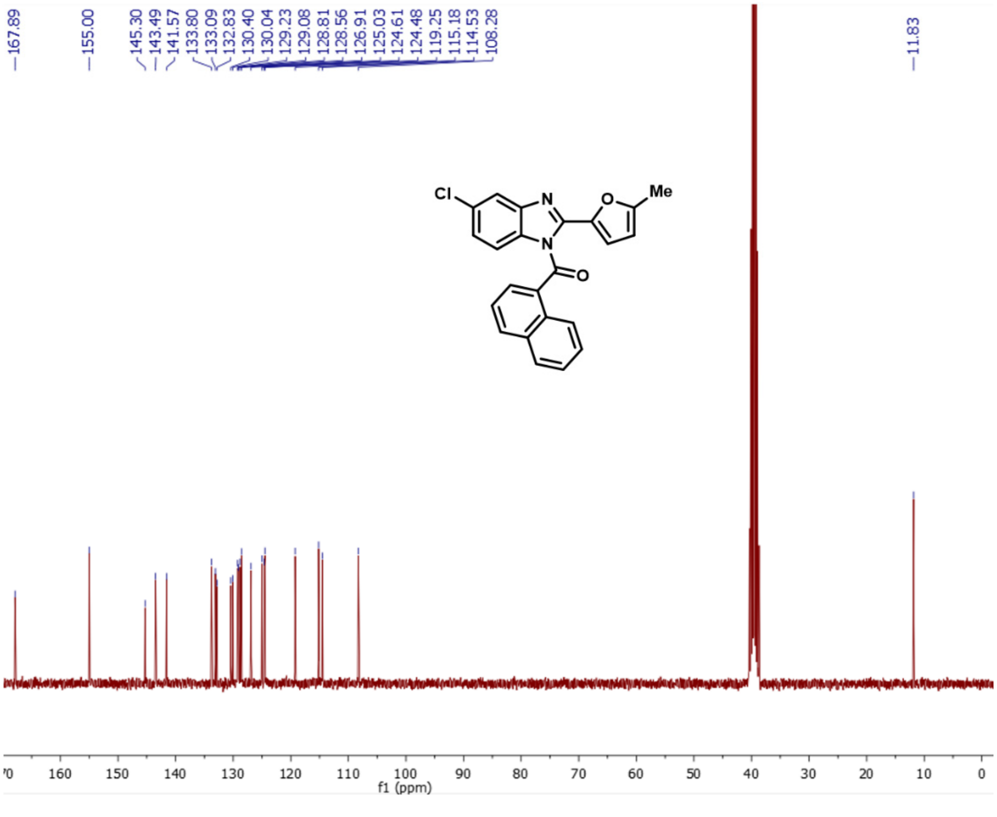

(6-chloro-2-(5-methylfuran-2-yl)-1*H*-benzo[*d*]imidazol-1-yl)(naphthalen-1-yl)methanone (**4e’**)

^{1}H NMR (300 MHz, DMSO-d^6)

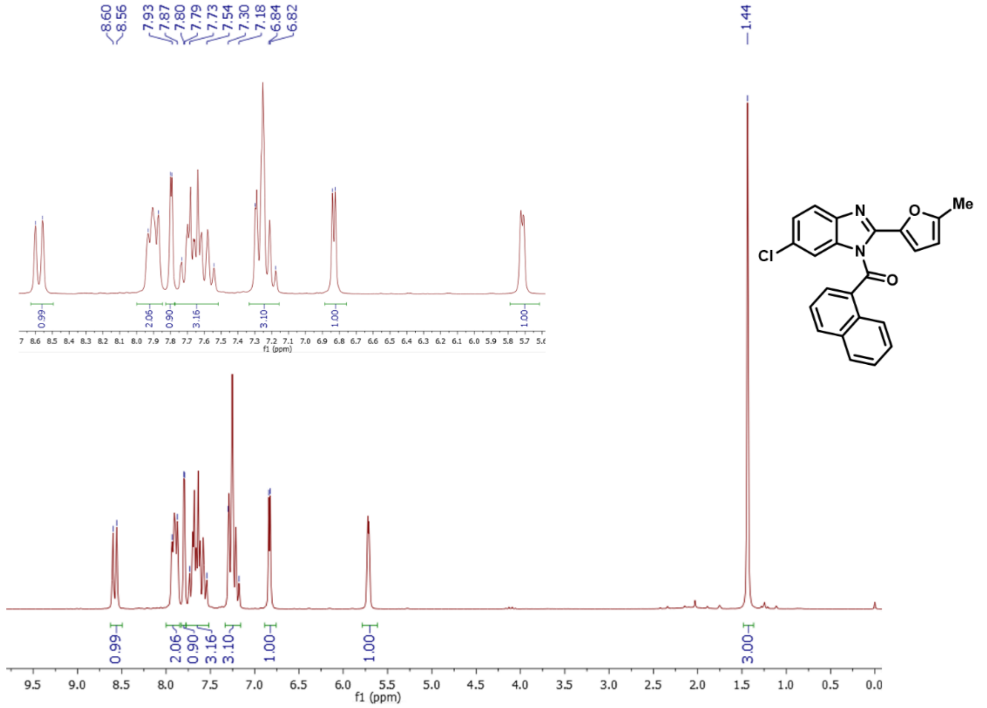

^{13}C NMR (75.5 MHz, DMSO-d^6)

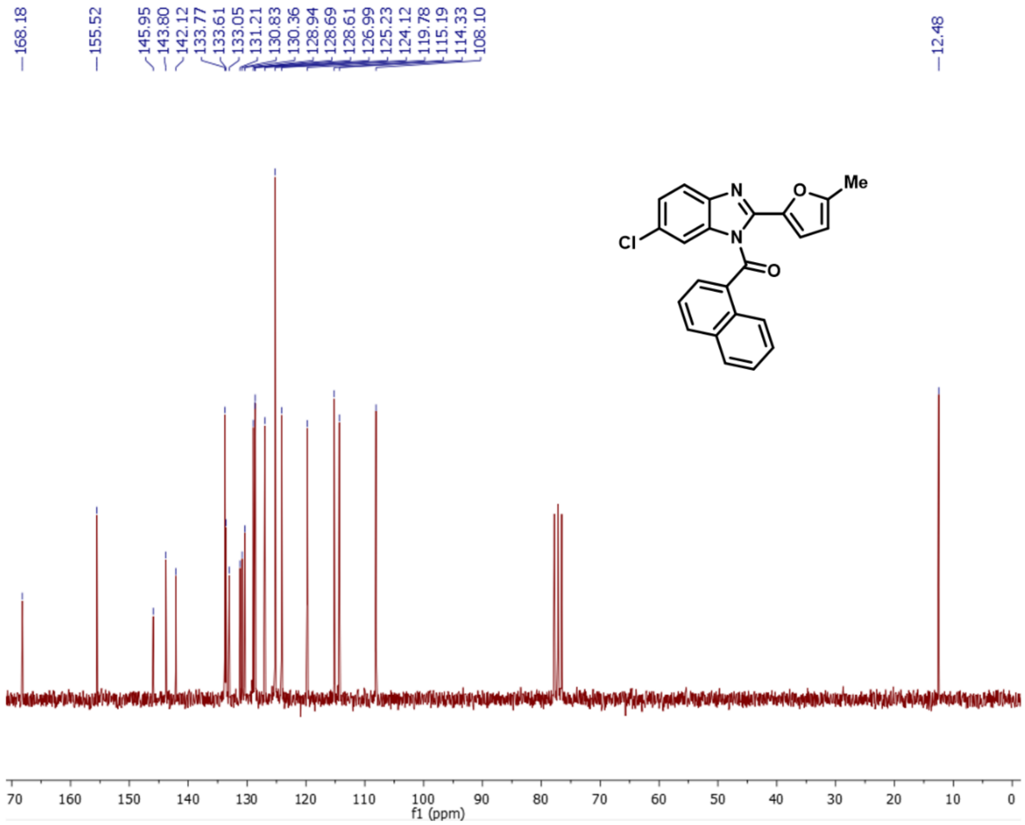

(5-chloro-2-(furan-2-yl)-1*H*-benzo[*d*]imidazol-1-yl)(naphthalen-1-yl)methanone (**4f**)

^{1}H NMR (300 MHz, DMSO-d^{6})

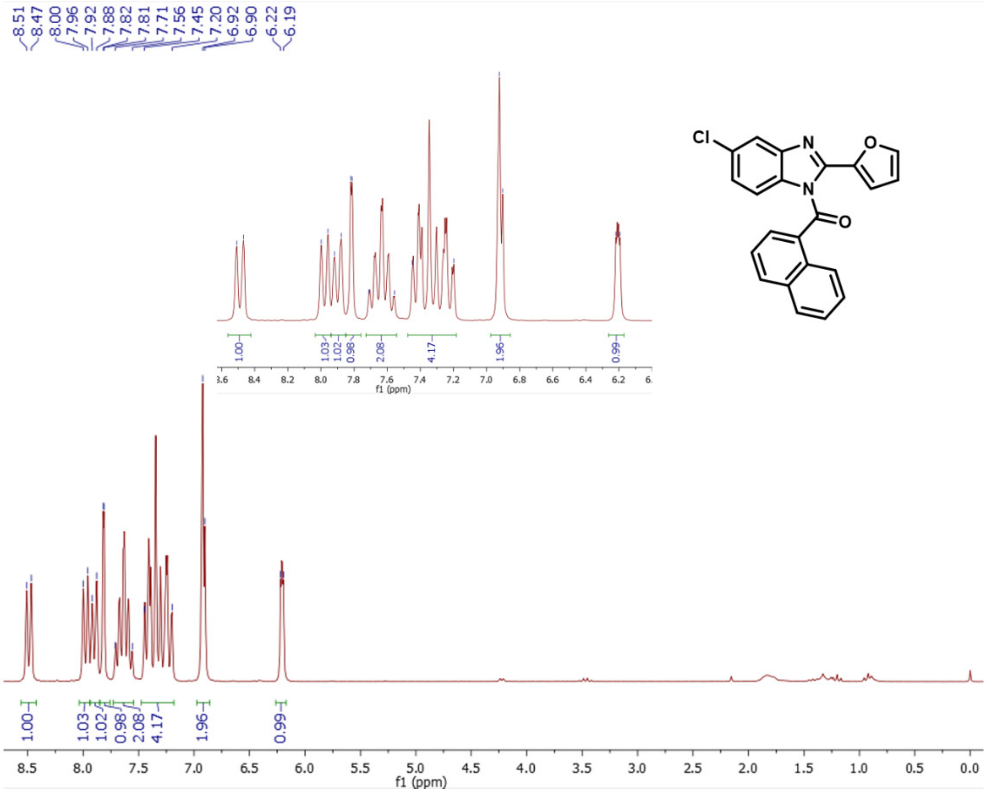

^{13}C NMR (75.5 MHz, DMSO-d^{6})

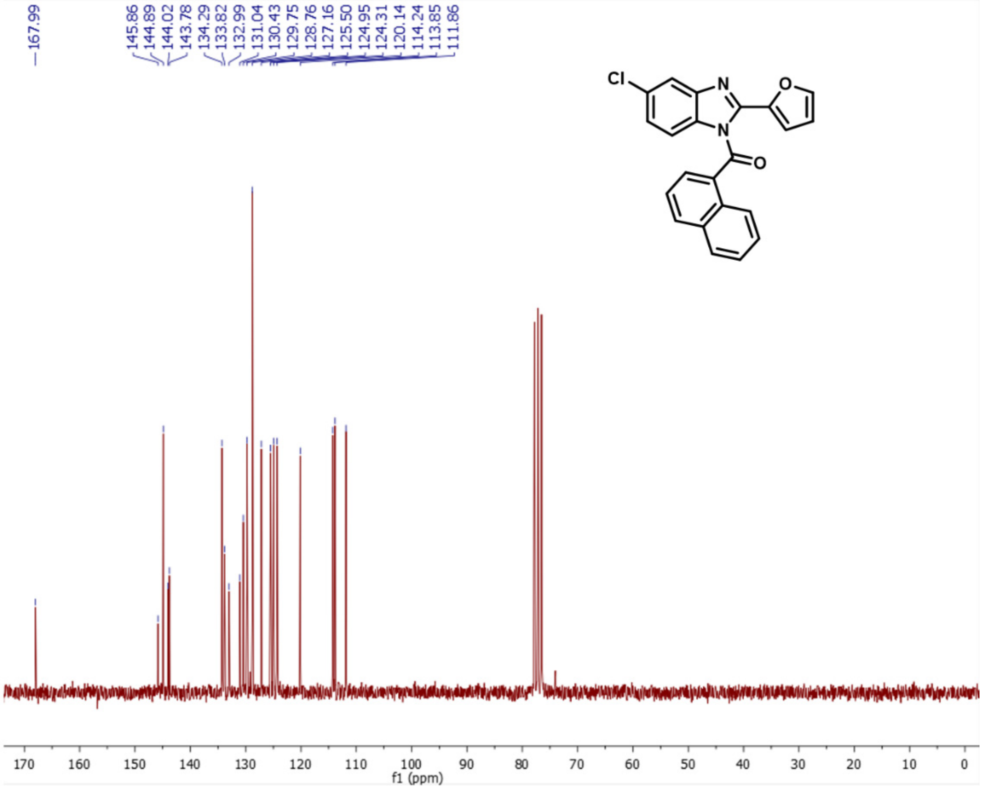

(6-chloro-2-(furan-2-yl)-1*H*-benzo[*d*]imidazol-1-yl)(naphthalen-1-yl)methanone (**4f'**)

^{1}H NMR (300 MHz, DMSO-d^{6})

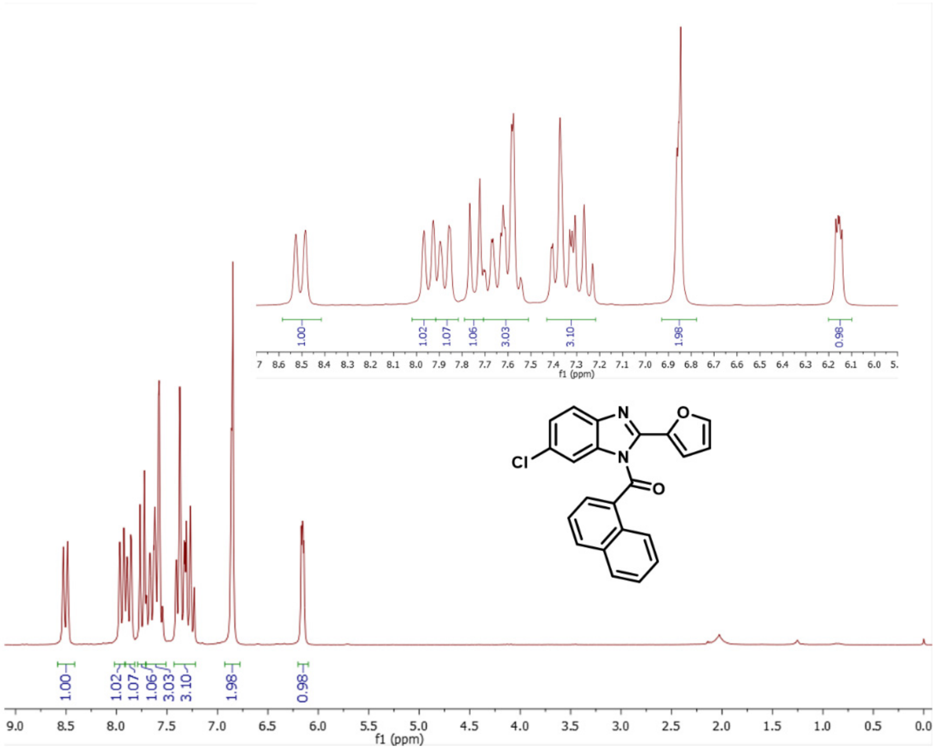

^{13}C NMR (75.5 MHz, DMSO-d^{6})

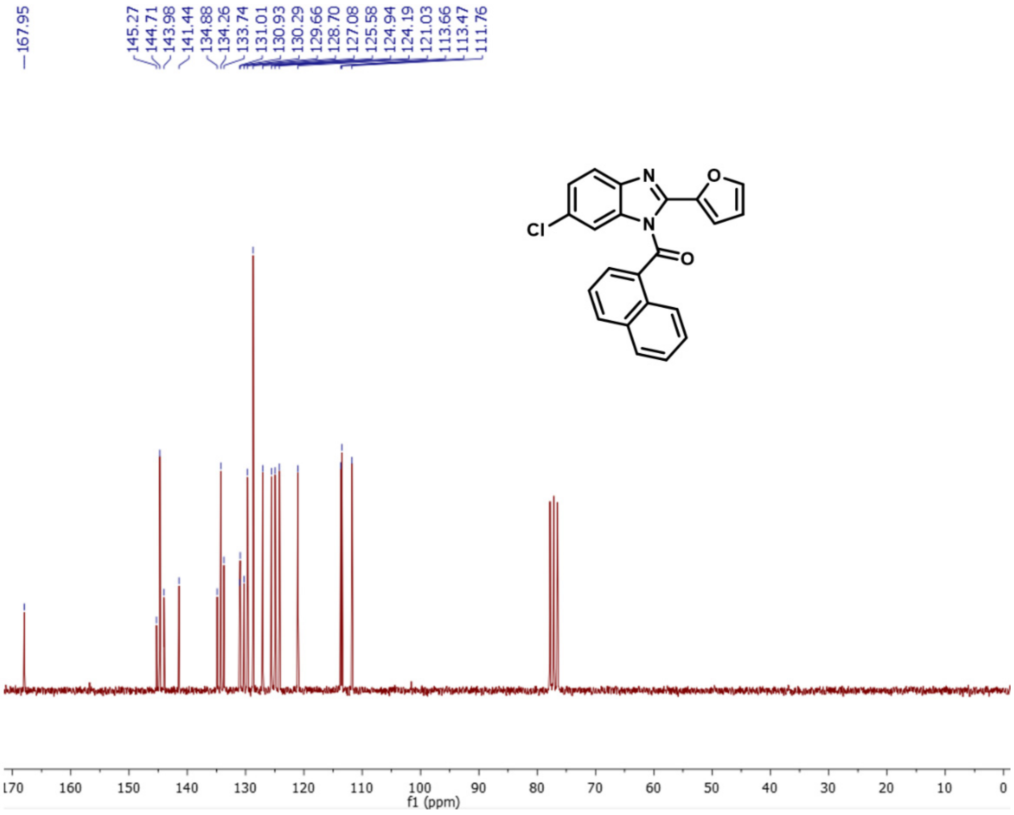

(5-fluoro-2-(pyridin-3-yl)-1*H*-benzo[*d*]imidazol-1-yl)(naphthalen-1-yl)methanone (**5a**)

^{1}H NMR (300 MHz, DMSO-d^{6})

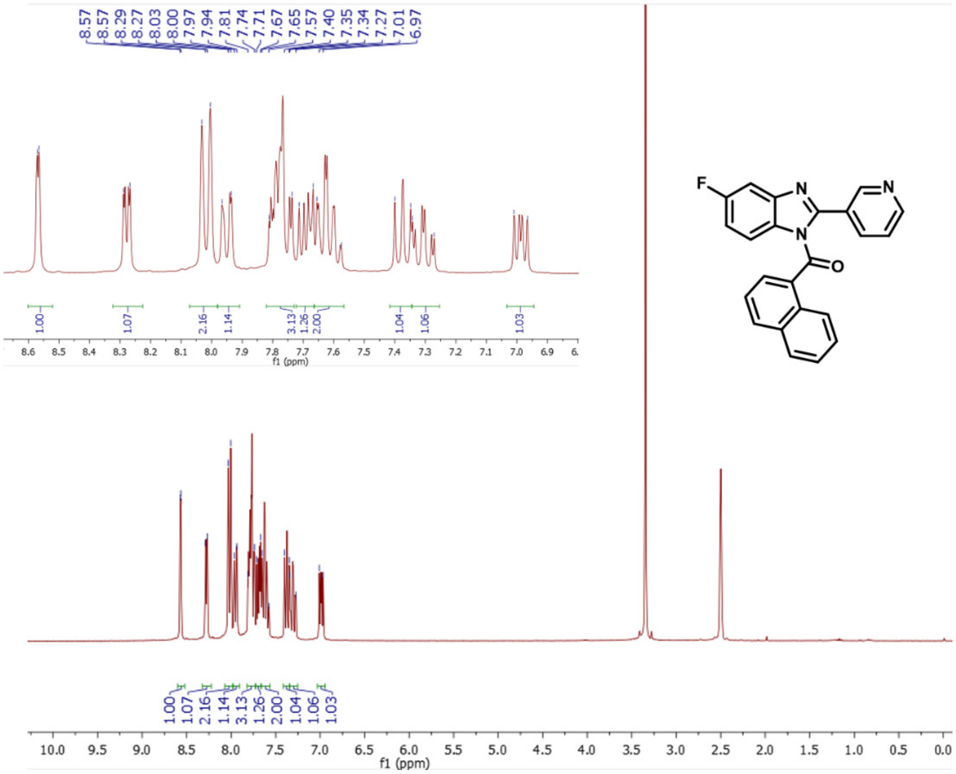

^{13}C NMR (75.5 MHz, DMSO-d^{6})

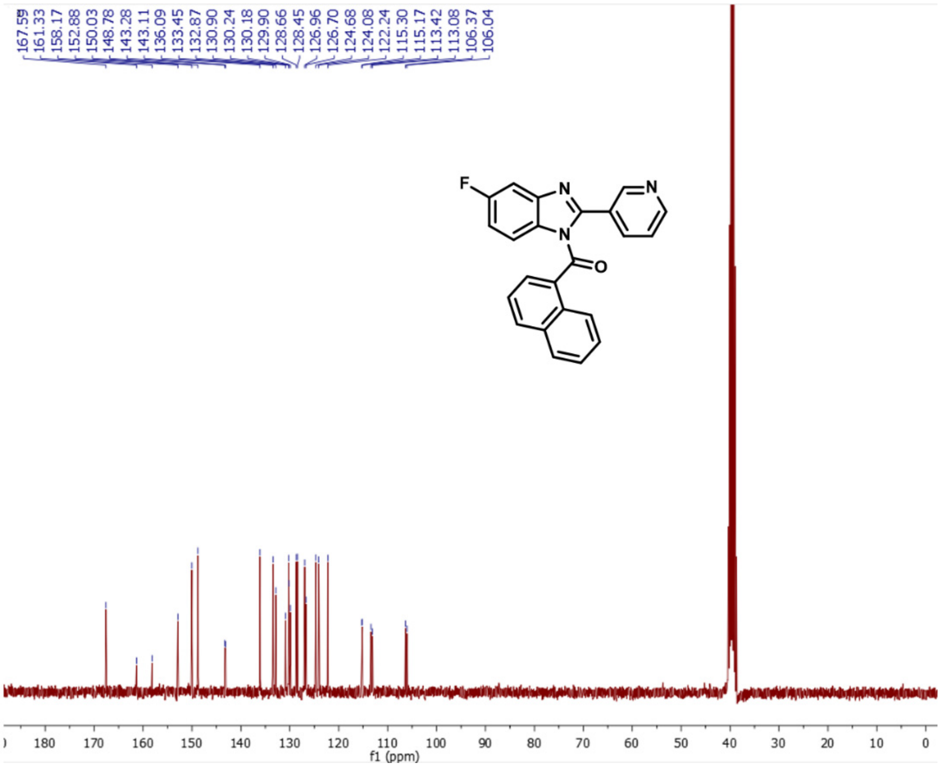

^{19}F NMR (282 MHz, DMSO-d^6)

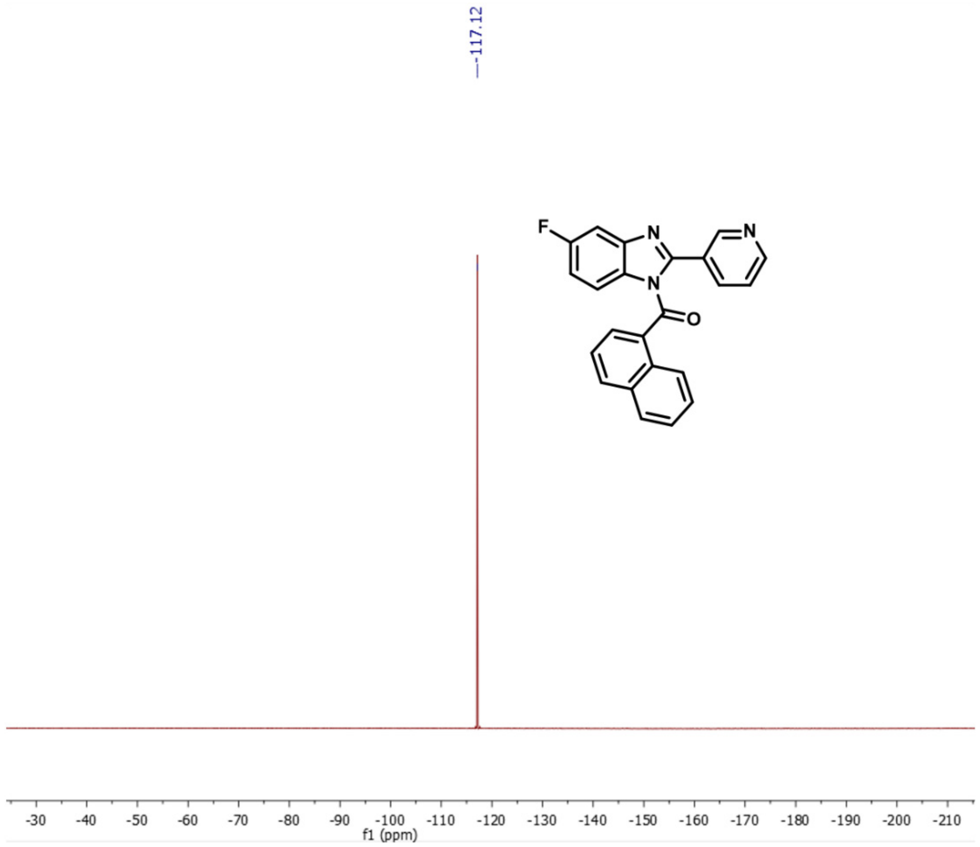

(6-fluoro-2-(pyridin-3-yl)-1*H*-benzo[*d*]imidazol-1-yl)(naphthalen-1-yl)methanone (**5a'**)

^{1}H NMR (300 MHz, DMSO-d^{6})

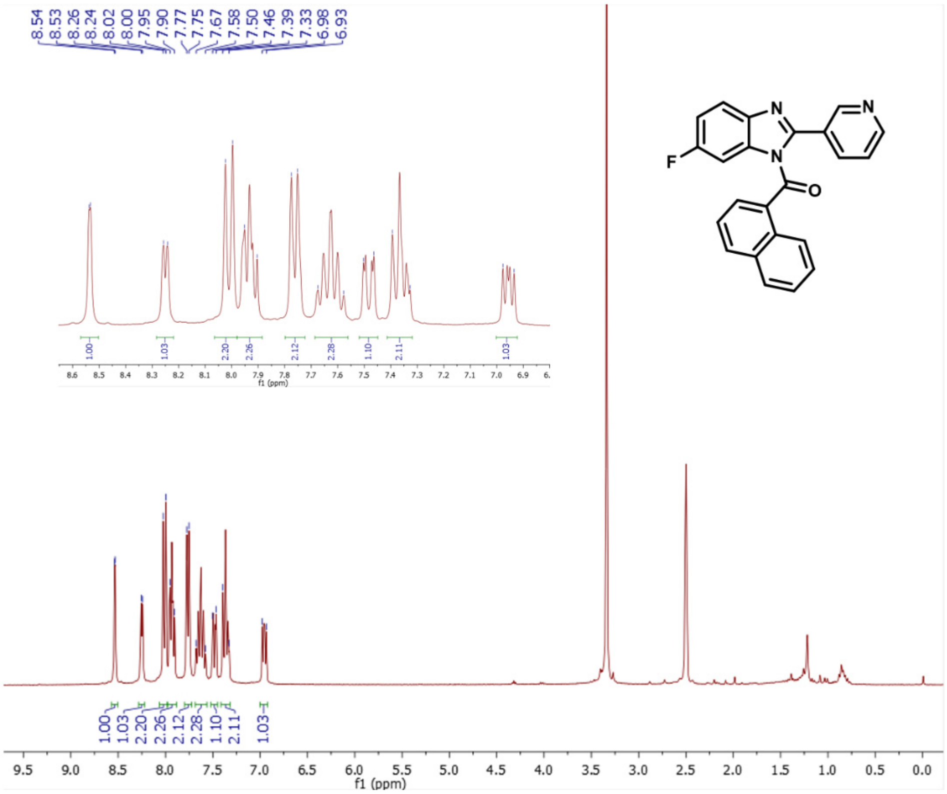

^{13}C NMR (75.5 MHz, DMSO-d^{6})

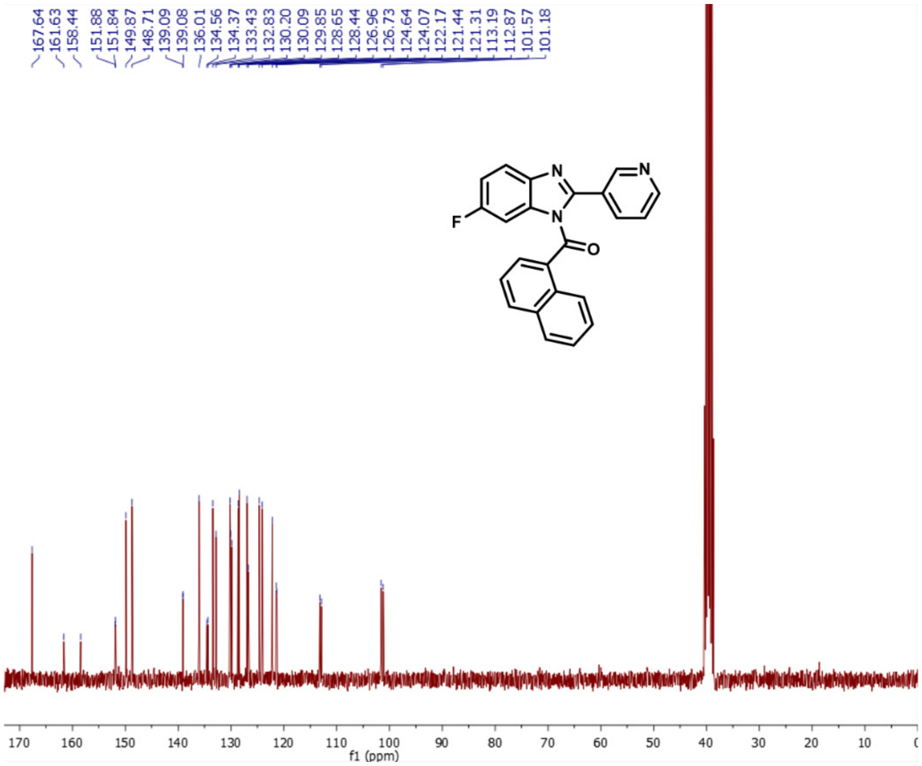

^{19}F NMR (282 MHz, DMSO-d^{6})

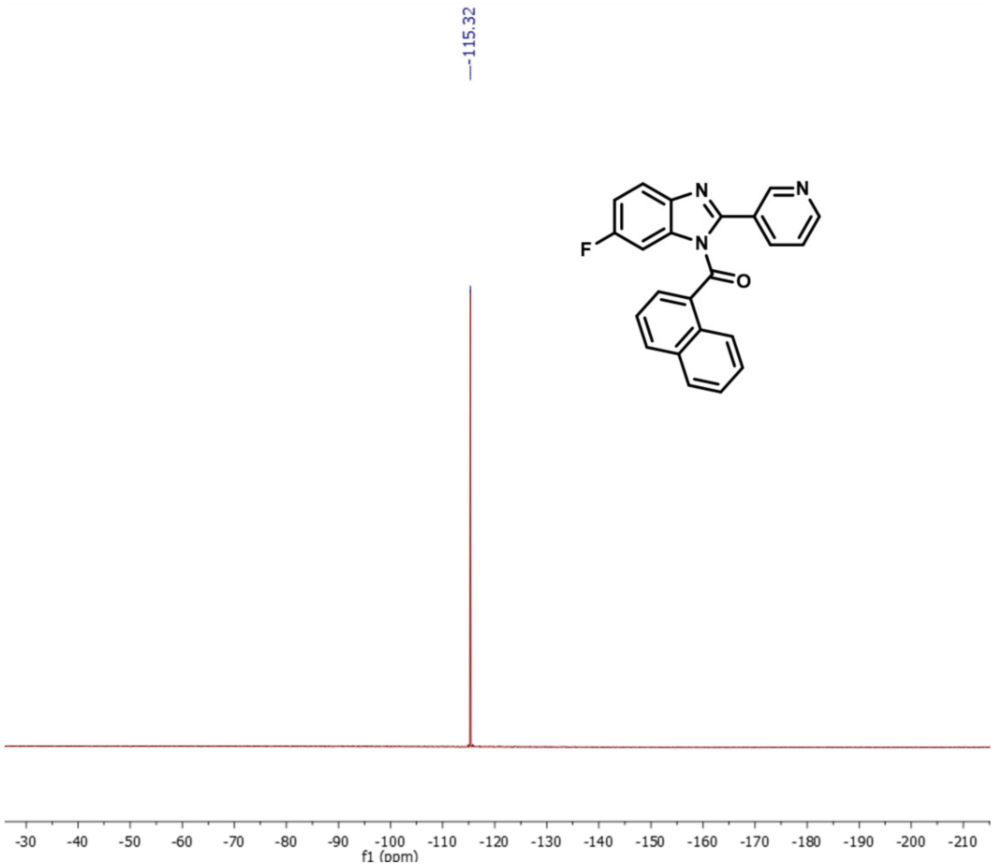

(5-fluoro-2-(3-methoxyphenyl)-1*H*-benzo[*d*]imidazol-1-yl)(naphthalen-1-yl)methanone (**5b**)

^{1}H NMR (300 MHz, DMSO-d^{6})

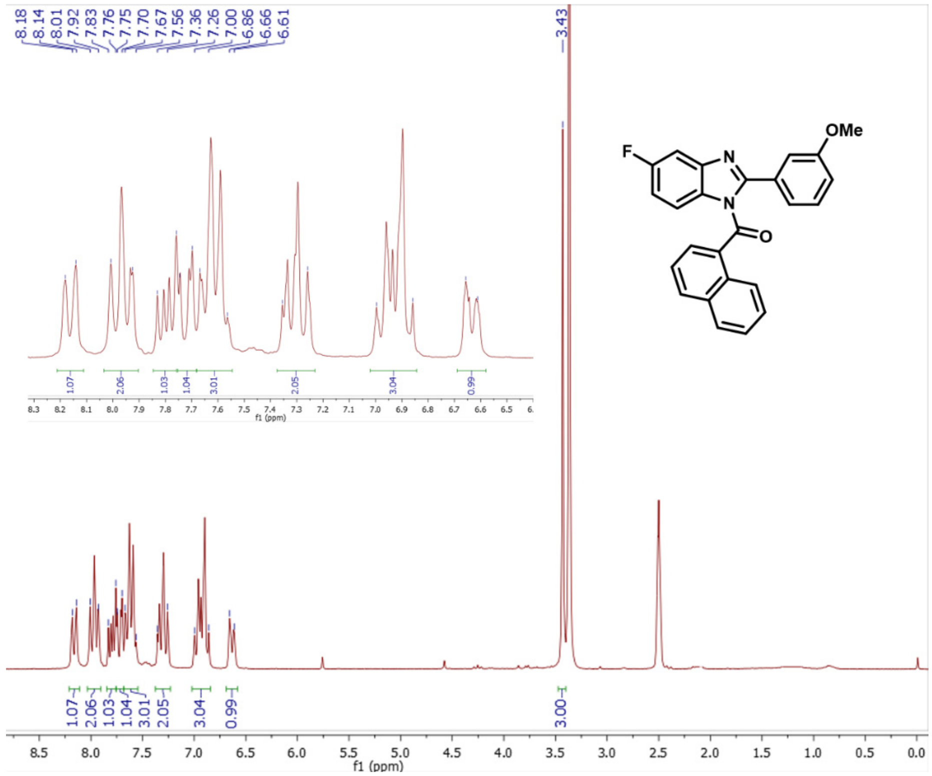

^{13}C NMR (75.5 MHz, DMSO-d^{6})

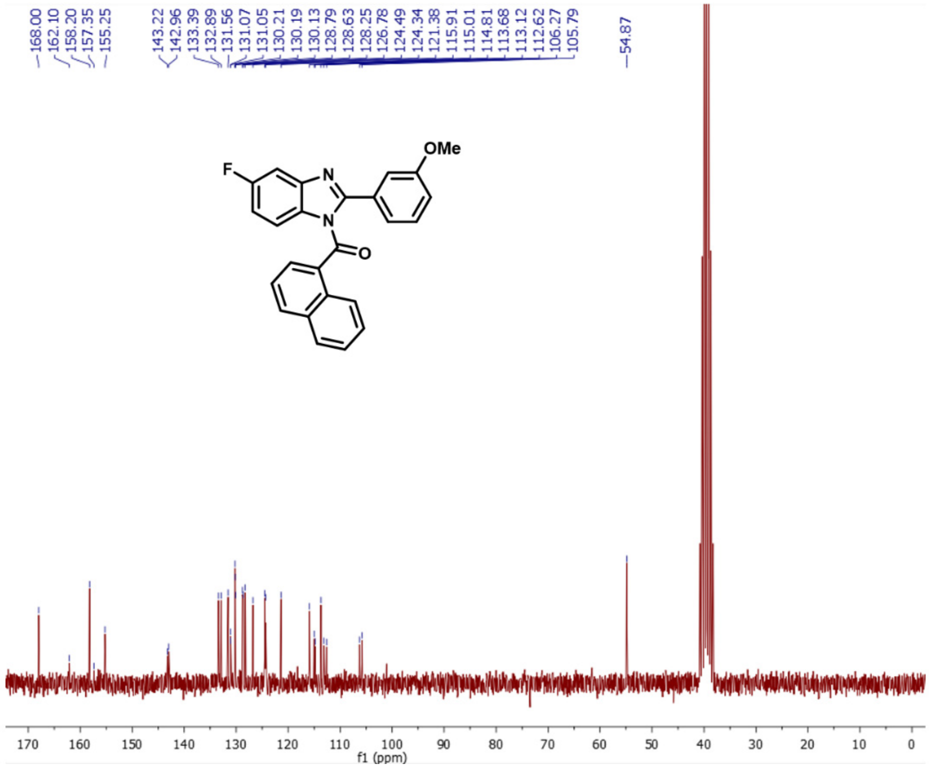

^{19}F NMR (282 MHz, DMSO-d^{6})

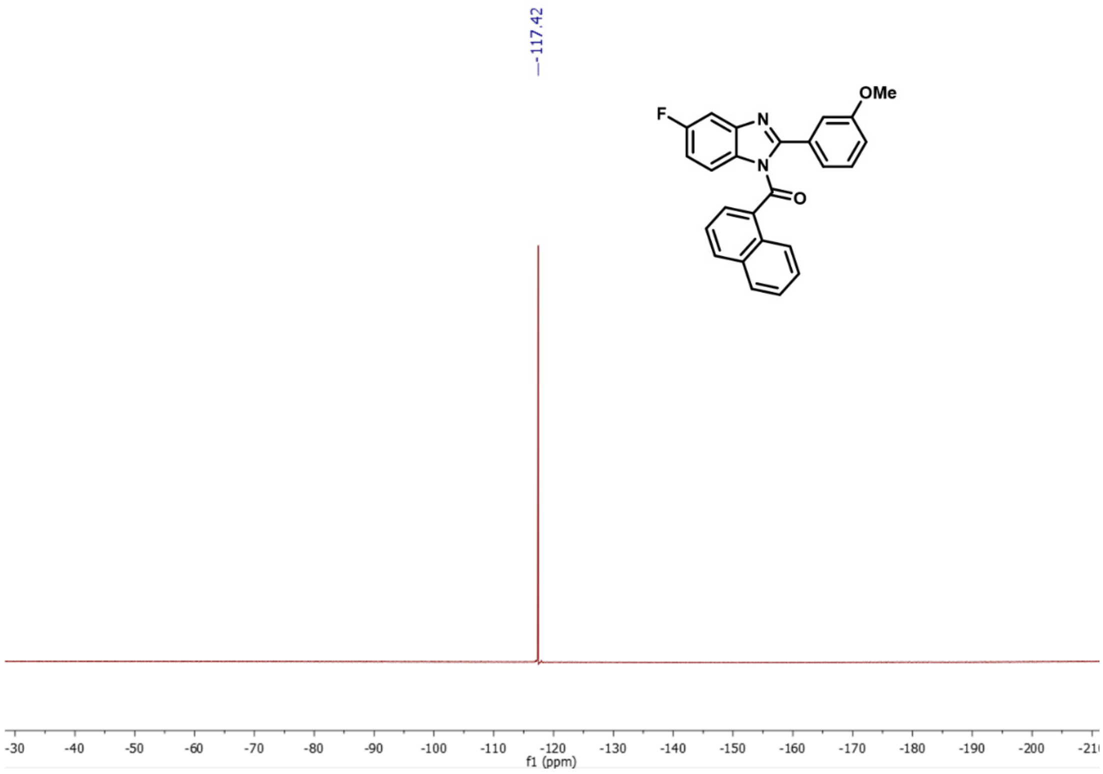

(6-fluoro-2-(3-methoxyphenyl)-1*H*-benzo[*d*]imidazol-1-yl)(naphthalen-1-yl)methanone (**5b'**)

^{1}H NMR (300 MHz, DMSO-d^{6})

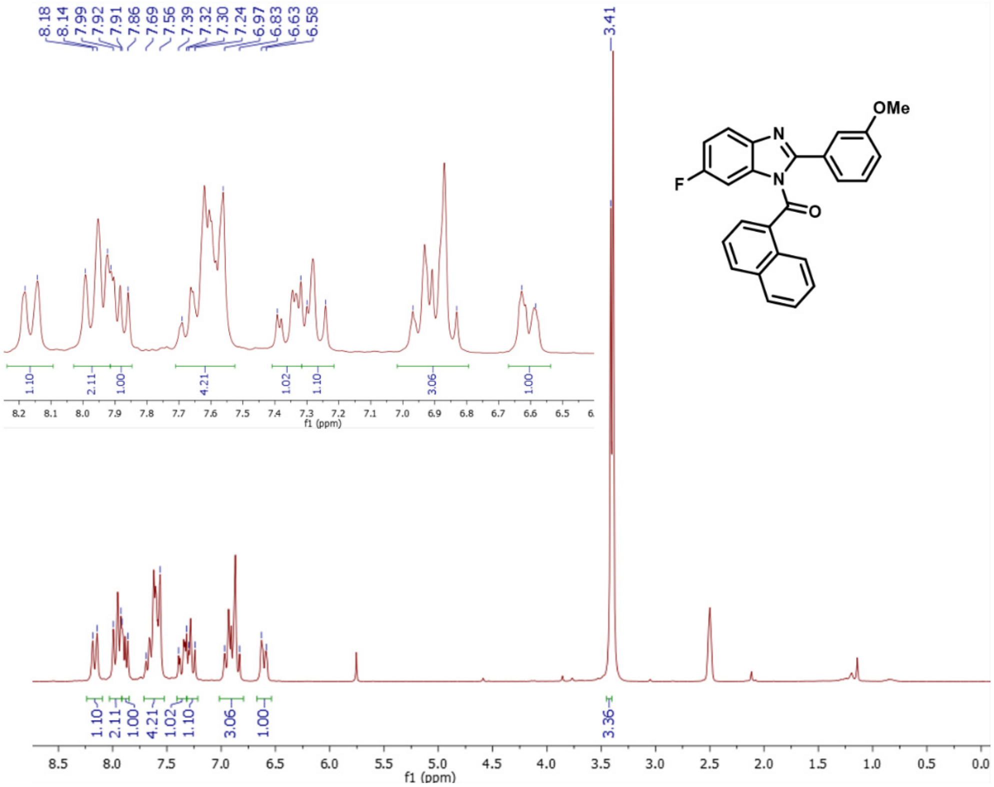

^{13}C NMR (75.5 MHz, DMSO-d^{6})

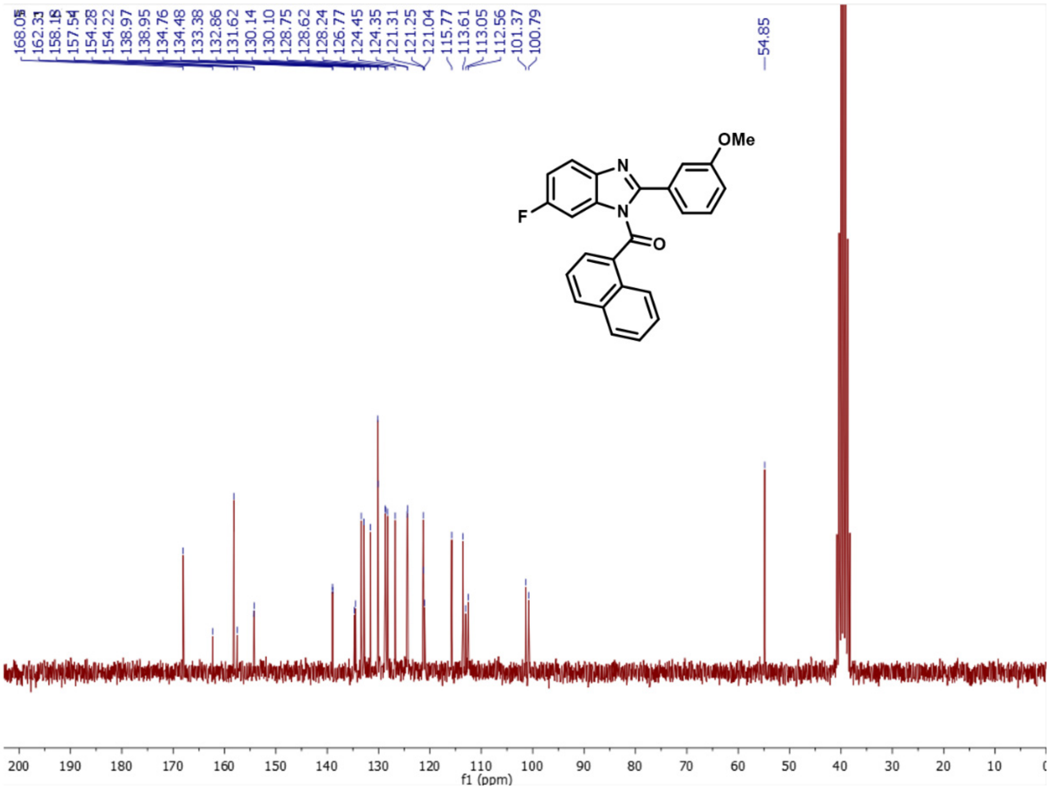

^{19}F NMR (282 MHz, DMSO-d^{6})

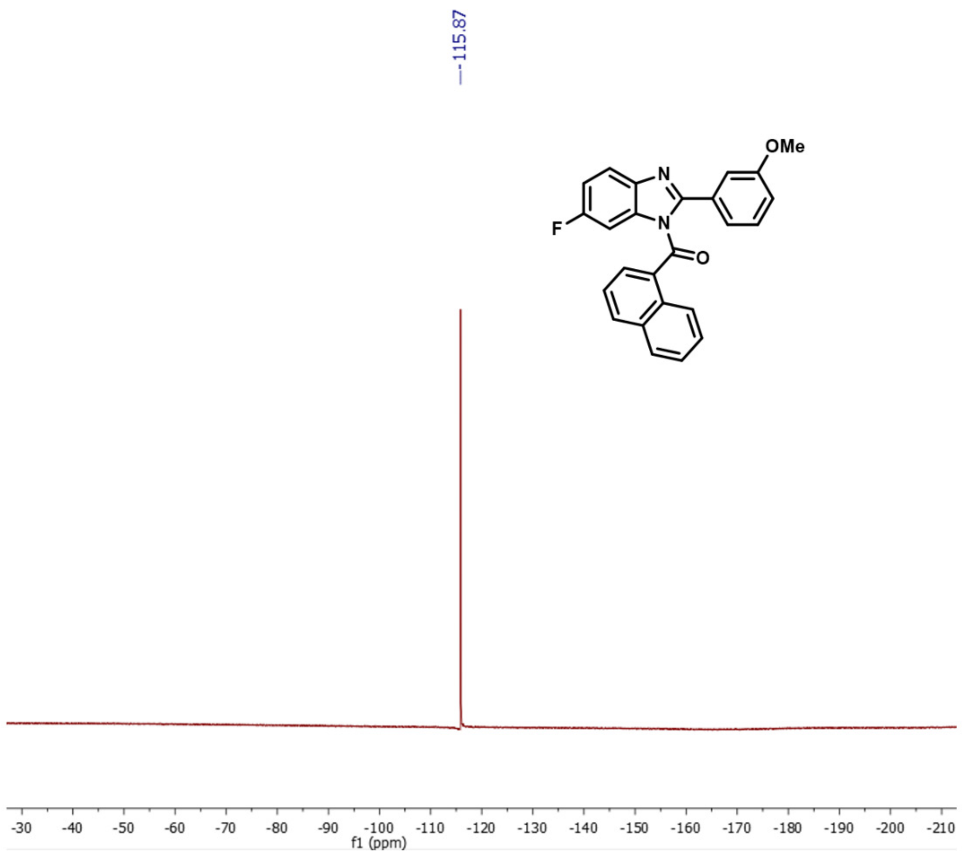

(5-fluoro-2-(5-methylisoxazol-3-yl)-1*H*-benzo[*d*]imidazol-1-yl)(naphthalen-1-yl)methanone (**5c**)

^{1}H NMR (300 MHz, DMSO-d^{6})

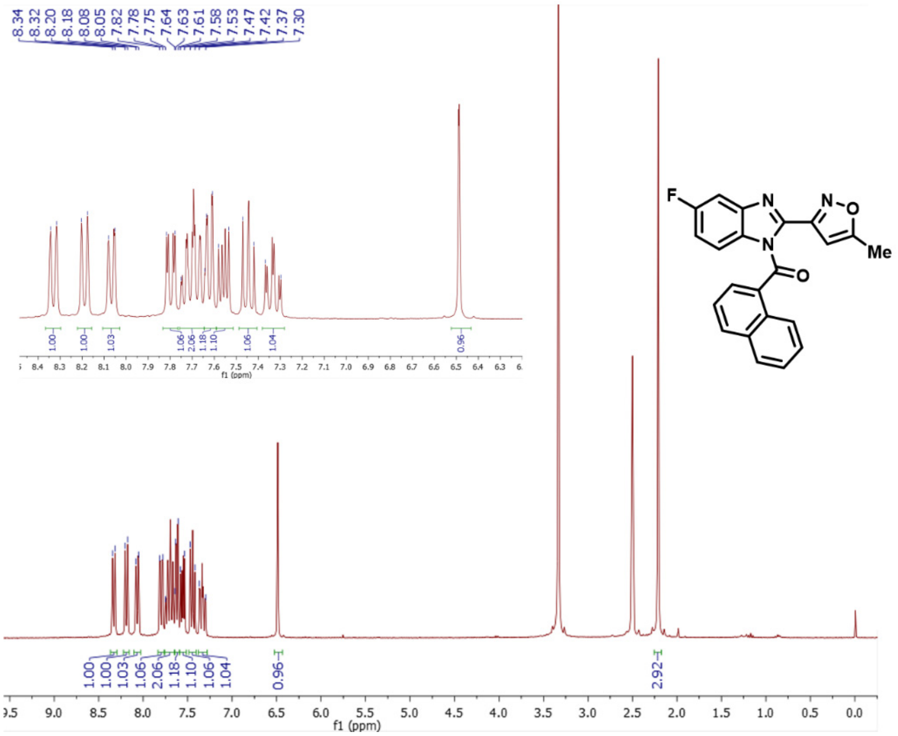

^{13}C NMR (75.5 MHz, DMSO-d^{6})

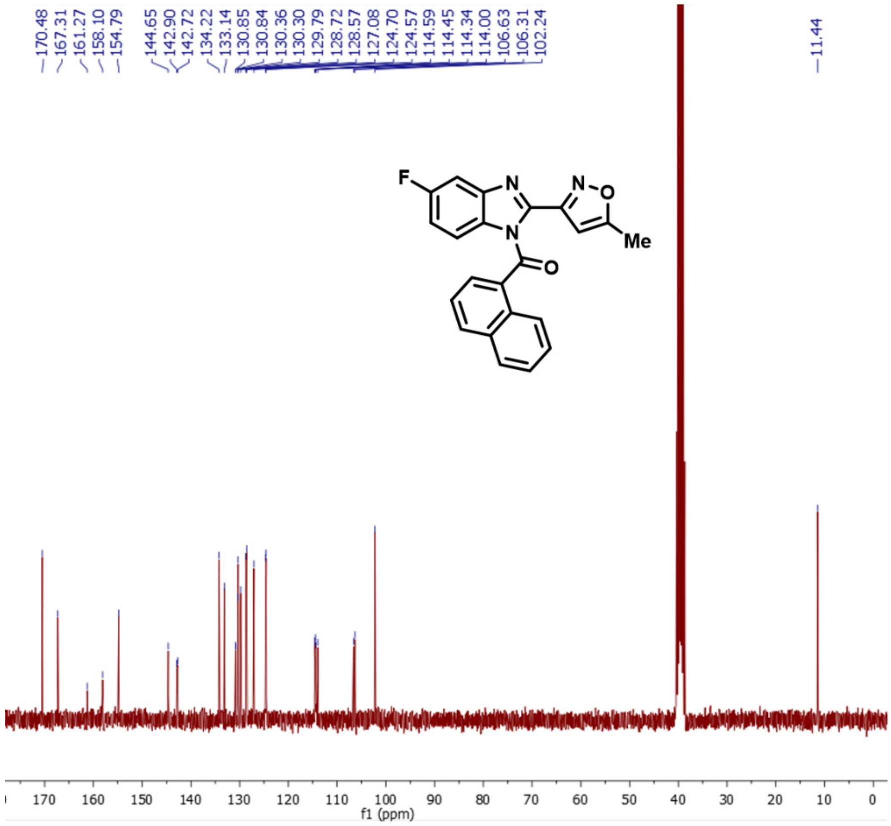

^{19}F NMR (282 MHz, DMSO-d^6)

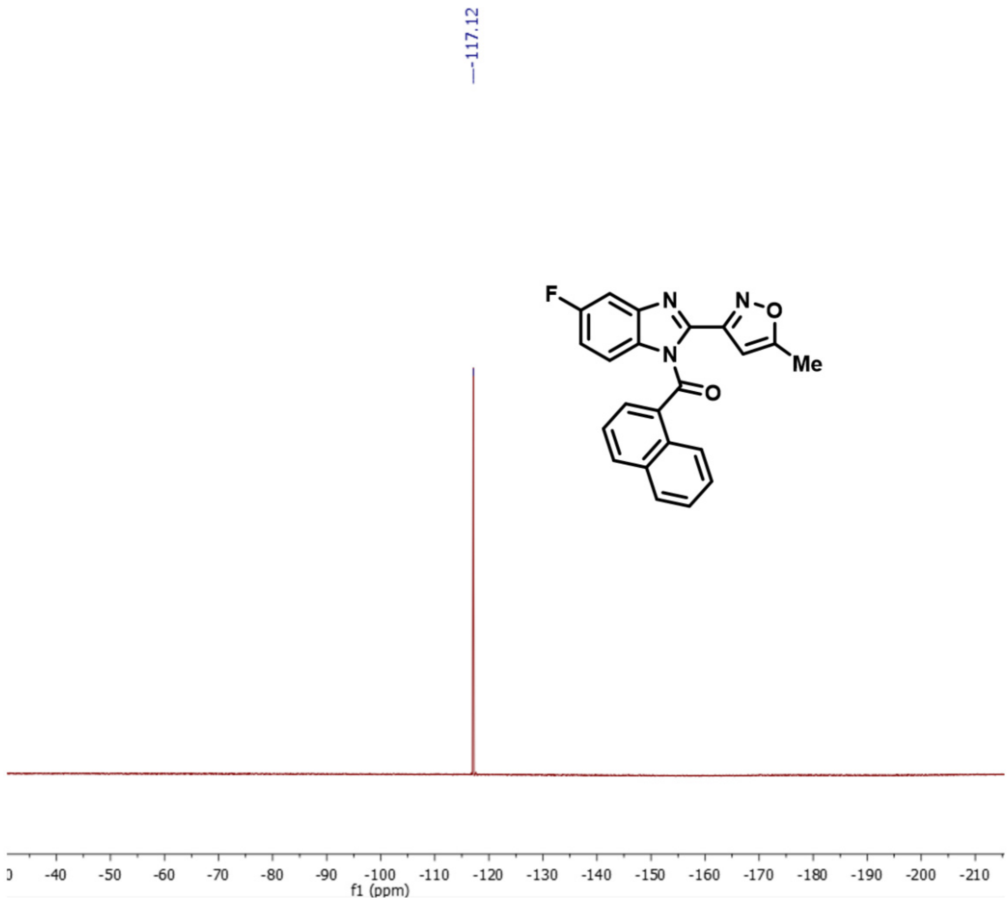

(6-fluoro-2-(5-methylisoxazol-3-yl)-1*H*-benzo[*d*]imidazol-1-yl)(naphthalen-1-yl)methanone (**5c'**)

^{1}H NMR (300 MHz, DMSO-d^6)

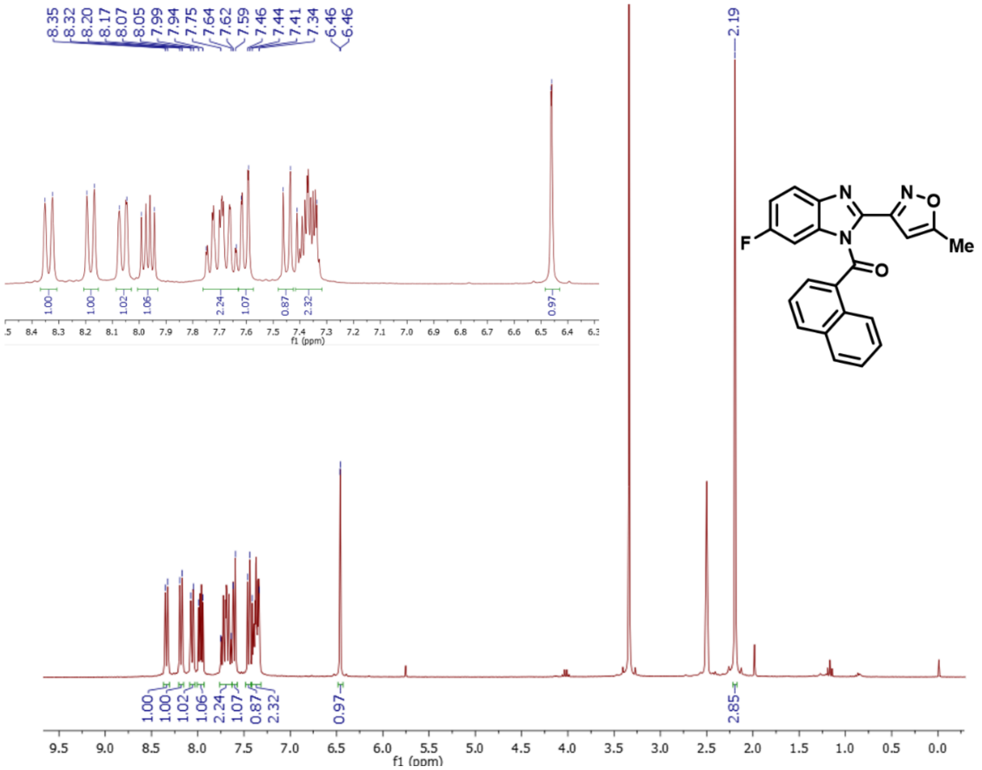

^{13}C NMR (75.5 MHz, DMSO-d^6)

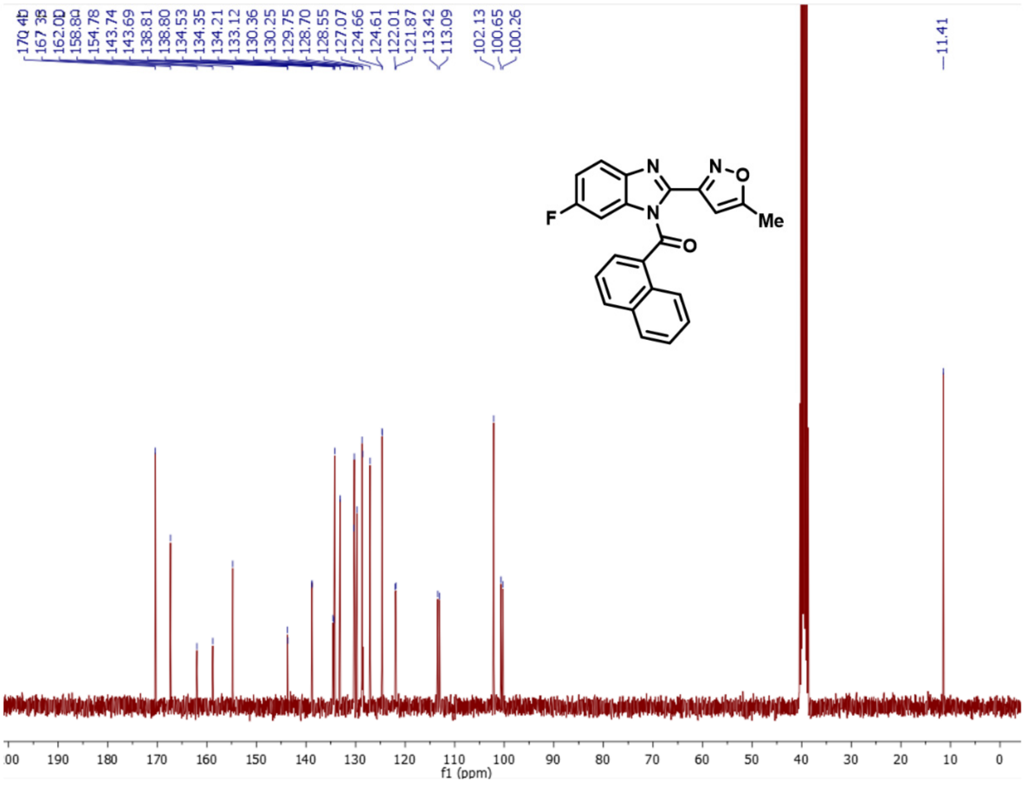

^{19}F NMR (282 MHz, DMSO-d6)

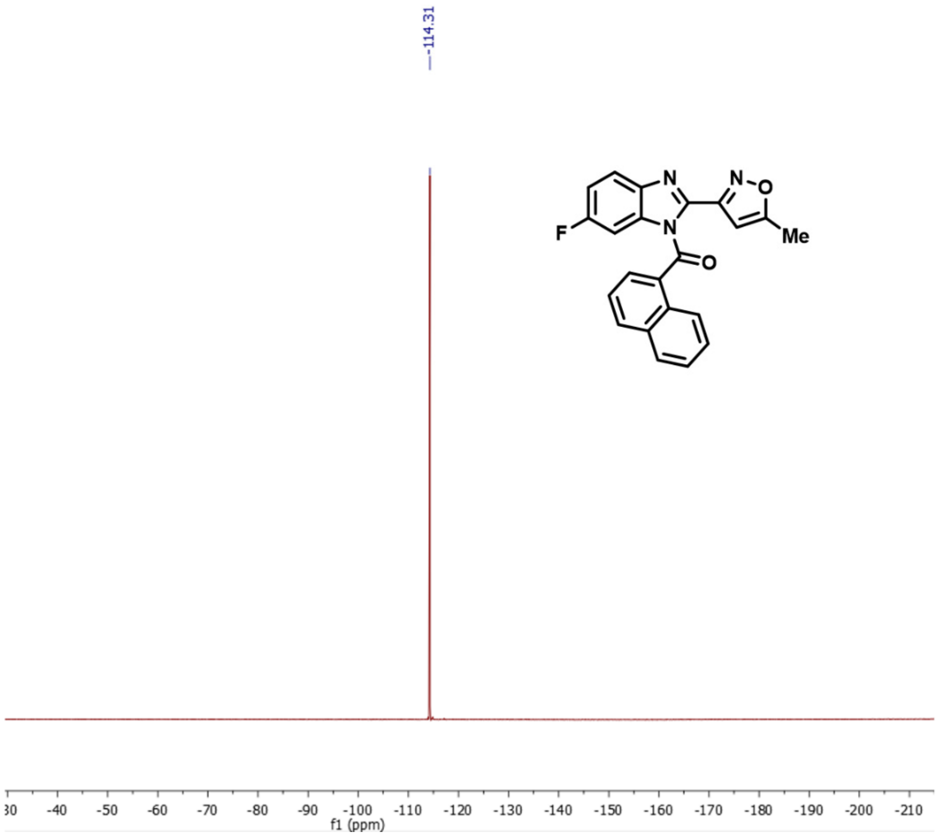

(5-fluoro-2-(isoxazol-3-yl)-1*H*-benzo[*d*]imidazol-1-yl)(naphthalen-1-yl)methanone (**5d**)

^{1}H NMR (300 MHz, DMSO-d^{6})

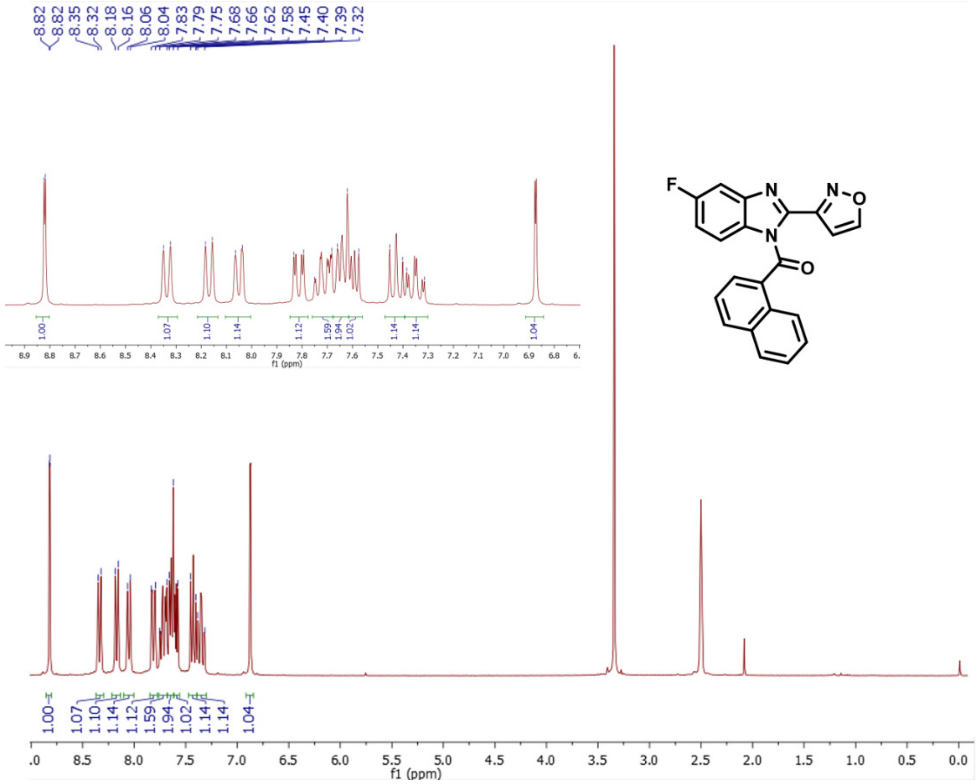

^{13}C NMR (75.5 MHz, DMSO-d^{6})

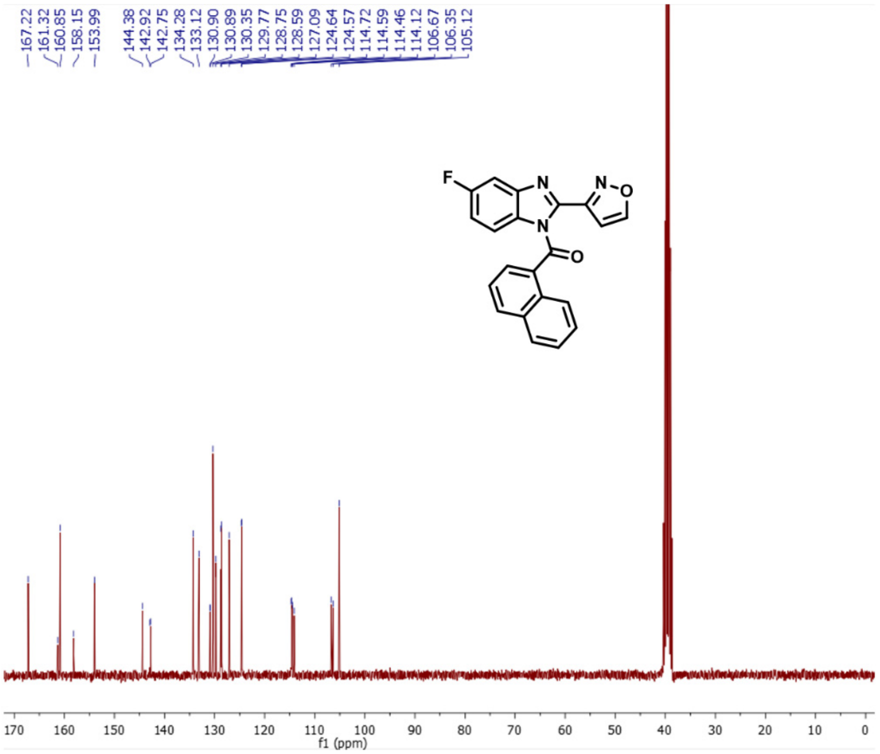

^{19}F NMR (282 MHz, DMSO-d^{6})

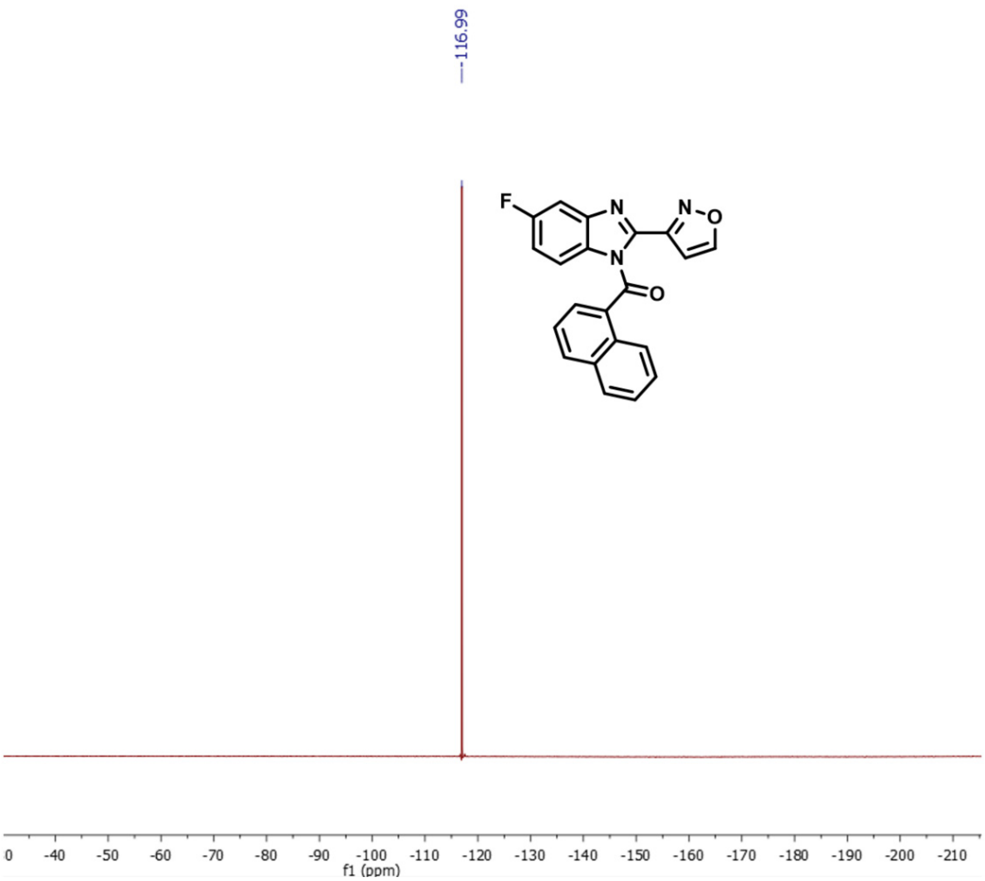

(6-fluoro-2-(isoxazol-3-yl)-1*H*-benzo[*d*]imidazol-1-yl)(naphthalen-1-yl)methanone (**5d’**)

^{1}H NMR (300 MHz, DMSO-d^{6})

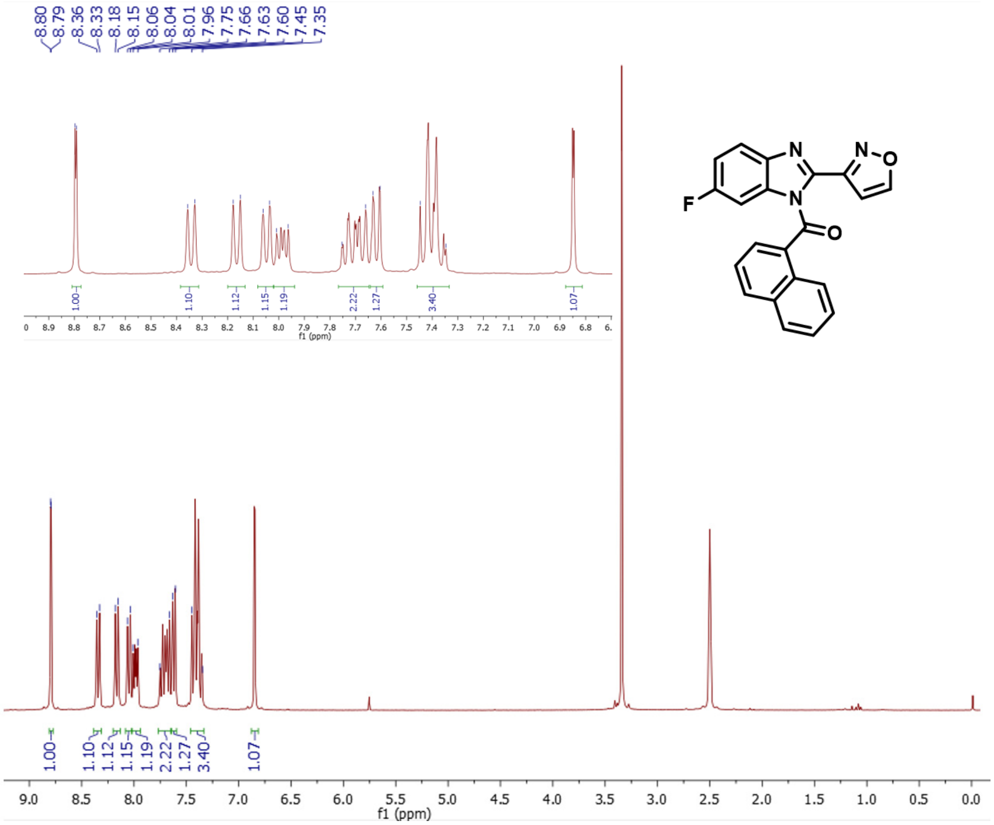

^{13}C NMR (75.5 MHz, DMSO-d^{6})

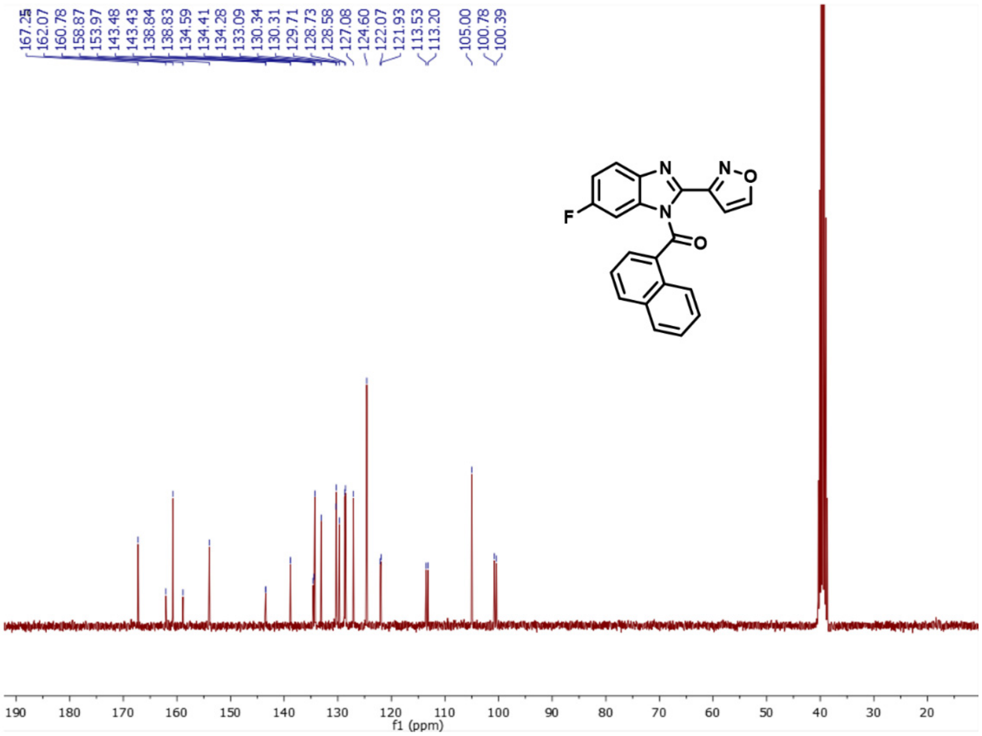

^{19}F NMR (282 MHz, DMSO-d^6)

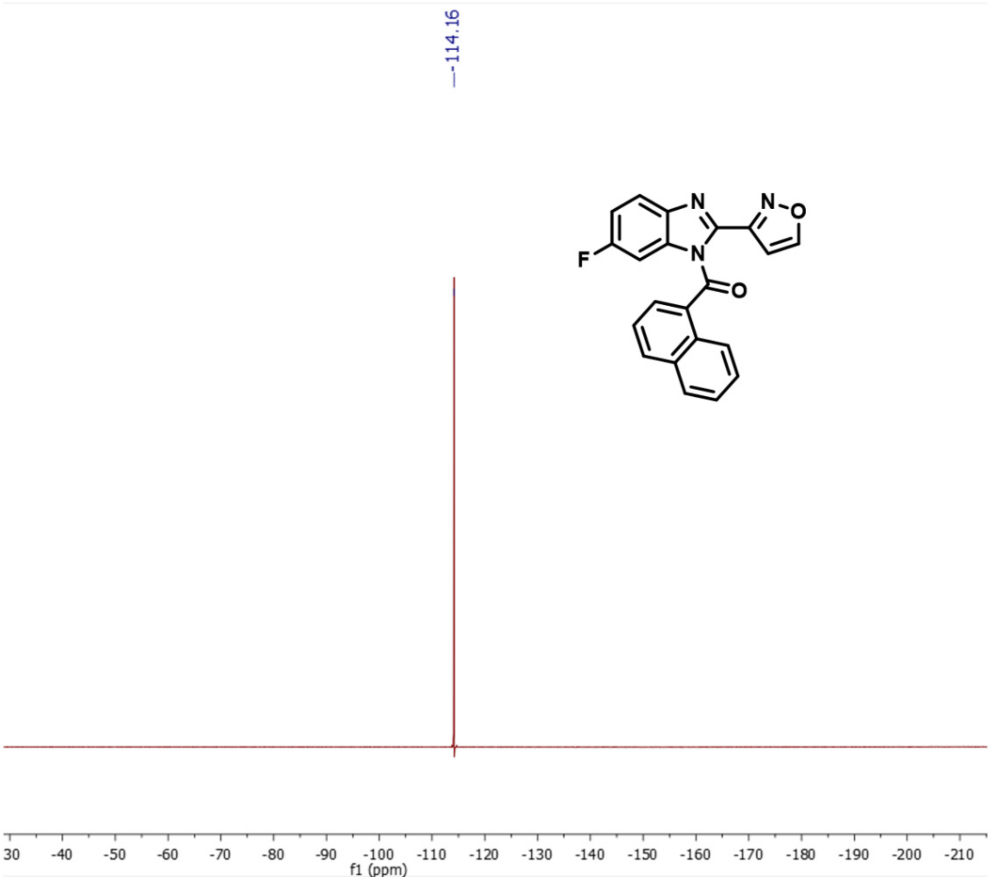

(5-fluoro-2-(5-methylfuran-2-yl)-1*H*-benzo[*d*]imidazol-1-yl)(naphthalen-1-yl)methanone (**5e**)

^{1}H NMR (300 MHz, DMSO-d^{6})

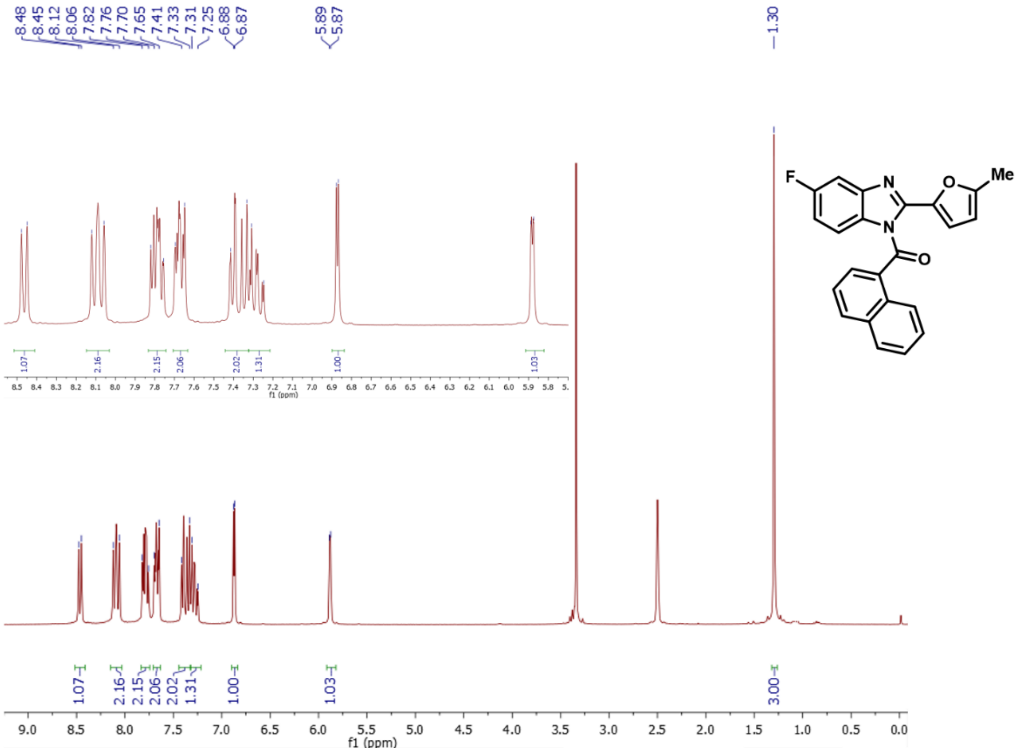

^{13}C NMR (75.5 MHz, DMSO-d^{6})

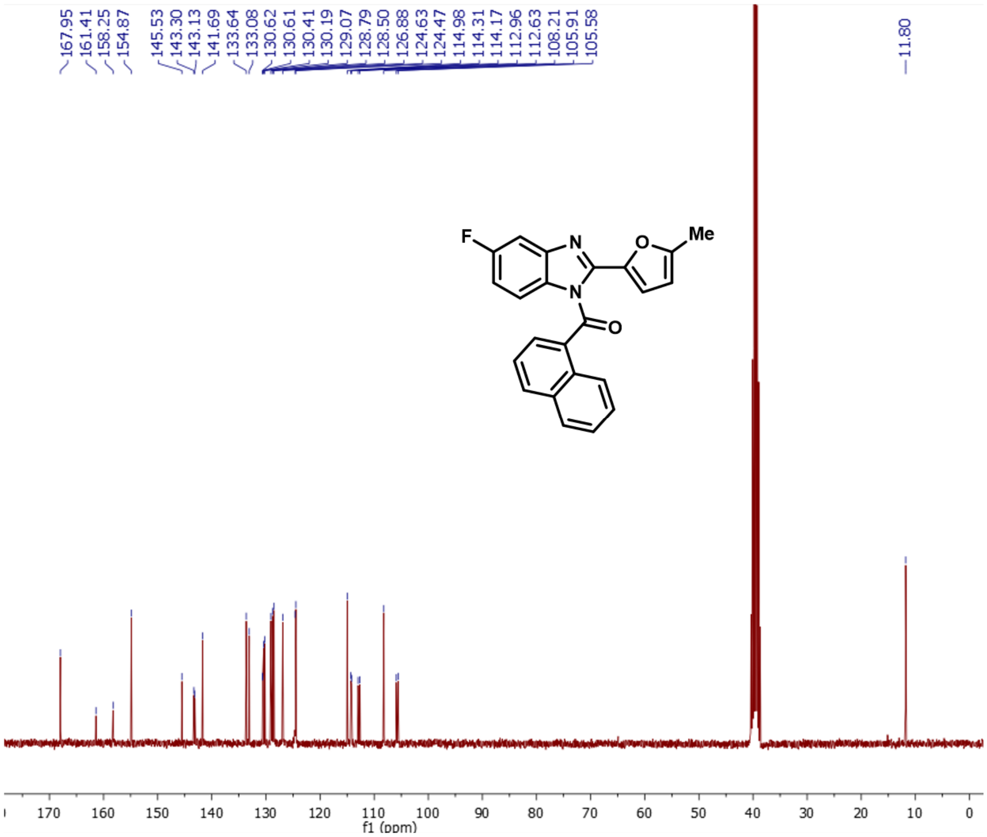

^{19}F NMR (282 MHz, DMSO-d^{6})

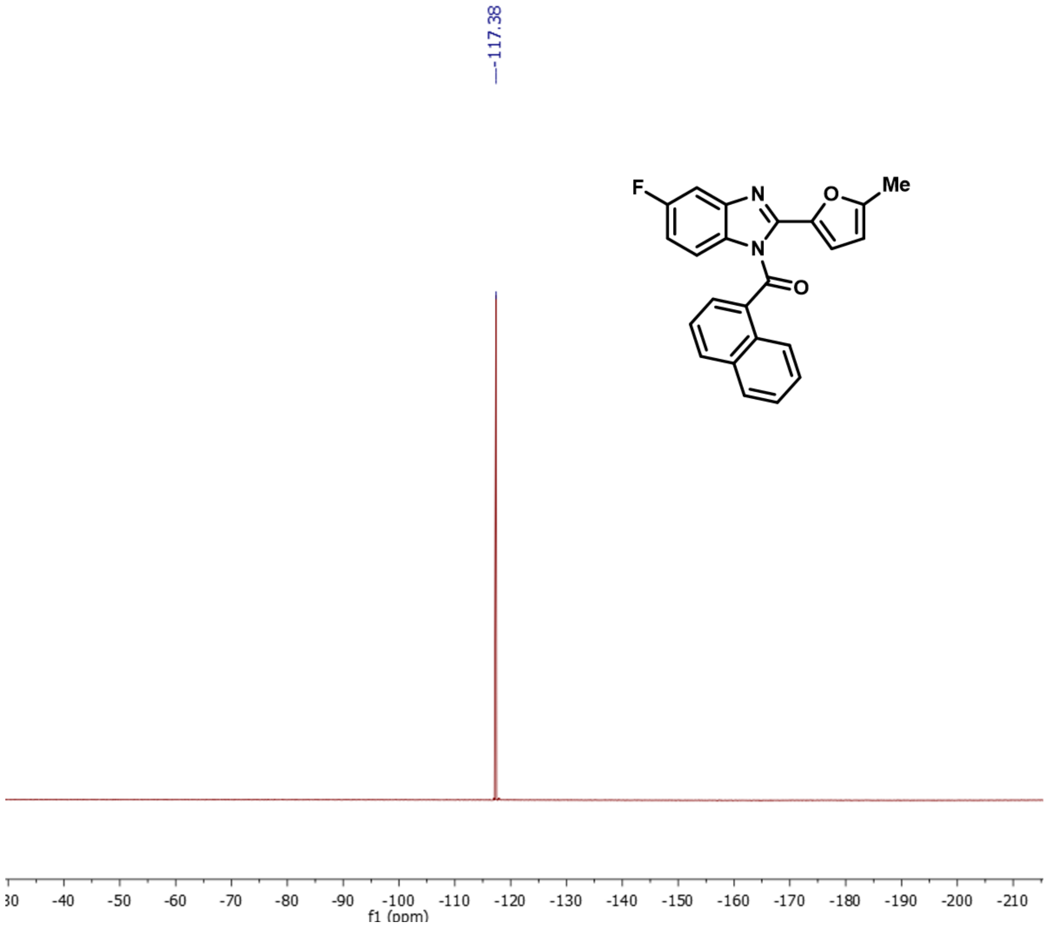

(6-fluoro-2-(5-methylfuran-2-yl)-1*H*-benzo[*d*]imidazol-1-yl)(naphthalen-1-yl)methanone (**5e’**)

^{1}H NMR (300 MHz, DMSO-d^{6})

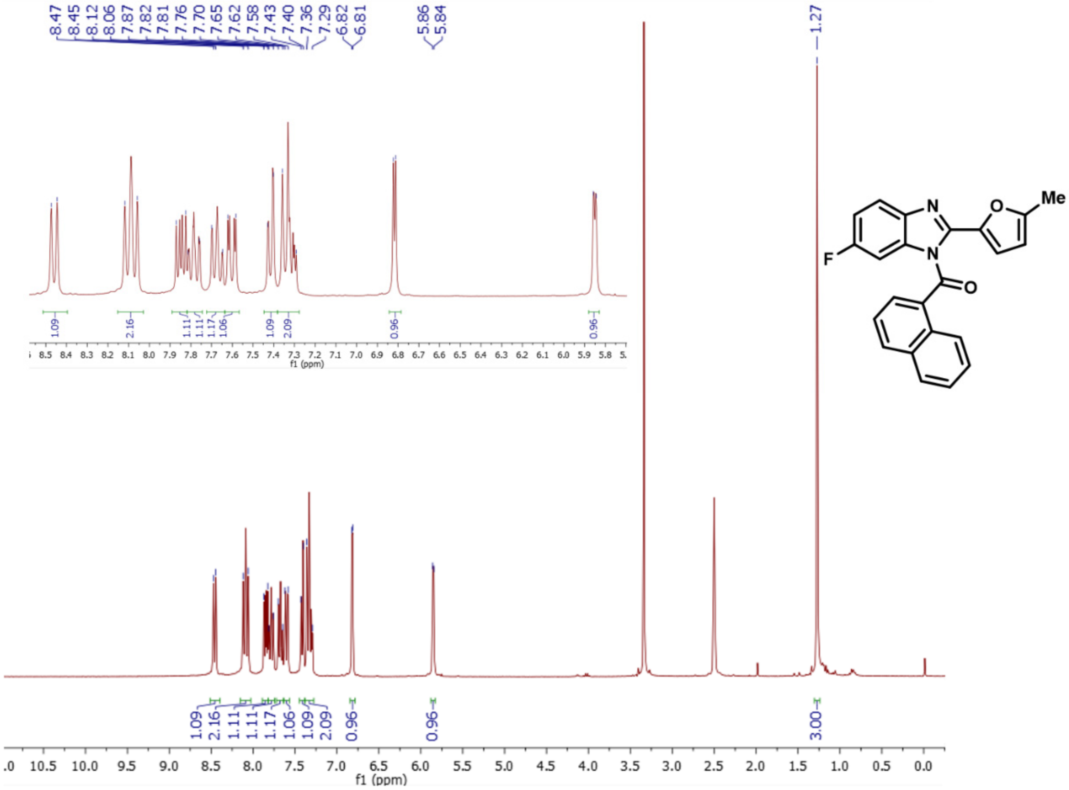

^{13}C NMR (75.5 MHz, DMSO-d^{6})

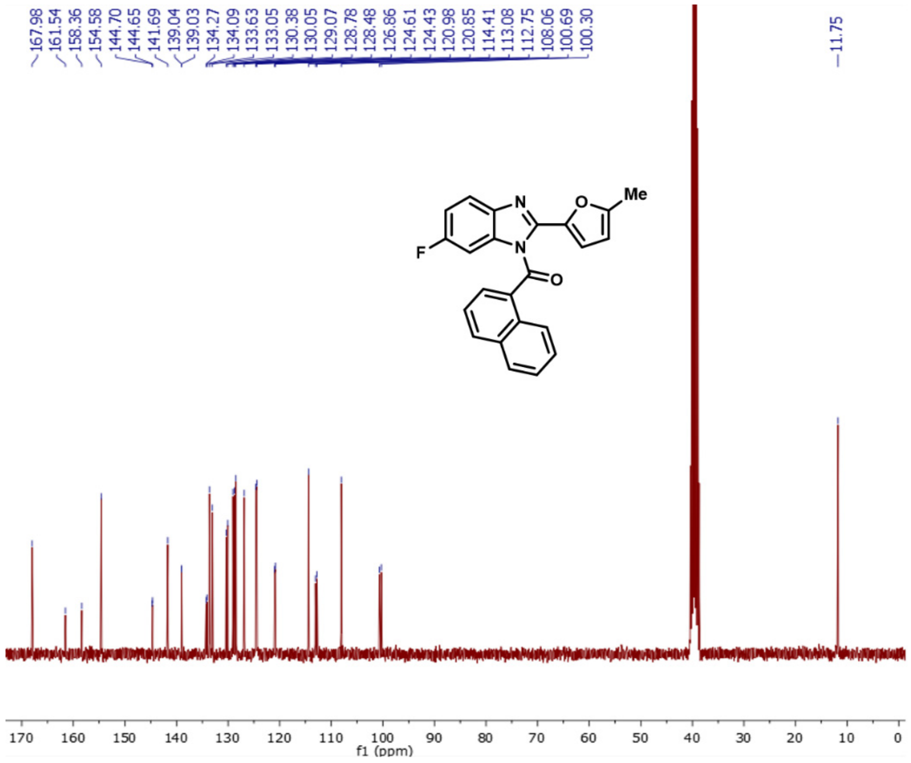

^{19}F NMR (282 MHz, DMSO-d^6)

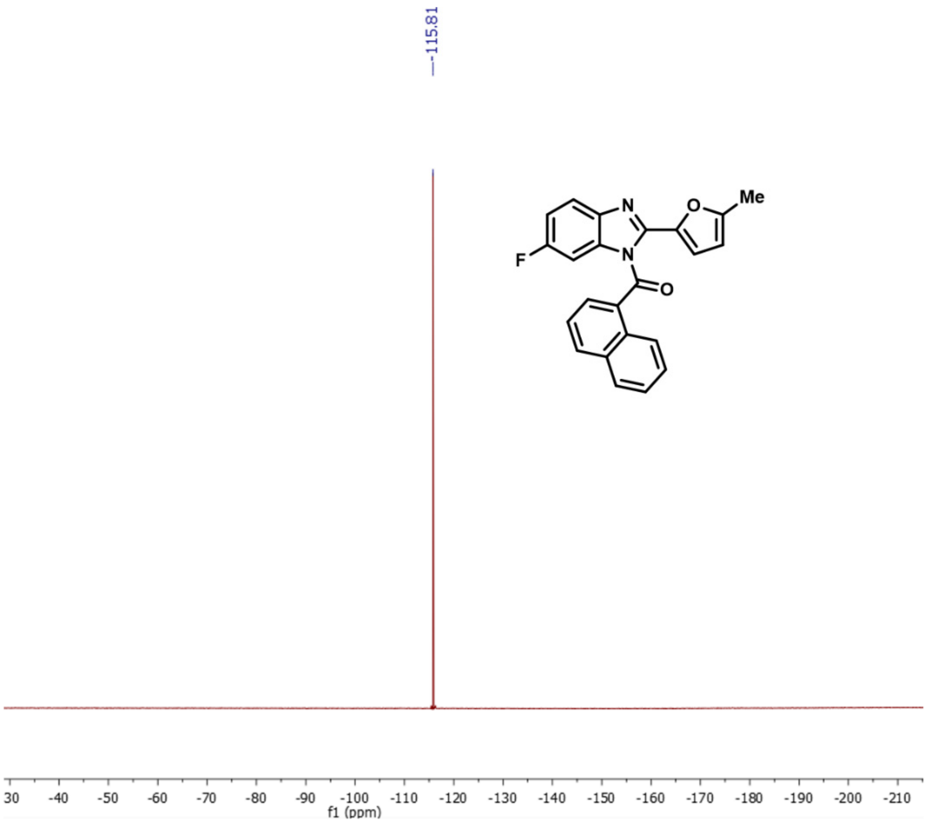

(5-fluoro-2-(furan-2-yl)-1*H*-benzo[*d*]imidazol-1-yl)(naphthalen-1-yl)methanone (**5f**)

^{1}H NMR (300 MHz, DMSO-d^{6})

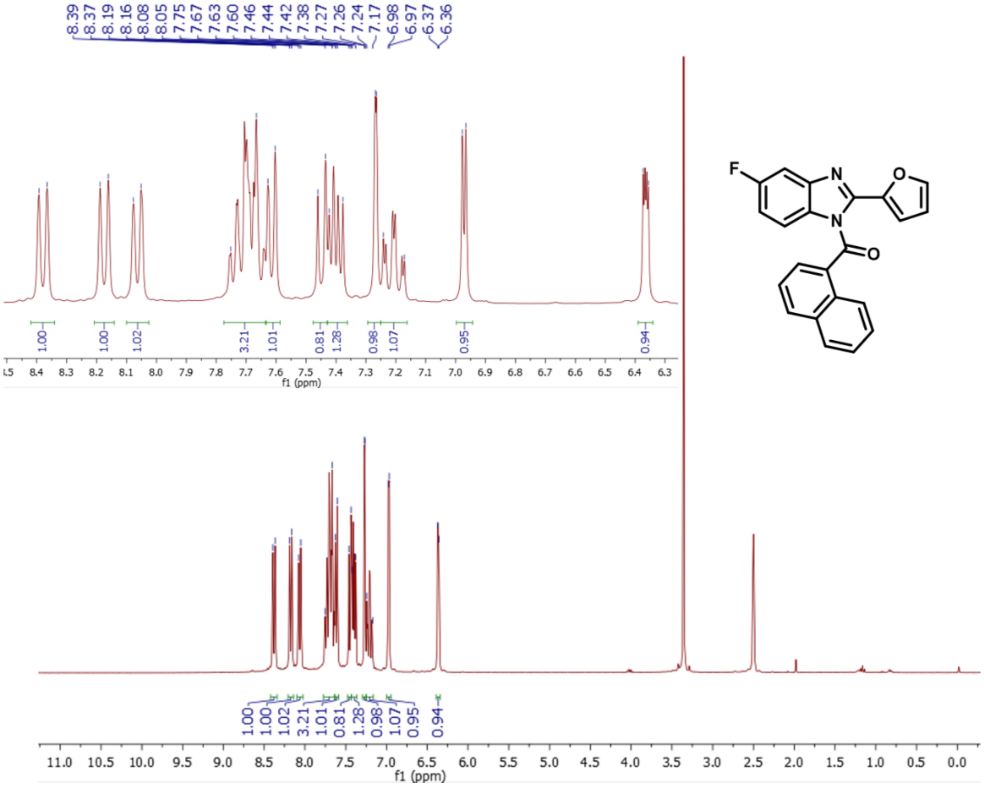

^{13}C NMR (75.5 MHz, DMSO-d^{6})

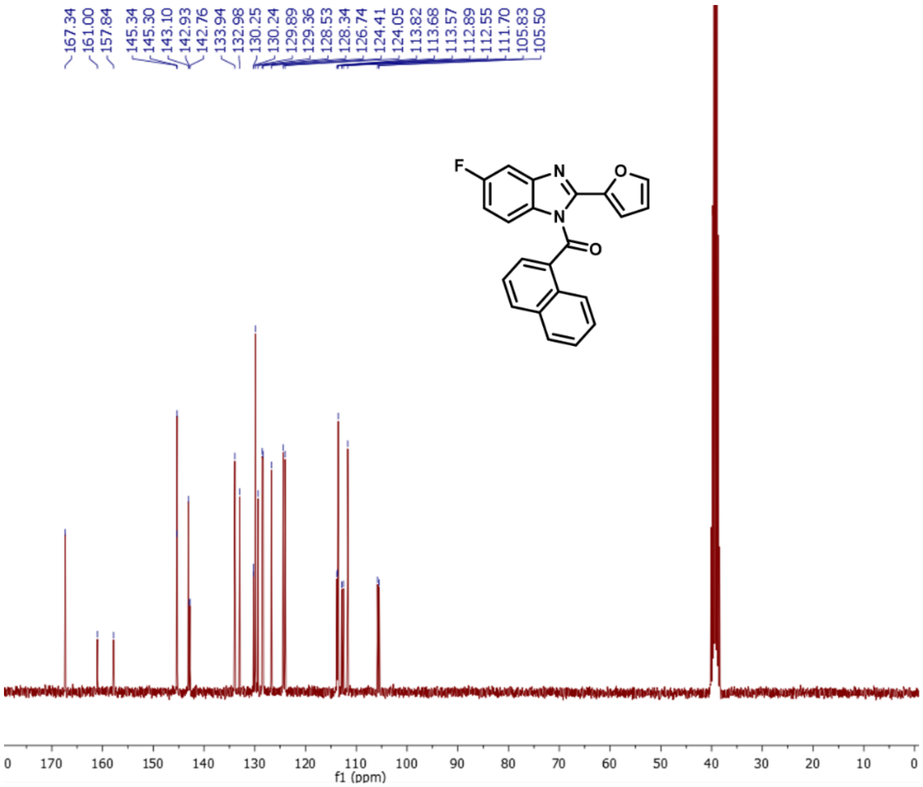

^{19}F NMR (282 MHz, DMSO-d^6)

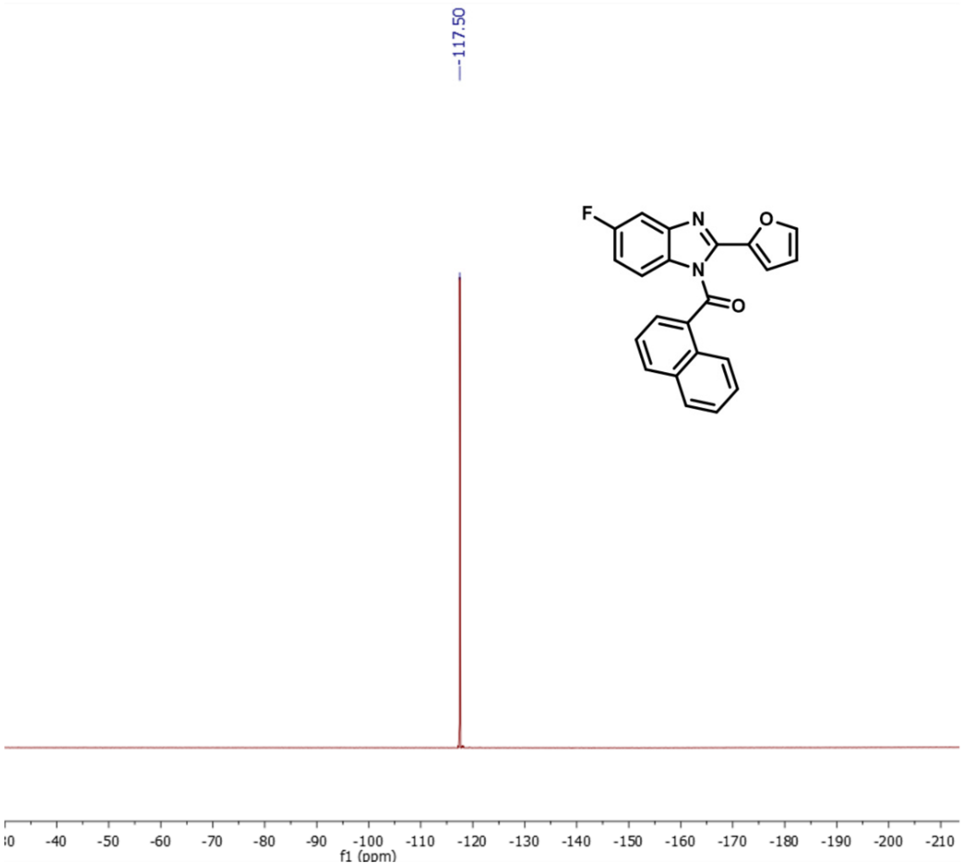

(6-fluoro-2-(furan-2-yl)-1*H*-benzo[*d*]imidazol-1-yl)(naphthalen-1-yl)methanone (**5f’**)

^{1}H NMR (300 MHz, DMSO-d^{6})

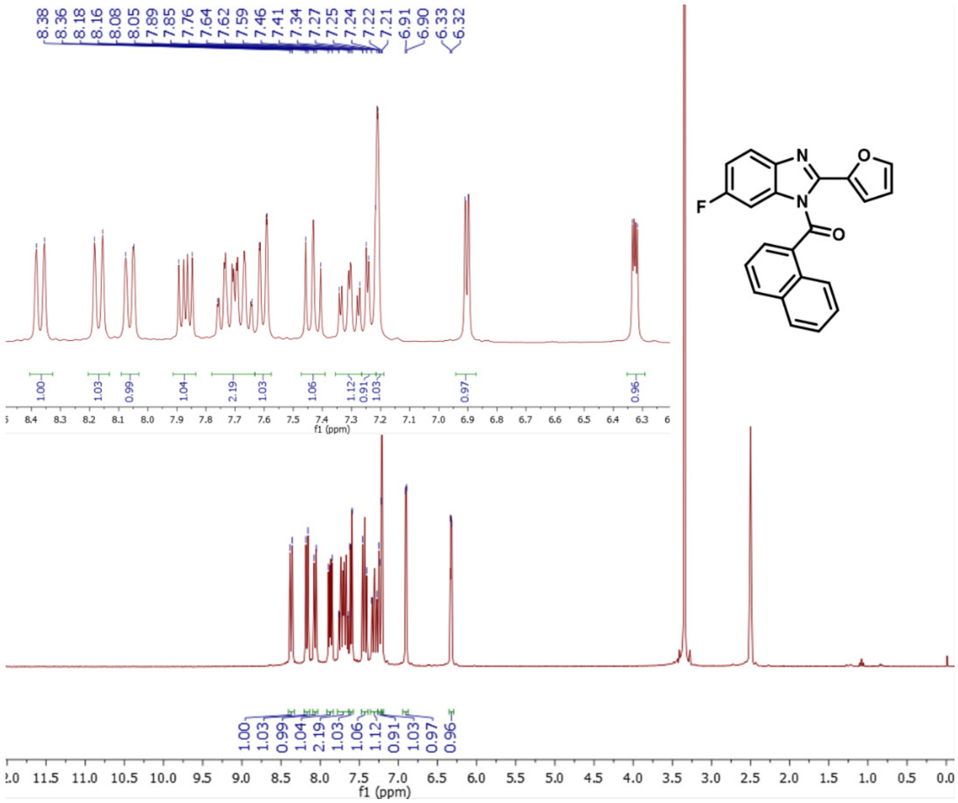

^{13}C NMR (75.5 MHz, DMSO-d^{6})

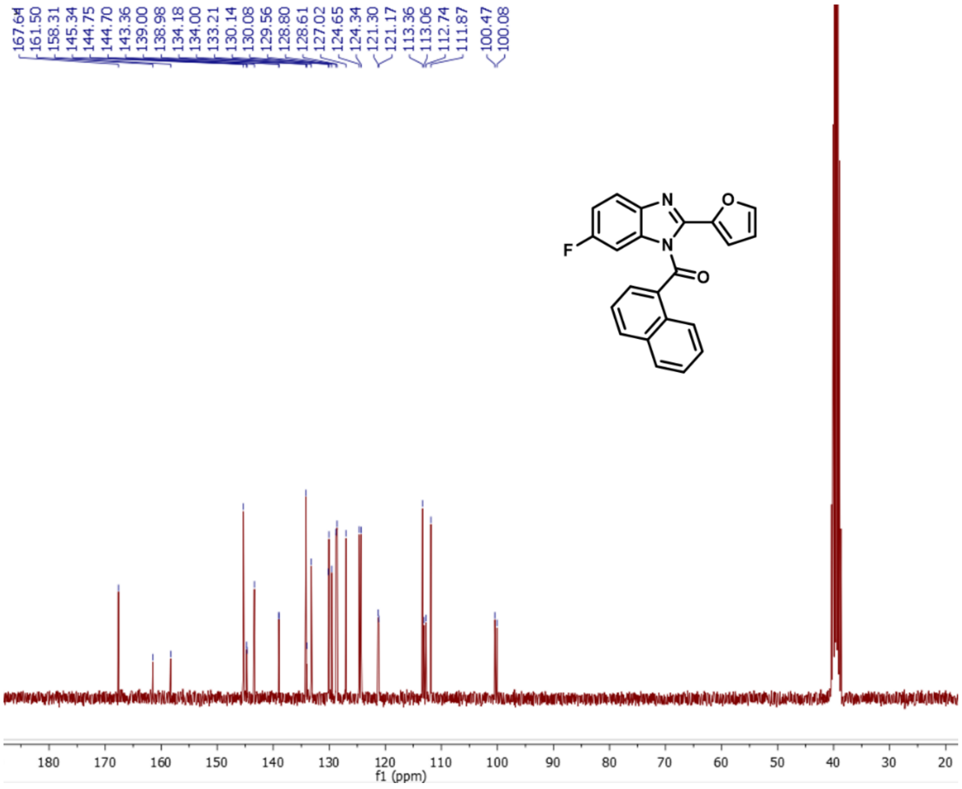

^{19}F NMR (282 MHz, DMSO-d^6)

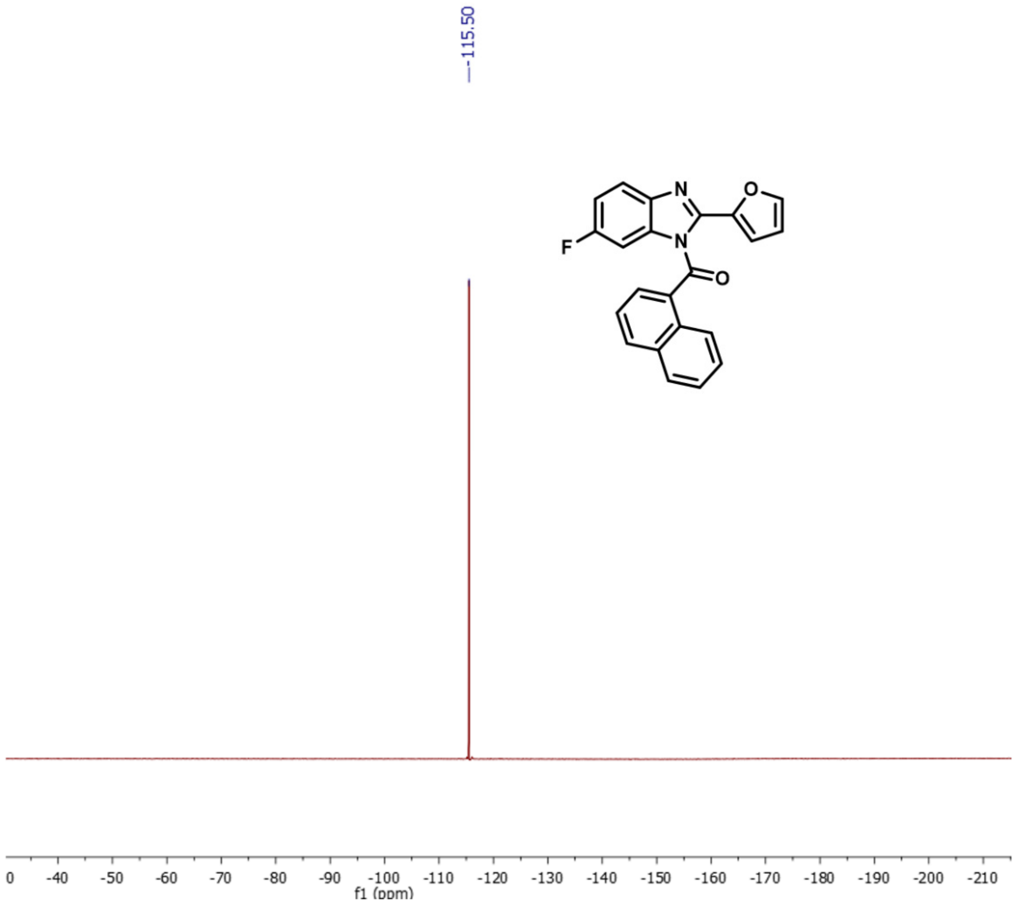

FT-IR spectra of compounds

(5-chloro-2-(pyridin-3-yl)-1*H*-benzo[*d*]imidazol-1-yl)(naphthalen-1-yl)methanone (**4a**)

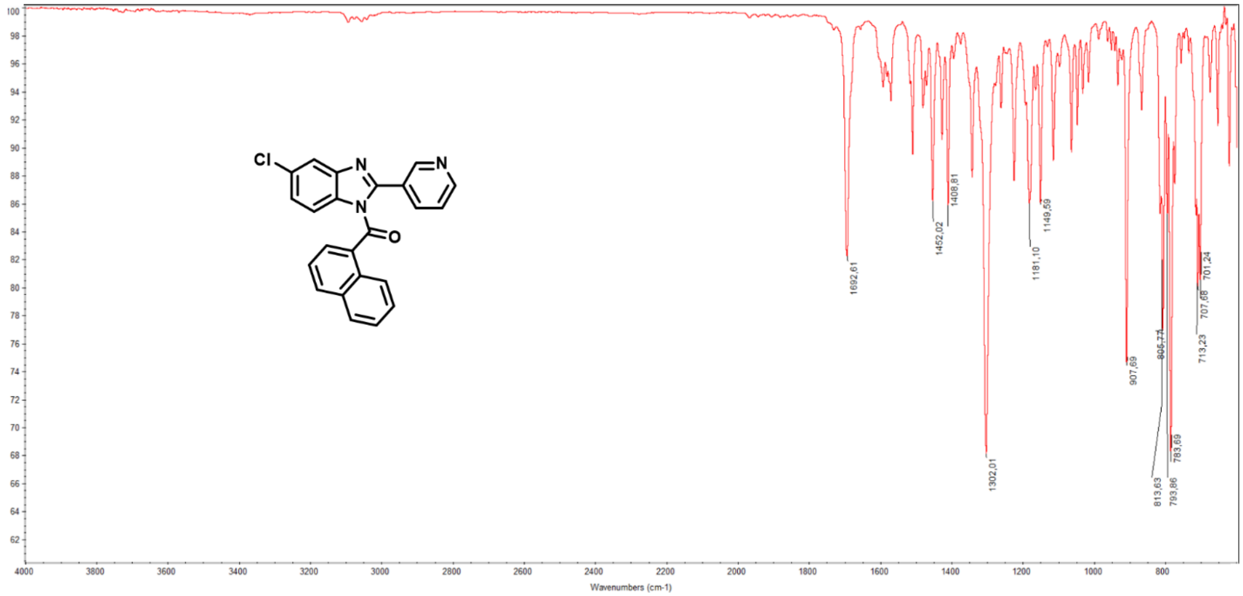

(6-chloro-2-(pyridin-3-yl)-1H-benzo[*d*]imidazol-1-yl)(naphthalen-1-yl)methanone (**4a'**)

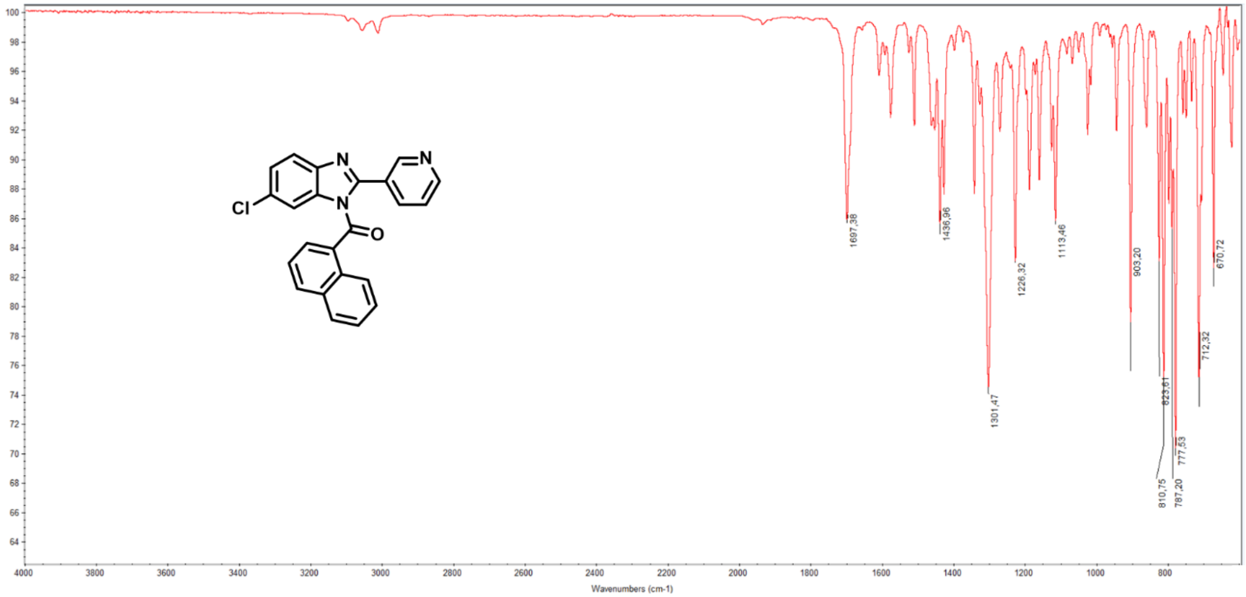

(5-chloro-2-(3-methoxyphenyl)-1*H*-benzo[*d*]imidazol-1-yl)(naphthalen-1-yl)methanone (**4b**)

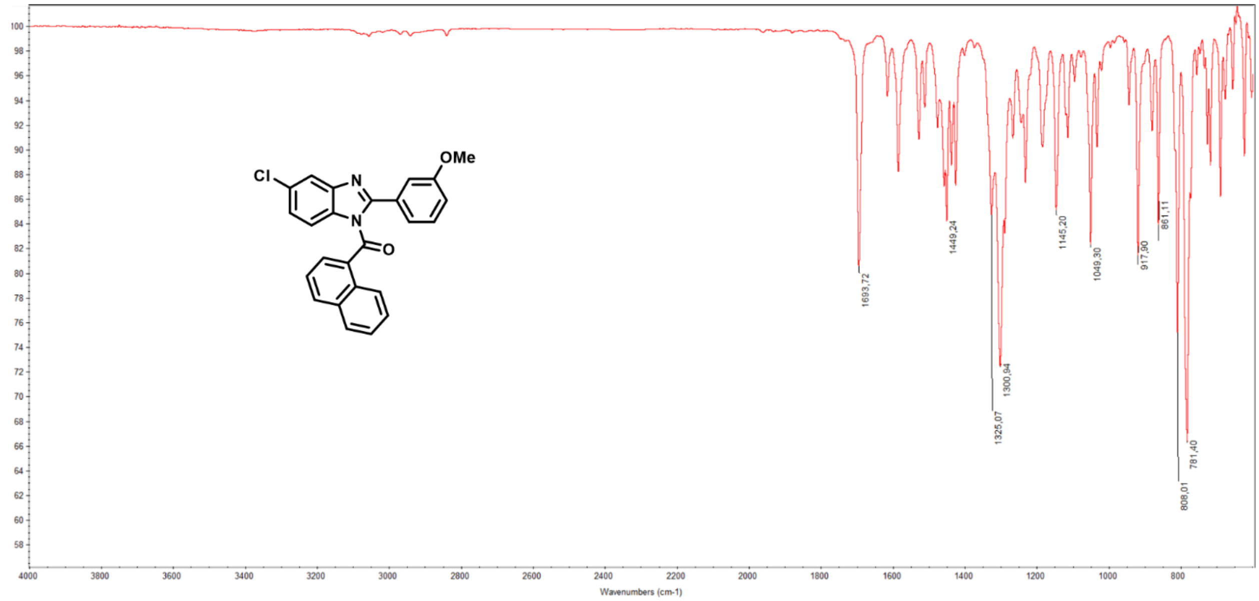

(6-chloro-2-(3-methoxyphenyl)-1*H*-benzo[*d*]imidazol-1-yl)(naphthalen-1-yl)methanone (**4b’**)

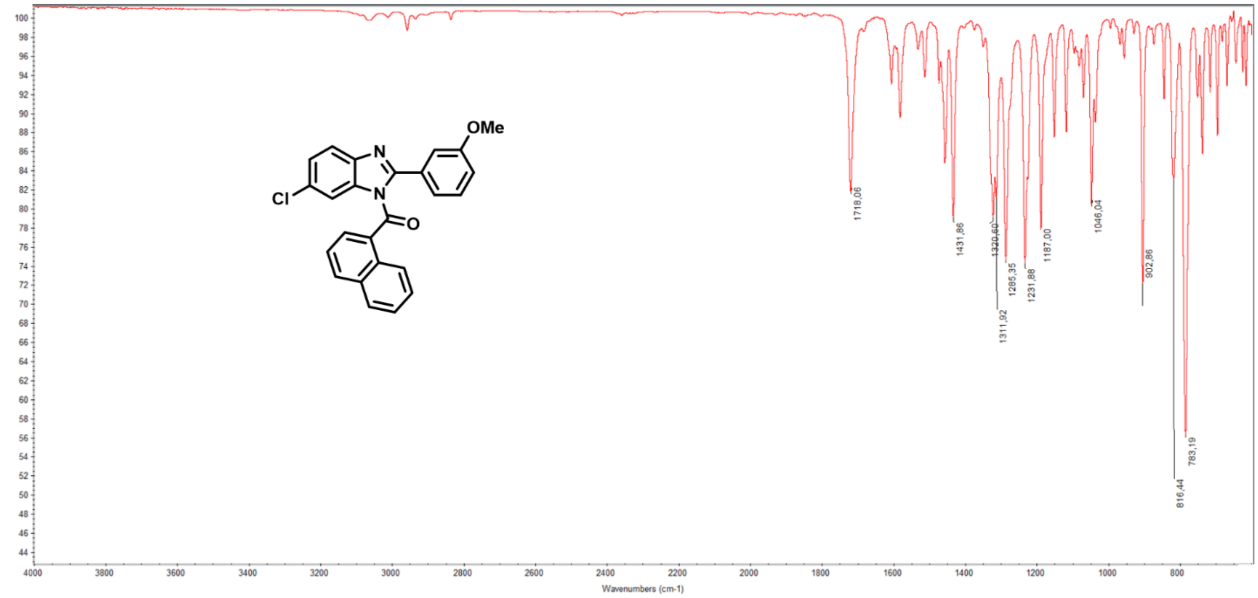

(5-chloro-2-(5-methylisoxazol-3-yl)-1*H*-benzo[*d*]imidazol-1-yl)(naphthalen-1-yl)methanone (**4c**)

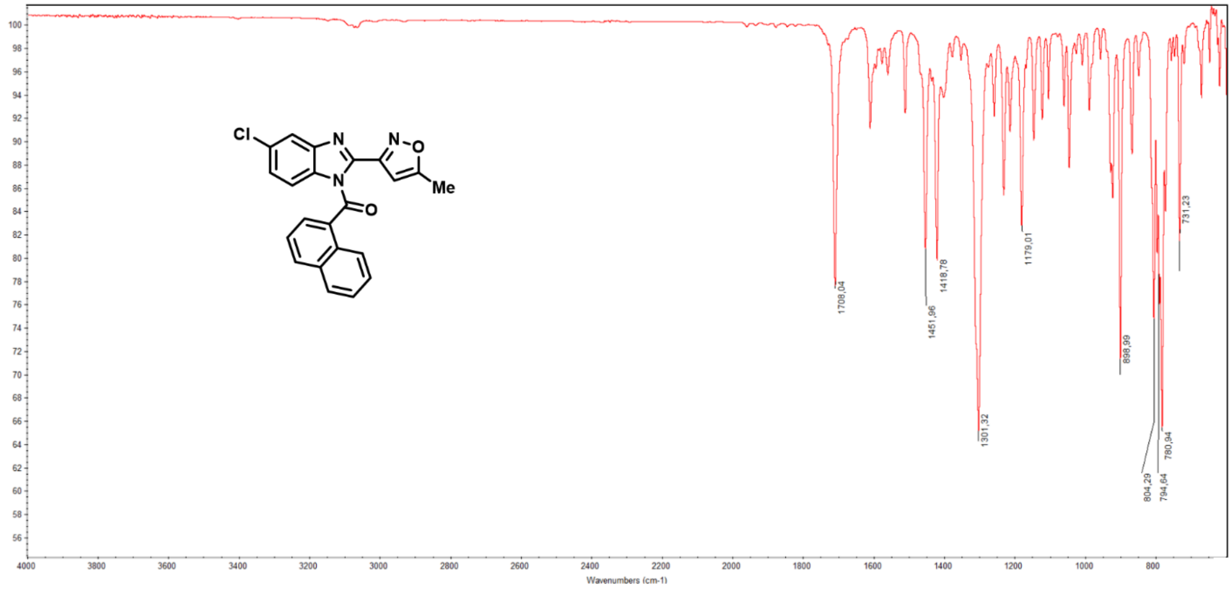

(6-chloro-2-(5-methylisoxazol-3-yl)-1*H*-benzo[*d*]imidazol-1-yl)(naphthalen-1-yl)methanone (**4c'**)

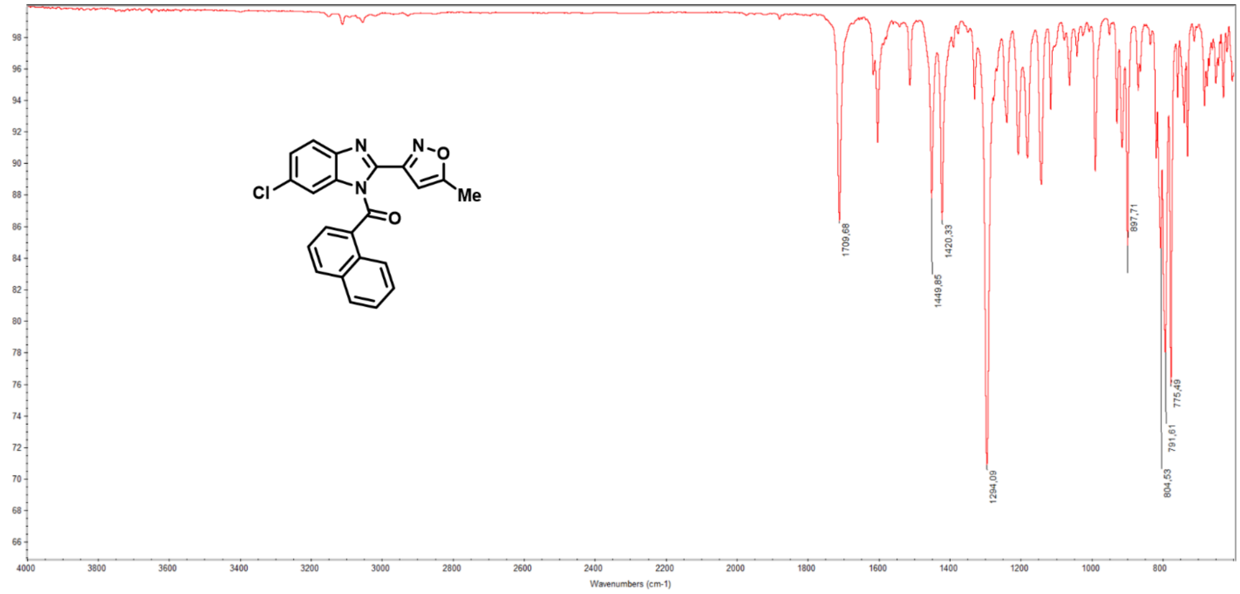

(5-chloro-2-(isoxazol-3-yl)-1*H*-benzo[*d*]imidazol-1-yl)(naphthalen-1-yl)methanone (**4d**)

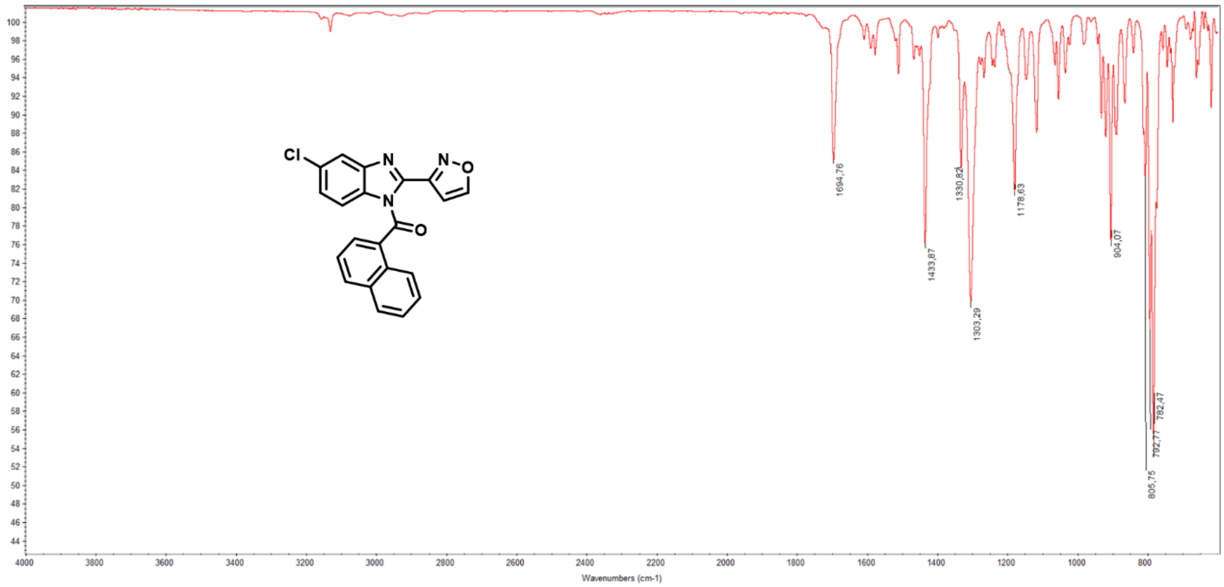

(6-chloro-2-(isoxazol-3-yl)-1*H*-benzo[*d*]imidazol-1-yl)(naphthalen-1-yl)methanone (**4d'**)

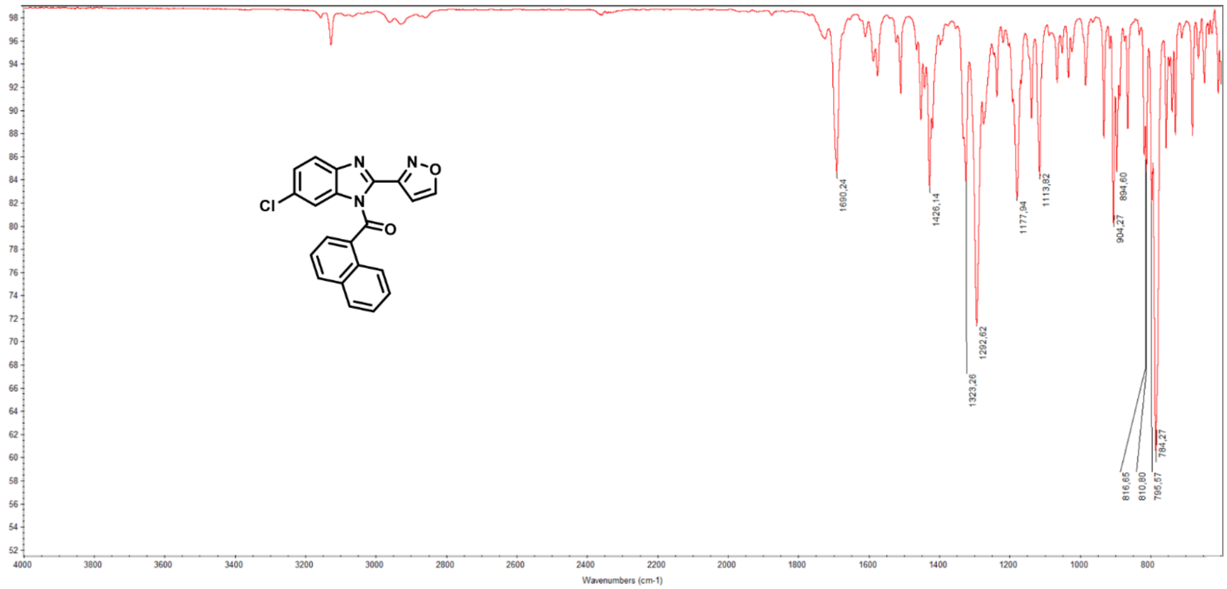

(5-chloro-2-(5-methylfuran-2-yl)-1*H*-benzo[*d*]imidazol-1-yl)(naphthalen-1-yl)methanone (**4e**)

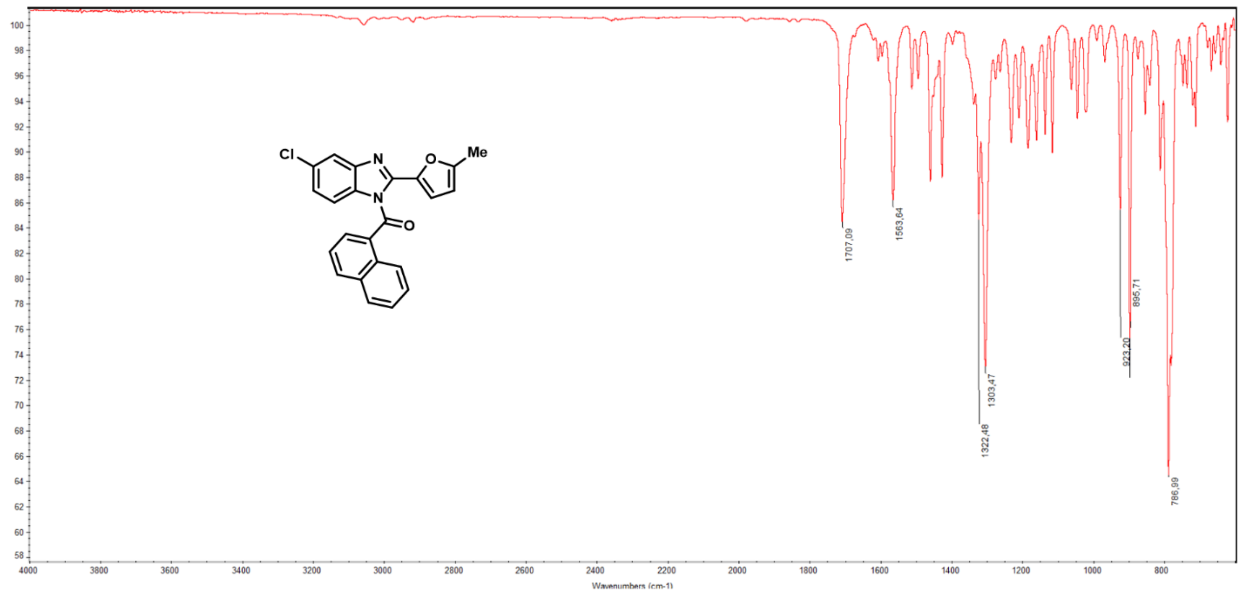

(6-chloro-2-(5-methylfuran-2-yl)-1*H*-benzo[*d*]imidazol-1-yl)(naphthalen-1-yl)methanone (**4e'**)

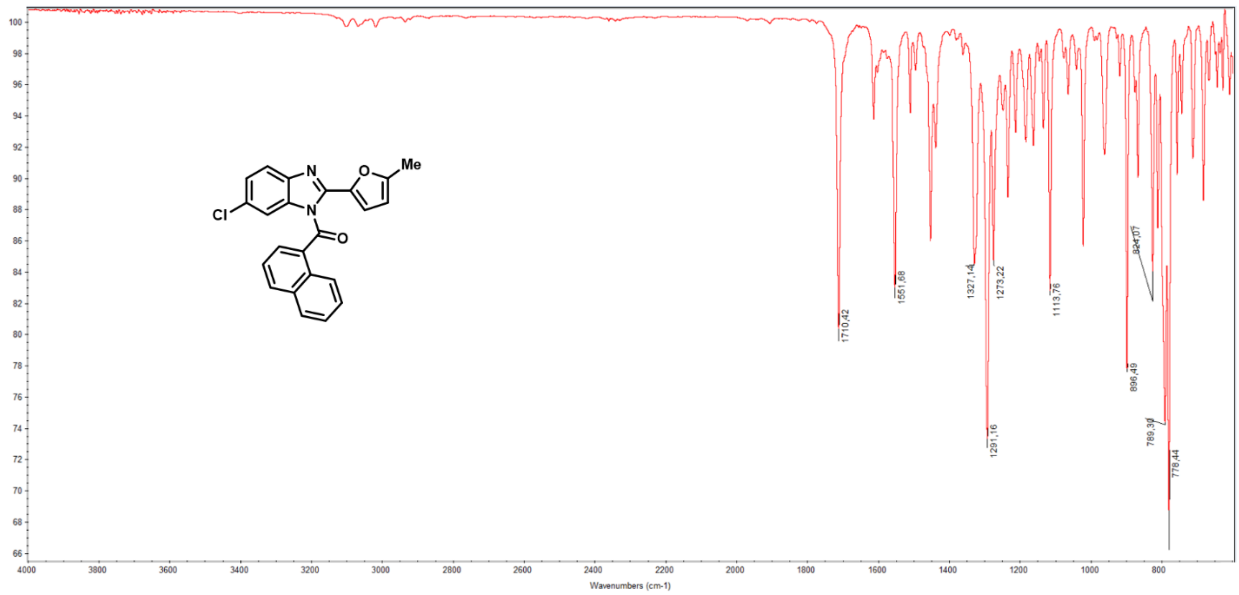

(5-chloro-2-(furan-2-yl)-1*H*-benzo[*d*]imidazol-1-yl)(naphthalen-1-yl)methanone (**4f**)

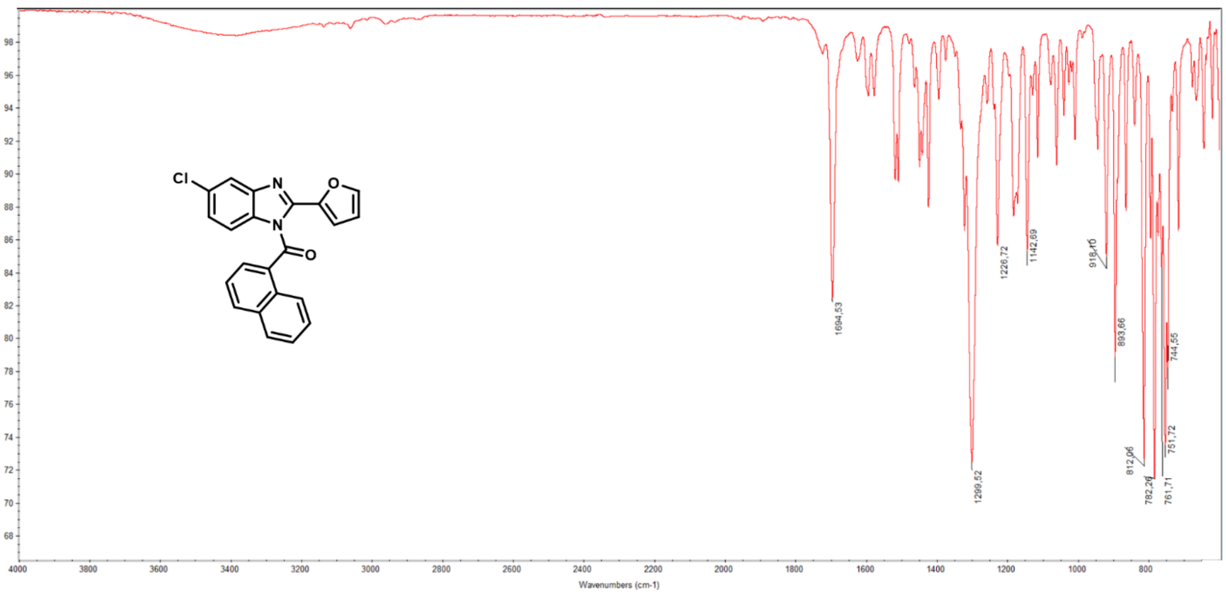

(6-chloro-2-(furan-2-yl)-1*H*-benzo[*d*]imidazol-1-yl)(naphthalen-1-yl)methanone (**4f'**)

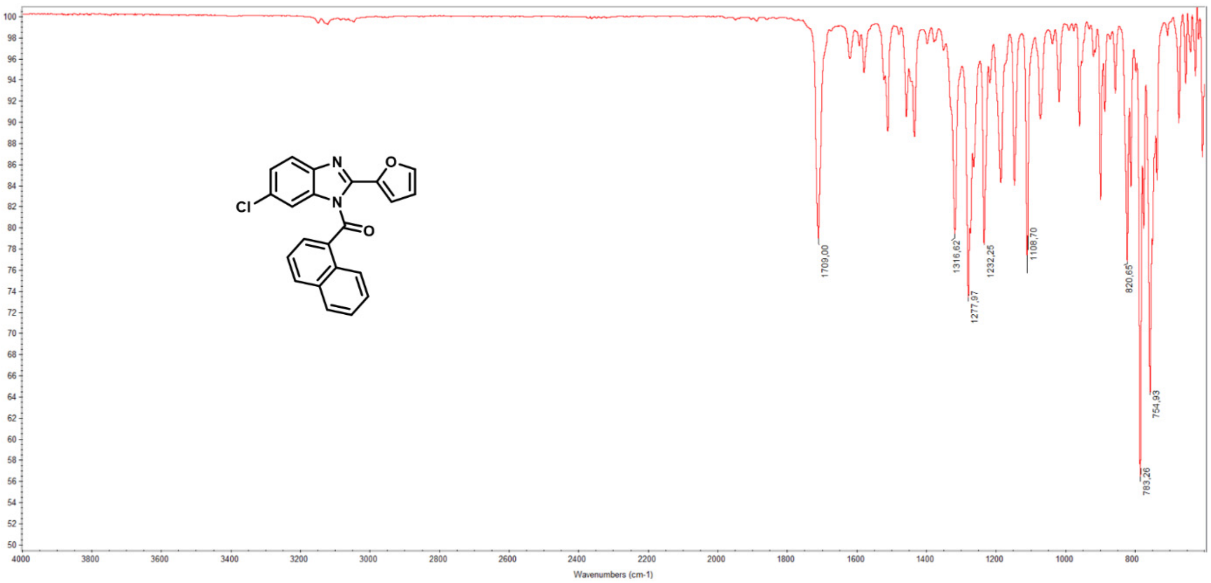

(5-fluoro-2-(pyridin-3-yl)-1*H*-benzo[*d*]imidazol-1-yl)(naphthalen-1-yl)methanone (**5a**)

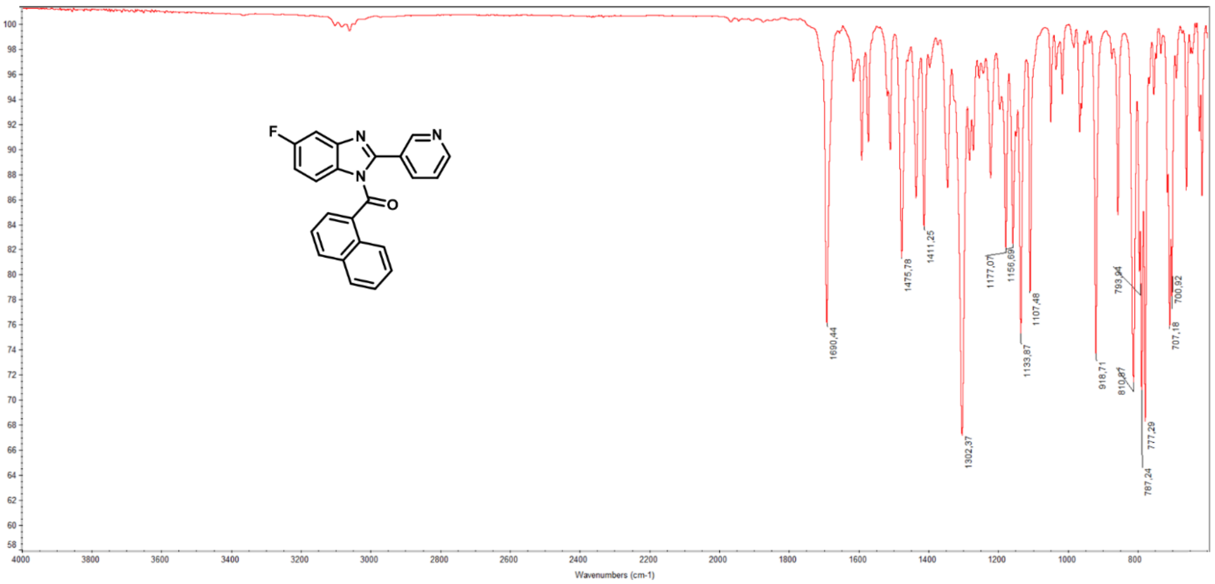

(6-fluoro-2-(pyridin-3-yl)-1*H*-benzo[*d*]imidazol-1-yl)(naphthalen-1-yl)methanone (**5a'**)

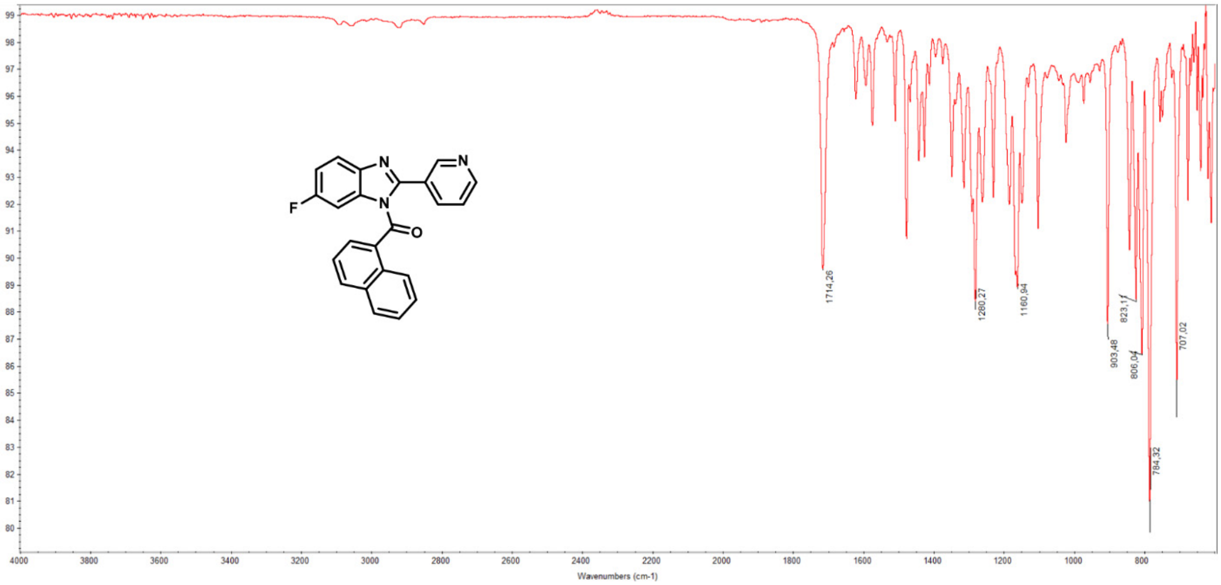

(5-fluoro-2-(3-methoxyphenyl)-1*H*-benzo[*d*]imidazol-1-yl)(naphthalen-1-yl)methanone (**5b**)

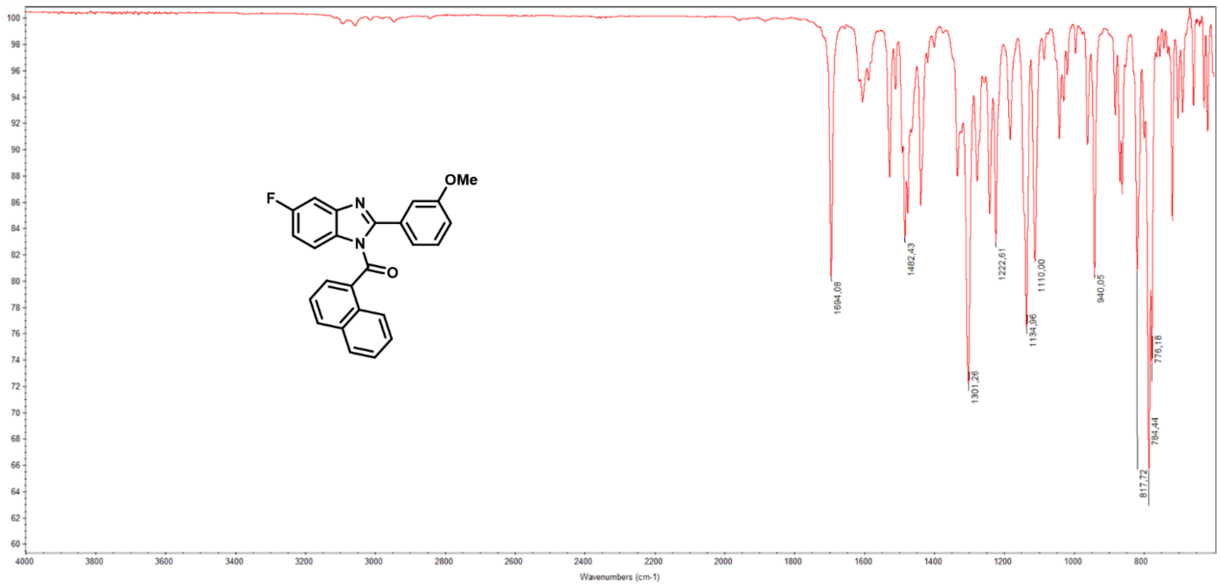

(6-fluoro-2-(3-methoxyphenyl)-1*H*-benzo[*d*]imidazol-1-yl)(naphthalen-1-yl)methanone (**5b’**)

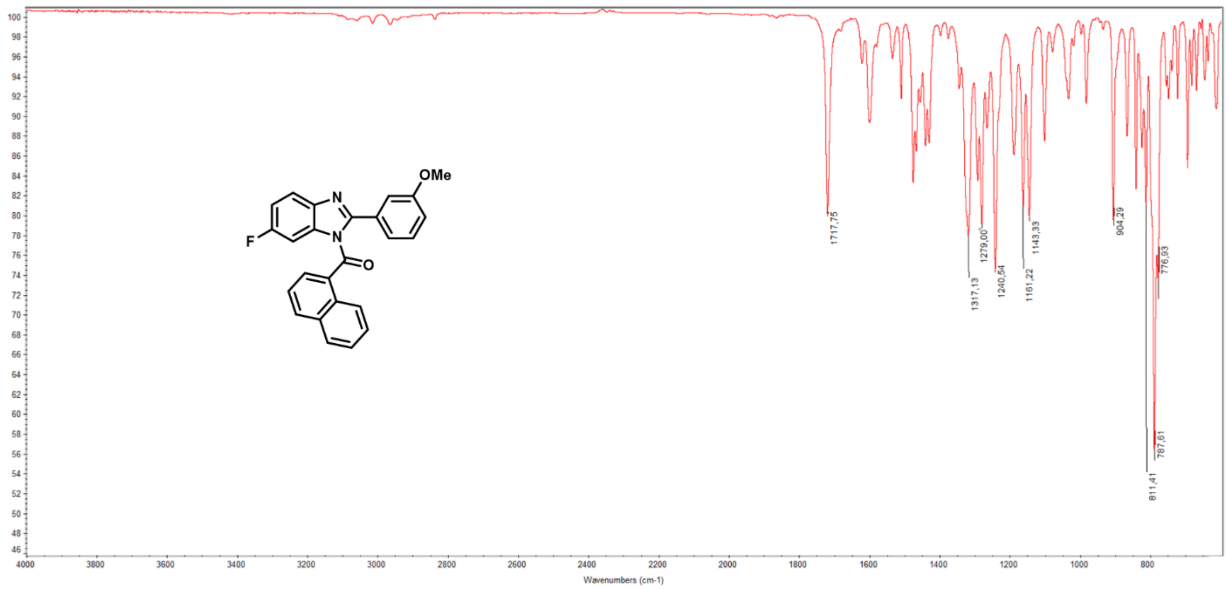

(5-fluoro-2-(5-methylisoxazol-3-yl)-1*H*-benzo[*d*]imidazol-1-yl)(naphthalen-1-yl)methanone (**5c**)

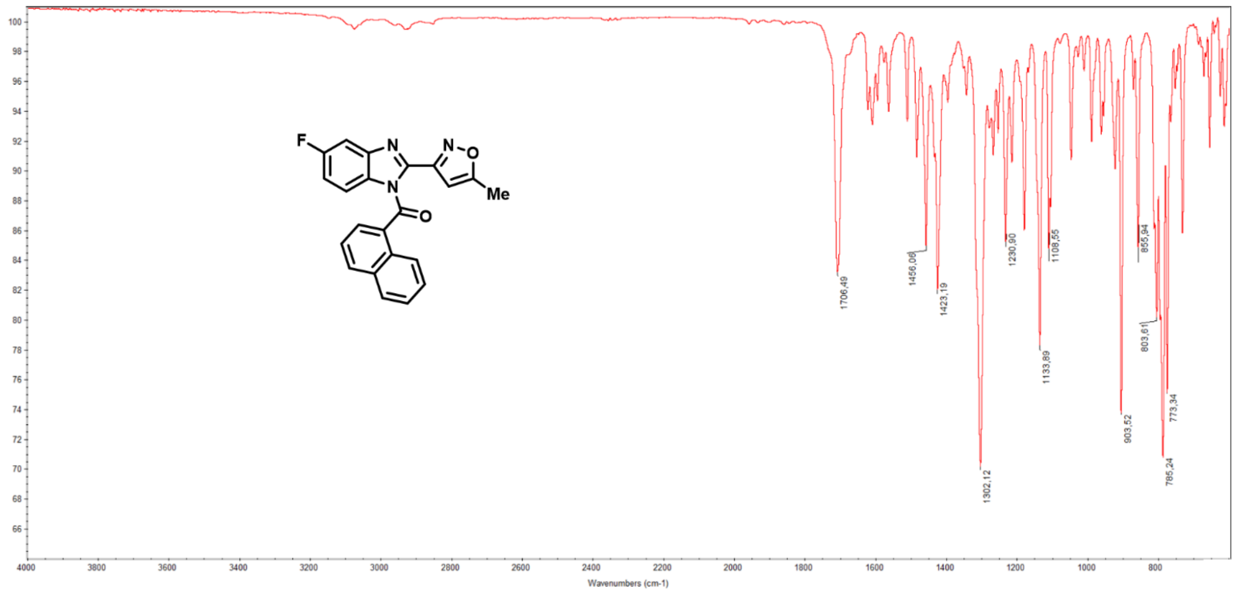

(6-fluoro-2-(5-methylisoxazol-3-yl)-1*H*-benzo[*d*]imidazol-1-yl)(naphthalen-1-yl)methanone (**5c'**)

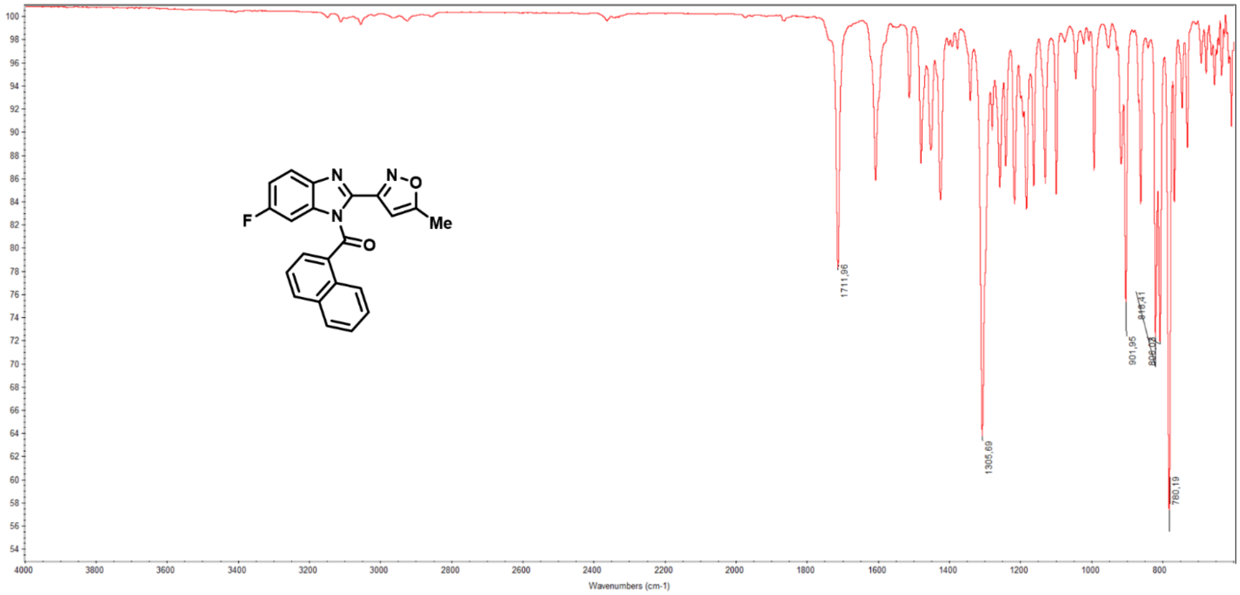

(5-fluoro-2-(isoxazol-3-yl)-1*H*-benzo[*d*]imidazol-1-yl)(naphthalen-1-yl)methanone (**5d**)

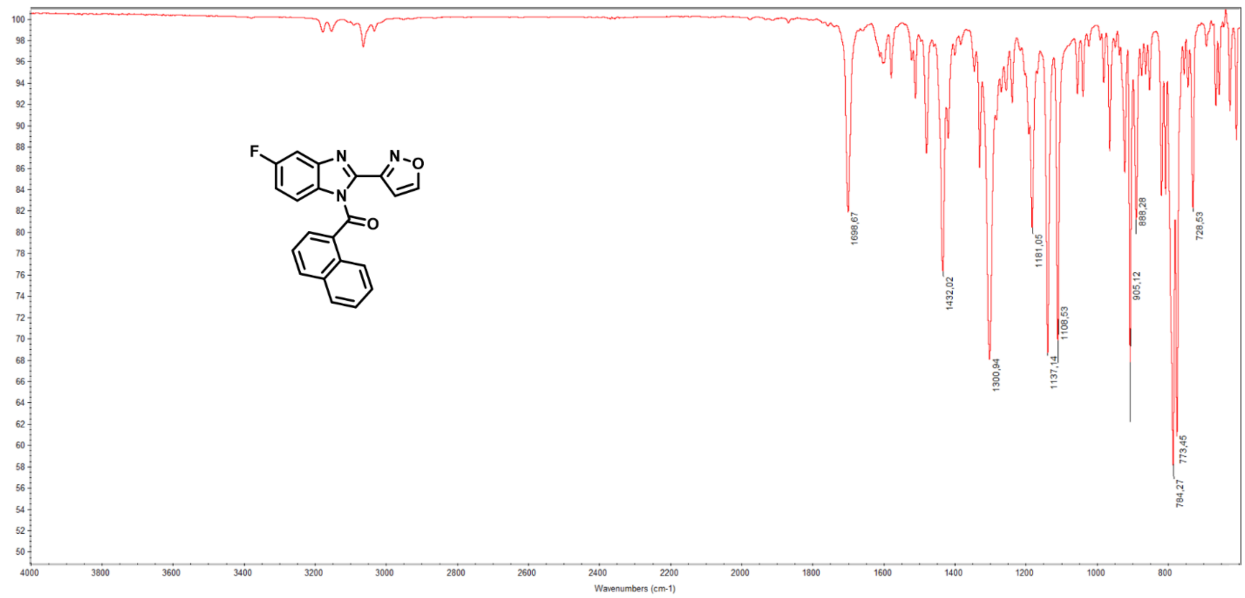

(6-fluoro-2-(isoxazol-3-yl)-1*H*-benzo[*d*]imidazol-1-yl)(naphthalen-1-yl)methanone (**5d'**)

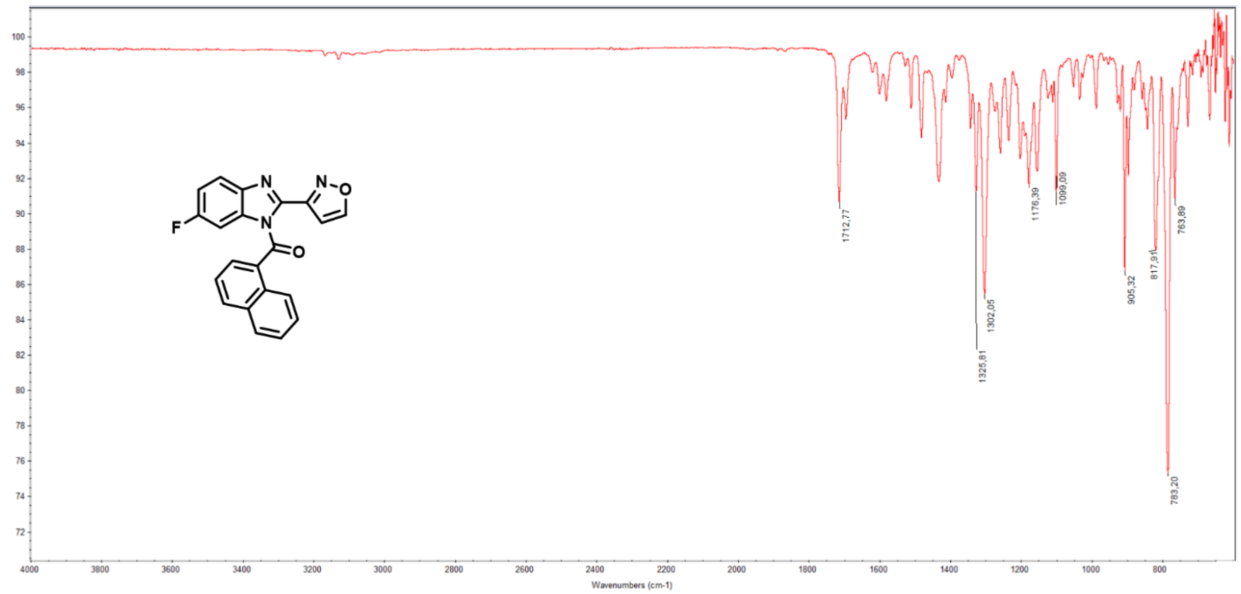

(5-fluoro-2-(5-methylfuran-2-yl)-1*H*-benzo[*d*]imidazol-1-yl)(naphthalen-1-yl)methanone (**5e**)

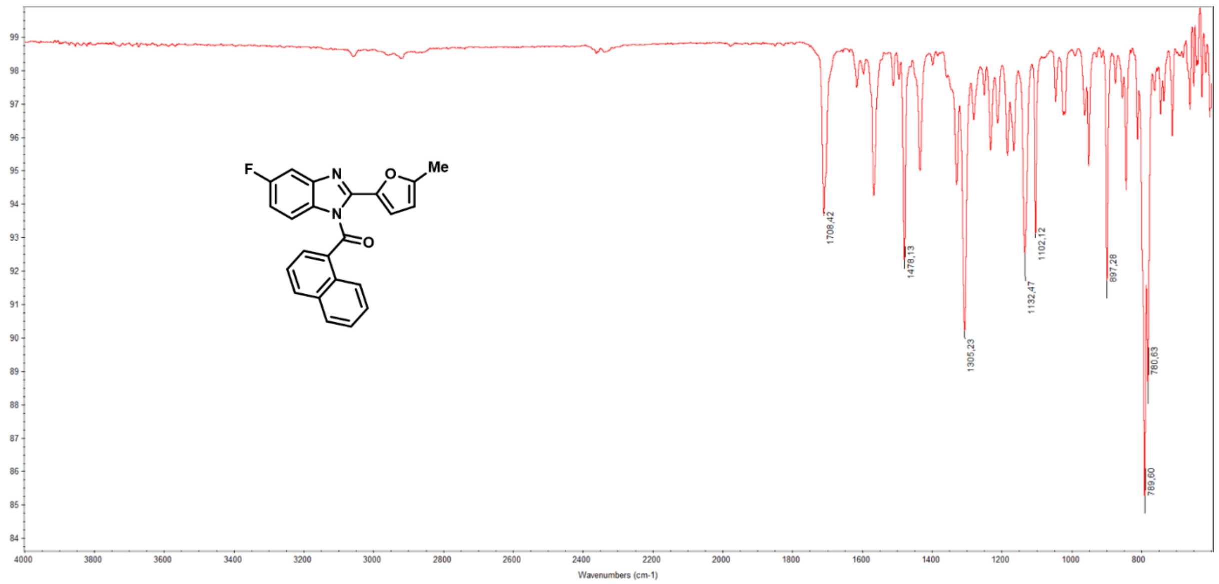

(6-fluoro-2-(5-methylfuran-2-yl)-1*H*-benzo[*d*]imidazol-1-yl)(naphthalen-1-yl)methanone (**5e'**)

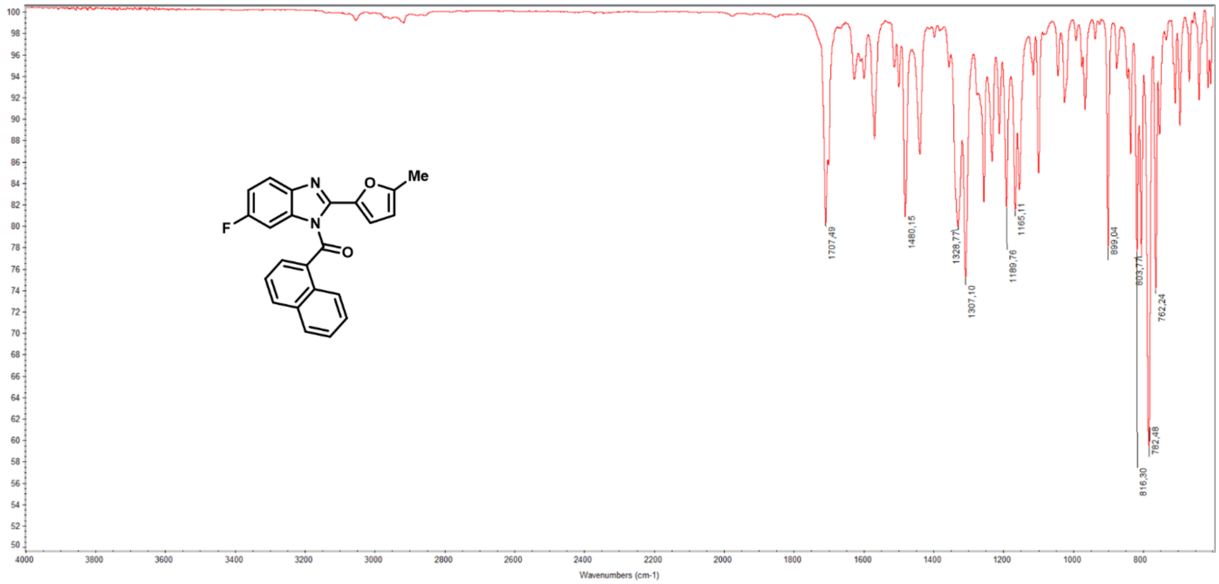

(5-fluoro-2-(furan-2-yl)-1*H*-benzo[*d*]imidazol-1-yl)(naphthalen-1-yl)methanone (**5f**)

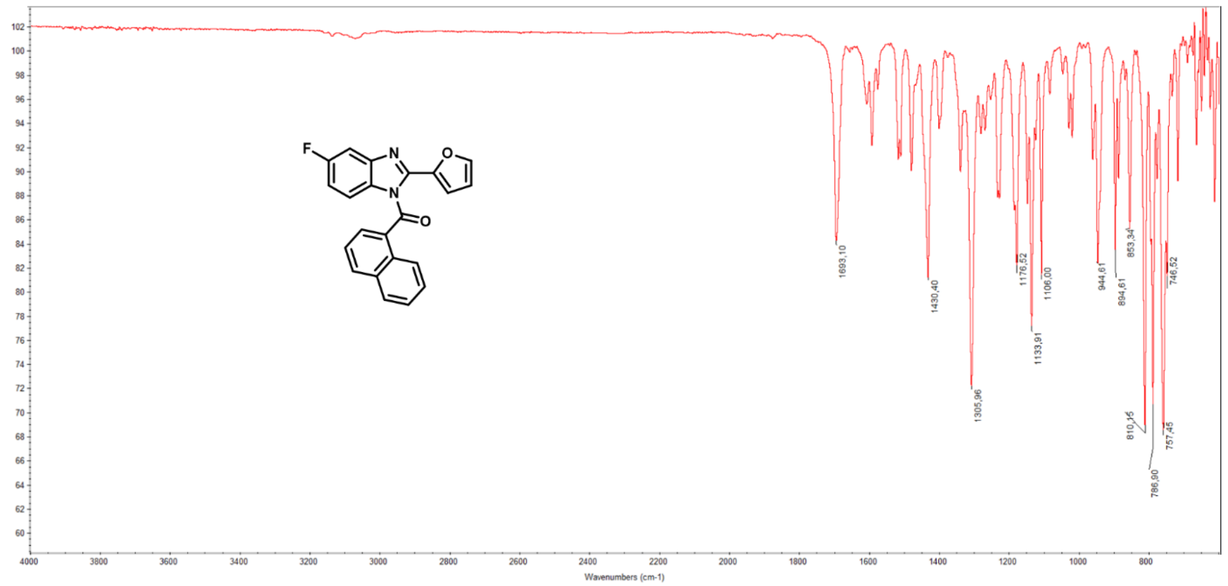

(6-fluoro-2-(furan-2-yl)-1*H*-benzo[*d*]imidazol-1-yl)(naphthalen-1-yl)methanone (**5f’**)

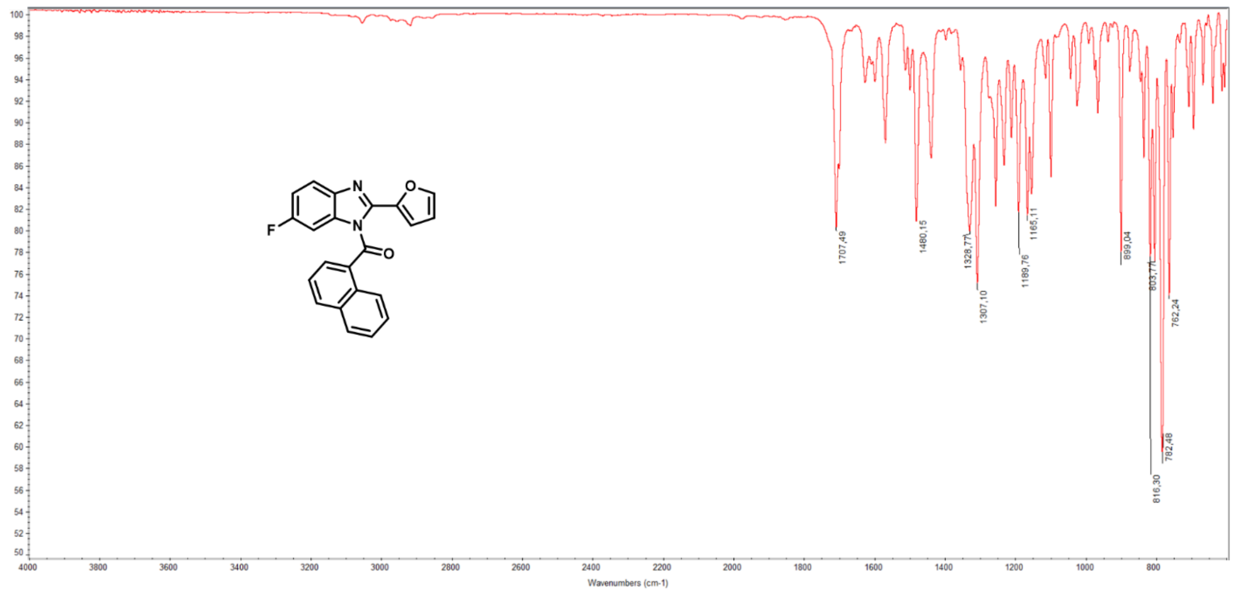

Biological experiments

Binding assays CB1/CB2 (radioligand displacement)

For CB_1 and CB_2 receptor binding assays, the compounds were subjected to a screening using three different concentrations (0.1 mM, 1.0 mM and 10 µM) of the synthesized compounds; HEK-293 cell membranes overexpressing either the human cannabinoid CB_1 receptor –hCB1 HEK293-EBNA membranes– (Perkin Elmer Inc., Singapore Pte. Ltd. Product No.: RBHCB1M400UA), or the human recombinant cannabinoid CB_2 receptor –hCB2 HEK293-EBNA Membranes– (Perkin Elmer Inc., Singapore Pte. Ltd. Product No.: RBXCB2M400UA,); and $[^3H]$-(-)cis-3-[2-hydroxy-4-(1,1-dimethylheptyl)phenyl]-*trans*-4-(3-hydroxypropyl)cyclohexanol ($[^3H]$CP-55,940; Kd for CB_1 = 0.16 nM and Kd for CB_2 = 0.18 nM) as the high affinity ligand. Compounds that displaced $[^3H]$CP-55,940 by more than 50% at 1 mM were further analyzed. Receptors were incubated with compounds at 30 °C for 1.5 h at different increasing concentrations using 0.5 nM of $[^3H]$CP-55,940. In all cases, Ki values were calculated applying Cheng-Prusoff equation[1]. Displacement curves were generated using GraphPad, GraphPad Software, Inc., La Jolla, CA, USA.

Preparation of incubation tubes at 30 °C for 90 minutes**:**

- **Blank tube:** 100 µL $[^3H]$CP-55,940 (radioligand) + 100 µL buffer = 200 µL.
- **Control tube:** 100 µL $[^3H]$CP-55,940 + 50 µL CB proteins + 50 µL buffer = 200 µL.
- **WIN-55,212 tube:** 100 µL $[^3H]$CP-55,940 + 50 µL CB proteins + 50 µL WIN-55,212-2 = 200 µL.
- **Competitor (regioisomers) tubes:** 100 µL $[^3H]$CP-55,940 + 50 µL CB proteins + 50 µL competitor at the appropriate concentration = 200 µL total.

[1] Y.-C. Cheng, W.H. Prusoff, Relationship between the inhibition constant (K1) and the concentration of inhibitor which causes 50 percent inhibition (I50) of an enzymatic reaction, Biochem. Pharmacol. 22 (1973) 3099e3108.

Cell cultures.

Human embryonic kidney 293 (HEK293), neoplastic glioblastoma cells (U87MG), and acute promyelocytic leukemia cells (HL-60) were maintained at 5% CO2 and 37 °C in DMEM, EMEM and RPMI medium, respectively containing 10% Fetal Bovine Serum and 100 U/mL penicillin and 100 mg/mL streptomycin.

Cell viability: ([MTT - (3-(4,5-dimethylthiazol-2-yl)-2,5-diphenyltetrazolium bromide) - formazan]

HEK293, U87MG, and HL-60 cells were first quantified to determine the exact number to be used in the MTT cell viability assays, ensuring that absorbance values remained below 1 unit in accordance with the Beer-Lambert(1).

HEK293, U87MG and HL-60 cell lines were seed in a flat-bottom 96-wells plate at a density of 10,000 cells per well. Cells were then incubated with WIN-55,212-2, AM251 and AM630 at various concentrations (0.001 µM to 10 µM) in 200 µL of respective culture medium supplemented with 10% fetal bovine serum (FBS), at 37 °C for 72 hours. Subsequently, 20 µL of 5 mg/mL MTT solution was added to each well, and cells were incubated at 37 °C for 4 h. The resulting formazan crystals were then solubilized with 10% sodium dodecyl sulfate (SDS) in 0.1 mM HCl, followed by overnight incubation at 37 °C. Untreated cells were used as the control, 0.1% of dimethyl sulfoxide (DMSO) as the vehicle control, and 0.2 % of Triton X-100 as a positive control for cell death. IC_{50} toxicity values of WIN-55,212-2 in U87MG cells and HL-60 cell line were determined.

Competition assay between WIN-55,212-2 and AM251 in U87MG cells was conducted under the same conditions described in the previous paragraph. Therefore, WIN-55,212-2 was assayed in the presence of increasing concentrations of the CB_1 inverse agonist/antagonist AM251 (0 µM to 2.0 µM), while WIN-55,212-2 was kept at a fixed concentration of 0.2 µM, based on the previously determined IC_{50}. The stimulation with both CB ligands was performed simultaneously.

Cell viability assays of HEK293, U87MG and HL-60 cell lines using compounds that displaced more than 50% of the radioligand [^{3}H]CP-55,940 at the CB_1 receptor (regioisomers **4a'**, **4b'**, **4d**, **4e'**, **4f**, **4f'**, **5b**, **5b'**, **5d**, **5e**, **5f** and **5f'**) were performed (table 2). All compounds were incubated at 0.1 µM in 200 µL of culture medium supplemented with 10% FBS, in a flat-bottom 96-well plate at a density of 10,000 cells per well, at 37°C for 72 hours. Next, 20 µL of a 5 mg/mL MTT solution was added, and the cells were incubated at 37°C for 4 hours. Then, they were solubilized with 10% sodium dodecyl sulfate (SDS) in 0.1 mM HCl and incubated overnight at 37°C. Formazan absorbance of each well was measured using the EPOCH microplate reader (Biotek, USA) at a wavelength of 570 nm.

---

[1]Mosorov, V., The Lambert-Beer law in time domain form and its application. Applied Radiation and Isotopes, 2017. 128: p. 1-5

Molecular docking experiments

Docking simulations were performed for regioisomers **4d**, **4f**, **5b**, **5d**, **5e**, **5f**, **5f'**, and WIN-55,212-2. Energetic minimization of each molecule were carried out using the LigPrep tool in the program Maestro Schrodinger suite v.11.8 (Schrödinger, LLC)(1). Cannabinoid receptor type 1 structure obtained from cryo-electron microscopy [CB_1 receptor PDBID: 7WV9(2)], were obtained from the Protein Data Bank RCSB-PDB(3). Receptor optimization were performed using the Protein Preparation Wizard available in Maestro software. Water molecules (if applicable) were removed from the protein active site (orthosteric site). Appropriate ionization states for acid and basic amino acid residues, as well as polar hydrogen atoms were considered at physiological pH = 7.4. The enclosing box was configured as a cube with 26 Å length, and the OPLS3e force field was employed for protein energy minimization. The centroid of the selected residue was determined based on the putative orthosteric active site of the CB_1 receptor and its known catalytic amino acids, where the ortostheric ligand CP-55,940 is positioned. The Glide Induced Fit Docking protocol has been used for the final couplings(4). Compounds were punctuated by the Glide scoring function in the extra-precision mode (Glide XP; Schrödinger, LLC)(5, 6) and were filtered on the basis of the best scores, in order to obtain the potential intermolecular interactions between compounds and the receptor, as well as the binding mode and docking descriptors.

Validation of the Binding Pocket via CP-55,940 Redocking

Redocking validation results showing agreement with the crystallographic pose of CP-55,940.

In red: crystallographic CP-55,940 ligand.
In white: redocked CP-55,940 ligand.

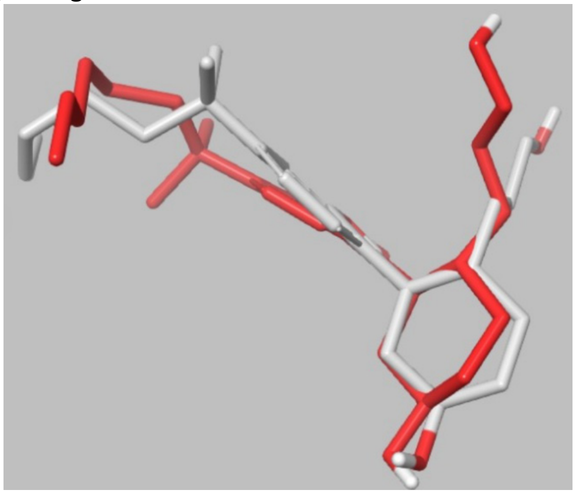

[1] Release, S., 2: Maestro, version 11.8. Schrödinger, LLC, New York. 2018.

[2] Yang, X., et al., Molecular mechanism of allosteric modulation for the cannabinoid receptor CB1. Nature Chemical Biology, 2022. 18(8): p. 831-840.

[3] Westbrook, J. RCSB protein data bank: Structural biology views for basic and applied research. in ACTA CRYSTALLOGRAPHICA A FOUNDATION AND ADVANCES. 2017. INT UNION CRYSTALLOGRAPHY 2 ABBEY SQ, CHESTER, CH1 2HU, ENGLAND.

[4] Sherman, W., et al., Novel procedure for modeling ligand/receptor induced fit effects. Journal of medicinal chemistry, 2006. 49(2): p. 534-553.

[5] Friesner, R.A., et al., *Extra precision glide: Docking and scoring incorporating a model of hydrophobic enclosure for protein− ligand complexes.* Journal of medicinal chemistry, 2006. **49**(21): p. 6177-6196.

[6] DeLano, W.L., *The PyMOL molecular graphics system.* http://www.pymol.org/,2002.
